# Supplementary material for: Estimating the impact of Viamo call-in information services on sexual and reproductive health knowledge, behavior, and outcomes among women of reproductive age: A 2-arm open label randomized controlled trial in Uganda
Source: PLoS One. 2026 Feb 13;21(2):e0342327. doi: 10.1371/journal.pone.0342327 (PMC12904432; doi:10.1371/journal.pone.0342327)
Supplement: S2 File — (DOCX) [file pone.0342327.s002.docx]

**Supplemental Materials to**

*“Estimating the Knowledge and Health Impact of Call-in Information Services Serving Vulnerable Populations: Results from a 2-Arm Open Label Randomized Controlled Trial in Uganda”*

Contents

[S1: Examples of Promotional Messaging by Type 3](#_Toc208413645)

[S2: Survey Items 4](#_Toc208413646)

[Reproductive health knowledge 4](#_Toc208413647)

[Familiarity with, and use of contraceptive methods 4](#_Toc208413648)

[S3 Supplementary Tables 5](#_Toc208413649)

[Supplemental Materials Table ST1: Dates of baseline and endline collection, by district 5](#_Toc208413650)

[Supplemental Materials Table ST2: Percentage of correct answers on knowledge questions: treated and control, baseline vs. endline 6](#_Toc208413651)

[Supplemental Figures 7](#_Toc208413652)

[Supplemental Materials Figure SF 1: Proportion of respondents disagreeing with the statement that condoms cause infertility at baseline and endline by study group 7](#_Toc208413653)

[7](#_Toc208413654)

# S1: Examples of Promotional Messaging by Type

**Generic SMS**: [@[contact.name](http://contact.name)], the new month is here. You have 10 free calls to access information on 161. Call now and enjoy!

**DIV-Content Specific SMS:** [@[contact.name](http://contact.name)], feeling tired or low energy? You might have anemia. Call 161 press 2 then press 1 to learn more about it!

**Financial Promotion:** Hi [@[contact.name](http://contact.name)], join the 161 SUPER-USER club this November by calling 161 for free to earn 30 pts (UGX.3000) We've added even more new content just for you.

**Outbound Call Script:**

Narrator: We have special new content from Cindy Sanyu, exclusively on the 161 service!

English audio clip in Cindy's original voice: **“This is Cindy Sanyu, the king herself.”**

Narrator: Now, Cindy has a message for you...

*Cindy's Music Clip (10 seconds)*

Cindy's audio clip in Cindy's original voice (English): **"I want to tell women that you are built for this. You are built for this. Everything that you need is inside of you. Whoever created you made every part of you ready for what you're doing.”**

Narrator: To listen to more of Cindy's advice for free, call 161 now, or stay on the line to be connected automatically!

# S2: Survey Items

## Reproductive health knowledge

Participants were asked to answer whether the following statements are true or false. We summed the correct answers to form our reproductive health knowledge score

1. Condoms cause infertility in men
2. To put on a condom, you should first unroll it
3. When putting on a condom, it is important to leave space at the tip
4. When using a condom, it is important for the man to pull his penis out right after ejaculation, while it is still stiff
5. Birth control pills (known as The Pill) are taken once every day, whether or not you have sex
6. It is important that women should “take a rest” from the pill every year because the pill builds up in a woman’s body over time
7. If a woman is having side effects with one kind of pill, switching to another type or brand might help
8. After a woman stops taking birth control pills, she is unable to get pregnant for at least six months
9. Emergency contraception must be taken within 1 hour of having unprotected sex
10. Symptoms of gonorrhea in females will appear the day after becoming infected
11. Gonorrhea infection makes it easier to get HIV and other STDs and pass them to sex partners
12. If day 1 is the first day of a woman’s period, she has the greatest chance of becoming pregnant during days 8-­19
13. You can have a sexually transmitted infection without having any symptoms or knowing you are a carrier
14. A woman cannot get pregnant if she urinates immediately after having sex
15. Circumcised males don't get HIV

## Familiarity with, and use of contraceptive methods

Participants were asked if they had a. heard of and b. ever used the following contraceptive methods, and a score out of ten calculated for a. and b. for each participant.

1. Female sterilization
2. Male sterilization
3. Intra-uterine device (IUD)
4. Injectables
5. Implants
6. Female pill
7. Male condoms
8. Female condoms
9. Emergency contraceptive
10. Foam/Jelly/film
11. Beads
12. Withdrawal
13. Breastfeeding

# S3 Supplementary Tables

## Supplemental Materials Table ST1: Dates of baseline and endline collection, by district

|  | Baseline | | Phone Endline | | In person endine | |
| --- | --- | --- | --- | --- | --- | --- |
|  |  | |  | |  | |
|  | **Start** | **End** | **Start** | **End** | **Start** | **End** |
| Kampala | January 26, 2023 | March 6, 2023 | May 8, 2024 | July 21, 2024 | May 13, 2024 | August 4, 2024 |
| Katakwi | December 6, 2022 | January 26, 2023 | May 8, 2024 | July 3, 2024 | May 14, 2024 | July 18, 2024 |
| Madi-Okollo | December 6, 2022 | January 31, 2023 | May 8, 2024 | July 4, 2024 | May 14, 2024 | July 9, 2024 |
| Rwampara | December 6, 2022 | February 14, 2023 | May 8, 2024 | July 6, 2024 | May 14, 2024 | August 4, 2024 |

## Supplemental Materials Table ST2: Percentage of correct answers on knowledge questions: treated and control, baseline vs. endline

|  | **Baseline** | | **Endline** | | **Difference** | | **DID** | | **DID p-value** |
| --- | --- | --- | --- | --- | --- | --- | --- | --- | --- |
|  |  |  |  |  |  |  |  |  |  |
|  |  |  |  |  |  |  |  |  |  |
| Condoms cause infertility | 0.6 | 0.6 | 0.6 | 0.6 | 0.60% | 2.60% | 2.00% | 0.12 | 0.3 |
| Unroll condom | 0.7 | 0.7 | 0.7 | 0.7 | 7.80% | 7.80% | 0.00% | 0 | 0.98 |
| Condoms space | 0.6 | 0.7 | 0.7 | 0.7 | 8.20% | 7.50% | -0.60% | 0 | 0.7 |
| Condom withdraw after six | 0.6 | 0.6 | 0.7 | 0.7 | 13.40% | 12.90% | -0.50% | 0 | 0.78 |
| Pill daily | 0.7 | 0.7 | 0.8 | 0.8 | 6.10% | 8.50% | 2.40% | 0 | 0.16 |
| Pill need break | 0.2 | 0.2 | 0.2 | 0.2 | 1.00% | 2.00% | 1.00% | 0.13 | 0.49 |
| Switching pills | 0.9 | 0.9 | 0.9 | 0.9 | -0.90% | -0.60% | 0.30% | 0.63 | 0.84 |
| Infertility after pill | 0.6 | 0.5 | 0.6 | 0.6 | 5.20% | 5.70% | 0.50% | 0 | 0.75 |
| Emergency one hour | 0.3 | 0.3 | 0.2 | 0.2 | -6.80% | -8.40% | -1.60% | 0 | 0.33 |
| Gonorrhea symptoms | 0.5 | 0.4 | 0.5 | 0.5 | 3.70% | 3.50% | -0.20% | 0.04 | 0.93 |
| Gonorrhea co-infection | 0.8 | 0.8 | 0.9 | 0.9 | 6.50% | 7.00% | 0.40% | 0 | 0.76 |
| Fertile period | 0.8 | 0.8 | 0.8 | 0.8 | 6.20% | 5.80% | -0.40% | 0 | 0.8 |
| STDs without symptoms | 0.8 | 0.8 | 0.8 | 0.8 | 2.10% | 2.60% | 0.40% | 0.04 | 0.74 |
| Urination as contraception | 0.7 | 0.6 | 0.6 | 0.7 | -1.30% | 3.80% | 5.10% | 0 | 0 |
| Circumcised no HIV | 0.8 | 0.8 | 0.8 | 0.8 | 1.20% | 2.30% | 1.10% | 0.05 | 0.46 |

Table notes: simple differences p-value tests for significant changes in the treated group between baseline and endline. DID p-value captures additional improvements relative to the control group (difference in differences).

# Supplemental Figures

## Supplemental Materials Figure SF 1: Proportion of respondents disagreeing with the statement that condoms cause infertility at baseline and endline by study group

### **
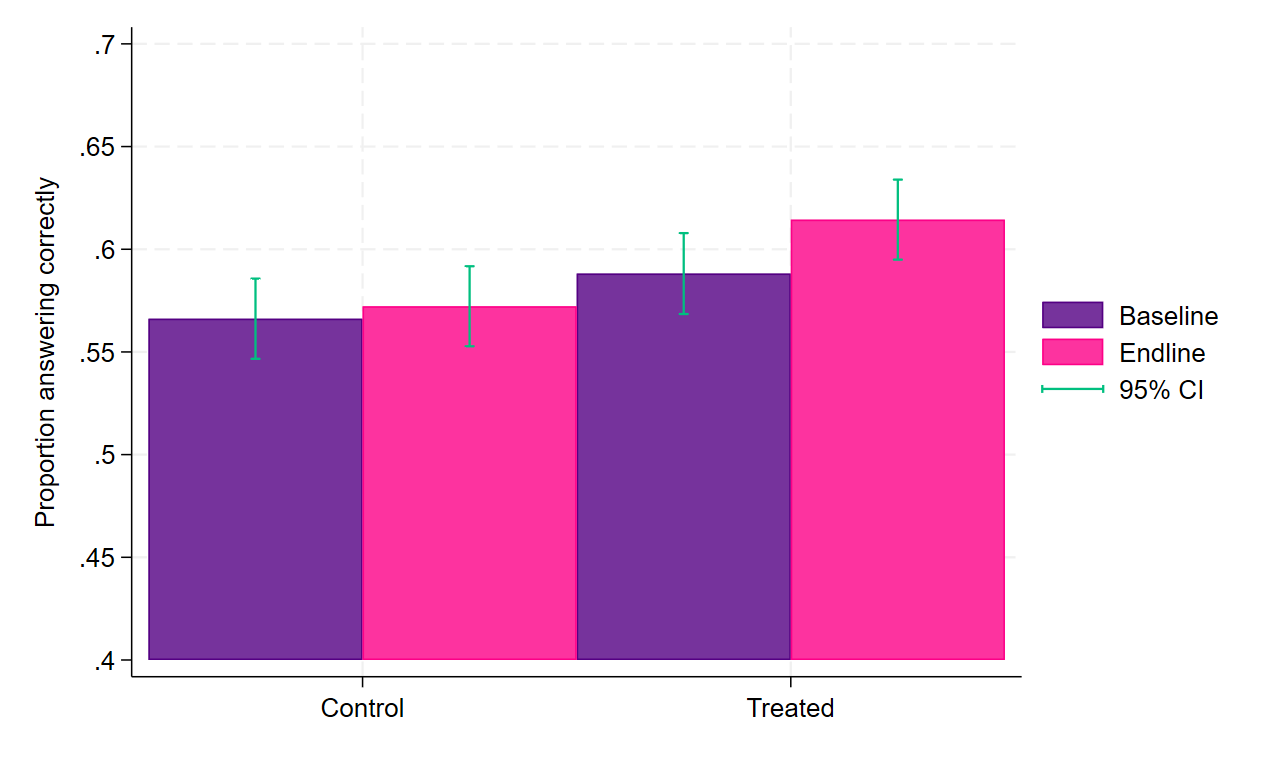
**
