# Supplementary material for: Estimating the impact of Viamo call-in information services on sexual and reproductive health knowledge, behavior, and outcomes among women of reproductive age: A 2-arm open label randomized controlled trial in Uganda
Source: PLoS One. 2026 Feb 13;21(2):e0342327. doi: 10.1371/journal.pone.0342327 (PMC12904432; doi:10.1371/journal.pone.0342327)
Supplement: Attachment — Submitted filename: PONE-D-25-05733_R1-AF.pdf [file pone.0342327.s008.pdf]

PLOS ONE

Estimating the Impact of Viamo Call-in Information Services on Sexual and Reproductive Health Knowledge, Behavior, and Outcomes among women of reproductive age: Results from a 2-Arm Open Label Randomized Controlled Trial in Uganda

--Manuscript Draft--

**Manuscript Number:**	PONE-D-25-05733R1
**Article Type:**	Research Article
**Full Title:**	Estimating the Impact of Viamo Call-in Information Services on Sexual and Reproductive Health Knowledge, Behavior, and Outcomes among women of reproductive age: Results from a 2-Arm Open Label Randomized Controlled Trial in Uganda
**Short Title:**	Improving health knowledge through call-in information services
**Corresponding Author:**	Günther Fink<br>Swiss Tropical Institute: Schweizerisches Tropen- und Public Health-Institut<br>SWITZERLAND
**Keywords:**	Health knowledge; call-in services; randomized trial; Uganda; sexual health; Reproductive Health; Nutrition; Viamo.
**Abstract:**	Background<br><br>Despite substantial increases in access to schooling and to the internet, health information and knowledge remains limited among many vulnerable populations. Given the near universal cell-phone coverage in low- and middle-income countries, call-in services offer an interesting new platform to provide high quality information to targeted populations. In this paper, we assessed the impact of the Viamo 3-2-1 call-in Platform on health knowledge and behavior among women of reproductive age in Uganda.<br><br>Methods<br><br>We conducted a 2-arm open label randomized controlled trial involving 6,000 women, ages 18-49 years, with access to a simple feature cell phone in Uganda. Fifty enumeration areas were randomly selected in Kampala, Madi-Okollo, Rwampara and Katakwi districts. After completion of baseline, women were randomized with equal probability to treatment and control using a tablet-generated random number draw. Study staff introduced treated women to the call-in service and encouraged its use through various incentives and promotional messages. Primary outcomes were sexual and reproductive health knowledge scores as well as iron supplementation in pregnancy. Secondary outcomes included self-reported use of contraception, healthy diets, birth spacing and birth outcomes as well as hemoglobin levels and BMI. Intention to treat as well as per protocol estimates were used to assess program impact. Standard errors were corrected for the cluster-based sampling of women.<br><br>Results<br><br>A total of 6,011 women were enrolled into the study between December 6, 2022 and February 11, 2023; 3,052 were assigned to treatment, 2,959 used as controls. Endline data was collected between May 8, 2024 and August 4, 2024. Overall follow-up rates were 92% in both the treatment and the control group. Treatment increased average sexual and reproductive health knowledge by 0.07 standard deviations (95% CI [0.01, 0.11], p-value = 0.02) and increased the likelihood of taking iron supplements by 4% pts among recently pregnant women ([0.003, 0.08], p-value 0.04). Results for secondary outcomes were mixed, with positive changes for nutritional intake and family planning behaviors and intentions and no impact on hemoglobin levels and BMI. No adverse events or outcomes were reported.<br><br>Conclusions

Powered by Editorial Manager® and ProduXion Manager® from Aries Systems Corporation

	The results of this trial suggest that access to call-in services can increase health knowledge and to some extent also health behaviours. Longer and more intensive program exposure may be needed to yield measurable changes in reproductive outcomes and nutritional status.
**Order of Authors:**	Günther Fink
	Branwen Nia Owen
	Jan Will
	Ilyena Kozain
**Opposed Reviewers:**	
**Response to Reviewers:**	1. Please ensure that your manuscript meets PLOS ONE's style requirements, including those for file naming. The PLOS ONE style templates can be found at https://journals.plos.org/plosone/s/file?id=wjVg/PLOSOne_formatting_sample_main_body.pdf<br>and<br>https://journals.plos.org/plosone/s/file?id=ba62/PLOSOne_formatting_sample_title_authors_affiliations.pdf<br>We have reviewed the manuscript and reformatted the text following the PLOS one style templates.<br>2. Please describe in your methods section how capacity to provide consent was determined for the participants in this study. Please also state whether your ethics committee or IRB approved this consent procedure. If you did not assess capacity to consent please briefly outline why this was not necessary in this case.<br>We read out the consent form to illiterate women and asked them to provide a thumb print to express their explicit consent instead of signing. This process was suggested and approved by local IRB. Women not able to understand the scope of the study as well as women not able or willing to consent using either modality were excluded from the study.<br>3. Please include a complete copy of PLOS' questionnaire on inclusivity in global research in your revised manuscript. Our policy for research in this area aims to improve transparency in the reporting of research performed outside of researchers' own country or community. The policy applies to researchers who have travelled to a different country to conduct research, research with Indigenous populations or their lands, and research on cultural artefacts. The questionnaire can also be requested at the journal's discretion for any other submissions, even if these conditions are not met. Please find more information on the policy and a link to download a blank copy of the questionnaire here: https://journals.plos.org/plosone/s/best-practices-in-research-reporting. Please upload a completed version of your questionnaire as Supporting Information when you resubmit your manuscript.<br>We have completed the inclusivity questionnaire and have uploaded it with the revised documents.<br>4. Thank you for stating the following financial disclosure:<br>"This project was funded through USAID DIV grant number 7200AA21FA00018"<br>Please state what role the funders took in the study. If the funders had no role, please state: "The funders had no role in study design, data collection and analysis, decision to publish, or preparation of the manuscript."<br>If this statement is not correct you must amend it as needed.<br>Please include this amended Role of Funder statement in your cover letter; we will change the online submission form on your behalf.<br><br>This is correct - we have included this statement in the cover letter as suggested.<br><br>5. Thank you for stating the following in the Competing Interests section:<br>"The first, second and third author declare no competing interest. The last author (IK) is currently employed by Viamo, the company operating the service analyzed. She was not involved in the data analysis and did not modify the way the results are presented or described in any way."<br>Please confirm that this does not alter your adherence to all PLOS ONE policies on sharing data and materials, by including the following statement: "This does not alter our adherence to PLOS ONE policies on sharing data and materials." (as detailed online in our guide for authors http://journals.plos.org/plosone/s/competing-interests). If there are restrictions on sharing of data and/or materials, please state these. Please

	note that we cannot proceed with consideration of your article until this information has been declared.<br>Please include your updated Competing Interests statement in your cover letter; we will change the online submission form on your behalf.<br>We have included an updated conflict of interest statement in the cover letter.<br>6. When completing the data availability statement of the submission form, you indicated that you will make your data available on acceptance. We strongly recommend all authors decide on a data sharing plan before acceptance, as the process can be lengthy and hold up publication timelines. Please note that, though access restrictions are acceptable now, your entire data will need to be made freely accessible if your manuscript is accepted for publication. This policy applies to all data except where public deposition would breach compliance with the protocol approved by your research ethics board. If you are unable to adhere to our open data policy, please kindly revise your statement to explain your reasoning and we will seek the editor's input on an exemption. Please be assured that, once you have provided your new statement, the assessment of your exemption will not hold up the peer review process.<br>We have uploaded a complete replication package comprising both the data files and the Stata DO files creating figures and tables as supplementary materials.<br><br>7. We note that Figure 1 in your submission contain [map/satellite] images which may be copyrighted. All PLOS content is published under the Creative Commons Attribution License (CC BY 4.0), which means that the manuscript, images, and Supporting Information files will be freely available online, and any third party is permitted to access, download, copy, distribute, and use these materials in any way, even commercially, with proper attribution. For these reasons, we cannot publish previously copyrighted maps or satellite images created using proprietary data, such as Google software (Google Maps, Street View, and Earth). For more information, see our copyright guidelines: http://journals.plos.org/plosone/s/licenses-and-copyright.<br><br>We require you to either (1) present written permission from the copyright holder to publish these figures specifically under the CC BY 4.0 license, or (2) remove the figures from your submission:<br>This shapefile comes from GADM, which explicitly allows such use: “Using the data to create maps for publishing of academic research articles is allowed. Thus you can use the maps you made with GADM data for figures in articles published by PLoS, Springer Nature, Elsevier, MDPI, etc. You are allowed (but not required) to publish these articles (and the maps they contain) under an open license such as CC-BY as is the case with PLoS journals and may be the case with other open access articles.” (https://gadm.org/license.html),<br>We have added the source to the legend of Figure 1.<br><br>8. Please include captions for your Supporting Information files at the end of your manuscript, and update any in-text citations to match accordingly. Please see our Supporting Information guidelines for more information: http://journals.plos.org/plosone/s/supporting-information.<br>We have relabeled the supplemental material figures following these guidelines.<br><br>9. If the reviewer comments include a recommendation to cite specific previously published works, please review and evaluate these publications to determine whether they are relevant and should be cited. There is no requirement to cite these works unless the editor has indicated otherwise.<br>NA – we did not received any such requests.<br><br><br>Reviewers' comments:<br>Reviewer #1:<br>Thank you for sharing this very interesting study for review. Below are some points that might help the authors improve their manuscript.<br>Thank you for the careful review and very helpful suggestions – we have done our best to address all of them as outlined in further detail below.<br>Introduction:<br>- It would be beneficial to mention a clear hypothesis or research question at the end of the introduction. This will guide readers in understanding how the results align with the

Powered by Editorial Manager® and ProduXion Manager® from Aries Systems Corporation

study's goals.
Thank you for this suggestion. We have added the following sentence to the Introduction:
"Our primary hypothesis was that access to the platform can improve sexual and reproductive health knowledge and attitudes."

Methods:
- The methodology is well-designed and aligns with the CONSORT checklist. However, further details are needed regarding the data collection tools. Specifically:
- Which tools were used to assess the primary and secondary variables?
Apologies for the lack of detail here in the original manuscript. We provide further details on the outcomes variables on page 8 and 9 now, where we write:
"The two primary outcomes of the study were reproductive health knowledge and nutritional supplementation in pregnancy. For reproductive health, we used a 15-item questionnaire of true/false statements (items listed in Supplemental Materials S2). This tool was based on previous studies conducted in sub-Saharan Africa [7] and adapted to match local context. To facilitate interpretation of regression results, we converted the raw scores (number of correct answers out of 15) into z-scores with mean zero and standard deviation one. For antenatal care, we used self-reported iron supplementation as an outcome measure. Secondary outcomes included contraceptive use, prenatal care uptake, hemoglobin level (as a biomarker indicating actual iron intake), body mass index, and birth spacing.

For pregnancy related outcomes, we analyzed two separate sets of women: first, women reporting to be pregnant at the time of the endline survey (currently pregnant), and second, women who completed a pregnancy in the year preceding the endline survey (recently pregnant). For current pregnancies, we analyzed self-report iron supplementation as well as self-reported use of antenatal care services, and whether women indicate the pregnancy was planned. For recently completed pregnancies, we analysed the same variables, but also analysed pregnancy loss and low birth weight (reported weight at birth < 2.5kgs) as additional secondary outcomes.

- Validity and reliability of the measurement tools should be addressed to ensure the robustness of the results.
We added some more details in the Methods section, where we write:
"This tool was based on previous studies conducted in sub-Saharan Africa [7] and adapted to match local context."
For the secondary outcomes, self-reporting biases are definitely a concern which we discuss in the Limitations section of the paper, where we write:
"Third, many of the behaviors measured were self-reported; while we implemented verification measures by taking pictures of antenatal and child health cards and asking women for evidence of contraceptive packaging, such direct evidence was generally only available for a small minority of women, and could thus not be used in the analysis. It is clearly possible that access to the service changes norms and reporting bias, which could explain some of the behavioral changes reported and detected in our analysis. The primary outcome of the study - the reproductive knowledge score - should not be subject to any reporting bias as all women should have the incentive to give the correct answer on the true/false knowledge quiz."
- The term "normalized" is used to describe the data collection tool. This terminology is not common in this context. Consider replacing it with terms like "valid and reliable."
Our apologies – we just meant to say that we created a z-score out of the raw number of correct answers prior to analysis. We have rephrased this as follows in the revised Methods section:
"To facilitate interpretation of regression results, we converted the raw scores (number of correct answers out of 15) into z-scores with mean zero and standard deviation one."
- The terms "reproductive health knowledge" and "antenatal care" seem to overlap or create confusion. Please clarify the distinction between these terms and ensure consistency throughout the manuscript.
Apologies for the confusion: we meant to refer to nutritional supplementation during antenatal care here, and have made this more explicit in the revised version of the manuscript where we write:
"For nutritional supplementation during pregnancy, we used self-reported iron supplementation as an outcome measure"

	- Details regarding exclusion criteria are missing and should be provided.<br>Apologies for the lack of detail here – we have added a new section on inclusion and exclusion criteria, which reads as follows:<br>"..Study eligibility was assessed by trained study enumerators. Women living in selected enumeration areas were eligible for inclusion in the study if they were aged 18-49 years and had access to an Airtel (a mobile network operator) phone number within their household. Given the explicit focus on antenatal care, family planning and post-partum, all women who were pregnant during the listing were automatically invited to join the study; non-pregnant women were randomly selected for the study until the 45 women per cluster target was reached. In clusters with less than 45 women, all women were invited to participate in the study. Women were ineligible if they had used the Viamo platform previously, they planned to move outside of the study area within 12 months of baseline or if they did not speak one of the six languages available on the platform: Ateso, English, Luganda, Lugbara, Luo or Runyakitara."<br><br>- Clarify who assessed eligibility and whether they were blinded during the randomization process.<br>We have added the following information:<br>"Study eligibility was assessed by trained study enumerators."<br>- The manuscript contains 11 tables, which may be excessive. Consider merging some tables or limiting the number to those most essential for clarity. Ensure all tables mentioned in the manuscript are explained in the results section.<br>We agree that there is lots of information in this manuscript, but really believe that all of these results are important. Given that there is no formal limit at PLOS One, we would thus prefer to leave all figures and tables in, and have verified all of them are mentioned in the text. If you feel strongly that some figures should go to the Supplemental Materials we would of course be happy to accommodate that.<br>- There is no mention of the interventional materials used with the experimental group. This information is necessary for understanding the study design and replication in future research.<br>We fully agree with this. We provide a detailed description of the intervention on page 6. The intervention is easy and complex at the same time: it is easy, because essentially women are just invited to call a toll-free number and then get to access content designed for them. The complexity comes from the fact that women can freely navigate content. We show the most commonly accessed items in Table 3, and provide a more detailed example in supplemental materials S1.<br>- The discussion section lacks comparisons with previous studies. Including these would contextualize the findings and add depth.<br>Thank you for this suggestion. We have added the following paragraph to the Disucssion:<br>"In terms of the primary outcome, our results seem consistent with a recent review of mHealth intervention, which suggests that digital information sources can improve sexual and reproductive health knowledge as well as use of sexual and reproductive health services [10]. Compared to the estimates reported for an interactive intervention in Ghana [7, 11] the effect sizes observed here seem smaller, which seems plausible given that not all women engaged with the service on a regular service. "<br>- Discuss the study's limitations and strengths to aid future researchers.<br>We discuss strengths and limitatios at the very end of the Discussion section, where we write:<br>"Despite the very large, stratified and regionally representative sample, the study has some limitations. First, there were not as many pregnant women as anticipated, reducing power on pregnancy-related behaviors and outcomes to some extent. Second, we found that the proportion of women using Airtel phones was substantially lower in some rural districts, making it very difficult to reach enrollment targets in some communities. Even though a more selected sample clearly does not undermine the internal validity of the study (since randomization was done at the individual level), it is not clear whether the final set of women enrolled in the study is fully representative of the larger populations in the four study districts as well as Uganda overall - since the service is currently only available to Airtel users, this study simply focused on women with access to this service. Third, many of the behaviors measured were self-reported; while we implemented verification measures by taking pictures of antenatal and child health cards and asking women for evidence of contraceptive packaging, such direct evidence was generally only available for a small minority of women, and could thus not be used in the analysis. It is clearly possible that access to the service changes

	norms and reporting bias, which could explain some of the behavioral changes reported and detected in our analysis. The primary outcome of the study - the reproductive knowledge score - should not be subject to any reporting bias as all women should have the incentive to give the correct answer on the true/false knowledge quiz. Fourth, the individual-level randomization made the study vulnerable to spillover effects. Qualitative feedback collected at the end of the study suggests that treated women both invited other community members – spouses, friends and other family members – to use the service and actively shared new information with them. Given that some of this shared information likely reached the control group, it seems likely that the impact reported here underestimate the true causal effect of the program. We controlled for direct service use by excluding active users in the per protocol analysis (Table 10) – this does however not capture direct sharing of knowledge among community members."<br><br>- The trial flowchart should be reshaped to align with the original CONSORT 2010 format. available at CONSORT 2010<br>We tried to follow the original template as closely as possible. The main challenge with our study is that we had two follow-up modalities, which required as to make the format a bit wider than usual. We are not sure how to simplify this but are open to suggestions.<br><br><br>Reviewer #2:<br>I commend the authors' work to assess the impact of Viamo's call-in platform on sexual and reproductive health knowledge, behaviors and outcomes.<br>Please find my comments below (pg numbers used in the absence of line numbers):<br>Thank you for the nice feedback and very helpful suggestions – we really appreciate it!<br>-pg 1/27: Title: Please consider revising the title to reflect the aim of this manuscript and the analysis/discussion/conclusion. For instance, "Estimating the Impact of Viamo Call-in Information Services on Sexual and Reproductive Health Knowledge, Behavior, and Outcomes among women of reproductive age: Results from a 2-Arm Open Label Randomized Controlled Trial in Uganda" may be a more appropriate title<br>Thank you for this suggestion – we have adopted this new title.<br>-pg 1/27: Please maintain a consistent spelling for "behavior". In some aspects of the manuscript, this is spelt at "behaviour"<br>Thanks for catching this and our apologies for the inconsistent US/UK spelling – we now use behavior throughout.<br>-pg 2/27: Please cite this source of this sentence, "The platform is free of charge, and currently has over 4 million users in Uganda, and 42 million users in LMICs globally."<br>This was an internal number provided by Viamo; we have replaced this now with an official UNDP estimate (which is slightly lower):<br>"The platform is free of charge, and currently has over 4 million users in Uganda, and 35 million users in LMICs globally [10].»<br>-pg 9/27: "Follow-up rates (completing either the phone or the in person survey or both) were 92% in both arms." Please review this sentence and consider removing it since the estimates do not correspond with Figure 2 and the other sentences in this section of the manuscript better interpret the numbers.<br>Apologies for the lack of clarity here. We used two parallel modalities (with overlapping questions) to reach households at endline. Some of the households completed both and some just one survey – 92% completed either one or both surveys. We have tried to be more clear about this in the revised Results section where we write:<br>"At endline, 87% (n=2,678) of control arm participants, and 86% (n=2,555) of intervention arm participants completed the phone survey. Slightly fewer participants completed the in-person survey (81% of both arms). 92% of participants in both arms completed at least one of the two modalities (i.e. either a phone or an in-person survey)."<br><br>-pg 11/27: Table 2: Please review the results with *** (p-value <0.01). All the P-values are >0.01 and ** (p-value<0.05) would be more appropriate. In addition, some p-values are not significant (0.471 and 0.958) and should not have any asterisk on them.<br>Sorry for the confusion. The numbers in parentheses here are standard errors, and the p-values are all substantially below 0.01 in Table 2. We have made this clear in the revised Table 2 legend that now says:<br>"Notes: All point estimates displayed correspond to estimated mean differences in

outcomes. Numbers in parentheses are robust standard errors.»
The asterisk notation (* for < 0.10, *** for < 0.01) is the default of Stata's outreg package – we would be happy to revise this if you feel strongly about this.
-pg 12/27: Table 3 footnote: How was C2L calculated? It doesn't seem to Times listened / Times reached based on the table. Please add the formula to the footnote.
This rate is simply the proportion of callers who listen to the message all the way to the end – this is automatically registered in the system. We have clarified this in the revised legend of the table, where we now write:
"C2L (caller-to-listener ratio) defines the % completion listening rate of a message, i.e. the proportion of callers who listen to the message all the way to the end."
-pg 13/27: Table 4: Please review the result with *** (p-value <0.01) because ** (p-value<0.05) would be more appropriate for p-value 0.0265 – row 2, column 2.
Once again, we apologize for the lack of clarity. The numbers in parentheses are not p-values but estimated standard errors. We have made this clear in the legend of the table now where we write:
"Coefficients displayed are mean differences with standard errors in parentheses."
-pg 15/27: Table 5: Please review the results with *** (p-value <0.01). All the P-values are >0.01 but <0.05 and ** (p-value<0.05) would be more appropriate.
-pg 16/27: Table 6: Please review the results with * (p-value <0.01). All of them are <0.05 and ** (p-value<0.05) would be more appropriate.
-pg 17/27: Table 7: Please review the results with *** (p-value <0.01) and * (p-value <0.10) and correct them as appropriate. All the p-values should have ** except p-value for 0.0174 (0.00965) which should have-pg 18/27: Table 8: Please include the corresponding asterix for the estimates with significant p-values in this table.
-pg 19/27: Table 9: Please include the corresponding asterix for the estimates with significant p-values in this table.
-pg 20/27: Table 10: Please include the corresponding asterix for the estimates with significant p-values in this table.
-pg 21/27: Table 11: Please review the results with p-values 0.0344*, 0.0331* and 0.0455* (where * is p-value <0.10) and correct them to ** (p-value <0.05) as appropriate. All should have ** except p-value: 0.00965 which should have ***
Once again, we apologize for the lack of clarity. The numbers in parentheses are not p-values but estimated standard errors. We have clearly indicated in the legends of all tables now.
-pgs 22-24: Discussion: Please incorporate existing studies that support or dispute your findings in the discussion section. This section needs a lot more work and is currently only a repetition/elaboration of the results' interpretation.
We have carefully revised the Discussion and added the following next text block to better place the study results into the existing literature:

"In terms of the primary outcome, our results seem consistent with a recent review of mHealth intervention, which suggests that digital information sources can improve sexual and reproductive health knowledge as well as use of sexual and reproductive health services [11]. Compared to the estimates reported for an interactive intervention in Ghana [7, 12] the effect sizes observed here seem smaller, which seems plausible given that not all women engaged with the service on a regular service. Compared to previous studies conducted in other parts of Africa we also observed rather high sexual and reproductive health knowledge at baseline – most women were for example familiar with a range of contraceptive methods (beyond traditional methods, condoms and pills), and myths regarding side effects of contraception were less common that seen in other settings [7, 12] – this likely also contributes to the somewhat smaller knowledge impacts seen here.
In terms of policy, one of the main advantages of the platform used is its relatively low cost – particularly when run at large scale; most of the costs of content development are related to the initial set-up, while operating costs are relatively minor. More research is needed here to investigate the cost-effectiveness of these new mobile technology channels as tools to improve health knowledge among vulnerable populations relative to traditional channels such as radio, tv or community outreach activities. "

Reviewer #3: I struggled to review this paper specifically the results section.
I could not figure out what metric was used to measure impact (such odd ratios).
The measurement of the outcome is not clear or easily understood.

	We apologize for the lack of clarity in the original manuscript. We have added additional details on the outcome measurements in the Tables, and also have added further details in on the main outcome in the revised Methods section, where we write: “For reproductive health, we used a 15-item questionnaire of true/false statements (items listed in Supplemental Materials S2). This tool was based on previous studies conducted in sub-Saharan Africa [7] and adapted to match local context. To facilitate interpretation of regression results, we converted the raw scores (number of correct answers out of 15) into z-scores with mean zero and standard deviation one. For nutritional supplementation during pregnancy, we used self-reported iron supplementation as an outcome measure. Secondary outcomes included contraceptive use, prenatal care uptake, hemoglobin level (as a biomarker indicating actual iron intake), body mass index, and birth spacing.” We hope this makes it easier to understand our analysis now.
**Additional Information:**	
**Question**	**Response**
**Financial Disclosure**<br><br>Enter a financial disclosure statement that describes the sources of funding for the work included in this submission. Review the submission guidelines for detailed requirements. View published research articles from *PLOS ONE* for specific examples.<br><br>This statement is required for submission and **will appear in the published article** if the submission is accepted. Please make sure it is accurate.<br><br>**Funded studies**<br>Enter a statement with the following details:<br>• Initials of the authors who received each award<br>• Grant numbers awarded to each author<br>• The full name of each funder<br>• URL of each funder website<br>• Did the sponsors or funders play any role in the study design, data collection and analysis, decision to publish, or preparation of the manuscript?<br><br>Did you receive funding for this work?	Yes
Please add funding details.<br>as follow-up to "**Financial Disclosure**<br><br>Enter a financial disclosure statement that describes the sources of funding for the work included in this submission. Review the submission guidelines for detailed	This project was funded through USAID DIV grant number 7200AA21FA00018. The funders had no role in study design, data collection and analysis, decision to publish, or preparation of the manuscript.

requirements. View published research articles from *PLOS ONE* for specific examples.<br><br>This statement is required for submission and **will appear in the published article** if the submission is accepted. Please make sure it is accurate.<br><br>**Funded studies**<br>Enter a statement with the following details:<br>• Initials of the authors who received each award<br>• Grant numbers awarded to each author<br>• The full name of each funder<br>• URL of each funder website<br>• Did the sponsors or funders play any role in the study design, data collection and analysis, decision to publish, or preparation of the manuscript?<br><br>Did you receive funding for this work?"	
Please select the country of your main research funder (please select carefully as in some cases this is used in fee calculation).<br>as follow-up to "**Financial Disclosure**<br><br>Enter a financial disclosure statement that describes the sources of funding for the work included in this submission. Review the submission guidelines for detailed requirements. View published research articles from *PLOS ONE* for specific examples.<br><br>This statement is required for submission and **will appear in the published article** if the submission is accepted. Please make sure it is accurate.	UNITED STATES - US

Powered by Editorial Manager® and ProduXion Manager® from Aries Systems Corporation

**Funded studies**<br>Enter a statement with the following details:<br>• Initials of the authors who received each award<br>• Grant numbers awarded to each author<br>• The full name of each funder<br>• URL of each funder website<br>• Did the sponsors or funders play any role in the study design, data collection and analysis, decision to publish, or preparation of the manuscript?<br><br>Did you receive funding for this work?"	
**Competing Interests**<br><br>Use the instructions below to enter a competing interest statement for this submission. On behalf of all authors, disclose any competing interests that could be perceived to bias this work—acknowledging all financial support and any other relevant financial or non-financial competing interests.<br><br>This statement is required for submission and **will appear in the published article** if the submission is accepted. Please make sure it is accurate and that any funding sources listed in your Funding Information later in the submission form are also declared in your Financial Disclosure statement.<br><br>View published research articles from *PLOS ONE* for specific examples.	The first, second and third author declare no competing interest. The last author (IK) is currently employed by Viamo, the company operating the service analyzed. She was not involved in the data analysis and did not modify the way the results are presented or described in any way.

Powered by Editorial Manager® and ProduXion Manager® from Aries Systems Corporation

**NO authors have competing interests**<br><br>Enter: *The authors have declared that no competing interests exist.*<br><br>**Authors with competing interests**<br><br>Enter competing interest details beginning with this statement:<br><br>*I have read the journal's policy and the authors of this manuscript have the following competing interests: [insert competing interests here]*<br><br>* typeset	
**Ethics Statement**<br><br>Enter an ethics statement for this submission. This statement is required if the study involved:<br><br>• Human participants<br>• Human specimens or tissue<br>• Vertebrate animals or cephalopods<br>• Vertebrate embryos or tissues<br>• Field research<br><br>Write "N/A" if the submission does not require an ethics statement.<br><br>General guidance is provided below. Consult the submission guidelines for detailed instructions. **Make sure that all information entered here is included in the Methods section of the manuscript.**	Ethical clearance for the study was obtained from the Mildmay Uganda Research Ethics Committee (MUREC-2022-124), the ethics committee for North-Western Switzerland (AO_2022-00034) as well as by the Innovations for Poverty Action internal review board (#00006083).

**Format for specific study types**<br><br>**Human Subject Research (involving human participants and/or tissue)**<br>• Give the name of the institutional review board or ethics committee that approved the study<br>• Include the approval number and/or a statement indicating approval of this research<br>• Indicate the form of consent obtained (written/oral) or the reason that consent was not obtained (e.g. the data were analyzed anonymously)<br><br>**Animal Research (involving vertebrate animals, embryos or tissues)**<br>• Provide the name of the Institutional Animal Care and Use Committee (IACUC) or other relevant ethics board that reviewed the study protocol, and indicate whether they approved this research or granted a formal waiver of ethical approval<br>• Include an approval number if one was obtained<br>• If the study involved *non-human primates*, add *additional details* about animal welfare and steps taken to ameliorate suffering<br>• If anesthesia, euthanasia, or any kind of animal sacrifice is part of the study, include briefly which substances and/or methods were applied<br><br>**Field Research**<br><br>Include the following details if this study involves the collection of plant, animal, or other materials from a natural setting:<br>• Field permit number<br>• Name of the institution or relevant body that granted permission	
**Data Availability**<br><br>Authors are required to make all data underlying the findings described fully available, without restriction, and from the time of publication. PLOS allows rare exceptions to address legal and ethical concerns. See the PLOS Data Policy and FAQ for detailed information.	Yes - all data are fully available without restriction

A Data Availability Statement describing where the data can be found is required at submission. Your answers to this question constitute the Data Availability Statement and **will be published in the article**, if accepted.<br><br>**Important:** Stating 'data available on request from the author' is not sufficient. If your data are only available upon request, select 'No' for the first question and explain your exceptional situation in the text box.<br><br>Do the authors confirm that all data underlying the findings described in their manuscript are fully available without restriction?	
**Describe where the data may be found in full sentences. If you are copying our sample text, replace any instances of XXX with the appropriate details.**<br><br>• If the data are **held or will be held in a public repository**, include URLs, accession numbers or DOIs. If this information will only be available after acceptance, indicate this by ticking the box below. For example: *All XXX files are available from the XXX database (accession number(s) XXX, XXX.*).<br>• If the data are all contained **within the manuscript and/or Supporting Information files**, enter the following: *All relevant data are within the manuscript and its Supporting Information files.*<br>• If neither of these applies but you are able to provide **details of access elsewhere**, with or without limitations, please do so. For example:<br><br>*Data cannot be shared publicly because of [XXX]. Data are available from the XXX Institutional Data Access / Ethics Committee (contact via XXX) for researchers who meet the criteria for access to confidential data.*<br><br>*The data underlying the results presented in the study are available from (include the name of the third party*	We have uploaded a replication package file which contains all data as well as the code used to create figures and tables.

and contact information or URL).<br>• This text is appropriate if the data are owned by a third party and authors do not have permission to share the data.<br><br>* typeset	
Additional data availability information:	

Powered by Editorial Manager® and ProduXion Manager® from Aries Systems Corporation

Cover Letter

Swiss TPH
Swiss Tropical and Public Health Institute

Prof. Dr. Günther Fink
guenther.fink@swisstph.ch
Direct +41 61 284 8354

Professor of Epidemiology
& Household Economics
and Unit Head

To the
Poshan Thapa, PhD
Academic Editor
PLOS One

Allschwil, September 12th 2025

Re: Estimating the Knowledge and Health Impact of Call-in Information Services Serving Vulnerable Populations

Dear Dr. Thapa,

Thank you for giving us a chance to revise our manuscript titled "*Estimating the Knowledge and Health Impact of Call-in Information Services Serving Vulnerable Populations: Results from a 2-Arm Open Label Randomized Controlled Trial in Uganda*". We are grateful for the very constructive feedback received, and have done our best to address all comments as outlined in further detail in the reply-to-reviewers document attached.

We are also happy to confirm the funder statement:

"The funders had no role in study design, data collection and analysis, decision to publish, or preparation of the manuscript."

The revised conflict of interest statement should read as follows:

"The first, second and third author declare no competing interest. The last author (IK) is currently employed by Viamo, the company operating the service analyzed. She was not involved in the data analysis and did not modify the way the results are presented or described in any way. This does not alter our adherence to PLOS ONE policies on sharing data and materials.""

Please do not hesitate to contact me if you have any questions or require any additional information.

Kind regards,

Günther Fink

Günther Fink, PhD
Head, Household Economics and Health System Research Unit
Swiss Tropical and Public Health Institute
Eckenstein-Geigy Professor of Epidemiology & Household Economics
University of Basel
Tel: +41 (0)61 284 8354
guenther.fink@swisstph.ch

Manuscript

Click here to access/download;Manuscript;Manuscript 26 September 2025.docx

Estimating the Impact of Viamo Call-in Information Services on Sexual and Reproductive Health Knowledge, Behavior, and Outcomes among women of reproductive age: Results from a 2-Arm Open Label Randomized Controlled Trial in Uganda

Günther Fink[1,2*], Branwen Nia Owen[1,2], Jan Will[3], Ilyena Kozain[4]

1 University of Basel, Basel, Switzerland

2 Swiss Tropical and Public Health Institute, Allschwil, Switzerland

3 Innovations for Poverty Action, Kampala, Uganda

4 Viamo, Kampala, Uganda

*** Corresponding author:** Swiss Tropical and Public Health Institute, Kreuzstrasse 2, 4123 Allschwil, Switzerland. Email: guenther.fink@swisstph.ch.

Abstract

Background. Despite substantial increases in access to schooling and to the internet, health information and knowledge remains limited among many vulnerable populations. Given the near universal cell-phone coverage in low- and middle-income countries, call-in services offer an interesting new platform to provide high quality information to targeted populations. In this paper, we assessed the impact of the Viamo 3-2-1 call-in Platform on health knowledge and behavior among women of reproductive age in Uganda.

Methods. We conducted a 2-arm open label randomized controlled trial involving 6,000 women, ages 18-49 years, with access to a simple feature cell phone in Uganda. Fifty enumeration areas were randomly selected in Kampala, Madi-Okollo, Rwampara and Katakwi districts. After completion of baseline, women were randomized with equal probability to treatment and control using a tablet-generated random number draw. Study staff introduced treated women to the call-in service and encouraged its use through various incentives and promotional messages. Primary outcomes were sexual and reproductive health knowledge scores as well as iron supplementation in pregnancy. Secondary outcomes included self-reported use of contraception, healthy diets, birth spacing and birth outcomes as well as hemoglobin levels and BMI. Intention to treat as well as per protocol estimates were used to assess program impact. Standard errors were corrected for the cluster-based sampling of women.

Results. A total of 6,011 women were enrolled into the study between December 6, 2022 and February 11, 2023; 3,052 were assigned to treatment, 2,959 used as controls. Endline data was collected between May 8, 2024 and August 4, 2024. Overall follow-up rates were 92% in both the treatment and the control group. Treatment increased average sexual and reproductive health knowledge by 0.07 standard deviations (95% CI [0.01, 0.11], p-value = 0.02) and increased the likelihood of taking iron supplements by 4% pts among recently pregnant women ([0.003, 0.08], p-value 0.04). Results for secondary outcomes were mixed, with positive

changes for nutritional intake and family planning behaviors and intentions and no impact on hemoglobin levels and BMI. No adverse events or outcomes were reported.

Conclusions. The results of this trial suggest that access to call-in services can increase health knowledge and to some extent also health behaviors. Longer and more intensive program exposure may be needed to yield measurable changes in reproductive outcomes and nutritional status.

Introduction

In low- and middle-income countries (LMIC), access to timely and accurate information in national languages is often limited, particularly among the poor. Whereas the internet is a common source of information in many countries, only 10% of Ugandans, and 36% of Sub-Saharan Africans were estimated to use the internet in 2021; far lower than the global average of 63% [1]. This lack of information access can contribute to poor health decisions, such as inadequate prenatal care and ineffective family planning methods [2].

Public health messaging in LMICs is often disseminated through mass media channels such as radio messages and billboards. These channels have proven to be effective at reaching large numbers of people [3] and increasing knowledge, but have had an insufficient impact on changing behaviors in many areas of public health [4, 5]. Mass media campaigns often fail to create behavior change because, in part, people cannot access the information at a moment of need. Cell phones, abundant in Uganda [6], [illegible]fer opportunities to address this gap, as information can be accessed at any time, from anywhere. Recent evidence suggests that interventions to improve reproductive health knowledge through easy access technologies can effectively improve reproductive health knowledge and use of modern contraception [7-9].

Viamo’s platform was created with the objective of addressing the need for accessible and timely health information. Through an easy to remember dial-in number, callers can obtain up-to-date news as well as key information on topics related to nutrition, maternal and child health, and sexual and reproductive health. The platform is free of charge, and currently has over 4 million users in Uganda, and 35 million users in LMICs globally [10]. With the aim of rigorously assessing the impact of the Viamo’s call-in service on sexual and reproductive health knowledge and related behavior, we conducted a randomized controlled trial (RCT) with women of reproductive age across four districts of Uganda. Our primary hypothesis was that access to the platform can improve sexual and reproductive health knowledge and attitudes.

Methods

Study Design

This study was designed as a two-arm, non-blinded, individual-level randomised controlled trial focusing on women's nutrition and reproductive health. Given that the main intervention – a phone-based information service – was equally available to both arms, we used an encouragement design, actively introducing women in the treated arm to the service and encouraging them to use it throughout the study period. Blinding of the intervention was not possible given the nature of the program.

Study Setting and Participant Recruitment

This study was conducted in four districts of Uganda: Kampala, Katakwi, Madi-Okollo and Rwampara. These four districts were purposely chosen to cover all major regions of Uganda. To recruit women within these four districts, a two-stage random sampling process was followed: first, a list of all enumeration areas in each district was obtained, and 50 enumeration areas were randomly chosen using a simple random number draw created with the Stata statistical software package. In rural areas, enumeration areas corresponded to villages, and in urban areas, to residential blocks. All households within each selected enumeration area were visited to identify eligible women. Fig 1 shows the spatial location of the study districts within Uganda.

Inclusion and exclusion criteria

Study eligibility was assessed by trained study enumerators. Women living in selected enumeration areas were eligible for inclusion in the study if they were aged 18-49 years and had access to an Airtel (a mobile network operator) phone number within their household. Given the explicit focus on antenatal care, family planning and post-partum, all women who were pregnant during the listing were automatically invited to join the study; non-pregnant women were randomly selected for the study until the 45 women per cluster target was reached. In clusters with less than 45 women, all women were invited to participate in the study.

Women were ineligible if they had used the Viamo platform previously, they planned to move outside of the study area within 12 months of baseline or if they did not speak one of the six languages available on the platform: Ateso, English, Luganda, Lugbara, Luo or Runyakitara.

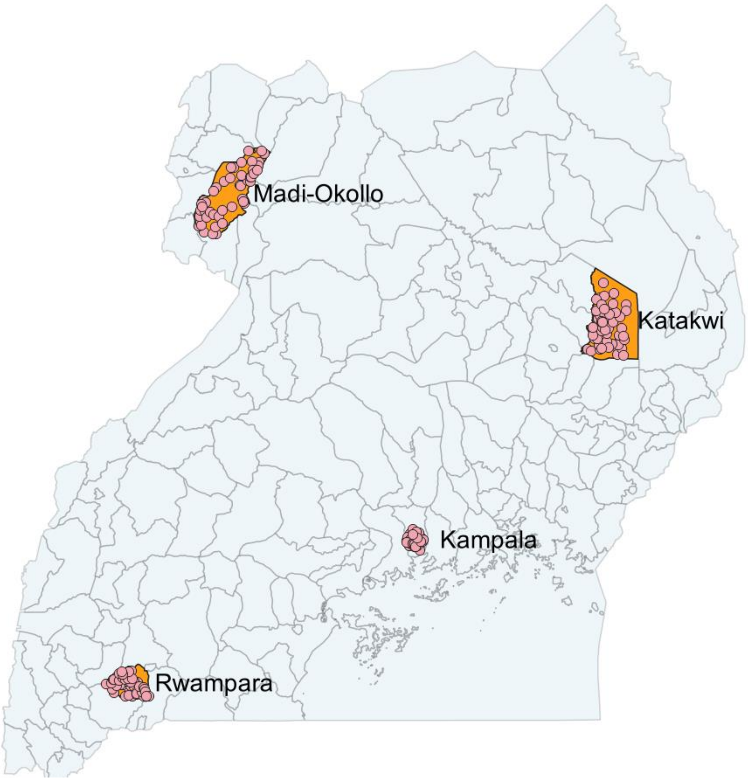

Figure 1: Spatial distribution of study districts (orange shapes) and study clusters (small circles). Uganda administrative shapefiles provided by https://gadm.org/data.html. Figure created by authors.

Intervention

In Uganda, the Viamo platform is available via voice and text channels on all mobile phones with an Airtel number and offers a wide variety of health and other information to all interested callers. To ensure increased service utilization among women randomly selected for the treatment, all treated women in the sample received:

1. A personalized introduction and mock call into platform (at time of baseline interview)
2. A promotional calendar to serve as a physical reminder on how to navigate the service and the benefits of the service (at time of baseline interview)
3. Continued encouragement to use the service in the form of text messages and outbound calls, incentivizing women to call the service.

The original sixty sexual and reproductive, maternal and neonatal health and nutrition key messages launched at the inception of the project were developed in conjunction with subject experts, end users, communication specialists, and the Ugandan Ministry of Health (MOH).

Throughout the implementation period, the key messages (2-4 mins of dialogue) were transformed into stylized dynamic content (7-45 mins), to reiterate critical concepts for enhanced learning and be able to promote new content. The release of DIV-dynamic content formats (dramas, talk shows, and celebrity interviews) was staggered and began in August 2022.

Following enrollment in the study (December 2022 - March 2023), Viamo employed targeted nudges, personalized campaigns and other outreach activities (such as release of new content), with the aim of maintaining and incentivizing usage rates (active calling into the Viamo Platform) among women in the treatment group (see Supplemental Materials S1 for an example).

Study Procedures

Study staff visited selected women at their homes, introduced the study and invited them to participate. The consent form was read aloud and explained when necessary. Literate women signed the consent form and illiterate women marked the form with a thumbprint, and a literate witness was asked to sign on their behalf. We did not assess participants' cognitive capacity to provide consent.

Baseline data was collected via face-to-face interview at each participants' home between December 6, 2022 and March 6, 2023. Upon completion of the baseline survey, treated women were introduced to the service by study staff. Endline data was collected between May 8, 2024 and August 4, 2024 in two separate surveys; a phone survey and a face-to-face in-person survey (see Supplementary Table 1 for dates by district, and Supplementary Table 2 for data collected by phone and in-person interview at endline). During all three surveys, the interviewer recorded responses on a tablet using the SurveyCTO software. Data collectors received training and on-going support and monitoring to ensure data quality. Participants received UGX10,000 (~USD $2.77) of phone credit as a token of gratitude after each survey interview.

Surveys collected data on socio-demographic characteristics, knowledge and use of the Viamo platform, reproductive health knowledge, contraceptive use, pregnancy planning and uptake of antenatal care, pregnancy outcomes, diet and exercise behaviors and select biomarkers.

Randomization

Upon completion of the baseline survey, we randomised arm allocation at the individual level based on a simple number draw generated by the SurveyCTO software. At the end of the survey, the software generated a random number from a uniform distribution [0,1] and allocated all draws > 0.5 to treatment.

Power Calculations

The study was powered to detect a 0.1 standard deviation (SD) difference in standardized knowledge scores (mean 0, SD 1) with power 0.9, assuming an average cluster size of 30, a follow-up rate of 90% and a design effect of 1.3. This effect size was based on an anticipated knowledge score difference of 0.2 SD among compliers (system users), and an average compliance of 10% in the control, and 60% in the treatment arm (a net compliance/usage differential of 50%).

Primary and secondary outcomes

The two primary outcomes of the study were reproductive health knowledge and nutritional supplementation in pregnancy. For reproductive health, we used a 15-item questionnaire of true/false statements (items listed in Supplemental Materials S2). This tool was based on previous studies conducted in sub-Saharan Africa [7] and adapted to match local context. To facilitate interpretation of regression results, we converted the raw scores (number of correct answers out of 15) into z-scores with mean zero and standard deviation one. For nutritional supplementation during pregnancy, we used self-reported iron supplementation as an outcome measure. Secondary outcomes included contraceptive use, prenatal care uptake, hemoglobin level (as a biomarker indicating actual iron intake), body mass index, and birth spacing.

For pregnancy related outcomes, we analyzed two separate sets of women: first, women reporting to be pregnant at the time of the endline survey (currently pregnant), and second, women who completed a pregnancy in the year preceding the endline survey (recently pregnant). For current pregnancies, we analyzed self-report iron supplementation as well as self-reported use of antenatal care services, and whether women indicate the pregnancy was planned. For recently completed pregnancies, we analyzed the same variables, but also analyzed pregnancy loss and low birth weight (reported weight at birth < 2.5kgs) as additional secondary outcomes.

Statistical Analysis

We began our analysis with a comparison of baseline characteristics across arms, as well as a comparison of follow-up rates to assess potential selective attrition. All primary models were estimated intent-to-treat, comparing outcomes between treated and control women at endline. For all outcomes, we estimated both unadjusted models (not including any covariates) and fully adjusted models, where we controlled for age, district, marital status, educational attainment and baseline knowledge variables. We used forest plots to illustrate item level changes for aggregated reproductive health knowledge and contraceptive use scores. In an exploratory analysis, we also estimated per protocol models and stratified models. Per protocol models were restricted to compliant women: specifically, we included only women in the control group who had never called the service, and women in the treatment group who had called the service at least five times. To investigate differential impact on subgroups, we estimated stratified models by age group (<30, 30+), educational attainment group (primary or less, secondary, higher) and district.

Study registration and ethical clearance

The trial was registered on June 03, 2022 at https://www.socialscienceregistry.org/trials/9544. Ethical clearance for the study was obtained from the Mildmay Uganda Research Ethics Committee (MUREC-2022-124), the ethics committee for North-Western Switzerland (AO_2022-00034) as well as by the Innovations for Poverty Action internal review board (#00006083).

Results

Of 54,259 women screened, 29% (n=15,532) were eligible to participate in the study (Fig 2). The most common reason for ineligibility was not having access to an Airtel numb[illegible] A total of 6,01[illegible] women completed the baseline survey and were randomised to the control (n=3,052) or the intervention arm (n=2,959). At endline, 87% (n=2,678) of control arm participants, and 86% (n=2,555) of intervention arm participants completed the phone survey. Slightly fewer participants completed the in-person survey (81% of both arms). 92% of participants in both arms completed at least one of the two modalities (i.e. either a phone or an in-person survey). The most common reason for loss to follow were participants being unreachable by phone, and participants having moved house, in the phone survey and in-person survey, respectively.

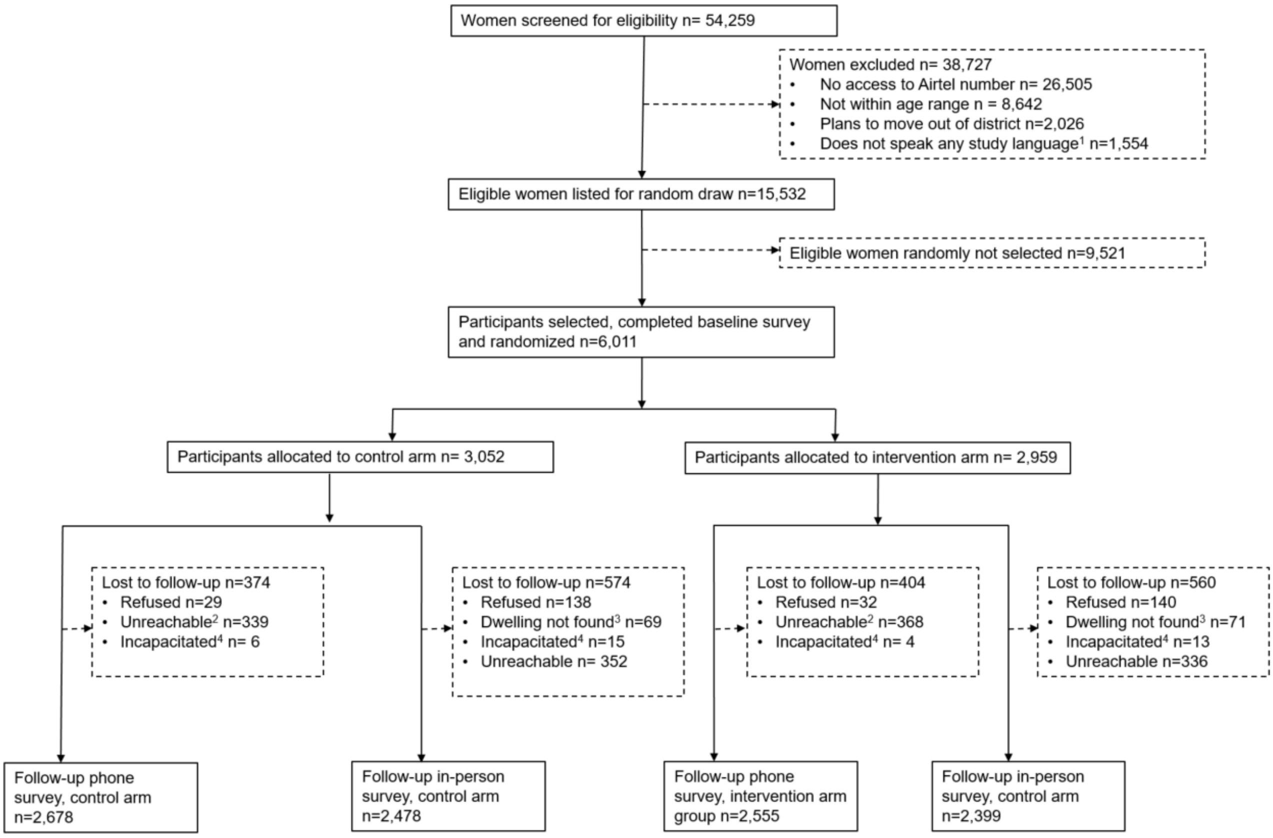

Figure 2: Consort Trial participant flow chart.
Figure 2 Notes: [1] Ateso, English, Luganda, Lugbara, Luo or Runyakitara; [2] Number out of service, phone being switched off, or calls unanswered. [3] Household visited at baseline was not found again. [4] Ill, intoxicated, or deceased

Table 1 shows descriptive statistics for the study sample. Over half were under 30 years old (53%), and a majority were currently married (58%). Twelve percent (12%) of subjects were recruited in Madi-Okollo, 21% in Katakwi, 32% in Rwampara and 35% in Kampala. Fewer than half of women lived in a household with piped water (47%) and very few had health insurance (2%), but most owned their own phone (84%). Most had previously been pregnant (88%) and nearly a seventh were currently pregnant (14%). The trial arms were well-balanced, with all characteristics very similar across arms (Table 1).

Table 1: Sample characteristics overall and by study arm

	Overall (n=6,011)	Control (n=3,052)	Treatment (n=2,959)
Characteristic	N (%)	N (%)	N (%)
Age 18-29	3,159 (53%)	1,609 (53%)	1,550 (52%)
Age 30-39	1,913 (32%)	979 (32%)	934 (32%)

	Overall (n=6,011)	Control (n=3,052)	Treatment (n=2,959)
Age 40+	939 (16%)	464 (15%)	475 (16%)
Currently married	3,503 (58%)	1,756 (58%)	1,747 (59%)
No education	159 (2.6%)	95 (3.1%)	64 (2.2%)
Primary education	3,173 (53%)	1,640 (54%)	1,533 (52%)
Secondary or higher education	2,611 (43%)	1,285 (42%)	1,326 (45%)
Education: other	68 (1.1%)	32 (1.0%)	36 (1.2%)
Speaks English	1,716 (29%)	862 (28%)	854 (29%)
Kampala	2,081 (35%)	1,071 (35%)	1,010 (34%)
Katakwi district	1,256 (21%)	619 (20%)	637 (22%)
Madi-Okollo district	741 (12%)	385 (13%)	356 (12%)
Rwampara district	1,933 (32%)	977 (32%)	956 (32%)
Household has piped water	2,799 (47%)	1,431 (47%)	1,368 (46%)
Household has health insurance	100 (1.7%)	45 (1.5%)	55 (1.9%)
Woman has her own phone	5,056 (84%)	2,527 (83%)	2,529 (85%)
Currently pregnant	867 (14%)	441 (14%)	426 (14%)
Previously pregnant (ever)	5,275 (88%)	2,683 (88%)	2,592 (88%)

Table 2 summarizes overall compliance with treatment and platform use. 50% of women in the control group indicated at endline that they had heard of the Viamo platform compared to 96% in the intervention arm. 20% of women in the control arm reported to have ever used the platform, and 7% reported to have used it in the last month. In the treatment arm, the same was true for 84% and 42% respectively. On average, subjects in the control arm reported four calls to the platform, women in the treatment arm reported an additional 18 calls on average. These numbers also align nicely with the Viamo platform metrics, which recorded an average of four calls for the control group, and an additional 31 calls among women in the treatment group (suggesting that on average women under-reported the number of actual calls they made). In a given month, a treatment user listened to an average of approximately 38 minutes of content on the platform

Table 2: Treatment Compliance

Outcome	Heard of the platform	Ever used the platform	Used the platform in the last month	Total number of self-reported calls to platform [a)]	Total number of registered calls to platform
Treatment effect	0.459***	0.642***	0.346***	18.25***	31.41***
	(0.0192)	(0.0143)	(0.0129)	(0.471)	(0.958)
Control group average	0.499	0.499	0.0709	4.075	4.012

Observations	5,233	5,233	5,233	5,233	5,233
R-squared	0.262	0.412	0.164	0.268	0.271

Notes: All point estimates displayed correspond to estimated mean differences in outcomes. Numbers in parentheses are robust standard errors. *p-value<0.10, **p-value<0.05, ***p-value<0.01, [a)] Self-reported number of calls were top-coded at 51 calls.

Table 3 provides an overview over the most popular content. The most popular single item was the local weather forecast, followed by information on food and general news. On average, 71% of the content listened to by women in the treatment group accessing the platform (n=2,825) was content specifically related to maternal health, sexual and reproductive health, family planning, or nutrition. For the control group members who accessed the platform (n=1,024) only 20% listened to the same thematically relevant content for this study.

Table 3. Top ten most popular messages listened to by treatment group on the platform (Dec 2023 - July 2024)

No	Message Title	Key Themes	Times Reached	Times Listened	Total Number of Listeners	C2L Ratio
1	Dynamic daily weather report	weather	14140	14140	2140	100%
2	To learn about what food keeps us healthy	health, nutrition, anemia	8858	6710	1753	89%
3	Dynamic daily news bulletin	news	5959	4488	1046	86%
4	To know what a healthy weight is	health, nutrition, BMI	4335	3585	1310	92%
5	To learn more about cervical cancer	SRH	4056	3417	1179	91%
6	What is folic acid and why take it?	health, pregnancy	4006	3177	1208	89%
7	What is sex?	puberty, SRH	3852	3500	1513	95%
8	DIV Drama Episode 1: “Family planning for a better future”	FP, SRH	3675	2053	633	77%

No	Message Title	Key Themes	Times Reached	Times Listened	Total Number of Listeners	C2L Ratio
9	To know the four ways to improve your chance for a healthy pregnancy	SRH, pregnancy	3660	2205	1453	78%
10	To learn how iron keeps us strong	nutrition, anemia	3558	2950	1092	89%

Notes: A listener is defined as someone who has listened to at least 75% of a key message. *C2L (caller-to-listener ratio) defines the % completion listening rate of a message, i.e. the proportion of callers who listen to the message all the way to the end. BMI=body mass index, FP=family planning, SRH=sexual and reproductive health

Table 4 shows estimated impact on knowledge. We find a 0.07 SD increase in the primary knowledge outcome score in unadjusted models, and an increase of 0.06 SDs in fully adjusted models. Treatment also increased the average number of reproductive methods women reported to be familiar with by 0.11 points (implying that one in 10 women know one additional method). For nutrition, the treatment reduced the false belief that local herbs could cure anemia by 3 % pts.

Table 4: Impact on Knowledge

Outcome	Reproductive health knowledge z-score	Beliefs that local herbs cure anemia	Number of contraceptive methods familiar with
Treatment effect	0.0708***	-0.0371**	0.116**
(unadjusted)	(0.0265)	(0.0152)	(0.0461)
Adjusted treatment effect	0.0591**	-0.0328**	0.0941**
	(0.0252)	(0.0148)	(0.0455)
Control group average	9.742	0.406	10.33
Survey modality	In person	In person	Phone
Observations	4,877	4,877	5,233

Notes: Table shows estimated impact on primary outcomes. Coefficients displayed are mean differences with standard errors in parentheses. Adjusted models control for age group (age < 20, age 20-34 (ref), age >=35), educational attainment, district, baseline family planning knowledge, baseline hemoglobin level and baseline body mass index. Standard errors were clustered at the enumeration area level. *p-value<0.10, **p-value<0.05, ***p-value<0.01,

Fig 3 shows item-specific changes in sexual and reproductive health knowledge. Largest improvements were observed for myths regarding male condoms causing infertility and post-sex urination preventing pregnancy; small improvements were seen for most questions.

Figure 3: Item-level change in knowledge

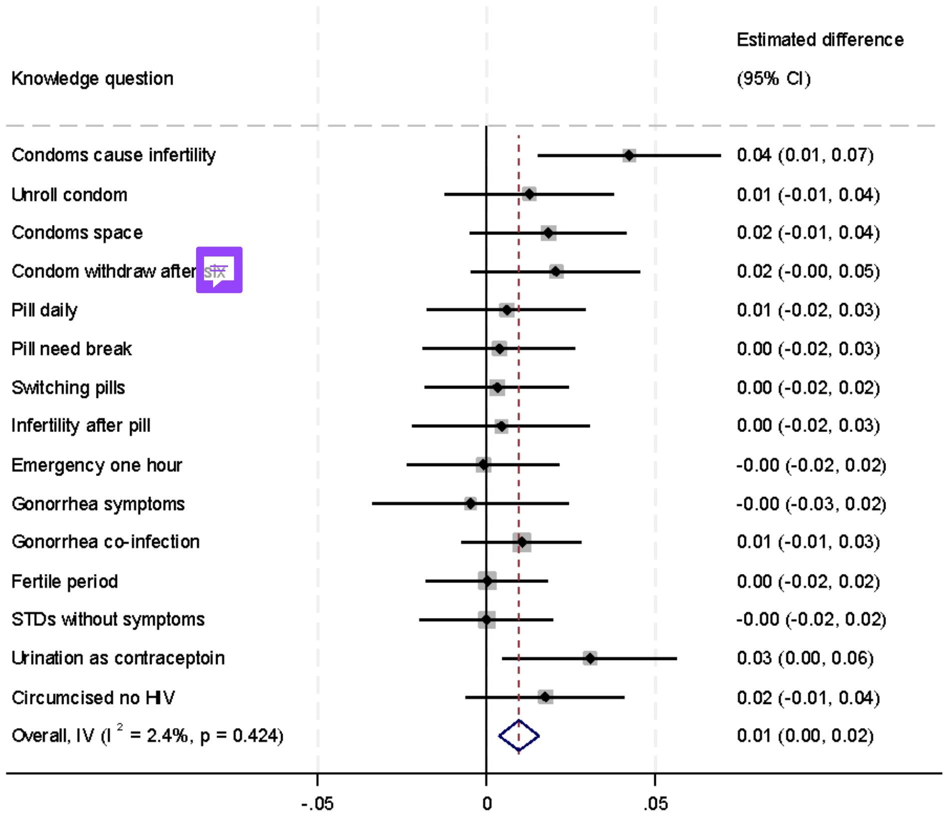

Notes: each point/square on the graphic represents the estimated coefficient obtained from regressing a binary indicator of giving a correct answer to the specific question on treatment - lines illustrate 95% confidence intervals.

Supplemental Materials Figure SF1 further illustrates the observed changes in knowledge for the item with the largest absolute change in knowledge score. Supplemental Materials Table

ST1 shows the proportion of women answering each item correctly at baseline and endline by treatment status for all items investigated.

Table 5 summarizes the estimated impact on sexual behavior as reported in person as well as on the phone. The two survey modalities yielded consistent results with 40% of women using modern contraception and the intervention resulting in an insignificant additional 2% of women using modern contraception. Positive impacts were found for the number of methods ever used (+0.14 methods in adjusted models) and the intention to use modern contraception in the future (+ 5% pt[illegible]

Table 5: Impact on self-reported sexual behavior

Outcome	Currently uses any contraception (in person interview) a)	Currently uses modern contraception (in person report interview) a)	Can show contraception to interviewer	Number of methods ever used (phone)	Currently uses any contraception (phone interview) a)	Currently uses modern contraception (phone)a)	Plans to use modern contraception in the future
Treatment effect	0.0162	0.0193	0.00320	0.164***	0.0189	0.0218	0.050***
(unadjusted)	(0.0147)	(0.0137)	(0.00667)	(0.0449)	(0.0143)	(0.0138)	(0.0124)
Adjusted treatment effect	0.0157	0.0198	0.00384	0.140***	0.0162	0.0192	0.0468***
	(0.0145)	(0.0134)	(0.00638)	(0.0410)	(0.0138)	(0.0133)	(0.0118)
Control group average	0.455	0.396	0.0718	2.956	0.474	0.396	0.396
Survey modality	In person	In person	In person	Phone	Phone	Phone	Phone
Observations	4,877	4,877	4,877	5,233	5,233	5,233	5,233

Notes: Coefficients displayed are mean differences with standard errors in parentheses. Adjusted models control for age group (age < 20, age 20-34 (ref), age >=35), educational attainment, district, baseline family planning knowledge, baseline hemoglobin level and baseline body mass index. Standard errors were clustered at the enumeration area level. *p-value<0.10, **p-value<0.05, ***p-value<0.01. a) The full list of contraceptives is shown in Supplemental Materials S2. Contraceptives not considered as "modern" are beads/calendar method, withdrawal and breastfeeding.

Fig 4 provides further details on current use of contraceptive methods with a focus on methods ever used. The intervention increased the use of implants, pills and condoms by 3-4 % pts – changes on other methods were not significant.

Figure 4: Estimated impact on contraceptives ever used

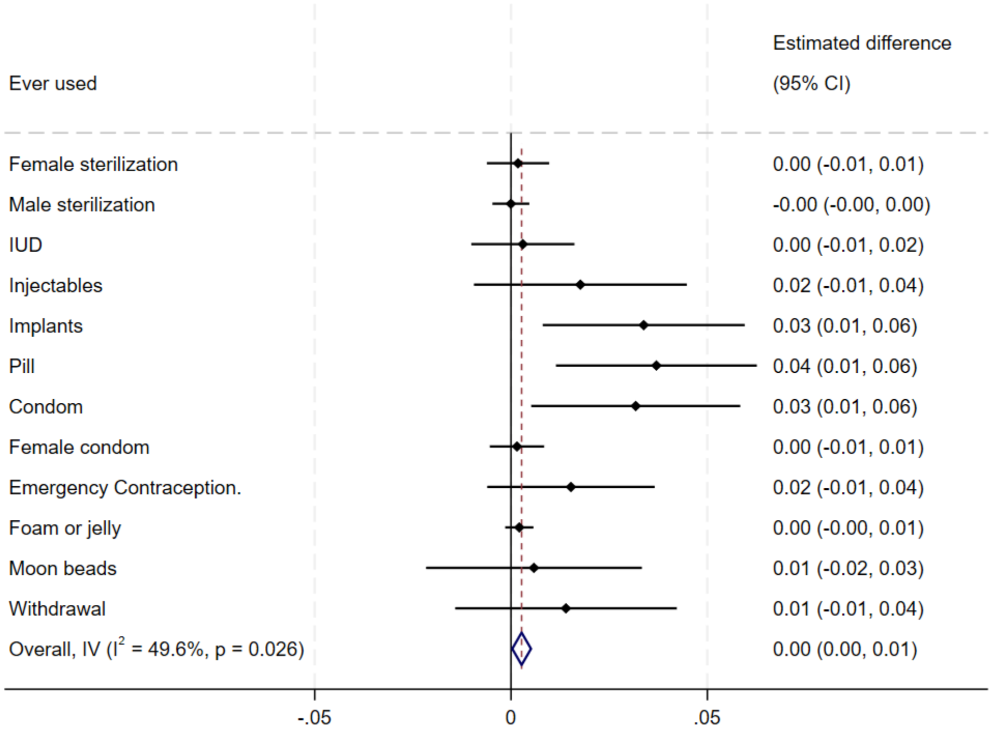

Figure notes: each point/square on the graphic represents the estimated coefficient obtained from regressing a binary indicator of having ever used the method in question on treatment - lines illustrate 95% confidence intervals.

Table 6 shows estimated impact on prenatal care. A total of 398 current pregnancies, as well as 653 pregnancies completed between baseline and endline were analyzed. No differences were found in antenatal care attendance or deworming. Treatment increased the likelihood of taking iron in current pregnancies by 8% pts and the likelihood of taking supplements in recently completed pregnancies by 4% pts [0.3,7.7].

Table 6: Impact on Prenatal Care

Outcome	Attended antenatal care	Currently taking iron	Deworming	Antenatal care for recent pregnancy	Recent pregnancy >=4 visits	Recent pregnancy: iron	Recent pregnancy: deworming
Treatment effect	0.0593	0.0845*	0.0167	0.00955	0.0229	0.0361*	0.0374
(unadjusted)	(0.0449)	(0.0431)	(0.0459)	(0.0161)	(0.0414)	(0.0189)	(0.0314)
Adjusted treatment effect	0.0453	0.0758*	0.00757	0.0156	0.0209	0.040**	0.0369
	(0.0468)	(0.0456)	(0.0483)	(0.0158)	(0.0412)	(0.0189)	(0.0310)
Control group average	0.621	0.605	0.451	0.958	0.454	0.901	0.808
Sample	Women currently pregnant			Women completing pregnancy last year			
Survey modality	In person						
Observations	398	398	398	653	619	653	647

Notes: Coefficients displayed are mean differences with standard errors in parentheses. Adjusted models control for age group (age < 20, age 20-34 (ref), age >=35), educational attainment, district, baseline family planning knowledge, baseline hemoglobin level and baseline body mass index. Standard errors were clustered at the enumeration area level. *p-value<0.10, **p-value<0.05, ***p-value<0.01

Table 7 shows estimated impact on other behavior. The intervention increased women's efforts to eat healthy by 4% pts; interestingly, these efforts were generally not linked to losing weight; on average, treated women were 2.5% less likely to attempt for weight loss, and 3% pts more likely to try to gain weight. In terms of food intake, the treatment appears to have increased the frequency of fruit and vegetable consumption, consistent with the results on healthier diets. This aligns with consistent messaging on increasing healthy habits such as regular activity, the inclusion of nutritious and iron rich foods, and maintaining a healthy body weight.

Table 7: Impact on Other Health Behaviors

Outcome	Aims for health diet	Tried to lose weight	Tried to gain weight	Times [illegible] fruit	Times ate vegetables	Times ate protein	Currently taking iron	Exercised

							supplements	
Treated	0.0407***	-0.025**	0.0355***	0.0686**	0.0484**	-0.00060	0.0156	0.0154
(Unadjusted)	(0.0144)	(0.0108)	(0.0128)	(0.0346)	(0.0245)	(0.0183)	(0.00988)	(0.0124)
Adjusted treatment effect	0.0312**	-0.025**	0.0316**	0.0583*	0.0371*	0.00202	0.0174*	0.0119
	(0.0123)	(0.0106)	(0.0126)	(0.0298)	(0.0213)	(0.0172)	(0.00965)	(0.0118)
Control group average	0.552	0.255	0.242	1.335	1.332	1.760	0.136	0.335
Survey modality	Phone survey							
Observations	5,233	5,233	5,233	5,233	5,232	5,233	5,233	5,233

Notes: Coefficients displayed are mean differences with standard errors in parentheses. Adjusted models control for age group (age < 20, age 20-34 (ref), age >=35), educational attainment, district, baseline family planning knowledge, baseline hemoglobin level and baseline body mass index. Standard errors were clustered at the enumeration area level. *p-value<0.10, **p-value<0.05, ***p-value<0.01.

Table 8 shows estimated impacts on pregnancy and birth outcomes. 41% of currently pregnant women in the control group reported that the current pregnancy was not planned; this proportion was 5%[illegible]s smaller in the treatment group, but not significantly different. Unplanned pregnancies in the past were slightly more common among treated women (but not statistically different). The proportion of previous pregnancies lost as well as the proportion of most recent births born with low birthweight (< 2500 grams) were substantially lower in the treated group – these differences were however not significant statistically due to the small number of observations here.

Table 8: Impact on pregnancy and birth outcomes

Outcome	Pregnancy was unplanned	Birth spacing < 2 years	Previous pregnancy unplanned	Previous pregnancy low	Previous pregnancy lost

				birthweig ht	
Treatment effect	-0.0605	0.0519	0.0260	-0.0143	-0.0301
(unadjusted)	(0.0481)	(0.0546)	(0.0341)	(0.0246)	(0.0216)
Adjusted treatment effect	-0.0531	0.0496	0.0215	-0.0157	-0.0305
	(0.0495)	(0.0516)	(0.0337)	(0.0245)	(0.0209)
Control group average	0.410	0.372	0.261	0.0888	0.0384
Survey modality	In person				
Sample	Currently pregnant		Completed pregnancy recently		
Observations	398	371	653	565	653

Notes: Coefficients displayed are mean differences with standard errors in parentheses. Adjusted models control for age group (age < 20, age 20-34 (ref), age >=35), educational attainment, district, baseline family planning knowledge, baseline hemoglobin level and baseline body mass index. Standard errors were clustered at the enumeration area level. *p-value<0.10, **p-value<0.05, ***p-value<0.01

Table 9 shows estimated impact on biomarkers. While we see some lower rates of anemia among treated women who currently are, or recently were pregnant (Table 9, column 2), these differences are not statistically significant. No changes were found for BMI overall, or any of the categories analyzed (underweight, overweight, obese).

Table 9: Impact on biomarkers

Outcome	Anemic (Hg<12 g/dL)	Anemic (Hg<12 g/dL)	Body mass index	Overweight (BMI >=25, <30)	Obese (BMI >=30)	Underweight (BMI <18.5)
Treatment effect	0.00745	-0.0495	0.183	0.00277	0.0150	0.0120
(unadjusted)	(0.0129)	(0.0315)	(0.165)	(0.0126)	(0.0106)	(0.00826)

Adjusted treatment effect	0.0111	-0.0468	0.0922	0.000613	0.0102	0.0140*
	(0.0127)	(0.0311)	(0.111)	(0.0121)	(0.00831)	(0.00808)
Control group average	0.23	0.23	24.89	0.26	0.15	0.08
Sample	All	Current or recent pregnancy	All	All	All	All
Observations	4,664	992	4,852	4,852	4,852	4,852

Notes: Coefficients displayed are mean differences with standard errors in parentheses. Adjusted models control for age group (age < 20, age 20-34 (ref), age >=35), educational attainment, district, baseline family planning knowledge, baseline hemoglobin level and baseline body mass index. Standard errors were clustered at the enumeration area level. *p-value<0.10, **p-value<0.05, ***p-value<0.01.

Table 10 shows the result of the per protocol analysis, where we compare control women who had never called the platform to treated women who had called the platform at least five times.. As expected, estimated impacts on knowledge scores are higher among compliers; however, these estimates become substantially smaller again when we adjust for women's differences in education and reproductive health knowledge at baseline.

Table 10: Per protocol analysis

Outcome	SRH Knowledge z-score	Currently uses modern method	Uses iron supplements	Used iron supplements	Lost pregnancy	Anemic	Anemic post pregnancy
Treatment effect	0.127***	0.0409**	0.0571	0.000959	-0.00654	0.00586	-0.0496
(Unadjusted)	(0.0332)	(0.0159)	(0.0515)	(0.0102)	(0.00671)	(0.0147)	(0.0345)
Adjusted treatment	0.0650**	0.0206	0.0575	0.0117	-0.00911	0.0168	0.0168
effect	(0.0308)	(0.0155)	(0.0523)	(0.00954)	(0.00668)	(0.0144)	(0.0144)
Control group average	-0.0613	0.374	0.643	0.915	0.0386	0.229	0.389

Sample	All	All	Pregnant now	Pregnant last year		All	Pregnant
Restriction	Compliers only						
Observations	3,561	3,561	292	3,313	3,313	3,400	727

Notes:*Only women in the control group who never called the service, and women in the treatment group who called the service at least five times are included in this table. Coefficients displayed are mean differences with standard errors in parentheses. Adjusted models control for age group (age < 20, age 20-34 (ref), age >=35), educational attainment, district, baseline family planning knowledge, baseline hemoglobin level and baseline body mass index. Standard errors were clustered at the enumeration area level. *p-value<0.10, **p-value<0.05, ***p-value<0.01. SRH=sexual and reproductive health.

Stratified Results

Table 11 shows stratified impact results by age and educational attainment group, as well as by district. For age, we find the weakest effects among women under age 20, and largest impacts among women 35 years and older. Educational level seemed to have little impact, with only slightly larger effects for women with secondary or higher education compared to women with less education. At the district level, impacts vary substantially, with the largest treatment effects in Madi-Okollo (the 0.12 SD increase here is almost twice as large as the effect we see in the sample overall), and the smallest impacts in Rwampara (0.02 SD).

Table 11: Stratified results

Stratum	Age <20	Age 20 - 34	Age 35+	Secondary or higher education	Primary education	Kampala	Katakwi	Madi-Okollo	Rwampara
Treatment effect	0.0213	0.0674*	0.0901*	0.0723*	0.0587*	0.0949**	0.0828*	0.122*	0.0221
	(0.127)	(0.0344)	(0.0500)	(0.0430)	(0.0331)	(0.0431)	(0.0455)	(0.0654)	(0.0517)
Observations	291	2,978	1,608	1,804	3,073	1,472	1,093	650	1,662

Notes: Coefficients displayed are mean differences with standard errors in parentheses. Adjusted models control for age group (age < 20, age 20-34 (ref), age >=35), educational attainment, district, baseline family planning knowledge, baseline hemoglobin level and baseline body mass index. Standard errors were clustered at the enumeration area level. *p-value<0.10, **p-value<0.05, ***p-value<0.01

Harms statement

No harm was reported during the trial.

Discussion

The main objective of this study was to assess the impact of Viamo's call-in platform on health knowledge, behaviors and outcomes. Conceptually, knowledge platforms could result in changes on three distinct levels: changes in health knowledge, changes in health behavior, and changes in health outcomes. The results presented here suggest that changes in knowledge definitely occurred, with treated women displaying improved sex and reproductive health knowledge, being better informed about anemia treatment and more familiar with a larger range of contraceptive methods. This increased familiarity with contraceptive knowledge was also partially visible in contraceptive use. Treated women were on average only marginally (and insignificantly) more likely to use modern contraception, but used a different mix of contraceptives, with higher usage of implants, and somewhat reduced reliance on injectables and calendar methods. In terms of future plans, the intervention seems to have substantially increased the proportion of women who say they will use modern contraception in the future.

For anemia - one of the key health challenges addressed on the platform - the treatment seems to have resulted in an increased uptake of supplements during pregnancy; these higher rates of supplementation also seem consistent with marginally lower rates of anemia among recently pregnant women. The estimated differences in anemia in this subgroup are however not statistically significant, which may partially be due to the relatively small number of women. Despite the high rates of anemia among non-pregnant women, we did not see any change in general protein intake or consumption of iron supplements[illegible] Given this lacking change (and the small proportion of women benefiting from additional supplementation during pregnancy), the insignificant impact on general hemoglobin levels makes sense.

We also see some interesting declines in the rates of pregnancy loss and the prevalence of low-birth-weight births in this subsample, but did not have sufficient statistical power here due to the relatively small number of women completing a pregnancy within the study period.

Given that a larger proportion of women in the treatment group claimed to aim for a healthy diet and to eat more fruit and vegetables, nutrition related content clearly reached its target. Interestingly, the messages received around "healthy body shapes" do not seem to have

triggered a major shift towards eating less and lower BMIs, but seems to have induced a desire to change in both directions, i.e. we see an increasing proportion of women who want to lose weight and an increasing proportion of women who want to gain weight (and hence a smaller proportion of women who want to stay as they are). These shifts in intentions and behaviors are not seen in the anthropometric data - the intervention neither changed average BMI nor the proportions of women classified as underweight, overweight or obese.

In terms of the primary outcome, our results seem consistent with a recent review of mHealth intervention, which suggests that digital information sources can improve sexual and reproductive health knowledge as well as use of sexual and reproductive health services [11]. Compared to the estimates reported for an interactive intervention in Ghana [7, 12] the effect sizes observed here seem smaller, which seems plausible given that not all women engaged with the service on a regular service. Compared to previous studies conducted in other parts of Africa we also observed rather high sexual and reproductive health knowledge at baseline – most women were for example familiar with a range of contraceptive methods (beyond traditional methods, condoms and pills), and myths regarding side effects of contraception were less common that seen in other settings [7, 12] – this likely also contributes to the somewhat smaller knowledge impacts seen here.

In terms of policy, one of the main advantages of the platform used is its relatively low cost – particularly when run at large scale; most of the costs of content development are related to the initial set-up, while operating costs are relatively minor. More research is needed here to investigate the cost-effectiveness of these new mobile technology channels as tools to improve health knowledge among vulnerable populations relative to traditional channels such as radio, tv or community outreach activities.

Despite the very large, stratified and regionally representative sample, the study has some limitations. First, there were not as many pregnant women as anticipated, reducing power on pregnancy-related behaviors and outcomes to some extent. Second, we found that the proportion of women using Airtel phones was substantially lower in some rural districts, making it very difficult to reach enrollment targets in some communities. Even though a more selected sample clearly does not undermine the internal validity of the study (since randomization was done at the individual level), it is not clear whether the final set of women enrolled in the study is fully representative of the larger populations in the four study districts as well as Uganda overall - since the service is currently only available to Airtel users, this

study simply focused on women with access to this service. Third, many of the behaviors measured were self-reported; while we implemented verification measures by taking pictures of antenatal and child health cards and asking women for evidence of contraceptive packaging, such direct evidence was generally only available for a small minority of women, and could thus not be used in the analysis. It is clearly possible that access to the service changes norms and reporting bias, which could explain some of the behavioral changes reported and detected in our analysis. The primary outcome of the study - the reproductive knowledge score - should not be subject to any reporting bias as all women should have the incentive to give the correct answer on the true/false knowledge quiz. Fourth, the individual-level randomization made the study vulnerable to spillover effects. Qualitative feedback collected at the end of the study suggests that treated women both invited other community members – spouses, friends and other family members – to use the service and actively shared new information with them. Given that some of this shared information likely reached the control group, it seems likely that the impact reported here underestimate the true causal effect of the program. We controlled for direct service use by excluding active users in the per protocol analysis (Table 10) – this does however not capture direct sharing of knowledge among community members.

Conclusions

Overall, the results presented here suggest that the platform can effectively improve health knowledge and to some extent also health behaviors among vulnerable populations. These changes in knowledge, awareness and intentions could plausibly also induce changes in health outcomes in the long run; longer-term studies will likely be needed to accurately measure such effects. Future research should also aim to quantify knowledge spillovers within communities as well as the cost-effectiveness of call-in platform like the one analyzed here relative to other communication channels such as TV, radio, posters or community-based activities.

Acknowledgements: We would like to thank all involved staff members at IPA Uganda as well as in the Viamo team for their efforts and continued support of the project. We are also grateful to the more than 6000 study participants for their willingness to participate in the study. Lastly, we would like to thank the USAID Development Innovation Ventures (DIV) team for their input and feedback throughout the project.

References

1. World Bank, *Individuals using the Internet (% of population) | Data.* 2025.
2. D'Souza, P., et al., *Factors influencing contraception choice and use globally: a synthesis of systematic reviews.* The European Journal of Contraception & Reproductive Health Care: The Official Journal of the European Society of Contraception, 2022. **27**(5): p. 364-372.
3. Noar, S.M., *A 10-year retrospective of research in health mass media campaigns: where do we go from here?* Journal of Health Communication, 2006. **11**(1): p. 21-42.
4. Wakefield, M.A., B. Loken, and R.C. Hornik, *Use of mass media campaigns to change health behaviour.* Lancet, 2010. **376**(9748): p. 1261-1271.
5. Stead, M., et al., *Mass media to communicate public health messages in six health topic areas: a systematic review and other reviews of the evidence*. Public Health Research. 2019, Southampton (UK): NIHR Journals Library.
6. Uganda Communications Commission, *Market performance report 2022/2023*. 2023, Uganda Communications Commission.
7. Rokicki, S., et al., *Impact of a Text-Messaging Program on Adolescent Reproductive Health: A Cluster–Randomized Trial in Ghana.* American Journal of Public Health, 2017. **107**(2): p. 298-305.
8. Unger, J.A., et al., *Short message service communication improves exclusive breastfeeding and early postpartum contraception in a low- to middle-income country setting: a randomised trial.* BJOG: an international journal of obstetrics and gynaecology, 2018. **125**(12): p. 1620-1629.
9. Harrington, E.K., et al., *An mHealth SMS intervention on Postpartum Contraceptive Use Among Women and Couples in Kenya: A Randomized Controlled Trial.* American Journal of Public Health, 2019. **109**(6): p. 934-941.
10. UNDP. *Digital X Solution Catalogue*. 2025 10 Sep 2025].
11. Onukwugha, F.I., et al., *The effectiveness and characteristics of mHealth interventions to increase adolescent's use of Sexual and Reproductive Health services in Sub-Saharan Africa: A systematic review.* PLOS ONE, 2022. **17**(1): p. e0261973.
12. Rokicki, S. and G. Fink, *Assessing the reach and effectiveness of mHealth: evidence from a reproductive health program for adolescent girls in Ghana.* BMC Public Health, 2017. **17**(1): p. 969.

Figure 3

Click here to access/download;Figure;Fig3.png

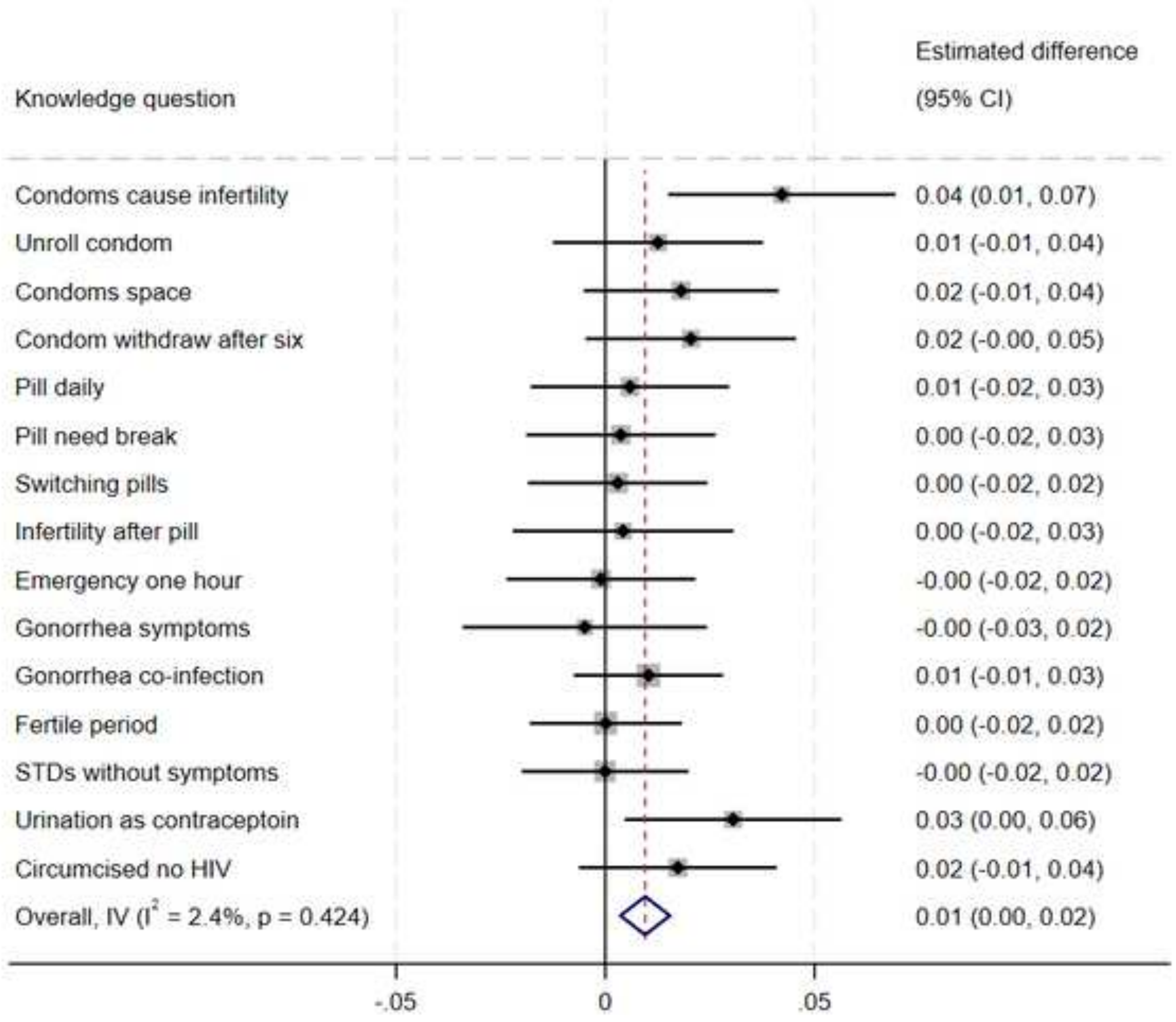

Figure 4

Click here to access/download;Figure;Fig4.png

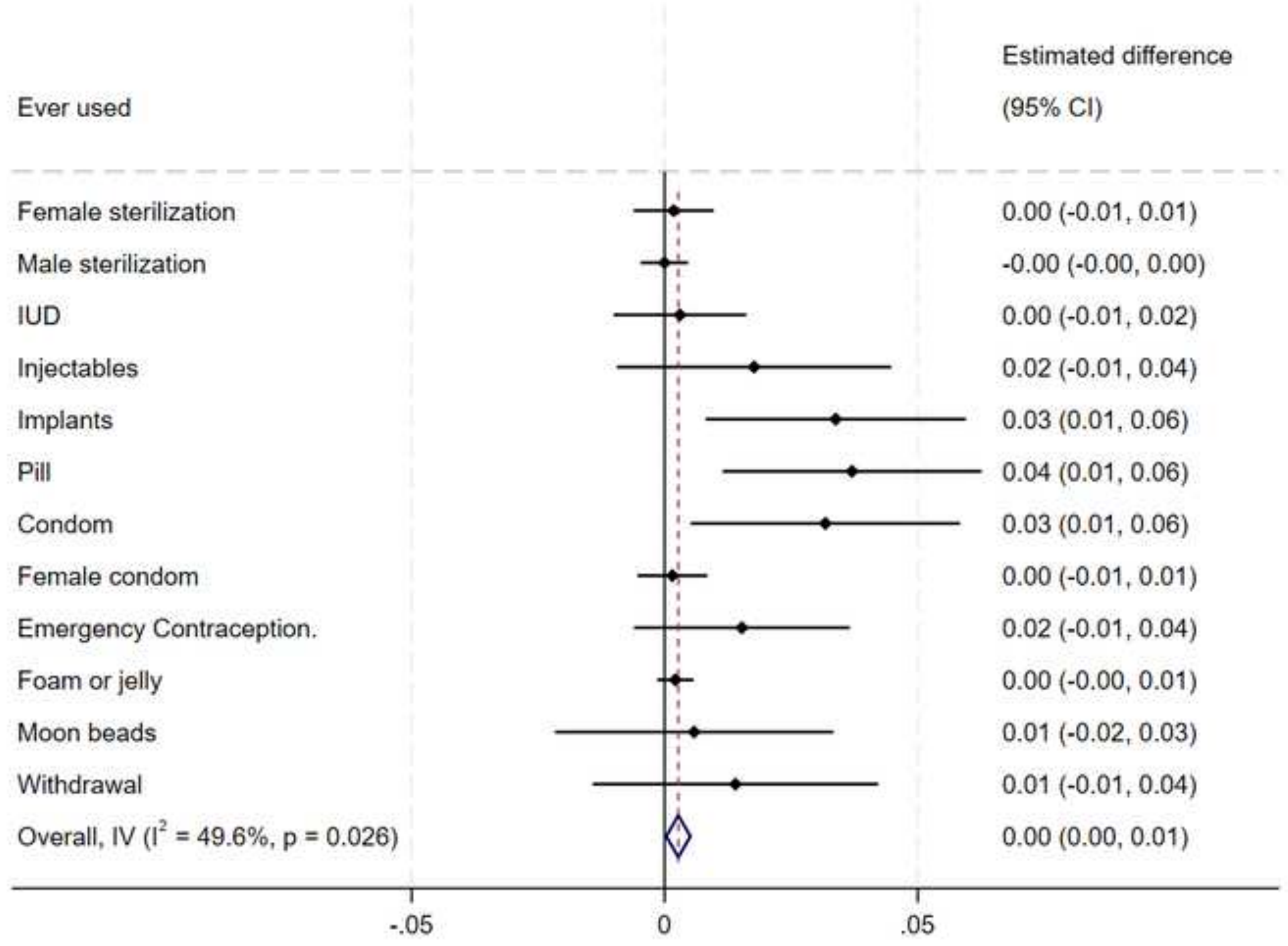

Figure 1

Click here to access/download;Figure;Fig1.jpg

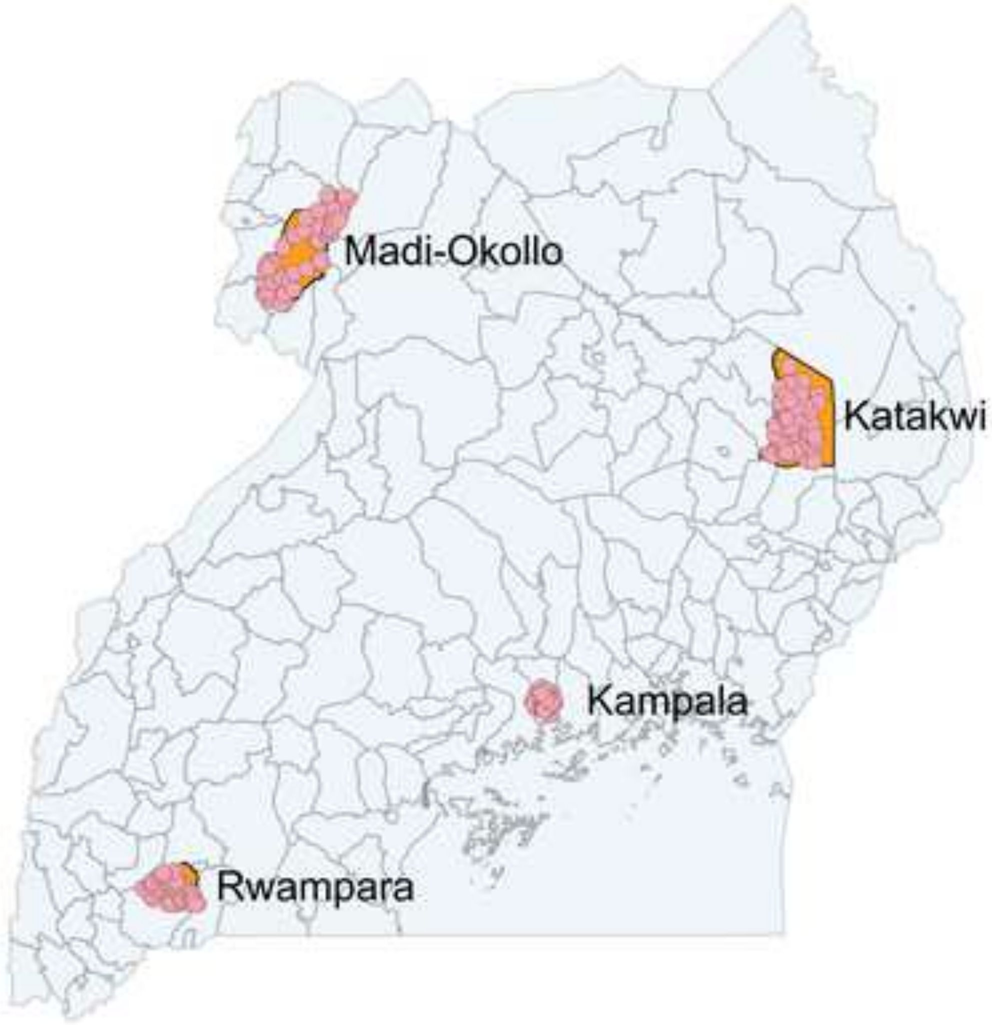

Figure 2

Click here to access/download;Figure;Fig2.png

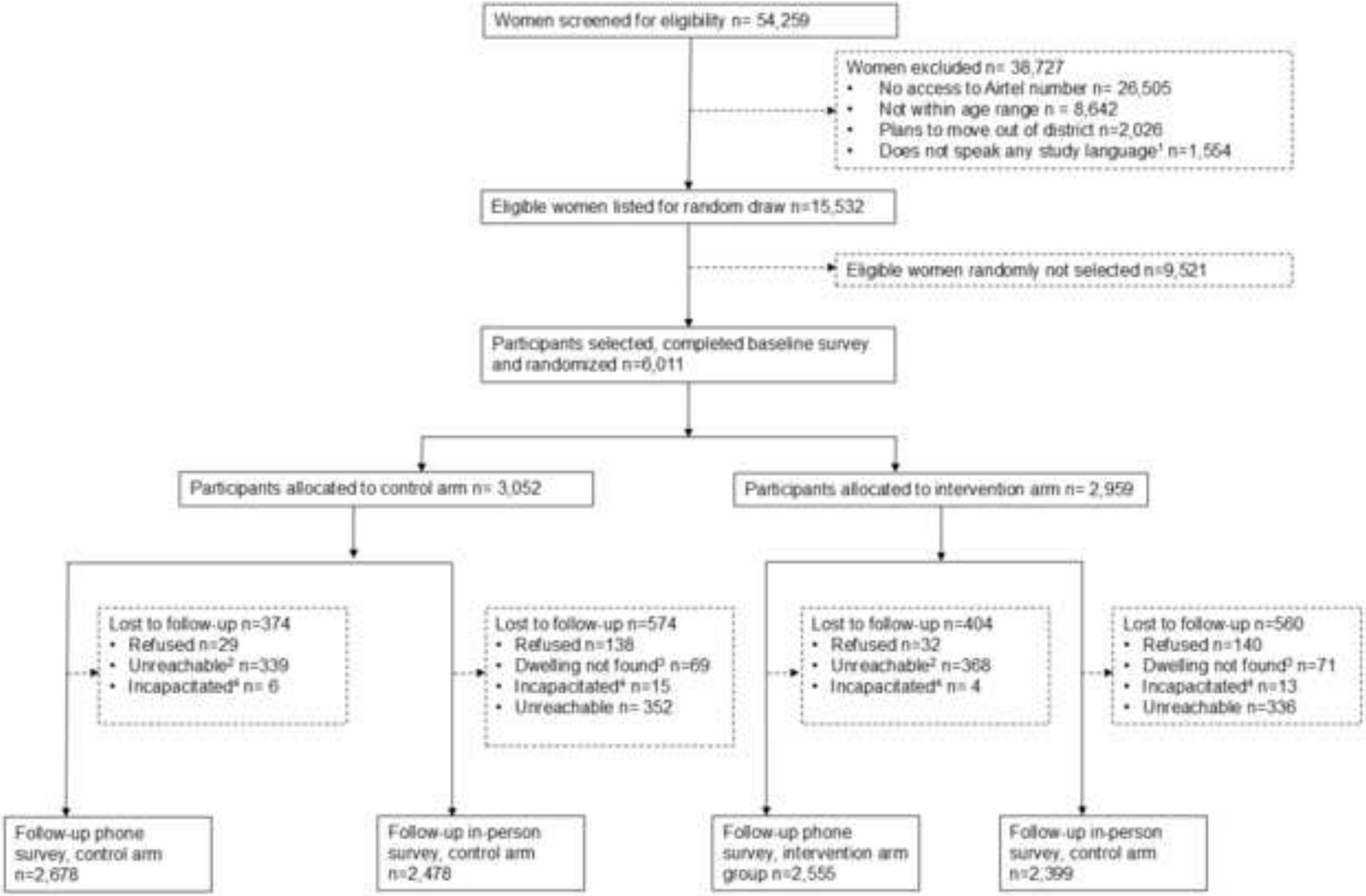

Click here to access/download
Supporting Information
Supplemental Materials 11 Sep 2025.docx

Click here to access/download
Supporting Information
Inclusivity-in-global-research-questionnaire 10 Sep 2025.docx

Click here to access/download
Supporting Information - Compressed/ZIP File Archive
Replication package csv.tar

Click here to access/download

Other

Checklist completed 11 Feb 2025.docx

Click here to access/download
Other
MUREC-2022-124_initial approval.pdf

IRB approval IPA

Click here to access/download

Other

4059_Initial_ApprovalLetter_Expedited.pdf

Click here to access/download
Other
AO_2022-00034_NZ_Ado_pos_220713_en.pdf

~~Title:~~ Estimating the ~~Knowledge and Health~~ Impact of Viamo Call-in Information Services ~~Serving Vulnerable Populations~~on Sexual and Reproductive Health Knowledge, Behavior, and Outcomes among women of reproductive age: Results from a 2-Arm Open Label Randomized Controlled Trial in Uganda

~~Authors:~~ Günther Fink[1,2*], Branwen Nia Owen[1,2], Jan Will[3], Ilyena Kozain[4]

~~Author Affiliations:~~

1 University of Basel, Basel, Switzerland

2 Swiss Tropical and Public Health Institute, Allschwil, Switzerland

3 Innovations for Poverty Action, Kampala, Uganda

4 Viamo, Kampala, Uganda

*** Corresponding author:** Swiss Tropical and Public Health Institute, Kreuzstrasse 2, 4123 Allschwil, Switzerland. Email: guenther.fink@swisstph.ch.

Style Definition: Normal: Font: (Default) Times New Roman, 12 pt

Style Definition: Heading 1: Font: (Default) Times New Roman, 18 pt

Style Definition: Heading 2: Font: (Default) Times New Roman, 16 pt, Bold

Style Definition: Heading 3: Font: Times New Roman

Style Definition: Heading 4: Font: (Default) Times New Roman

Style Definition: Normal (Web)

Style Definition: EndNote Bibliography Title

Style Definition: EndNote Bibliography

Formatted: Centered

Formatted: Font: 16 pt, Bold

Formatted: Font: 16 pt, Bold

Formatted: Font: 16 pt, Bold

Formatted: Font: 16 pt, Bold

Formatted: Font: Not Bold, Superscript

Formatted: Font: 12 pt, Not Bold

Abstract

Formatted: Font: 18 pt

Formatted: Font: 18 pt, Not Bold

Formatted: Heading 1

Background. Despite substantial increases in access to schooling and to the internet, health information and knowledge remains limited among many vulnerable populations. Given the near universal cell-phone coverage in low- and middle-income countries, call-in services offer an interesting new platform to provide high quality information to targeted populations. In this paper, we assessed the impact of the Viamo 3-2-1 call-in Platform on health knowledge and behavior among women of reproductive age in Uganda.

Formatted: Font: Not Bold

Methods. We conducted a 2-arm open label randomized controlled trial involving 6,000 women, ages 18-49 years, with access to a simple feature cell phone in Uganda. Fifty enumeration areas were randomly selected in Kampala, Madi-Okollo, Rwampara and Katakwi districts. After completion of baseline, women were randomized with equal probability to treatment and control using a tablet-generated random number draw. Study staff introduced treated women to the call-in service and encouraged its use through various incentives and promotional messages. Primary outcomes were sexual and reproductive health knowledge scores as well as iron supplementation in pregnancy. Secondary outcomes included self-reported use of contraception, healthy diets, birth spacing and birth outcomes as well as hemoglobin levels and BMI. Intention to treat as well as per protocol estimates were used to assess program impact. Standard errors were corrected for the cluster-based sampling of women.

Formatted: Font: Not Bold

Results. A total of 6,011 women were enrolled into the study between December 6, 2022 and February 11, 2023; 3,052 were assigned to treatment, 2,959 used as controls. Endline data was collected between May 8, 2024 and August 4, 2024. Overall follow-up rates were 92% in both the treatment and the control group. Treatment increased average sexual and reproductive health knowledge by 0.07 standard deviations (95% CI [0.01, 0.11], p-value = 0.02) and increased the likelihood of taking iron supplements by 4% pts among recently pregnant women ([0.003, 0.08], p-value 0.04). Results for secondary outcomes were mixed, with positive

changes for nutritional intake and family planning behaviors and intentions and no impact on hemoglobin levels and BMI. No adverse events or outcomes were reported.

Conclusions. The results of this trial suggest that access to call-in services can increase health knowledge and to some extent also health ~~behaviours~~behaviors. Longer and more intensive program exposure may be needed to yield measurable changes in reproductive outcomes and nutritional status.

Formatted: Font: Not Bold

Introduction

Formatted: Font: 18 pt

Formatted: Font: Not Bold

In low- and middle-income countries (LMIC), access to timely and accurate information in national languages is often limited, particularly among the poor. Whereas the internet is a common source of information in many countries, only 10% of Ugandans, and 36% of Sub-Saharan Africans were estimated to use the internet in 2021; far lower than the global average of 63% [1]. This lack of information access can contribute to poor health decisions, such as inadequate prenatal care and ineffective family planning methods [2].

Formatted: Line spacing: single

Public health messaging in LMICs is often disseminated through mass media channels such as radio messages and billboards. These channels have proven to be effective at reaching large numbers of people [3] and increasing knowledge, but have had an insufficient impact on changing behaviors in many areas of public health [4, 5]. Mass media campaigns often fail to create behavior change because, in part, people cannot access the information at a moment of need. Cell phones, abundant in Uganda [6], offer opportunities to address this gap, as information can be accessed at any time, from anywhere. Recent evidence suggests that interventions to improve reproductive health knowledge through easy access technologies can effectively improve reproductive health knowledge and use of modern contraception [7-9].

Formatted: Line spacing: single

Viamo’s platform was created with the objective of addressing the need for accessible and timely health information. Through an easy to remember dial-in number, callers can obtain up-to-date news as well as key information on topics related to nutrition, maternal and child health, and sexual and reproductive health. The platform is free of charge, and currently has over 4 million users in Uganda, and ~~42 million users in LMICs globally.~~35 million users in LMICs globally [10]. With the aim of rigorously assessing the impact of the Viamo’s call-in service on sexual and reproductive health knowledge and related behavior, we conducted a randomized controlled trial (RCT) with women of reproductive age across four districts of Uganda. Our primary hypothesis was that access to the platform can improve sexual and reproductive health knowledge and attitudes.

Formatted: Line spacing: single

Methods

Study Design

This study was designed as a two-arm, non-blinded, individual-level randomised controlled trial focusing on women's nutrition and reproductive health. Given that the main intervention – a phone-based information service – was equally available to both arms, we used an encouragement design, actively introducing women in the treated arm to the service and encouraging them to use it throughout the study period. Blinding of the intervention was not possible given the nature of the program.

Study Setting and Participant Recruitment

This study was conducted in four districts of Uganda: Kampala, Katakwi, Madi-Okollo and Rwampara. These four districts were purposely chosen to cover all major regions of Uganda. To recruit women within these four districts, a two-stage random sampling process was followed: first, a list of all enumeration areas in each district was obtained, and 50 enumeration areas were randomly chosen using a simple random number draw created with the Stata statistical software package. In rural areas, enumeration areas corresponded to villages, and in urban areas, to residential blocks. All households within each selected enumeration area were visited to identify eligible women. Fig 1 shows the spatial location of the study districts within Uganda.

Inclusion and exclusion criteria

Study eligibility was assessed by trained study enumerators. Women living in selected enumeration areas were eligible for inclusion in the study if they were aged 18-49 years and had access to an Airtel (a mobile network operator) phone number within their household. ~~Women were ineligible if they had used the Viamo platform previously, they planned to move outside of the study area within 12 months of baseline or if they did not speak one of the six languages available on the platform: Ateso, English, Luganda, Lugbara, Luo or Runyakitara.~~

~~In a second and final sampling step, we selected individual women for trial inclusion from a complete list of women in the enumeration unit.~~ Given the explicit focus on antenatal care, family planning and post-partum, all women who were pregnant during the listing were

Formatted: Font: 18 pt
Formatted: Heading 1, Line spacing: single
Formatted: Font: 16 pt
Formatted: Font: Not Bold
Formatted: Font color: Auto
Formatted: Heading 2, Line spacing: single
Formatted: Font: Not Bold, Font color: Auto
Formatted: Line spacing: single

automatically invited to join the study; non-pregnant women were randomly selected for the study until the 45 women per cluster target was reached. In clusters with less than 45 women, all women were invited to participate in the study. Figure 1 shows the spatial location of the study districts within Uganda.

Women were ineligible if they had used the Viamo platform previously, they planned to move outside of the study area within 12 months of baseline or if they did not speak one of the six languages available on the platform: Ateso, English, Luganda, Lugbara, Luo or Runyakitara.

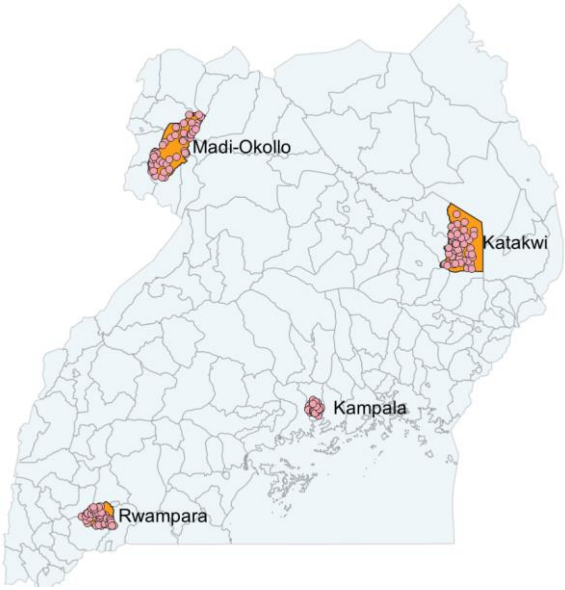

Figure 1: Spatial distribution of study districts (orange shapes) and study clusters (small circles)). Uganda administrative shapefiles provided by https://gadm.org/data.html. Figure created by authors.

Formatted Table

Formatted: Font color: Auto

Intervention

Formatted: Font: 16 pt, Font color: Auto

Formatted: Heading 2

Formatted: Font: Not Bold, Font color: Auto

Formatted: Line spacing: single

In Uganda, the Viamo platform is available via voice and text channels on all mobile phones with an Airtel number and offers a wide variety of health and other information to all interested callers. To ensure increased service utilization among women randomly selected for the treatment, all treated women in the sample received:

1. A personalized introduction and mock call into platform (at time of baseline interview)
2. A promotional calendar to serve as a physical reminder on how to navigate the service and the benefits of the service (at time of baseline interview)
3. Continued encouragement to use the service in the form of text messages and outbound calls, incentivizing women to call the service.

The original ~~60~~ sixty sexual and reproductive, maternal and neonatal health and nutrition key messages launched at the inception of the project were developed in conjunction with subject experts, end users, communication specialists, and the Ugandan Ministry of Health (MOH). Throughout the implementation period, the key messages (2-4 mins of dialogue) were transformed into stylized dynamic content (7-45 mins), to reiterate critical concepts for enhanced learning and be able to promote new content. The release of DIV-dynamic content formats (dramas, talk shows, and celebrity interviews) was staggered and began in August 2022.

Following enrollment in the study (December 2022 - March 2023), Viamo employed targeted nudges, personalized campaigns and other outreach activities (such as release of new content), with the aim of maintaining and incentivizing usage rates (active calling into the Viamo Platform) among women in the treatment group (see Supplemental Materials ~~Part 1~~S1 for an example).

Formatted: Line spacing: single

Study Procedures

Formatted: Font: 16 pt, Font color: Auto

Formatted: Font: Not Bold, Font color: Auto

Formatted: Heading 2, Line spacing: single

Study staff visited selected women at their homes, introduced the study and invited them to participate. The consent form was read aloud and explained when necessary. Literate women signed the consent form and illiterate women marked the form with a thumbprint, and a literate witness was asked to sign on their behalf. We did not assess participants' cognitive capacity to provide consent.

Baseline data was collected via face-to-face interview at each participants' home between December 6, 2022 and March 6, 2023. Upon completion of the baseline survey, treated women were introduced to the service by study staff. Endline data was collected between May 8, 2024 and August 4, 2024 in two separate surveys; a phone survey and a face-to-face in-person survey (see Supplementary Table 1 for dates by district, and Supplementary Table 2 for data collected by phone and in-person interview at endline). During all three surveys, the interviewer recorded responses on a tablet using the SurveyCTO software. Data collectors received training and on-going support and monitoring to ensure data quality. Participants received UGX10,000 (~USD $2.77) of phone credit as a token of gratitude after each survey interview.

Formatted: Line spacing: single

Surveys collected data on socio-demographic characteristics, knowledge and use of the Viamo platform, reproductive health knowledge, contraceptive use, pregnancy planning and uptake of antenatal care, pregnancy outcomes, diet and exercise ~~behaviours~~behaviors and select biomarkers.

Formatted: Line spacing: single

Randomization

Formatted: Font: 16 pt, Font color: Auto

Formatted: Font: Not Bold, Font color: Auto

Upon completion of the baseline survey, we randomised arm allocation at the individual level based on a simple number draw generated by the SurveyCTO software. At the end of the survey, the software generated a random number from a uniform distribution [0,1] and allocated all draws > 0.5 to treatment.

Power Calculations

The study was powered to detect a 0.1 standard deviation (SD) difference in standardized knowledge scores (mean 0, SD 1) with power 0.9, assuming an average cluster size of 30, a follow-up rate of 90% and a design effect of 1.3. This effect size was based on an anticipated knowledge score difference of 0.2 SD among compliers (system users), and an average compliance of 10% in the control, and 60% in the treatment arm (a net compliance/usage differential of 50%).

Primary and secondary outcomes

The two primary outcomes of the study were reproductive health knowledge and nutritional supplementation in pregnancy. For reproductive health, we used a 15-item questionnaire of true/false statements (items listed in Supplemental Materials Part 2), which we normalized to a standard normal variable with mean zero and standard deviation one. For antenatal careS2). This tool was based on previous studies conducted in sub-Saharan Africa [7] and adapted to match local context. To facilitate interpretation of regression results, we converted the raw scores (number of correct answers out of 15) into z-scores with mean zero and standard deviation one. For nutritional supplementation during pregnancy, we used self-reported iron supplementation as an outcome measure. Secondary outcomes included contraceptive use, prenatal care uptake, hemoglobin level (as a biomarker indicating actual iron intake), body mass index, and birth spacing.

For pregnancy related outcomes, we analyzed two separate sets of women: first, women reporting to be pregnant at the time of the endline survey (currently pregnant), and second, women who completed a pregnancy in the year preceding the endline survey (recently pregnant). For current pregnancies, we analyzed self-report iron supplementation, as well as self-reported use of antenatal care services, and whether women indicate the pregnancy was planned. For recently completed pregnancies, we analysedanalyzed the same variables, but also analysedanalyzed pregnancy loss and low birth weight births(reported weight at birth < 2.5kgs) as additional secondary outcomes.

Formatted: Heading 2, Line spacing: single

Formatted: Line spacing: single

Statistical Analysis

We began our analysis with a comparison of baseline characteristics across arms, as well as a comparison of follow-up rates to assess potential selective attrition. All primary models were estimated intent-to-treat, comparing outcomes between treated and control women at endline. For all outcomes, we estimated both unadjusted models (not including any covariates) and fully adjusted models, where we controlled for age, district, marital status, educational attainment and baseline knowledge variables. We used forest plots to illustrate item level changes for aggregated reproductive health knowledge and contraceptive use scores. In an exploratory analysis, we also estimated per protocol models and stratified models. Per protocol models

Formatted: Heading 2, Line spacing: single

were restricted to compliant women: specifically, we included only women in the control group who had never called the service, and women in the treatment group who had called the service at least five times. To investigate differential impact on subgroups, we estimated stratified models by age group (<30, 30+), educational attainment group (primary or less, secondary, higher) and district.

Study registration and ethical clearance

Formatted: Font: 16 pt

Formatted: Heading 2, Line spacing: single

Formatted: Font: Not Bold

The trial was registered on June 03, 2022 at https://www.socialscienceregistry.org/trials/9544. Ethical clearance for the study was obtained from the Mildmay Uganda Research Ethics Committee (MUREC-2022-124), the ethics committee for North-Western Switzerland (AO_2022-00034) as well as by the Innovations for Poverty Action internal review board (#00006083).

Results

Formatted: Font: 18 pt

Formatted: Heading 1, Line spacing: single

Of 54,259 women screened, 29% (n=15,532) were eligible to participate in the study (~~Figure~~Fig 2). The most common reason for ineligibility was not having access to an Airtel number. A total of 6,011 women completed the baseline survey and were randomised to the control (n=3,052) or the intervention arm (n=2,959). ~~Follow-up rates (completing either the phone or the in-person survey or both) were 92% in both arms.~~ At endline, 87% (n=2,678) of control arm participants, and 86% (n=2,555) of intervention arm participants completed the phone survey. Slightly fewer participants completed the in-person survey (81% of both arms). 92% of participants in both arms completed at least one of the two modalities (i.e. either a phone or an in-person survey). The most common reason for loss to follow were participants being unreachable by phone, and participants having moved house, in the phone survey and in-person survey, respectively.

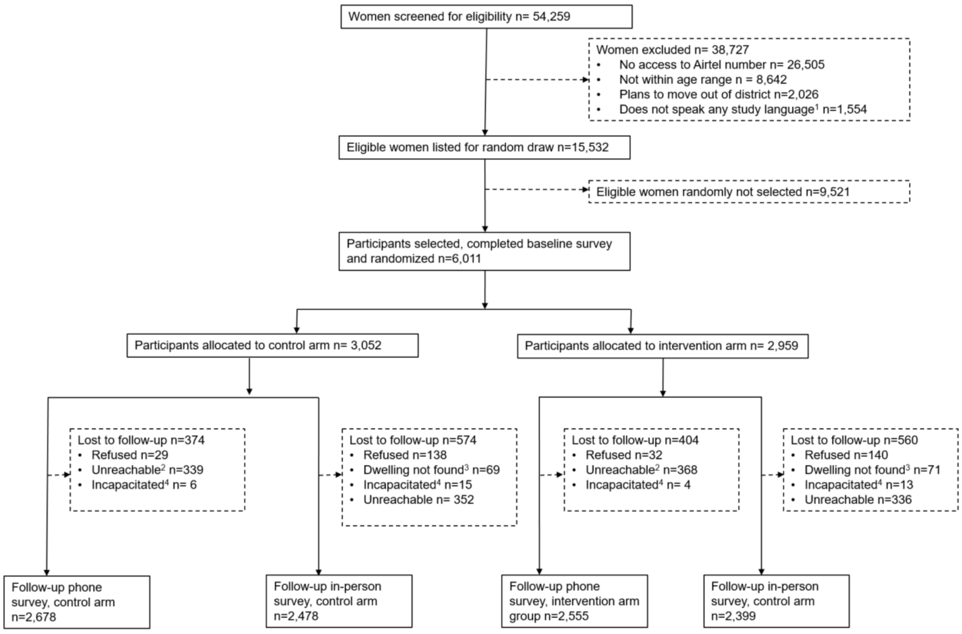

Figure 2: Consort Trial participant flow chart.

Figure 2 Notes: [1] Ateso, English, Luganda, Lugbara, Luo or Runyakitara; [2] Number out of service, phone be[illegible] switched off, or calls unanswered. [3] Household visited at baseline was not found again. [4] Ill, intoxicated, [illegible] deceased

Table 1 shows descriptive statistics for the study sample. Over half were under 30 years old (53%), and a majority were currently married (58%). Twelve percent (12%) of subjects were recruited in Madi-Okollo, 21% in Katakwi, 32% in Rwampara and 35% in Kampala. Fewer than half of women lived in a household with piped water (47%) and very few had health insurance (2%), but most owned their own phone (84%). Most had previously been pregnant (88%) and nearly a seventh were currently pregnant (14%). The trial arms were well-balanced, with all characteristics very similar across arms (Table 1). ~~Characteristics of the sample followed up at endline did not differ systematically from the whole sample at baseline (Supplementary Tables 3-6).~~

Formatted Table

Formatted: Font: 11 pt

Formatted: Font: 11 pt, Font color: Auto

Formatted: Font: 11 pt

Formatted: Font: 11 pt, Font color: Auto

Formatted: Font color: Auto

Formatted: Indent: Left: 0", Right: 0", Border: Top: (No border), Bottom: (No border), Left: (No border), Right: (No border), Between : (No border)

Table 1: Sample characteristics overall and by study arm

	Overall (n=6,011)	Control (n=3,052)	Treatment (n=2,959)
Characteristic	N (%)	N (%)	N (%)
Age 18-29	3,159 (53%)	1,609 (53%)	1,550 (52%)
Age 30-39	1,913 (32%)	979 (32%)	934 (32%)
Age 40+	939 (16%)	464 (15%)	475 (16%)
Currently married	3,503 (58%)	1,756 (58%)	1,747 (59%)
No education	159 (2.6%)	95 (3.1%)	64 (2.2%)
Primary education	3,173 (53%)	1,640 (54%)	1,533 (52%)
Secondary or higher education	2,611 (43%)	1,285 (42%)	1,326 (45%)
Education: other	68 (1.1%)	32 (1.0%)	36 (1.2%)
Speaks English	1,716 (29%)	862 (28%)	854 (29%)
Kampala	2,081 (35%)	1,071 (35%)	1,010 (34%)
Katakwi district	1,256 (21%)	619 (20%)	637 (22%)
Madi-Okollo district	741 (12%)	385 (13%)	356 (12%)
Rwampara district	1,933 (32%)	977 (32%)	956 (32%)
Household has piped water	2,799 (47%)	1,431 (47%)	1,368 (46%)
Household has health insurance	100 (1.7%)	45 (1.5%)	55 (1.9%)
Woman has her own phone	5,056 (84%)	2,527 (83%)	2,529 (85%)
Currently pregnant	867 (14%)	441 (14%)	426 (14%)
Previously pregnant (ever)	5,275 (88%)	2,683 (88%)	2,592 (88%)

Table 2 summarizes overall compliance with treatment and platform use. 50% of women in the control group indicated at endline that they had heard of the Viamo platform compared to 96% in the intervention arm. 20% of women in the control arm reported to have ever used the platform, and 7% reported to have used it in the last month. In the treatment arm, the same was true for 84% and 42% respectively. On average, subjects in the control arm reported four calls to the platform, women in the treatment arm reported an additional 18 calls on average. These numbers also align nicely with the Viamo platform metrics, which recorded an average of four calls for the control group, and an additional 31 calls among women in the treatment group (suggesting that on average women under-reported the number of actual calls they made). In a given month, a treatment user listened to an average of approximately 38 minutes of content on the platform

Table 2: Treatment Compliance

Outcome	Heard of the platform	Ever used the platform	Used the platform in the last month	Total number of self-reported calls to platform a)	Total number of registered calls to platform
Treatment effect	0.459***	0.642***	0.346***	18.25***	31.41***

	(0.0192)	(0.0143)	(0.0129)	(0.471)	(0.958)
Control group average	0.499	0.499	0.0709	4.075	4.012
Observations	5,233	5,233	5,233	5,233	5,233
R-squared	0.262	0.412	0.164	0.268	0.271

~~Notes:~~Notes: All point estimates displayed correspond to estimated mean differences in outcomes. Numbers in parentheses are robust standard errors. *p-value<0.10, **p-value<0.05, ***p-value<0.01, [a)] Self-reported number of calls were top-coded at 51 calls.

Table 3 provides an overview over the most popular content. The most popular single item was the local weather forecast, followed by information on food and general news. On average, 71% of the content listened to by women in the treatment group accessing the platform (n=2,825) was content specifically related to maternal health, sexual and reproductive health, family planning, or nutrition. For the control group members who accessed the platform (n=1,024) only 20% listened to the same thematically relevant content for this study.

Table 3. Top ten most popular messages listened to by treatment group on the platform (Dec 2023 - July 2024)

No	Message Title	Key Themes	Times Reached	Times Listened	Total Number of Listeners	C2L Ratio
1	Dynamic daily weather report	weather	14140	14140	2140	100%
2	To learn about what food keeps us healthy	health, nutrition, anemia	8858	6710	1753	89%
3	Dynamic daily news bulletin	news	5959	4488	1046	86%
4	To know what a healthy weight is	health, nutrition, BMI	4335	3585	1310	92%
5	To learn more about cervical cancer	SRH	4056	3417	1179	91%

Formatted: Font: 10 pt, Font color: Auto
Formatted: Left
Formatted: Font: 10 pt, Font color: Auto
Formatted: Left
Formatted: Font: 10 pt
Formatted: Left
Formatted: Font: 10 pt, Font color: Auto
Formatted: Left
Formatted: Font: 10 pt, Font color: Auto
Formatted: Left
Formatted: Font: 10 pt
Formatted: Font: 10 pt, Font color: Auto
Formatted: Left
Formatted: Font: 10 pt, Font color: Auto
Formatted: Left
Formatted: Superscript
Formatted: Font: 10 pt
Formatted: Font color: Auto
Formatted: Line spacing: single
Formatted: Font color: Auto
Formatted: Left
Formatted: Font: 12 pt
Formatted Table

No	Message Title	Key Themes	Times Reached	Times Listened	Total Number of Listeners	C2L Ratio
6	What is folic acid and why take it?	health, pregnancy	4006	3177	1208	89%
7	What is sex?	puberty, SRH	3852	3500	1513	95%
8	DIV Drama Episode 1: "Family planning for a better future"	FP, SRH	3675	2053	633	77%
9	To know the four ways to improve your chance for a healthy pregnancy	SRH, pregnancy	3660	2205	1453	78%
10	To learn how iron keeps us strong	nutrition, anemia	3558	2950	1092	89%

Formatted: Font: 12 pt

Formatted: Left

Notes: A listener is defined as someone who has listened to at least 75% of a key message.
*C2L (caller-to-listener ratio) defines the % completion listening rate of a message, i.e. the proportion of callers who listen to the message all the way to the end. BMI=body mass index, FP=family planning, SRH=sexual and reproductive health

Formatted: Font color: Auto

Formatted: Font color: Auto

Formatted: Font color: Auto

Table 4 shows estimated impact on knowledge. We find a 0.07 SD increase in the primary knowledge outcome score in unadjusted models, and an increase of 0.06 SDs in fully adjusted models. Treatment also increased the average number of reproductive methods women reported to be familiar with by 0.11 points (implying that one in 10 women know one additional method). For nutrition, the treatment reduced the false belief that local herbs could cure anemia by 3 % pts.

Formatted: Line spacing: single

Table 4: Impact on Knowledge

Outcome	Reproductive health knowledge z-score	Beliefs that local herbs cure anemia	Number of contraceptive methods familiar wi[illegible]
Treatment effect	0.0708***	-0.0371**	0.116**
(unadjusted)	(0.0265)	(0.0152)	(0.0461)

Formatted: Font: 10 pt, Font color: Auto

Formatted: Left

Formatted Table

Formatted: Font color: Auto

Formatted: Left

Formatted: Font: 10 pt, Font color: Auto

Formatted: Font: 10 pt, Font color: Auto

Adjusted treatment effect	0.0591**	-0.0328**	0.0941**
	(0.0252)	(0.0148)	(0.0455)
Control group average	9.742	0.406	10.33
Survey modality	In person	In person	Phone
Observations	4,877	4,877	5,233

Notes: Table shows estimated impact on primary outcomes. Coefficients displayed are mean differences with standard errors in parentheses. Adjusted models control for age group (age < 20, age 20-34 (ref), age >=35), educational attainment, district, baseline family planning knowledge, baseline hemoglobin level and baseline body mass index. Standard errors were clustered at the enumeration area level. *p-value<0.10, **p-value<0.05, ***p-value<0.01,

~~Figure~~Fig 3 shows item-specific changes in sexual and reproductive health knowledge. Largest improvements were observed for myths regarding male condoms causing infertility and post-sex urination preventing pregnancy; small improvements were seen for most questions.

Figure 3: Item-level change in knowledge

Formatted: Font: 10 pt, Font color: Auto
Formatted: Font: 10 pt
Formatted: Left
Formatted: Centered
Formatted: Font: 10 pt, Font color: Auto
Formatted: Font: 10 pt, Font color: Auto
Formatted: Left
Formatted: Font: 10 pt, Font color: Auto
Formatted: Left
Formatted: Font: 10 pt
Formatted: Centered
Formatted: Font: 10 pt, Font color: Auto
Formatted: Font: 10 pt, Font color: Auto
Formatted: Font: 10 pt, Font color: Auto
Formatted: Font: 10 pt, Font color: Auto
Formatted: Left
Formatted: Font: 11 pt
Formatted: Space After: 6 pt
Formatted: Font: 12 pt
Formatted: Line spacing: single

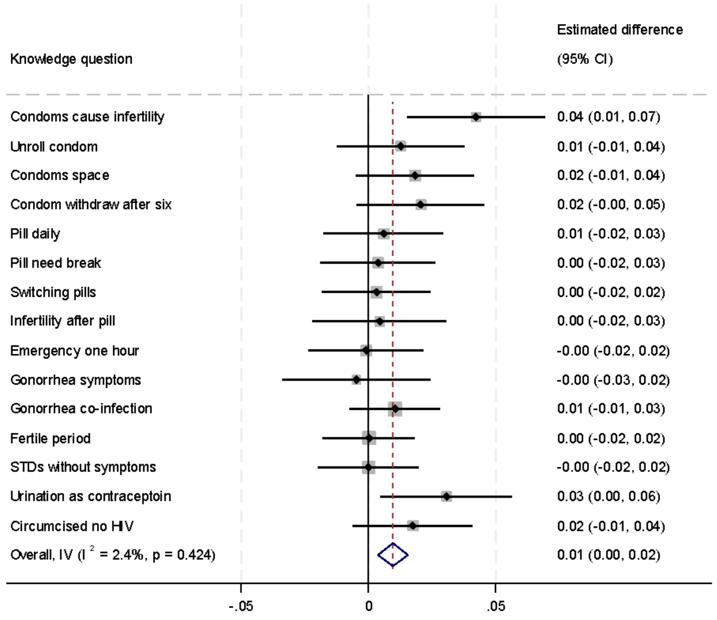

~~Figure 3 notes~~Notes: each point/square on the graphic represents the estimated coefficient obtained from regressing a binary indicator of giving a correct answer to the specific question on treatment - lines illustrate 95% confidence intervals.

Formatted: Font: 11 pt

Supplemental Materials Figure ~~SF 1~~SF1 further illustrates the observed changes in knowledge for the item with the largest absolute change in knowledge score. Supplemental Materials Table ~~S2~~ST1 shows the proportion of women answering each item correctly at baseline and endline by treatment status for all items investigated.

Formatted: Line spacing: single

Table 5 summarizes the estimated impact on sexual ~~behaviour~~behavior as reported in person as well as on the phone. The two survey modalities yielded consistent results with 40% of women using modern contraception and the intervention resulting in an insignificant additional 2% of women using modern contraception. Positive impacts were found for the number of methods ever used (+0.14 methods in adjusted models) and the intention to use modern contraception in the future (+ 5% pts).

Formatted: Line spacing: single

Table 5: Impact on self-reported sexual ~~behaviour~~behavior

Outcome	Currently uses any contraception (in person interview) a)	Currently uses modern contraception (in person report interview) a)	Can show contraception to interviewer	Number of methods ever used (phone)	Currently uses any contraception (phone interview) a)	Currently uses modern contraception (phone)a)	Plans to modern contracepti[illegible] in the futur[illegible]
Treatment effect	0.0162	0.0193	0.00320	0.164***	0.0189	0.0218	0.050***
(unadjusted)	(0.0147)	(0.0137)	(0.00667)	(0.0449)	(0.0143)	(0.0138)	(0.0124)
Adjusted treatment effect	0.0157	0.0198	0.00384	0.140***	0.0162	0.0192	0.0468***
	(0.0145)	(0.0134)	(0.00638)	(0.0410)	(0.0138)	(0.0133)	(0.0118)
Control group average	0.455	0.396	0.0718	2.956	0.474	0.396	0.396
Survey modality	In person	In person	In person	Phone	Phone	Phone	Phone
Observations	4,877	4,877	4,877	5,233	5,233	5,233	5,233

Notes: Coefficients displayed are mean differences with standard errors in parentheses. Adjusted models control for age group (age < 20, age 20-34 (ref), age >=35), educational attainment, district, baseline family planning knowledge, baseline hemoglobin level and baseline body mass index. Standard errors were clustered at the enumeration area level. *p-value<0.10, **p-value<0.05, ***p-value<0.01. a) The full list of contraceptives is shown in Supplemental Materials ~~Section 1~~S2. Contraceptives not considered as "modern" are beads/calendar method, withdrawal and breastfeeding.

~~Figure~~Fig 4 provides further details on current use of contraceptive methods~~.~~ with a focus on methods ever used. The intervention increased the use of implants, pills and condoms by ~~approximately 2~~3-4 % pts~~, and lowered the use of beads (calendar method) and injectables marginally, resulting in the small insignificant overall change seen in Table 5.~~ – changes on other methods were not significant.

Figure 4: Estimated impact on ~~current contraceptive use~~contraceptives ever used

Formatted: Font color: Auto
Formatted: Font color: Auto
Formatted: Font color: Auto
Formatted: Left
Formatted: Font color: Auto
Formatted: Font color: Auto
Formatted: Font color: Auto
Formatted: Font color: Auto
Formatted: Font color: Auto
Formatted: Font color: Auto
Formatted: Left
Formatted Table
Formatted: Font color: Auto
Formatted: Left
Formatted: Font color: Auto
Formatted: Font color: Auto
Formatted: Left
Formatted: Font color: Auto
Formatted: Left
Formatted: Left
Formatted: Font color: Auto
Formatted: Left
Formatted: Font color: Auto
Formatted: Left
Formatted: Font color: Auto
Formatted: Left
Formatted: Left
Formatted: Font color: Auto
Formatted: Left
Formatted: Font color: Auto
Formatted: Left
Formatted: Font color: Auto
Formatted: Left
Formatted: Font: Italic
Formatted: Font: 11 pt
Formatted: Font: (Default) Garamond, 11 pt
Formatted: Font: 12 pt
Formatted: Line spacing: single

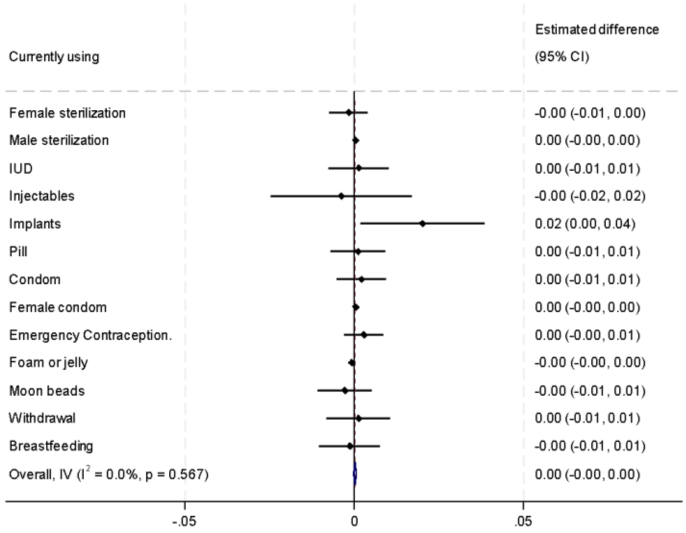

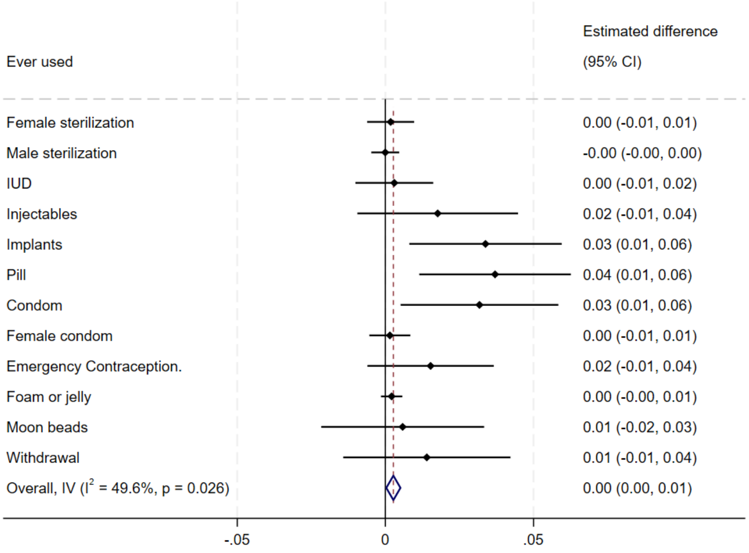

Figure notes: each point/square on the graphic represents the estimated coefficient obtained from regressing a binary indicator of having ever used the method in question on treatment - lines illustrate 95% confidence intervals.

Formatted: Line spacing: single

Table 6 shows estimated impact on prenatal care. A total of 398 current pregnancies, as well

as 653 pregnancies completed between baseline and endline were ~~analysed~~analyzed. No differences were found in antenatal care attendance or deworming. Treatment increased the likelihood of taking iron in current pregnancies by 8% pts and the likelihood of taking supplements in recently completed pregnancies by 4% pts [0.3,7.7].

Table 6: Impact on Prenatal Care

Outcome	Attended antenatal care	Currently taking iron	Deworming	Antenatal care for recent pregnancy	Recent pregnancy >=4 visits	Recent pregnancy: iron	Recent pregnancy: deworming
Treatment effect	0.0593	0.0845*	0.0167	0.00955	0.0229	0.0361*	0.0374
(unadjusted)	(0.0449)	(0.0431)	(0.0459)	(0.0161)	(0.0414)	(0.0189)	(0.0314)
Adjusted treatment effect	0.0453	0.0758*	0.00757	0.0156	0.0209	0.040**	0.0369
	(0.0468)	(0.0456)	(0.0483)	(0.0158)	(0.0412)	(0.0189)	(0.0310)
Control group average	0.621	0.605	0.451	0.958	0.454	0.901	0.808
Sample	Women currently pregnant			Women completing pregnancy last year			
Survey modality	In person						
Observations	398	398	398	653	619	653	647

Notes: Coefficients displayed are mean differences with standard errors in parentheses. Adjusted models control for age group (age < 20, age 20-34 (ref), age >=35), educational attainment, district, baseline family planning knowledge, baseline hemoglobin level and baseline body mass index. Standard errors were clustered at the enumeration area level. *p-value<0.10, **p-value<0.05, ***p-value<0.01

Table 7 shows estimated impact on other ~~behaviour~~behavior. The intervention increased women's efforts to eat healthy by 4% pts; interestingly, these efforts were generally not linked to losing weight; on average, treated women were 2.5% less likely to attempt for weight loss, and 3% pts more likely to try to gain weight. In terms of food intake, the treatment appears to have increased the frequency of fruit and vegetable consumption, consistent with the results on healthier diets. This aligns with consistent messaging on increasing healthy habits such as

Formatted: Font: 10 pt, Font color: Auto
Formatted: Left
Formatted: Font: 10 pt, Font color: Auto
Formatted: Left
Formatted Table
Formatted: Font: 10 pt
Formatted: Font: 10 pt, Font color: Auto
Formatted: Left
Formatted: Font: 10 pt, Font color: Auto
Formatted: Left
Formatted: Font: 10 pt, Font color: Auto
Formatted: Left
Formatted: Font: 10 pt
Formatted: Left
Formatted: Font: 10 pt, Font color: Auto
Formatted: Font: 10 pt
Formatted: Font: 10 pt, Font color: Auto
Formatted: Left
Formatted: Font: 10 pt
Formatted: Font: 10 pt, Font color: Auto
Formatted: Font: 10 pt, Font color: Auto
Formatted: Left
Formatted: Font: 10 pt, Font color: Auto
Formatted: Left
Formatted: Font: 10 pt
Formatted: Left
Formatted: Font: 10 pt, Font color: Auto
Formatted: Left
Formatted: Font: 10 pt, Font color: Auto
Formatted: Left
Formatted: Font: 10 pt
Formatted: Font: 10 pt, Font color: Auto
Formatted: Left
Formatted: Font: 10 pt, Font color: Auto
Formatted: Left
Formatted: Font: 10 pt, Font color: Auto
Formatted: Left
Formatted: Font: 10 pt
Formatted: Font: 10 pt
Formatted: Line spacing: single

regular activity, the inclusion of nutritious and iron rich foods, and maintaining a healthy body weight.

Table 7: Impact on Other Health ~~Behaviours~~Behaviors

Outcome	Aims for health diet	Tried to lose weight	Tried to gain weight	Times at fruit	Times ate vegetables	Times ate protein	Currently taking iron supplements	Exercised
Treated	0.0407***	-0.025**	0.0355***	0.0686**	0.0484**	-0.00060	0.0156	0.0154
(Unadjusted)	(0.0144)	(0.0108)	(0.0128)	(0.0346)	(0.0245)	(0.0183)	(0.00988)	(0.0124)
Adjusted treatment effect	0.0312**	-0.025**	0.0316**	0.0583*	0.0371*	0.00202	0.0174*	0.0119
	(0.0123)	(0.0106)	(0.0126)	(0.0298)	(0.0213)	(0.0172)	(0.00965)	(0.0118)
Control group average	0.552	0.255	0.242	1.335	1.332	1.760	0.136	0.335
Survey modality	Phone survey							
Observations	5,233	5,233	5,233	5,233	5,232	5,233	5,233	5,233

Notes: Coefficients displayed are mean differences with standard errors in parentheses. Adjusted models control for age group (age < 20, age 20-34 (ref), age >=35), educational attainment, district, baseline family planning knowledge, baseline hemoglobin level and baseline body mass index. Standard errors were clustered at the enumeration area level. *p-value<0.10, **p-value<0.05, ***p-value<0.01.

Table 8 shows estimated impacts on pregnancy and birth outcomes. 41% of currently pregnant women in the control group reported that the current pregnancy was not planned; this proportion was 5% pts smaller in the treatment group, but not significantly different. Unplanned pregnancies in the past were slightly more common among treated women (but not statistically different). The proportion of previous pregnancies lost as well as the proportion of most recent births born with low birthweight (< 2500 grams) were substantially lower in the

treated group – these differences were however not significant statistically due to the small number of observations here.

Table 8: Impact on pregnancy and birth outcomes

Outcome	Pregnancy was unplanned	Birth spacing < 2 years	Previous pregnancy unplanned	Previous pregnancy low birthweight	Previous pregnancy lost
Treatment effect	-0.0605	0.0519	0.0260	-0.0143	-0.0301
(unadjusted)	(0.0481)	(0.0546)	(0.0341)	(0.0246)	(0.0216)
Adjusted treatment effect	-0.0531	0.0496	0.0215	-0.0157	-0.0305
	(0.0495)	(0.0516)	(0.0337)	(0.0245)	(0.0209)
Control group average	0.410	0.372	0.261	0.0888	0.0384
Survey modality	In person				
Sample	Currently pregnant		Completed pregnancy recently		
Observations	398	371	653	565	653

Notes: Coefficients displayed are mean differences with standard errors in parentheses. Adjusted models control for age group (age < 20, age 20-34 (ref), age >=35), educational attainment, district, baseline family planning knowledge, baseline hemoglobin level and baseline body mass index. Standard errors were clustered at the enumeration area level. *p-value<0.10, **p-value<0.05, ***p-value<0.01

Table 9 shows estimated impact on biomarkers. While we see some lower rates of anemia among treated women who currently are, or recently were pregnant (Table 9, column 2), these differences are not statistically significant. No changes were found for BMI overall, or any of the categories ~~analysed~~analyzed (underweight, overweight, obese).

Table 9: Impact on biomarkers

Formatted: Font color: Auto
Formatted: Font color: Auto
Formatted: Left
Formatted Table
Formatted: Font color: Auto
Formatted: Left
Formatted: Font color: Auto
Formatted: Left
Formatted: Font color: Auto
Formatted: Left
Formatted: Font color: Auto
Formatted: Left
Formatted: Left
Formatted: Font color: Auto
Formatted: Left
Formatted: Font color: Auto
Formatted: Left
Formatted: Font color: Auto
Formatted: Left
Formatted: Font color: Auto
Formatted: Left
Formatted: Font color: Auto
Formatted: Left
Formatted: Font color: Auto
Formatted: Left
Formatted: Font: 10 pt
Formatted: Line spacing: single
Formatted: Font: Not Bold

Outcome	Anemic (Hg<12 g/dL)	Anemic (Hg<12 g/dL)	Body mass index	Overweight (BMI >=25, <30)	Obese (BMI >=30)	Underweight (BMI <18.5)
Treatment effect	0.00745	-0.0495	0.183	0.00277	0.0150	0.0120
(unadjusted)	(0.0129)	(0.0315)	(0.165)	(0.0126)	(0.0106)	(0.00826)
Adjusted treatment effect	0.0111	-0.0468	0.0922	0.000613	0.0102	0.0140*
	(0.0127)	(0.0311)	(0.111)	(0.0121)	(0.00831)	(0.00808)
Control group average	0.23	0.23	24.89	0.26	0.15	0.08
Sample	All	Current or recent pregnancy	All	All	All	All
Observations	4,664	992	4,852	4,852	4,852	4,852

Notes: Coefficients displayed are mean differences with standard errors in parentheses. Adjusted models control for age group (age < 20, age 20-34 (ref), age >=35), educational attainment, district, baseline family planning knowledge, baseline hemoglobin level and baseline body mass index. Standard errors were clustered at the enumeration area level. *p-value<0.10, **p-value<0.05, ***p-value<0.01.

Table 10 shows the result of the per protocol analysis, where we compare control women who had never called the platform to treated women who had called the platform at least five times.. As expected, estimated impacts on knowledge scores are higher among compliers; however, these estimates become substantially smaller again when we adjust for women's differences in education and reproductive health knowledge at baseline.

Table 10: Per protocol analysis

Outcome	SRH Knowledge z-score	Currently uses modern method	Uses iron supplements	Used iron supplements	Lost pregnancy	Anemic	Anem post pregn

Treatment effect	0.127***	0.0409**	0.0571	0.000959	-0.00654	0.00586	-0.04[illegible]
(Unadjusted)	(0.0332)	(0.0159)	(0.0515)	(0.0102)	(0.00671)	(0.0147)	(0.03[illegible]
Adjusted treatment	0.0650**	0.0206	0.0575	0.0117	-0.00911	0.0168	0.016[illegible]
effect	(0.0308)	(0.0155)	(0.0523)	(0.00954)	(0.00668)	(0.0144)	(0.014[illegible]
Control group average	-0.0613	0.374	0.643	0.915	0.0386	0.229	0.389
Sample	All	All	Pregnant now	Pregnant last year		All	Pregn[illegible]
Restriction	Compliers only						
Observations	3,561	3,561	292	3,313	3,313	3,400	727

*Notes:*Only women in the control group who never called the service, and women in the treatment group who called the service at least five times are included in this table. Coefficients displayed are mean differences with standard errors in parentheses. Adjusted models control for age group (age < 20, age 20-34 (ref), age >=35), educational attainment, district, baseline family planning knowledge, baseline hemoglobin level and baseline body mass index. Standard errors were clustered at the enumeration area level. *p-value<0.10, **p-value<0.05, ***p-value<0.01. SRH=sexual and reproductive health.

Stratified Results

Table 11 shows stratified impact results by age and educational attainment group, as well as by district. For age, we find the weakest effects among women under age 20, and largest impacts among women 35 years and older. Educational level seemed to have little impact, with only slightly larger effects for women with secondary or higher education compared to women with less education. At the district level, impacts vary substantially, with the largest treatment effects in Madi-Okollo (the 0.12 SD increase here is almost twice as large as the effect we see in the sample overall), and the smallest impacts in Rwampara (0.02 SD).

Table 11: Stratified results

Stratum	Age <20	Age 20 - 34	Age 35+	Secondary or higher education	Primary education	Kampala	Katakwi	Madi-Okollo	Rwampara

Formatted: Font color: Auto
Formatted: Left
Formatted: Font color: Auto
Formatted: Left
Formatted: Font color: Auto
Formatted: Font: 10 pt
Formatted: Left
Formatted: Left
Formatted: Font color: Auto
Formatted: Left
Formatted: Font color: Auto
Formatted: Left
Formatted: Font color: Auto
Formatted: Left
Formatted: Font: 10 pt
Formatted: Left
Formatted: Font color: Auto
Formatted: Font: 10 pt
Formatted: Left
Formatted: Font color: Auto
Formatted: Left
Formatted: Left
Formatted: Font color: Auto
Formatted Table
Formatted: Left
Formatted: Font color: Auto
Formatted: Left
Formatted: Font color: Auto
Formatted: Left
Formatted: Font: 10 pt, Font color: Auto
Formatted: Font: 10 pt
Formatted: Font: 12 pt
Formatted: Font: 16 pt
Formatted: Heading 2
Formatted: Font: Not Bold
Formatted: Line spacing: single
Formatted: Font: 10 pt
Formatted: Left

Treatment effect	0.0213	0.0674*	0.0901*	0.0723**	0.0587*	0.0949**	0.0828*	0.122*	0.0221
	(0.127)	(0.0344)	(0.0500)	(0.0430)	(0.0331)	(0.0431)	(0.0455)	(0.0654)	(0.0517)
Observations	291	2,978	1,608	1,804	3,073	1,472	1,093	650	1,662

Notes: Coefficients displayed are mean differences with standard errors in parentheses. Adjusted models control for age group (age < 20, age 20-34 (ref), age >=35), educational attainment, district, baseline family planning knowledge, baseline hemoglobin level and baseline body mass index. Standard errors were clustered at the enumeration area level. *p-value<0.10, **p-value<0.05, ***p-value<0.01

Harms statement

No harm was reported during the trial.

Discussion

The main objective of this study was to assess the impact of Viamo's call-in platform on health knowledge, ~~behaviours~~behaviors and outcomes. Conceptually, knowledge platforms could result in changes on three distinct levels: changes in health knowledge, changes in health ~~behaviour~~behavior, and changes in health outcomes. The results presented here suggest that changes in knowledge definitely occurred, with treated women displaying improved sex and reproductive health knowledge, being better informed about anemia treatment and more familiar with a larger range of contraceptive methods. This increased familiarity with contraceptive knowledge was also partially visible in contraceptive use. Treated women were on average only marginally (and insignificantly) more likely to use modern contraception, but used a different mix of contraceptives, with higher usage of implants, and somewhat reduced reliance on injectables and calendar methods. In terms of future plans, the intervention seems to have substantially increased the proportion of women who say they will use modern contraception in the future.

For anemia - one of the key health challenges addressed on the platform - the treatment seems to have resulted in an increased uptake of supplements during pregnancy; these higher rates of supplementation also seem consistent with marginally lower rates of anemia among recently pregnant women. The estimated differences in anemia in this subgroup are however not statistically significant, which may partially be due to the relatively small number of women. Despite the high rates of anemia among non-pregnant women, we did not see any change in

Formatted: Font: 10 pt
Formatted Table
Formatted: Font: 10 pt
Formatted: Font: 10 pt
Formatted: Left
Formatted: Font: 10 pt
Formatted: Font: Not Bold
Formatted: Font: 16 pt
Formatted: Heading 2
Formatted: Font: Not Bold
Formatted: Font: 18 pt
Formatted: Heading 1, Line spacing: single

general protein intake or consumption of iron supplements. Given this lacking change (and the small proportion of women benefiting from additional supplementation during pregnancy), the insignificant impact on general hemoglobin levels makes sense.

We also see some interesting declines in the rates of pregnancy loss and the prevalence of low-birth-weight births in this subsample, but did not have sufficient statistical power here due to the relatively small number of women completing a pregnancy within the study period.

Formatted: Normal, Line spacing: single

Given that a larger proportion of women in the treatment group claimed to aim for a healthy diet and to eat more fruit and vegetables, nutrition related content clearly reached its target. Interestingly, the messages received around "healthy body shapes" ~~don't~~do not seem to have triggered a major shift towards eating less and lower BMIs, but seems to have induced a desire to change in both directions, i.e. we see an increasing proportion of women who want to lose weight and an increasing proportion of women who want to gain weight (and hence a smaller proportion of women who want to stay as they are). These shifts in intentions and behaviors are not seen in the anthropometric data - the intervention neither changed average BMI nor the proportions of women classified as underweight, overweight or obese.

Formatted: Line spacing: single

In terms of the primary outcome, our results seem consistent with a recent review of mHealth intervention, which suggests that digital information sources can improve sexual and reproductive health knowledge as well as use of sexual and reproductive health services [11]. Compared to the estimates reported for an interactive intervention in Ghana [7, 12] the effect sizes observed here seem smaller, which seems plausible given that not all women engaged with the service on a regular service. Compared to previous studies conducted in other parts of Africa we also observed rather high sexual and reproductive health knowledge at baseline – most women were for example familiar with a range of contraceptive methods (beyond traditional methods, condoms and pills), and myths regarding side effects of contraception were less common that seen in other settings [7, 12] – this likely also contributes to the somewhat smaller knowledge impacts seen here.

In terms of policy, one of the main advantages of the platform used is its relatively low cost – particularly when run at large scale; most of the costs of content development are related to the

initial set-up, while operating costs are relatively minor. More research is needed here to investigate the cost-effectiveness of these new mobile technology channels as tools to improve health knowledge among vulnerable populations relative to traditional channels such as radio, tv or community outreach activities.

Despite the very large, stratified and regionally representative sample, the study has some limitations. First, there were not as many pregnant women as anticipated, reducing power on pregnancy-related ~~behaviours~~behaviors and outcomes to some extent. Second, we found that the proportion of women using Airtel phones was substantially lower in some rural districts, making it very difficult to reach enrollment targets in some communities. Even though a more selected sample clearly does not undermine the internal validity of the study (since randomization was done at the individual level), it is not clear whether the final set of women enrolled in the study is fully representative of the larger populations in the four study districts as well as Uganda overall - since the service is currently only available to Airtel users, this study simply focused on women with access to this service~~.~~. Third, many of the ~~behaviours~~behaviors measured were self-reported; while we implemented verification measures by taking pictures of antenatal and child health cards and asking women for evidence of contraceptive packaging, such direct evidence was generally only available for a small minority of women, and could thus not be used in the analysis. It is clearly possible that access to the service changes norms and reporting bias, which could explain some of the ~~behavioural~~behavioral changes reported and detected in our analysis. The primary outcome of the study - the reproductive knowledge score - should not be subject to any reporting bias as all women should have the incentive to give the correct answer on the true/false knowledge quiz. Fourth, the individual-level randomization made the study vulnerable to spillover effects. Qualitative feedback collected at the end of the study suggests that treated women both invited other community members – spouses, friends and other family members – to use the service and actively shared new information with them. Given that some of this shared information likely reached the control group, it seems likely that the impact reported here underestimate the true causal effect of the program. We controlled for direct service use by excluding active users in the per protocol analysis (Table 10) – this does however not capture direct sharing of knowledge among community members.

Formatted: Line spacing: single

Conclusions

Overall, the results presented here suggest that the platform can effectively improve health knowledge and to some extent also health ~~behaviours~~behaviors among vulnerable populations. These changes in knowledge, awareness and intentions could plausibly also induce changes in health outcomes in the long run; longer-term studies will likely be needed to accurately measure such effects. Future research should also aim to quantify knowledge spillovers within communities as well as the cost-effectiveness of call-in platform like the one ~~analysed~~analyzed here relative to other communication channels such as TV, radio, posters or community-based activities.

Formatted: Font: 18 pt

Formatted: Font: Not Bold

Formatted: Line spacing: single, Border: Top: (No border), Bottom: (No border), Left: (No border), Right: (No border), Between : (No border)

Acknowledgements: We would like to thank all involved staff members at IPA Uganda as well as in the Viamo team for their efforts and continued support of the project. We are also grateful to the more than 6000 study participants for their willingness to participate in the study. Lastly, we would like to thank the USAID Development Innovation Ventures (DIV) team for their input and feedback throughout the project.

Funding: this project was funded through USAID DIV grant number 7200AA21FA00018.

Role of funder: The funder played no role in the analysis or interpretation of the data.

Formatted: Line spacing: single, Border: Top: (No border), Bottom: (No border), Left: (No border), Right: (No border), Between : (No border)

Formatted: Line spacing: single, Border: Top: (No border), Bottom: (No border), Left: (No border), Right: (No border), Between : (No border)

References

1. World Bank, *Individuals using the Internet (% of population) | Data*. 2025.
2. D'Souza, P., et al., *Factors influencing contraception choice and use globally: a synthesis of systematic reviews.* The European Journal of Contraception & Reproductive Health Care: The Official Journal of the European Society of Contraception, 2022. **27**(5): p. 364-372.
3. Noar, S.M., *A 10-year retrospective of research in health mass media campaigns: where do we go from here?* Journal of Health Communication, 2006. **11**(1): p. 21-42.
4. Wakefield, M.A., B. Loken, and R.C. Hornik, *Use of mass media campaigns to change health behaviour.* Lancet, 2010. **376**(9748): p. 1261-1271.
5. Stead, M., et al., *Mass media to communicate public health messages in six health topic areas: a systematic review and other reviews of the evidence*. Public Health Research. 2019, Southampton (UK): NIHR Journals Library.
6. Uganda Communications Commission, *Market performance report 2022/2023*. 2023, Uganda Communications Commission.
7. Rokicki, S., et al., *Impact of a Text-Messaging Program on Adolescent Reproductive Health: A Cluster–Randomized Trial in Ghana.* American Journal of Public Health, 2017. **107**(2): p. 298-305.
8. Unger, J.A., et al., *Short message service communication improves exclusive breastfeeding and early postpartum contraception in a low- to middle-income country setting: a randomised trial.* BJOG: an international journal of obstetrics and gynaecology, 2018. **125**(12): p. 1620-1629.
9. Harrington, E.K., et al., *An mHealth SMS intervention on Postpartum Contraceptive Use Among Women and Couples in Kenya: A Randomized Controlled Trial.* American Journal of Public Health, 2019. **109**(6): p. 934-941.
10. UNDP. *Digital X Solution Catalogue*. 2025 10 Sep 2025].
11. Onukwugha, F.I., et al., *The effectiveness and characteristics of mHealth interventions to increase adolescent's use of Sexual and Reproductive Health services in Sub-Saharan Africa: A systematic review.* PLOS ONE, 2022. **17**(1): p. e0261973.
12. Rokicki, S. and G. Fink, *Assessing the reach and effectiveness of mHealth: evidence from a reproductive health program for adolescent girls in Ghana.* BMC Public Health, 2017. **17**(1): p. 969.

Formatted: Font: 16 pt

Formatted: Space Before: 0 pt, After: 0 pt

Formatted: English (United States)

Formatted: Font: 12 pt, Not Bold

Journal Requirements:

When submitting your revision, we need you to address these additional requirements.

1. Please ensure that your manuscript meets PLOS ONE's style requirements, including those for file naming. The PLOS ONE style templates can be found at

https://journals.plos.org/plosone/s/file?id=wjVg/PLOSOne_formatting_sample_main_body.pdf

and

https://journals.plos.org/plosone/s/file?id=ba62/PLOSOne_formatting_sample_title_authors_affiliations.pdf

We have reviewed the manuscript and reformatted the text following the PLOS one style templates.

2. Please describe in your methods section how capacity to provide consent was determined for the participants in this study. Please also state whether your ethics committee or IRB approved this consent procedure. If you did not assess capacity to consent please briefly outline why this was not necessary in this case.

We read out the consent form to illiterate women and asked them to provide a thumb print to express their explicit consent instead of signing. This process was suggested and approved by local IRB. Women not able to understand the scope of the study as well as women not able or willing to consent using either modality were excluded from the study.

3. Please include a complete copy of PLOS' questionnaire on inclusivity in global research in your revised manuscript. Our policy for research in this area aims to improve transparency in the reporting of research performed outside of researchers' own country or community. The policy applies to researchers who have travelled to a different country to conduct research, research with Indigenous populations or their lands, and research on cultural artefacts. The questionnaire can also be requested at the journal's discretion for any other submissions, even if these conditions are not met. Please find more information on the policy and a link to download a blank copy of the questionnaire here: https://journals.plos.org/plosone/s/best-practices-in-research-reporting. Please upload a completed version of your questionnaire as Supporting Information when you resubmit your manuscript.

We have completed the inclusivity questionnaire and have uploaded it with the revised documents.

4. Thank you for stating the following financial disclosure:
"This project was funded through USAID DIV grant number 7200AA21FA00018"
Please state what role the funders took in the study. If the funders had no role, please state: "The funders had no role in study design, data collection and analysis, decision to publish, or preparation of the manuscript."
If this statement is not correct you must amend it as needed.

Please include this amended Role of Funder statement in your cover letter; we will change the online submission form on your behalf.

This is correct - we have included this statement in the cover letter as suggested.

5. Thank you for stating the following in the Competing Interests section:

"The first, second and third author declare no competing interest. The last author (IK) is currently employed by Viamo, the company operating the service analyzed. She was not involved in the data analysis and did not modify the way the results are presented or described in any way."

Please confirm that this does not alter your adherence to all PLOS ONE policies on sharing data and materials, by including the following statement: "This does not alter our adherence to PLOS ONE policies on sharing data and materials." (as detailed online in our guide for authors http://journals.plos.org/plosone/s/competing-interests). If there are restrictions on sharing of data and/or materials, please state these. Please note that we cannot proceed with consideration of your article until this information has been declared.

Please include your updated Competing Interests statement in your cover letter; we will change the online submission form on your behalf.

We have included an updated conflict of interest statement in the cover letter.

6. When completing the data availability statement of the submission form, you indicated that you will make your data available on acceptance. We strongly recommend all authors decide on a data sharing plan before acceptance, as the process can be lengthy and hold up publication timelines. Please note that, though access restrictions are acceptable now, your entire data will need to be made freely accessible if your manuscript is accepted for publication. This policy applies to all data except where public deposition would breach compliance with the protocol approved by your research ethics board. If you are unable to adhere to our open data policy, please kindly revise your statement to explain your reasoning and we will seek the editor's input on an exemption. Please be assured that, once you have provided your new statement, the assessment of your exemption will not hold up the peer review process.

We have uploaded a complete replication package comprising both the data files and the Stata DO files creating figures and tables as supplementary materials.

7. We note that Figure 1 in your submission contain [map/satellite] images which may be copyrighted. All PLOS content is published under the Creative Commons Attribution License (CC BY 4.0), which means that the manuscript, images, and Supporting Information files will be freely available online, and any third party is permitted to access, download, copy, distribute, and use these materials in any way, even commercially, with proper attribution. For these reasons, we cannot publish previously copyrighted maps or satellite images created using proprietary data, such as Google software (Google Maps,

Street View, and Earth). For more information, see our copyright guidelines: http://journals.plos.org/plosone/s/licenses-and-copyright.

We require you to either (1) present written permission from the copyright holder to publish these figures specifically under the CC BY 4.0 license, or (2) remove the figures from your submission:

This shapefile comes from GADM, which explicitly allows such use: "*Using the data to create maps for publishing of academic research articles is allowed. Thus you can use the maps you made with GADM data for figures in articles published by PLoS, Springer Nature, Elsevier, MDPI, etc. You are allowed (but not required) to publish these articles (and the maps they contain) under an open license such as CC-BY as is the case with PLoS journals and may be the case with other open access articles.*" (https://gadm.org/license.html),

We have added the source to the legend of Figure 1.

8. Please include captions for your Supporting Information files at the end of your manuscript, and update any in-text citations to match accordingly. Please see our Supporting Information guidelines for more information: http://journals.plos.org/plosone/s/supporting-information.

We have relabeled the supplemental material figures following these guidelines.

9. If the reviewer comments include a recommendation to cite specific previously published works, please review and evaluate these publications to determine whether they are relevant and should be cited. There is no requirement to cite these works unless the editor has indicated otherwise.

NA – we did not received any such requests.

Reviewers' comments:

Reviewer #1:

Thank you for sharing this very interesting study for review. Below are some points that might help the authors improve their manuscript.

Thank you for the careful review and very helpful suggestions – we have done our best to address all of them as outlined in further detail below.

Introduction:

- It would be beneficial to mention a clear hypothesis or research question at the end of the introduction. This will guide readers in understanding how the results align with the study's goals.

Thank you for this suggestion. We have added the following sentence to the Introduction:

"Our primary hypothesis was that access to the platform can improve sexual and reproductive health knowledge and attitudes."

Methods:

- The methodology is well-designed and aligns with the CONSORT checklist. However, further details are needed regarding the data collection tools. Specifically:

- Which tools were used to assess the primary and secondary variables?

Apologies for the lack of detail here in the original manuscript. We provide further details on the outcomes variables on page 8 and 9 now, where we write:

"The two primary outcomes of the study were reproductive health knowledge and nutritional supplementation in pregnancy. For reproductive health, we used a 15-item questionnaire of true/false statements (items listed in Supplemental Materials S2). This tool was based on previous studies conducted in sub-Saharan Africa [7] and adapted to match local context. To facilitate interpretation of regression results, we converted the raw scores (number of correct answers out of 15) into z-scores with mean zero and standard deviation one. For antenatal care, we used self-reported iron supplementation as an outcome measure. Secondary outcomes included contraceptive use, prenatal care uptake, hemoglobin level (as a biomarker indicating actual iron intake), body mass index, and birth spacing.

For pregnancy related outcomes, we analyzed two separate sets of women: first, women reporting to be pregnant at the time of the endline survey (currently pregnant), and second, women who completed a pregnancy in the year preceding the endline survey (recently pregnant). For current pregnancies, we analyzed self-report iron supplementation as well as self-reported use of antenatal care services, and whether women indicate the pregnancy was planned. For recently completed pregnancies, we analysed the same variables, but also analysed pregnancy loss and low birth weight (reported weight at birth < 2.5kgs) as additional secondary outcomes.

- Validity and reliability of the measurement tools should be addressed to ensure the robustness of the results.

We added some more details in the Methods section, where we write:

"*This tool was based on previous studies conducted in sub-Saharan Africa [7] and adapted to match local context.*"

For the secondary outcomes, self-reporting biases are definitely a concern which we discuss in the Limitations section of the paper, where we write:

"Third, many of the behaviors measured were self-reported; while we implemented verification measures by taking pictures of antenatal and child health cards and asking women for evidence of contraceptive packaging, such direct evidence was generally only available for a small minority of women, and could thus not be used in the analysis. It is clearly possible that access to the service changes norms and reporting bias, which could explain some of the behavioral changes reported and detected in our analysis. The primary outcome of the study - the reproductive knowledge score - should not be subject to any reporting bias as all women should have the incentive to give the correct answer on the true/false knowledge quiz."

- The term "normalized" is used to describe the data collection tool. This terminology is not common in this context. Consider replacing it with terms like "valid and reliable."

Our apologies – we just meant to say that we created a z-score out of the raw number of correct answers prior to analysis. We have rephrased this as follows in the revised Methods section:

"To facilitate interpretation of regression results, we converted the raw scores (number of correct answers out of 15) into z-scores with mean zero and standard deviation one."

- The terms "reproductive health knowledge" and "antenatal care" seem to overlap or create confusion. Please clarify the distinction between these terms and ensure consistency throughout the manuscript.

Apologies for the confusion: we meant to refer to nutritional supplementation during antenatal care here, and have made this more explicit in the revised version of the manuscript where we write:

"For nutritional supplementation during pregnancy, we used self-reported iron supplementation as an outcome measure"

- Details regarding exclusion criteria are missing and should be provided.

Apologies for the lack of detail here – we have added a new section on inclusion and exclusion criteria, which reads as follows:

"..Study eligibility was assessed by trained study enumerators. Women living in selected enumeration areas were eligible for inclusion in the study if they were aged 18-49 years and

had access to an Airtel (a mobile network operator) phone number within their household. Given the explicit focus on antenatal care, family planning and post-partum, all women who were pregnant during the listing were automatically invited to join the study; non-pregnant women were randomly selected for the study until the 45 women per cluster target was reached. In clusters with less than 45 women, all women were invited to participate in the study. Women were ineligible if they had used the Viamo platform previously, they planned to move outside of the study area within 12 months of baseline or if they did not speak one of the six languages available on the platform: Ateso, English, Luganda, Lugbara, Luo or Runyakitara."

- Clarify who assessed eligibility and whether they were blinded during the randomization process.

We have added the following information***:***

"Study eligibility was assessed by trained study enumerators."

- The manuscript contains 11 tables, which may be excessive. Consider merging some tables or limiting the number to those most essential for clarity. Ensure all tables mentioned in the manuscript are explained in the results section.

We agree that there is lots of information in this manuscript, but really believe that all of these results are important. Given that there is no formal limit at PLOS One, we would thus prefer to leave all figures and tables in, and have verified all of them are mentioned in the text. If you feel strongly that some figures should go to the Supplemental Materials we would of course be happy to accommodate that**.**

- There is no mention of the interventional materials used with the experimental group. This information is necessary for understanding the study design and replication in future research.

We fully agree with this. We provide a detailed description of the intervention on page 6. The intervention is easy and complex at the same time: it is easy, because essentially women are just invited to call a toll-free number and then get to access content designed for them. The complexity comes from the fact that women can freely navigate content. We show the most commonly accessed items in Table 3, and provide a more detailed example in supplemental materials S1.

- The discussion section lacks comparisons with previous studies. Including these would contextualize the findings and add depth.

Thank you for this suggestion. We have added the following paragraph to the Disucssion:

"*In terms of the primary outcome, our results seem consistent with a recent review of mHealth intervention, which suggests that digital information sources can improve sexual and reproductive health knowledge as well as use of sexual and reproductive health services [10]. Compared to the estimates reported for an interactive intervention in Ghana [7, 11] the*

effect sizes observed here seem smaller, which seems plausible given that not all women engaged with the service on a regular service. "

- Discuss the study's limitations and strengths to aid future researchers.

We discuss strengths and limitatios at the very end of the Discussion section, where we write:

"Despite the very large, stratified and regionally representative sample, the study has some limitations. First, there were not as many pregnant women as anticipated, reducing power on pregnancy-related behaviors and outcomes to some extent. Second, we found that the proportion of women using Airtel phones was substantially lower in some rural districts, making it very difficult to reach enrollment targets in some communities. Even though a more selected sample clearly does not undermine the internal validity of the study (since randomization was done at the individual level), it is not clear whether the final set of women enrolled in the study is fully representative of the larger populations in the four study districts as well as Uganda overall - since the service is currently only available to Airtel users, this study simply focused on women with access to this service. Third, many of the behaviors measured were self-reported; while we implemented verification measures by taking pictures of antenatal and child health cards and asking women for evidence of contraceptive packaging, such direct evidence was generally only available for a small minority of women, and could thus not be used in the analysis. It is clearly possible that access to the service changes norms and reporting bias, which could explain some of the behavioral changes reported and detected in our analysis. The primary outcome of the study - the reproductive knowledge score - should not be subject to any reporting bias as all women should have the incentive to give the correct answer on the true/false knowledge quiz. Fourth, the individual-level randomization made the study vulnerable to spillover effects. Qualitative feedback collected at the end of the study suggests that treated women both invited other community members – spouses, friends and other family members – to use the service and actively shared new information with them. Given that some of this shared information likely reached the control group, it seems likely that the impact reported here underestimate the true causal effect of the program. We controlled for direct service use by excluding active users in the per protocol analysis (Table 10) – this does however not capture direct sharing of knowledge among community members."

- The trial flowchart should be reshaped to align with the original CONSORT 2010 format. available at CONSORT 2010

We tried to follow the original template as closely as possible. The main challenge with our study is that we had two follow-up modalities, which required as to make the format a bit wider than usual. We are not sure how to simplify this but are open to suggestions.

Reviewer #2:

I commend the authors' work to assess the impact of Viamo's call-in platform on sexual and reproductive health knowledge, behaviors and outcomes.

Please find my comments below (pg numbers used in the absence of line numbers):

Thank you for the nice feedback and very helpful suggestions – we really appreciate it!

-pg 1/27: Title: Please consider revising the title to reflect the aim of this manuscript and the analysis/discussion/conclusion. For instance, "Estimating the Impact of Viamo Call-in Information Services on Sexual and Reproductive Health Knowledge, Behavior, and Outcomes among women of reproductive age: Results from a 2-Arm Open Label Randomized Controlled Trial in Uganda" may be a more appropriate title

Thank you for this suggestion – we have adopted this new title.

-pg 1/27: Please maintain a consistent spelling for "behavior". In some aspects of the manuscript, this is spelt at "behaviour"

Thanks for catching this and our apologies for the inconsistent US/UK spelling – we now use behavior throughout.

-pg 2/27: Please cite this source of this sentence, "The platform is free of charge, and currently has over 4 million users in Uganda, and 42 million users in LMICs globally."

This was an internal number provided by Viamo; we have replaced this now with an official UNDP estimate (which is slightly lower):

"The platform is free of charge, and currently has over 4 million users in Uganda, and 35 million users in LMICs globally [10].»

-pg 9/27: "Follow-up rates (completing either the phone or the in person survey or both) were 92% in both arms." Please review this sentence and consider removing it since the estimates do not correspond with Figure 2 and the other sentences in this section of the manuscript better interpret the numbers.

Apologies for the lack of clarity here. We used two parallel modalities (with overlapping questions) to reach households at endline. Some of the households completed both and some just one survey – 92% completed either one or both surveys. We have tried to be more clear about this in the revised Results section where we write:

"At endline, 87% (n=2,678) of control arm participants, and 86% (n=2,555) of intervention arm participants completed the phone survey. Slightly fewer participants completed the in-person survey (81% of both arms). 92% of participants in both arms completed at least one of the two modalities (i.e. either a phone or an in-person survey)."

-pg 11/27: Table 2: Please review the results with *** (p-value <0.01). All the P-values are >0.01 and ** (p-value<0.05) would be more appropriate. In addition, some p-values are not significant (0.471 and 0.958) and should not have any asterisk on them.

Sorry for the confusion. The numbers in parentheses here are standard errors, and the p-values are all substantially below 0.01 in Table 2. We have made this clear in the revised Table 2 legend that now says:

"Notes: All point estimates displayed correspond to estimated mean differences in outcomes. Numbers in parentheses are robust standard errors.»

The asterisk notation (* for < 0.10, *** for < 0.01) is the default of Stata's outreg package – we would be happy to revise this if you feel strongly about this.

-pg 12/27: Table 3 footnote: How was C2L calculated? It doesn't seem to Times listened / Times reached based on the table. Please add the formula to the footnote.

This rate is simply the proportion of callers who listen to the message all the way to the end – this is automatically registered in the system. We have clarified this in the revised legend of the table, where we now write:

"C2L (caller-to-listener ratio) defines the % completion listening rate of a message, i.e. the proportion of callers who listen to the message all the way to the end."

-pg 13/27: Table 4: Please review the result with *** (p-value <0.01) because ** (p-value<0.05) would be more appropriate for p-value 0.0265 – row 2, column 2.

Once again, we apologize for the lack of clarity. The numbers in parentheses are not p-values but estimated standard errors. We have made this clear in the legend of the table now where we write:

"Coefficients displayed are mean differences with standard errors in parentheses."

-pg 15/27: Table 5: Please review the results with *** (p-value <0.01). All the P-values are >0.01 but <0.05 and ** (p-value<0.05) would be more appropriate.

-pg 16/27: Table 6: Please review the results with * (p-value <0.01). All of them are <0.05 and ** (p-value<0.05) would be more appropriate.

-pg 17/27: Table 7: Please review the results with *** (p-value <0.01) and * (p-value <0.10) and correct them as appropriate. All the p-values should have ** except p-value for 0.0174 (0.00965) which should have-pg 18/27: Table 8: Please include the corresponding asterix for the estimates with significant p-values in this table.

-pg 19/27: Table 9: Please include the corresponding asterix for the estimates with significant p-values in this table.

-pg 20/27: Table 10: Please include the corresponding asterix for the estimates with significant p-values in this table.

-pg 21/27: Table 11: Please review the results with p-values 0.0344*, 0.0331* and 0.0455* (where * is p-value <0.10) and correct them to ** (p-value <0.05) as appropriate. All should have ** except p-value: 0.00965 which should have ***

Once again, we apologize for the lack of clarity. The numbers in parentheses are not p-values but estimated standard errors. We have clearly indicated in the legends of all tables now.

-pgs 22-24: Discussion: Please incorporate existing studies that support or dispute your findings in the discussion section. This section needs a lot more work and is currently only a repetition/elaboration of the results' interpretation.

We have carefully revised the Discussion and added the following next text block to better place the study results into the existing literature:

"In terms of the primary outcome, our results seem consistent with a recent review of mHealth intervention, which suggests that digital information sources can improve sexual and reproductive health knowledge as well as use of sexual and reproductive health services [11]. Compared to the estimates reported for an interactive intervention in Ghana [7, 12] the effect sizes observed here seem smaller, which seems plausible given that not all women engaged with the service on a regular service. Compared to previous studies conducted in other parts of Africa we also observed rather high sexual and reproductive health knowledge at baseline – most women were for example familiar with a range of contraceptive methods (beyond traditional methods, condoms and pills), and myths regarding side effects of contraception were less common that seen in other settings [7, 12] – this likely also contributes to the somewhat smaller knowledge impacts seen here.

In terms of policy, one of the main advantages of the platform used is its relatively low cost – particularly when run at large scale; most of the costs of content development are related to the initial set-up, while operating costs are relatively minor. More research is needed here to investigate the cost-effectiveness of these new mobile technology channels as tools to improve health knowledge among vulnerable populations relative to traditional channels such as radio, tv or community outreach activities. "

Reviewer #3: I struggled to review this paper specifically the results section.

I could not figure out what metric was used to measure impact (such odd ratios).

The measurement of the outcome is not clear or easily understood.

We apologize for the lack of clarity in the original manuscript. We have added additional details on the outcome measurements in the Tables, and also have added further details in on the main outcome in the revised Methods section, where we write:

"For reproductive health, we used a 15-item questionnaire of true/false statements (items listed in Supplemental Materials S2). This tool was based on previous studies conducted in sub-Saharan Africa [7] and adapted to match local context. To facilitate interpretation of regression results, we converted the raw scores (number of correct answers out of 15) into z-scores with mean zero and standard deviation one. For nutritional supplementation during pregnancy, we used self-reported iron supplementation as an outcome measure. Secondary outcomes included contraceptive use, prenatal care uptake, hemoglobin level (as a biomarker indicating actual iron intake), body mass index, and birth spacing."

We hope this makes it easier to understand our analysis now.
