# Supplementary material for: Estimating the impact of Viamo call-in information services on sexual and reproductive health knowledge, behavior, and outcomes among women of reproductive age: A 2-arm open label randomized controlled trial in Uganda
Source: PLoS One. 2026 Feb 13;21(2):e0342327. doi: 10.1371/journal.pone.0342327 (PMC12904432; doi:10.1371/journal.pone.0342327)
Supplement: Attachment — Submitted filename: PONE-D-25-05733_R2-AF.pdf [file pone.0342327.s010.pdf]

PLOS One

Estimating the Impact of Viamo Call-in Information Services on Sexual and Reproductive Health Knowledge, Behavior, and Outcomes among Women of Reproductive Age: A 2-Arm Open Label Randomized Controlled Trial in Uganda

--Manuscript Draft--

**Manuscript Number:**	PONE-D-25-05733R2
**Article Type:**	Research Article
**Full Title:**	Estimating the Impact of Viamo Call-in Information Services on Sexual and Reproductive Health Knowledge, Behavior, and Outcomes among Women of Reproductive Age: A 2-Arm Open Label Randomized Controlled Trial in Uganda
**Short Title:**	Improving health knowledge through call-in information services
**Corresponding Author:**	Günther Fink<br>Swiss Tropical Institute: Schweizerisches Tropen- und Public Health-Institut<br>SWITZERLAND
**Keywords:**	health knowledge; call-in services; randomized trial; Uganda; sexual health; reproductive health; Nutrition; Viamo.
**Abstract:**	Background. Despite substantial increases in access to schooling and to the internet, health information and knowledge remains limited among women of reproductive health in many low and middle income countries. Given the near universal cell-phone coverage in low- and middle-income countries, call-in services offer an interesting new platform to provide high quality information to targeted populations. In this paper, we assessed the impact of the Viamo 3-2-1 call-in Platform on health knowledge and behavior among women of reproductive age in Uganda.<br><br>Methods. We conducted a 2-arm open label randomized controlled trial involving 6,000 randomly selected women, ages 18-49 years, with access to a simple feature Airtel cell phone in Uganda. Fifty enumeration areas were randomly selected in Kampala, Madi-Okollo, Rwampara and Katakwi districts. Consenting women were asked to complete a baseline and endline survey implemented by trained interviewers using tablets through home visits. After completion of baseline, women were randomized with equal probability to treatment and control using a tablet-generated random number draw. Study staff introduced treated women to the call-in service and encouraged its use through various incentives and promotional messages. Primary outcomes were sexual and reproductive health knowledge scores as well as iron supplementation in pregnancy. Secondary outcomes included self-reported use of contraception, healthy diets, birth spacing and birth outcomes as well as hemoglobin levels and BMI. Linear regression models were used to estimate mean differences in outcomes between treatment and control. Standard errors were corrected for the cluster-based sampling of women.<br><br>Results. A total of 6,011 women were enrolled into the study between December 6, 2022 and February 11, 2023; 3,052 were assigned to treatment, 2,959 used as controls. Endline data was collected between May 8, 2024 and August 4, 2024. Overall follow-up rates were 92% in both the treatment and the control group. Treatment increased average sexual and reproductive health knowledge by 0.07 standard deviations (95% CI [0.01, 0.11], p-value = 0.02) and increased the likelihood of taking iron supplements by 4% pts among recently pregnant women ([0.003, 0.08], p-value 0.04). Results for secondary outcomes were mixed, with positive changes for nutritional intake and family planning behaviors and intentions and no impact on hemoglobin levels and BMI. No adverse events or outcomes were reported.<br><br>Conclusions. The results of this trial suggest that access to call-in services can increase health knowledge and to some extent also health behaviors. Longer and more intensive program exposure may be needed to yield measurable changes in reproductive outcomes and nutritional status.
**Order of Authors:**	Günther Fink

Powered by Editorial Manager® and ProduXion Manager® from Aries Systems Corporation

	Branwen Nia Owen
	Jan Will
	Ilyena Kozain
**Opposed Reviewers:**	
**Response to Reviewers:**	Reviewer #4:<br><br>Thank you for sharing this study for review. Below are some points that might help the authors revamp their manuscript.<br><br>Thank you for your kind and constructive comments – we have done our best to address all of them as outlined in detail below.<br><br>Abstract<br>It will be beneficial to add analysis section to the methods section of the abstract. Which statistical test were used.<br><br>Thank you for this suggestion. We have added the following sentence to the Abstract to clarify the empirical models used:<br><br>"Linear regression models were used to estimate mean differences in outcomes between treatment and control. Standard errors were corrected for the cluster-based sampling of women."<br><br>If you decide to retain vulnerable population in the title, it will be important to clarify which specific vulnerable population you studied. Otherwise, I will suggest that you remove the vulnerable population in the title and put "women of reproductive age", and hence the background section will need to be revised.<br><br>We have removed the "vulnerable populations" in the background section and now write:<br><br>"..knowledge remains limited among women of reproductive health in many low and middle income countries"<br><br>I am curious about the choice of test used in the data analysis and why the continuous variables<br><br>As stated above, we used linear regression models to estimate mean differences in outcomes between treated and controls. We also considered logistic regression models for the binary outcomes, but decided to stick with mean differences because they make comparisons in outcomes easy to communicate (and use cluster-robust standard errors to address non-normal residuals for binary outcomes).<br><br>Introduction<br>The first paragraph in this section sounds like a problem statement and also this entire section needs to be revised. I suggest that this section be revised as follows:<br>Introduce the reproductive age group as the predominant age group in Uganda<br>Health outcomes common among the reproductive age group (vulnerable population)<br>Emerging role of mobile phone app (Viamo Call-in Information Services)/internet coverage/technology in health education and improvement of reproductive and sexual health outcome in the Ugandan population. The proportion of the Ugandans with access to cell phones and/or internet. Previous studies in the impact of other cell phone apps on reproductive and sexual health outcome. This section should now end with the problem statement and the rational for this study.<br><br>We have restructured the Introduction as suggested and added several additional references. The first paragraph introduces the knowledge gap and highlights the health consequences of this gap for mothers and offsprings. The second paragraph summarizes current research on how to best improve knowledge among women of reproductive age. The third paragraph introduces cell phones as new tool and highlights key studies. The last one provides the objective and rationale of the study.

Powered by Editorial Manager® and ProduXion Manager® from Aries Systems Corporation

Methods

Study design: Were the 4 purposively selected districts representative of the entire Uganda population. This may act as a threat to external validity given that the 4 district doesn't represent the minority regions. I guess this is a limitation.

We agree this is a limitation and acknowledge this in the revised Discussion section, where we write: .

"…it is not clear whether the final set of women enrolled in the study is fully representative of the larger populations in the four study; it is also not clear if the four districts are fully representative of the country as a whole."

Sampling technique: I think the sampling technique you described was a multistage cluster sampling technique and not a 2-stage random sampling. Also take a good look at different studies and revise the entire sampling procedure. The selection of 45 per cluster makes me worry if the selection was proportionate or representative each cluster? In order words, were the clusters of equal population for 45 to be selected from each clusters?

We have changed the description to multi-stage sampling. The sampling was not proportional to population size and likely did not generate a fully representative sample – we say this clearly in the Discussion, where we write:

"Second, we found that the proportion of women using Airtel phones was substantially lower in some rural districts, making it very difficult to reach enrollment targets in some communities. We compensated this by enrolling more women in clusters with higher coverage. Even though a more selected sample clearly does not undermine the internal validity of the study (since randomization was done at the individual level), it is not clear whether the final set of women enrolled in the study is fully representative of the larger populations in the four study districts."

Sample size: How was the sample size of 6000 arrived at? It will make sense to add how it was calculated

We explain this in the revised Power Calculations section, where we write:
"The study was powered to detect a 0.1 standard deviation (SD) difference in standardized knowledge scores (mean 0, SD 1) with power 0.9. Assuming an average cluster size of 30, a follow-up rate of 90% and a design effect of 1.3, this required 100 clusters per arm, and a total sample size of 6000 women."

Randomization: The section on randomization is very scanty and needs clarification. At this point its still not clear what the non-treatment arm received. Did they also benefit from Viamo Call-in Information Services, its important to clearly state what the intervention the 2 groups received. Its important to clearly define the intervention and the non-intervention group (treatment vs control group) and exactly what they received because its not clear.

Apologies for the lack of clarity. We have added the following text to the Randomization section:

"Women selected for the treatment arm were then provided with a personalized introduction to the service (after the baseline survey was completed) and called the service directly from their own phone with the support of the interviewer to see how the system works. Treated women were also given a promotional calendar to serve as a physical reminder on how to navigate the service and the benefits of the service. Women in the control group were not given any intervention. Given that the service was freely available to all women, they were in theory able to call the service at any time, but not given any encouragement or support to do so by the study team."

Primary and secondary outcomes: The definition of primary and secondary outcomes (how were they measured) needs to be included in the methods section. Which tool was used to measure knowledge? Was it pretested or was it a validated tool?

Apologies for the lack of detail here. We now explain this in more detail in the revised Methods section:

"The two primary outcomes of the study were reproductive health knowledge and nutritional supplementation in pregnancy. For reproductive health, we used a 15-item questionnaire of true/false statements (items listed in Supplemental Materials S2). This tool was based on previous studies conducted in sub-Saharan Africa [11] and pretested locally to make sure all items were suitable for the study population. To facilitate interpretation of regression results, we converted the raw scores (number of correct answers out of 15) into z-scores with mean zero and standard deviation one. For nutrition knowledge, we analyzed a binary indicator for women (falsely) believing that herbs can cure anemia. For nutritional supplementation during pregnancy, we used self-reported iron supplementation as an outcome measure. Secondary outcomes included contraceptive knowledge (number of methods respondents are familiar with), contraceptive use (currently using any modern method), prenatal care uptake (any, number of visits), hemoglobin level (as a biomarker indicating actual iron intake), body mass index, and birth spacing.

For pregnancy related outcomes, we analyzed two separate sets of women: first, women reporting to be pregnant at the time of the endline survey (currently pregnant), and second, women who completed a pregnancy in the year preceding the endline survey (recently pregnant). For current pregnancies, we analyzed self-report iron supplementation as well as self-reported use of antenatal care services, and whether women indicate the pregnancy was planned. For recently completed pregnancies, we analyzed the same variables, but also analyzed pregnancy loss and low birth weight (reported weight at birth < 2.5kgs) as additional secondary outcomes. "

Data analysis: I have noticed that the majority of the variables were analyzed as continuous variables. Did you consider converting the continuous variables (scores) to categorical variables which then permits you to use different methods.

We have explained this in further detail in the revised Methods section, where we write:

"All primary models were estimated intent-to-treat, comparing outcomes between treated and control women at endline. For all outcomes, we estimated both unadjusted linear regression models (not including any covariates) and fully adjusted models, where we controlled for age, district, marital status, educational attainment and baseline knowledge variables. The main rationale for using linear regression models was to generate mean differences estimates that could be compared across the rather large set of outcome variables. To adjust for non-normal residuals, we used Huber's cluster-robust standard errors [15]."

Results
The presentation of results is not very clear. The tables are many and its not clear labelled if its correlation coefficient or a p-value.

We agree that there are many, but think it is important to show all of the results – also one of the reasons we chose PLOS ONE for this paper. We have added notes to all tables to be clear about what is shown (coefficients, SEs, p-values). For example, for the first regression Table (T2), the notes now say:

"All point estimates displayed correspond to estimated mean differences in outcomes. Numbers in parentheses are cluster-robust standard errors. Asterisks indicate p-value range of estimated differences: *p-value<0.10, **p-value<0.05, ***p-value<0.01"
Discussion
The discussion needs to be revised. It lack the comparison and contrast of the study findings with studies done locally and international.

There are not so many studies of similar nature, but we try to compare the main outcomes to the related literature in the Discussion, where we write:

"In terms of the primary outcome, our results seem consistent with a recent review of mHealth intervention, which suggests that digital information sources can improve sexual and reproductive health knowledge as well as use of sexual and reproductive

health services [16]. Compared to the estimates reported for an interactive intervention in Ghana [11, 17] the effect sizes observed here seem smaller, which seems plausible given that not all women engaged with the service on a regular service. Compared to previous studies conducted in other parts of Africa we also observed rather high sexual and reproductive health knowledge at baseline – most women were for example familiar with a range of contraceptive methods (beyond traditional methods, condoms and pills), and myths regarding side effects of contraception were less common that seen in other settings [11, 17] – this likely also contributes to the somewhat smaller knowledge impacts seen here. "
Please let us know if there are any papers that we missed and should be cited here.

Reviewer #5:

I appreciate the submission of this manuscript. Please find my comments below, which may be helpful in improving the quality of your work.

Thank you for the kind and helpful comments – we really appreciate your input and have done our best to address all suggestions and explained in further detail below.

Pg. 1/26: It is recommended that the phrase "results from" in the title be omitted, as this would shorten the title without detracting from its intended meaning.

Thank you for this suggestion – we have removed "results from" the title and agree that the shortened version sounds better.

Pg. 2/26: - It is recommended that the phrase "and Outcomes" be included in the title, as currently presented at the end of the background, to ensure that the title is complete.

We believe these titles are given by the journal and thus are not allowed to modify them. We do state the objective in the background; the outcomes are provided in the Methods subsection.

- Please ensure that the sampling method, data collection tools, a brief description of the data collection procedure, and the statistical tests used are clearly described in the methods section.

We have expanded the Methods section to provide more details: it now reads as follows:

"We conducted a 2-arm open label randomized controlled trial involving 6,000 randomly selected women, ages 18-49 years, with access to a simple feature Airtel cell phone in Uganda. Fifty enumeration areas were randomly selected in Kampala, Madi-Okollo, Rwampara and Katakwi districts. Consenting women were asked to complete a baseline and endline survey implemented by trained interviewers using tablets through home visits. After completion of baseline, women were randomized with equal probability to treatment and control using a tablet-generated random number draw. Study staff introduced treated women to the call-in service and encouraged its use through various incentives and promotional messages. Primary outcomes were sexual and reproductive health knowledge scores as well as iron supplementation in pregnancy. Secondary outcomes included self-reported use of contraception, healthy diets, birth spacing and birth outcomes as well as hemoglobin levels and BMI. Linear regression models were used to estimate mean differences in outcomes between treatment and control. Standard errors were corrected for the cluster-based sampling of women."

Pg. 3/26: Please mention keywords at the end of the abstract.

We have added the following keywords at the end of the abstract:

Keywords: reproductive health, call-in-services, health knowledge, Uganda, Viamo.

Pg. 4/26: Please provide further details regarding the Viamo platform and its operational mechanisms. Specifically, clarify whether the platform enables two-way

	interaction and whether users can experience a form of interactive support comparable to counseling, or whether information is delivered in a predominantly one-way manner, functioning mainly as a communication channel for educational purposes.<br>Please review relevant studies that have implemented similar interventions in the same research area, either locally or in other regions. Additionally, address studies that have evaluated the effectiveness of the Viamo platform or comparable mobile- or voice-based platforms for different purposes. Finally, summarize the existing literature and highlight the comparative advantage and necessity of the present intervention by identifying the gaps it aims to address. If no relevant studies are available, this should be clearly stated.<br><br>Based on the feedback provided by Reviewer 4, we have removed the description of the service from the Introduction (page 4) and instead provide more details on the platform in an expanded Methods-Intervention section, where we provide the requested details. We compare this to other studies in the Discussion section – there is however not that much literature on similar models. In practice, Viamo does not provide direct counseling, but it offers a range of different topics users can select from – we think it can be best described as something like a “user-guided learning system”.<br><br>Pg. 5/26 (Methods section):<br>- Please specify the exact nature of any encouragement designs and whether they may have preferentially attracted certain subgroups of participants. For example, as noted on page 7, "Participants received UGX 10,000 (~USD $2.77) of phone credit as a token of gratitude after each survey interview." Offering such incentives could disproportionately attract individuals with lower socioeconomic status, potentially limiting the generalizability. Therefore, if participants were aware of the incentives at the time of study invitation, this should be explicitly stated and discussed in the study limitations.<br><br>We have provided further details on the encouragement in the Methods-Randomization section now, where we write:<br><br>“Women selected for the treatment arm were then provided with a personalized introduction to the service (after the baseline survey was completed) and called the service directly from their own phone with the support of the interviewer to see how the system works. Treated women were also given a promotional calendar to serve as a physical reminder on how to navigate the service and the benefits of the service. Women in the control group were not given any intervention. Given that the service was freely available to all women, they were in theory able to call the service at any time, but not given any encouragement or support to do so by the study team.”<br><br>The UGX 10,000 were proposed by the Ugandan IRB. On the consent form, these were stated, but not as incentive to participate, but simply to “compensate you for the time it will take to complete the survey”. Given that the surveys took up to two hours, this seemed fair – we did not observe any differential participation in the study (i.e. there were almost no refusals).<br><br>- Please specify the exclusion (withdrawal) criteria during the study period, which criteria refer to circumstances that necessitate the removal of participants after enrollment, such as the death of a close family member.<br><br>We did not have any such exclusion criteria during the study and have added this information to the Inclusion and Exclusion criteria section now, where we write:<br><br>“There were no exclusion criteria (or exclusions) during the study.”<br><br>- Please specify the sample size and describe the method used for its calculation in the Methods section.<br><br>We have revised the Power Calculations section to directly explain how the sample size was derived:<br><br>“The study was powered to detect a 0.1 standard deviation (SD) difference in standardized knowledge scores (mean 0, SD 1) with power 0.9. Assuming an average

cluster size of 30, a follow-up rate of 90% and a design effect of 1.3, this required 100 clusters per arm, and a total sample size of 6000 women.”

- Please provide additional details on how participants used the Viamo platform. Clarify whether platform use was need-based and participant-driven or task-oriented as part of the intervention.

We have expanded to Intervention section to provide further details on this. The answer to your question is a mix of both – users could call at any time, but were also (in the treatment group only) encouraged to use the system:

“Treated women were encouraged to use the system whenever they had questions or wanted to know more about sexual and reproductive health. Viamo also employed targeted nudges, personalized campaigns and other outreach activities (such as release of new content), with the aim of maintaining and incentivizing usage rates (active calling into the Viamo Platform) among women in the treatment group (see Supplemental Materials S1 for an example).”

- You mentioned in the limitations that information leak or unintended use of the platform by control group participants may have occurred. Please clarify whether any measures were taken to limit access to the platform or prevent its use by the control group and, if so, describe these measures in the Methods section. In addition, indicate whether any promotional activities (such as social media or other communication channels) were used that might have increased awareness of the platform and led to its use among control group participants.

We were not able to prevent anybody from using the system, and state this clearly now in the revised Randomization section, where we write:

“Women in the control group were not given any intervention. Given that the service was freely available to all women, they were in theory able to call the service at any time, but not given any encouragement or support to do so by the study team.”

We are not aware of any social media or other programs promoting this channel and would have known since we worked closely with Viamo on this project.

Pg. 7/26: Were there specific intervals between each measurement time point? If so, please specify these intervals.

The idea was to have 15 months of program exposure between the two surveys. We have clarified this now in the revised Methods section where we write:

“Baseline data was collected via face-to-face interview at each participants’ home between December 6, 2022 and March 6, 2023. Upon completion of the baseline survey, treated women were introduced to the service by study staff. Endline data was collected approximately 15 months after recruitment, between May 8, 2024 and August 4, 2024 in two separate surveys.”

- If the reproductive health questionnaire used in the study has a specific name or is a standardized tool recognized in the literature, please provide its official name and any relevant references.

This is now explained in the revised Methods section as follows:

“For reproductive health, we used a 15-item questionnaire of true/false statements (items listed in Supplemental Materials S2). This tool was based on previous studies conducted in sub-Saharan Africa [11] and pretested locally to make sure all items were suitable for the study population. To facilitate interpretation of regression results, we converted the raw scores (number of correct answers out of 15) into z-scores with mean zero and standard deviation one.”

- The current description of study variables appears unclear and potentially confusing. The title refers to Sexual and Reproductive Health Knowledge, Behavior, and

Powered by Editorial Manager® and ProduXion Manager® from Aries Systems Corporation

Outcomes. The explanation provided under primary and secondary outcomes could clearly reflect the study objectives and variables if organized and presented systematically. For instance, the terms reproductive health "behavior" and "outcome" seem to overlap; if this is the case, consider removing one term from the title and text to maintain consistency. Otherwise, please provide a clear definition of each variable and explain how it is measured. Similarly, the term "sexual" may overlap with "reproductive health", as behaviors measured under "sexual behavior" might also fall under reproductive health outcomes. Therefore, please clarify this distinction or consider merging the two concepts where appropriate. A concise, organized, and precise description of variables and outcomes will improve understanding of the study's objectives and interpretation of results.

We are sorry for the confusion. "Sexual and reproductive health" is used as an umbrella term in the literature; we agree that it is not clear where the boundaries between "sexual" and "reproductive" are, but think this is not central to our question. For our project, we had a clear theory of change, going from knowledge to behavior and outcomes, and have tried to follow this in the way we present the results. As for the specific variables, we have tried to explain them in the revised Methods section, where we write:

"The two primary outcomes of the study were reproductive health knowledge and nutritional supplementation in pregnancy. For reproductive health, we used a 15-item questionnaire of true/false statements For nutrition knowledge, we analyzed a binary indicator for women (falsely) believing that herbs can cure anemia. For nutritional supplementation during pregnancy, we used self-reported iron supplementation as an outcome measure. Secondary outcomes included contraceptive knowledge (number of methods respondents are familiar with), contraceptive use (currently using any modern method), prenatal care uptake (any, number of visits), haemoglobin level (as a biomarker indicating actual iron intake), body mass index, and birth spacing.

For pregnancy related outcomes, we analyzed two separate sets of women: first, women reporting to be pregnant at the time of the endline survey (currently pregnant), and second, women who completed a pregnancy in the year preceding the endline survey (recently pregnant). For current pregnancies, we analyzed self-report iron supplementation as well as self-reported use of antenatal care services, and whether women indicate the pregnancy was planned. For recently completed pregnancies, we analyzed the same variables, but also analyzed pregnancy loss and low birth weight (reported weight at birth < 2.5kgs) as additional secondary outcomes. "

Pg. 9/26: Please describe the descriptive and inferential statistical tests used in the study, and specify the statistical methods applied for the presentation and interpretation of the tables.

We have clarified our main empirical model as follows:

"All primary models were estimated intent-to-treat, comparing outcomes between treated and control women at endline. For all outcomes, we estimated both unadjusted linear regression models (not including any covariates) and fully adjusted models, where we controlled for age, district, marital status, educational attainment and baseline knowledge variables. The main rationale for using linear regression models was to generate mean differences estimates that could be compared across the rather large set of outcome variables. To adjust for non-normal residuals, we used Huber's cluster-robust standard errors [15]. "

Pg. 11/26: Please mention p-values and place the asterisk indicators (*) next to the p-values only, and not next to other values. Consider this issue for all tables.

Apologies for the lack of clarity. We have edited the legends of all regression tables to clarify what the asterisks mean – the legend for Table 2 now says:

"All point estimates displayed correspond to estimated mean differences in outcomes. Numbers in parentheses are cluster-robust standard errors. Asterisks indicate p-value range of estimated differences: *p-value<0.10, **p-value<0.05, ***p-value<0.01."

Powered by Editorial Manager® and ProduXion Manager® from Aries Systems Corporation

	We have added the same information to all other regression tables.<br><br>Pg. 13/26: In Table 4, you referred to “Beliefs that local herbs cure anemia”; however, this variable has not been mentioned in either the primary or secondary outcomes, nor in the measurement instruments described in the supplementary files. Please provide clarification on this issue.<br><br>Apologies for this omission: we have added this to the Methods section now:<br><br>“For nutrition knowledge, we analyzed a binary indicator for women (falsely) believing that herbs can cure anemia.”<br><br>Pg. 15/26: Since Survey modality is reported at the end of the table, please refrain from repeating it in the outcome rows.<br><br>Good point – we have removed this information from the column heaers.<br><br>Pg. 15-20: Was a specific checklist or questionnaire used to collect the data presented in Tables 5–10? Please clarify this point. If data collection and evaluation were based on a checklist, please describe it in the relevant sections of the manuscript.<br><br>We did not use a checklist – all of these data were collected through a study tool developed for this project and implemented using tablets with the SurveyCTO software.<br><br>Pg. 15-19: The outcomes reported in Tables 5, 7, 8, and 9 may have been influenced by pregnancy and postpartum healthcare recommendations, medication use, or being under the supervision of a physician or nutritionist. Please clarify how these potential confounding factors were addressed in the analyses. If any strategies were implemented to control for them, please describe them explicitly; otherwise, please acknowledge this issue in the limitations section. In addition, it is recommended that the results for pregnant and non-pregnant women be reported separately in these tables.<br><br>We fully agree that these outcomes will be the results of many factors such as health care provider recommendations, but don’t think this is a concern here since treatment is randomized – as long as the randomization worked (which we are very confident here due to the large number of clusters and individual-level randomization), confounding should not be an issue.<br>As for the subsamples, we have reported them separately in the tables.<br><br>- Not all results have been fully interpreted, and the interpretation provided is rather brief. Please expand the Discussion section to provide a more comprehensive interpretation of the study findings. For instance, the observed reduction in pregnancy loss and low birth weight could be interpreted in relation to the level of antenatal care received. In addition, for each outcome discussed, please contextualize the findings within the existing literature by comparing them with results from similar studies and discussing areas of consistency or discrepancy.<br><br>We have expanded the Discussion section a bit and tried to provide a bit more context, but were not able to say much more on the literature, since we could not find many other studies using a similar approach or technology – please let us know if there are any studies that we missed.<br><br>- Please critically assess the generalization of the study findings and clarify the extent to which the results can be applied to other populations or settings.<br>We have added the following paragraph to the Discussion:<br><br>“Even though a more selected sample clearly does not undermine the internal validity of the study (since randomization was done at the individual level), it is not clear whether the final set of women enrolled in the study is fully representative of the larger populations in the four study districts; it is also not clear if the four districts are fully representative of the country as a whole. Since the service is currently only available to Airtel users, this study simply focused on women with access to this service. Even though it seems plausible that similar effects could be found with other phone

	providers, it is not clear if the results of this study can be generalized to the country as a whole or to other countries.”
Additional Information:	
Question	Response
**Financial Disclosure**<br><br>Enter a financial disclosure statement that describes the sources of funding for the work included in this submission. Review the submission guidelines for detailed requirements. View published research articles from *PLOS ONE* for specific examples.<br><br>This statement is required for submission and **will appear in the published article** if the submission is accepted. Please make sure it is accurate.<br><br>**Funded studies**<br>Enter a statement with the following details:<br>• Initials of the authors who received each award<br>• Grant numbers awarded to each author<br>• The full name of each funder<br>• URL of each funder website<br>• Did the sponsors or funders play any role in the study design, data collection and analysis, decision to publish, or preparation of the manuscript?<br><br>Did you receive funding for this work?	Yes
Please add funding details.<br>as follow-up to "**Financial Disclosure**<br><br>Enter a financial disclosure statement that describes the sources of funding for the work included in this submission. Review the submission guidelines for detailed requirements. View published research articles from *PLOS ONE* for specific examples.<br><br>This statement is required for submission and **will appear in the published article** if the submission is accepted. Please make sure it is accurate.	This project was funded through USAID DIV grant number 7200AA21FA00018. The funders had no role in study design, data collection and analysis, decision to publish, or preparation of the manuscript.

**Funded studies**<br>Enter a statement with the following details:<br>• Initials of the authors who received each award<br>• Grant numbers awarded to each author<br>• The full name of each funder<br>• URL of each funder website<br>• Did the sponsors or funders play any role in the study design, data collection and analysis, decision to publish, or preparation of the manuscript?<br><br>Did you receive funding for this work?"	
Please select the country of your main research funder (please select carefully as in some cases this is used in fee calculation).<br>as follow-up to "**Financial Disclosure**<br><br>Enter a financial disclosure statement that describes the sources of funding for the work included in this submission. Review the submission guidelines for detailed requirements. View published research articles from *PLOS ONE* for specific examples.<br><br>This statement is required for submission and **will appear in the published article** if the submission is accepted. Please make sure it is accurate.<br><br>**Funded studies**<br>Enter a statement with the following details:<br>• Initials of the authors who received each award<br>• Grant numbers awarded to each author<br>• The full name of each funder<br>• URL of each funder website<br>• Did the sponsors or funders play any role in the study design, data collection and analysis, decision to publish, or preparation of the manuscript?<br><br>Did you receive funding for this work?"	UNITED STATES - US

**Competing Interests**<br><br>Use the instructions below to enter a competing interest statement for this submission. On behalf of all authors, disclose any competing interests that could be perceived to bias this work—acknowledging all financial support and any other relevant financial or non-financial competing interests.<br><br>This statement is required for submission and **will appear in the published article** if the submission is accepted. Please make sure it is accurate and that any funding sources listed in your Funding Information later in the submission form are also declared in your Financial Disclosure statement.<br><br>View published research articles from *PLOS ONE* for specific examples.<br><br>**NO authors have competing interests**<br><br>Enter: *The authors have declared that no competing interests exist.*<br><br>**Authors with competing interests**<br><br>Enter competing interest details beginning with this statement:<br><br>*I have read the journal's policy and the authors of this manuscript have the following competing interests: [insert competing interests here]*<br><br>* typeset	The first, second and third author declare no competing interest. The last author (IK) is currently employed by Viamo, the company operating the service analyzed. She was not involved in the data analysis and did not modify the way the results are presented or described in any way.
**Ethics Statement**<br><br>Enter an ethics statement for this submission. This statement is required if the study involved:<br><br>• Human participants<br>• Human specimens or tissue<br>• Vertebrate animals or cephalopods	Ethical clearance for the study was obtained from the Mildmay Uganda Research Ethics Committee (MUREC-2022-124), the ethics committee for North-Western Switzerland (AO_2022-00034) as well as by the Innovations for Poverty Action internal review board (#00006083).

• Vertebrate embryos or tissues<br>• Field research<br><br>Write "N/A" if the submission does not require an ethics statement.<br><br>General guidance is provided below. Consult the submission guidelines for detailed instructions. **Make sure that all information entered here is included in the Methods section of the manuscript.**	

Powered by Editorial Manager® and ProduXion Manager® from Aries Systems Corporation

**Format for specific study types**<br><br>**Human Subject Research (involving human participants and/or tissue)**<br>• Give the name of the institutional review board or ethics committee that approved the study<br>• Include the approval number and/or a statement indicating approval of this research<br>• Indicate the form of consent obtained (written/oral) or the reason that consent was not obtained (e.g. the data were analyzed anonymously)<br><br>**Animal Research (involving vertebrate animals, embryos or tissues)**<br>• Provide the name of the Institutional Animal Care and Use Committee (IACUC) or other relevant ethics board that reviewed the study protocol, and indicate whether they approved this research or granted a formal waiver of ethical approval<br>• Include an approval number if one was obtained<br>• If the study involved *non-human primates*, add *additional details* about animal welfare and steps taken to ameliorate suffering<br>• If anesthesia, euthanasia, or any kind of animal sacrifice is part of the study, include briefly which substances and/or methods were applied<br><br>**Field Research**<br><br>Include the following details if this study involves the collection of plant, animal, or other materials from a natural setting:<br>• Field permit number<br>• Name of the institution or relevant body that granted permission	
**Data Availability**<br><br>Authors are required to make all data underlying the findings described fully available, without restriction, and from the time of publication. PLOS allows rare exceptions to address legal and ethical concerns. See the PLOS Data Policy and FAQ for detailed information.	Yes - all data are fully available without restriction

A Data Availability Statement describing where the data can be found is required at submission. Your answers to this question constitute the Data Availability Statement and **will be published in the article**, if accepted.<br><br>**Important:** Stating 'data available on request from the author' is not sufficient. If your data are only available upon request, select 'No' for the first question and explain your exceptional situation in the text box.<br><br>Do the authors confirm that all data underlying the findings described in their manuscript are fully available without restriction?	
**Describe where the data may be found in full sentences. If you are copying our sample text, replace any instances of XXX with the appropriate details.**<br><br>• If the data are **held or will be held in a public repository**, include URLs, accession numbers or DOIs. If this information will only be available after acceptance, indicate this by ticking the box below. For example: *All XXX files are available from the XXX database (accession number(s) XXX, XXX.*).<br>• If the data are all contained **within the manuscript and/or Supporting Information files**, enter the following: *All relevant data are within the manuscript and its Supporting Information files.*<br>• If neither of these applies but you are able to provide **details of access elsewhere**, with or without limitations, please do so. For example:<br><br>*Data cannot be shared publicly because of [XXX]. Data are available from the XXX Institutional Data Access / Ethics Committee (contact via XXX) for researchers who meet the criteria for access to confidential data.*<br><br>*The data underlying the results presented in the study are available from (include the name of the third party*	We have uploaded a replication package file which contains all data as well as the code used to create figures and tables.

and contact information or URL).<br>• This text is appropriate if the data are owned by a third party and authors do not have permission to share the data.<br><br>* typeset	
Additional data availability information:	

Swiss TPH
Swiss Tropical and Public Health Institute

Prof. Dr. Günther Fink
guenther.fink@swisstph.ch
Direct +41 61 284 8354

Professor of Epidemiology
& Household Economics
and Unit Head

To
Alfredo Luis Fort
Academic Editor
PLOS One

Allschwil, January16th 2026

Re: PONE-D-25-05733R1

Dear Dr. Fort,

Please find attached a revised version of our manuscript titled "*Estimating the Impact of Viamo Call-in Information Services on Sexual and Reproductive Health Knowledge, Behavior, and Outcomes among Women of Reproductive Age: A 2-Arm Open Label Randomized Controlled Trial in Uganda*". We have done our best to address the additional comments provided by the reviewers as described in further detail below.

Kind regards,

Günther Fink, PhD
Head, Household Economics and Health System Research Unit
Swiss Tropical and Public Health Institute
Eckenstein-Geigy Professor of Epidemiology & Household Economics
University of Basel
Tel: +41 (0)61 284 8354
guenther.fink@swisstph.ch

Reviewer #4:

Thank you for sharing this study for review. Below are some points that might help the authors revamp their manuscript.

Thank you for your kind and constructive comments – we have done our best to address all of them as outlined in detail below.

Abstract
It will be beneficial to add analysis section to the methods section of the abstract. Which statistical test were used.

Thank you for this suggestion. We have added the following sentence to the Abstract to clarify the empirical models used:

"Linear regression models were used to estimate mean differences in outcomes between treatment and control. Standard errors were corrected for the cluster-based sampling of women."

If you decide to retain vulnerable population in the title, it will be important to clarify which specific vulnerable population you studied. Otherwise, I will suggest that you remove the vulnerable population in the title and put "women of reproductive age", and hence the background section will need to be revised.

We have removed the "vulnerable populations" in the background section and now write:

"..knowledge remains limited among women of reproductive health in many low and middle income countries"

I am curious about the choice of test used in the data analysis and why the continuous variables

As stated above, we used linear regression models to estimate mean differences in outcomes between treated and controls. We also considered logistic regression models for the binary outcomes, but decided to stick with mean differences because they make comparisons in outcomes easy to communicate (and use cluster-robust standard errors to address non-normal residuals for binary outcomes).

Introduction
The first paragraph in this section sounds like a problem statement and also this entire section needs to be revised. I suggest that this section be revised as follows:
Introduce the reproductive age group as the predominant age group in Uganda
Health outcomes common among the reproductive age group (vulnerable population)
Emerging role of mobile phone app (Viamo Call-in Information Services)/internet coverage/technology in health education and improvement of reproductive and sexual health outcome in the Ugandan population. The proportion of the Ugandans with access to cell phones and/or internet. Previous studies in the impact of other cell phone apps on reproductive and sexual health outcome. This section should now end with the problem statement and the rational for this study.

We have restructured the Introduction as suggested and added several additional references. The first paragraph introduces the knowledge gap and highlights the health consequences of this gap for mothers and offsprings. The second paragraph summarizes current research on how to best improve knowledge among women of reproductive age. The third paragraph introduces cell phones as new tool and highlights key studies. The last one provides the objective and rationale of the study.

Methods

Study design: Were the 4 purposively selected districts representative of the entire Uganda population. This may act as a threat to external validity given that the 4 district doesn't represent the minority regions. I guess this is a limitation.

We agree this is a limitation and acknowledge this in the revised Discussion section, where we write: .

"...it is not clear whether the final set of women enrolled in the study is fully representative of the larger populations in the four study; it is also not clear if the four districts are fully representative of the country as a whole."

Sampling technique: I think the sampling technique you described was a multistage cluster sampling technique and not a 2-stage random sampling. Also take a good look at different studies and revise the entire sampling procedure. The selection of 45 per cluster makes me worry if the selection was proportionate or representative each cluster? In order words, were the clusters of equal population for 45 to be selected from each clusters?

We have changed the description to multi-stage sampling. The sampling was not proportional to population size and likely did not generate a fully representative sample – we say this clearly in the Discussion, where we write:

"Second, we found that the proportion of women using Airtel phones was substantially lower in some rural districts, making it very difficult to reach enrollment targets in some communities. We compensated this by enrolling more women in clusters with higher coverage. Even though a more selected sample clearly does not undermine the internal validity of the study (since randomization was done at the individual level), it is not clear whether the final set of women enrolled in the study is fully representative of the larger populations in the four study districts."

Sample size: How was the sample size of 6000 arrived at? It will make sense to add how it was calculated

We explain this in the revised Power Calculations section, where we write:
"The study was powered to detect a 0.1 standard deviation (SD) difference in standardized knowledge scores (mean 0, SD 1) with power 0.9. Assuming an average cluster size of 30, a follow-up rate of 90% and a design effect of 1.3, this required 100 clusters per arm, and a total sample size of 6000 women."

Randomization: The section on randomization is very scanty and needs clarification. At this point its still not clear what the non-treatment arm received. Did they also benefit from Viamo Call-in Information Services, its important to clearly state what the intervention the 2 groups received. Its important to clearly define the intervention and the non-intervention group (treatment vs control group) and exactly what they received because its not clear.

Apologies for the lack of clarity. We have added the following text to the Randomization section:

"Women selected for the treatment arm were then provided with a personalized introduction to the service (after the baseline survey was completed) and called the service directly from their own phone with the support of the interviewer to see how the system works. Treated women were also given a promotional calendar to serve as a physical reminder on how to navigate the service and the benefits of the service. Women in the control group were not given any intervention. Given that the service was freely available to all women, they were in theory able to call the service at any time, but not given any encouragement or support to do so by the study team."

Primary and secondary outcomes: The definition of primary and secondary outcomes (how were they measured) needs to be included in the methods section. Which tool was used to measure knowledge? Was it pretested or was it a validated tool?

Apologies for the lack of detail here. We now explain this in more detail in the revised Methods section:

"The two primary outcomes of the study were reproductive health knowledge and nutritional supplementation in pregnancy. For reproductive health, we used a 15-item questionnaire of true/false statements (items listed in Supplemental Materials S2). This tool was based on previous studies conducted in sub-Saharan Africa [11] and pretested locally to make sure all items were suitable for the study population. To facilitate interpretation of regression results, we converted the raw scores (number of correct answers out of 15) into z-scores with mean zero and standard deviation one. For nutrition knowledge, we analyzed a binary indicator for women (falsely) believing that herbs can cure anemia. For nutritional supplementation during pregnancy, we used self-reported iron supplementation as an outcome measure. Secondary outcomes included contraceptive knowledge (number of methods respondents are familiar with), contraceptive use (currently using any modern method), prenatal care uptake (any, number of visits), hemoglobin level (as a biomarker indicating actual iron intake), body mass index, and birth spacing.

For pregnancy related outcomes, we analyzed two separate sets of women: first, women reporting to be pregnant at the time of the endline survey (currently pregnant), and second, women who completed a pregnancy in the year preceding the endline survey (recently pregnant). For current pregnancies, we analyzed self-report iron supplementation as well as self-reported use of antenatal care services, and whether women indicate the pregnancy was planned. For recently completed pregnancies, we analyzed the same variables, but also analyzed pregnancy loss and low birth weight (reported weight at birth < 2.5kgs) as additional secondary outcomes. "

Data analysis: I have noticed that the majority of the variables were analyzed as continuous variables. Did you consider converting the continuous variables (scores) to categorical variables which then permits you to use different methods.

We have explained this in further detail in the revised Methods section, where we write:

"All primary models were estimated intent-to-treat, comparing outcomes between treated and control women at endline. For all outcomes, we estimated both unadjusted linear regression models (not including any covariates) and fully adjusted models, where we controlled for age, district, marital status, educational attainment and baseline knowledge variables. The main rationale for using linear regression models was to generate mean differences estimates that could be compared across the rather large set of outcome variables. To adjust for non-normal residuals, we used Huber's cluster-robust standard errors [15]."

Results
The presentation of results is not very clear. The tables are many and its not clear labelled if its correlation coefficient or a p-value.

We agree that there are many, but think it is important to show all of the results – also one of the reasons we chose PLOS ONE for this paper. We have added notes to all tables to be clear about what is shown (coefficients, SEs, p-values). For example, for the first regression Table (T2), the notes now say:

*"All point estimates displayed correspond to estimated mean differences in outcomes. Numbers in parentheses are cluster-robust standard errors. Asterisks indicate p-value range of estimated differences: *p-value<0.10, **p-value<0.05, ***p-value<0.01"*

Discussion
The discussion needs to be revised. It lack the comparison and contrast of the study findings with studies done locally and international.

There are not so many studies of similar nature, but we try to compare the main outcomes to the related literature in the Discussion, where we write:

"In terms of the primary outcome, our results seem consistent with a recent review of mHealth intervention, which suggests that digital information sources can improve sexual and reproductive health knowledge as well as use of sexual and reproductive health services [16]. Compared to the estimates reported for an interactive intervention in Ghana [11, 17] the effect sizes observed here seem smaller, which seems plausible given that not all women engaged with the service on a regular service. Compared to previous studies conducted in other parts of Africa we also observed rather high sexual and reproductive health knowledge at baseline – most women were for example familiar with a range of contraceptive methods (beyond traditional methods, condoms and pills), and myths regarding side effects of contraception were less common that seen in other settings [11, 17] – this likely also contributes to the somewhat smaller knowledge impacts seen here. "

Please let us know if there are any papers that we missed and should be cited here.

Reviewer #5:

I appreciate the submission of this manuscript. Please find my comments below, which may be helpful in improving the quality of your work.

Thank you for the kind and helpful comments – we really appreciate your input and have done our best to address all suggestions and explained in further detail below.

Pg. 1/26: It is recommended that the phrase "results from" in the title be omitted, as this would shorten the title without detracting from its intended meaning.

Thank you for this suggestion – we have removed "results from" the title and agree that the shortened version sounds better.

Pg. 2/26: - It is recommended that the phrase "and Outcomes" be included in the title, as currently presented at the end of the background, to ensure that the title is complete.

We believe these titles are given by the journal and thus are not allowed to modify them. We do state the objective in the background; the outcomes are provided in the Methods subsection.

- Please ensure that the sampling method, data collection tools, a brief description of the data collection procedure, and the statistical tests used are clearly described in the methods section.

We have expanded the Methods section to provide more details: it now reads as follows:

"We conducted a 2-arm open label randomized controlled trial involving 6,000 randomly selected women, ages 18-49 years, with access to a simple feature Airtel cell phone in Uganda. Fifty enumeration areas were randomly selected in Kampala, Madi-Okollo, Rwampara and Katakwi districts. Consenting women were asked to complete a baseline and endline survey implemented by trained interviewers using tablets through home visits. After completion of baseline, women were randomized with equal probability to treatment and control using a tablet-generated random number draw. Study staff introduced treated women to the call-in service and encouraged its use through various incentives and promotional messages. Primary outcomes were sexual and reproductive health knowledge scores as well as iron supplementation in pregnancy. Secondary outcomes included self-reported use of contraception, healthy diets, birth spacing and birth outcomes as well as hemoglobin levels and BMI. Linear regression models were used to estimate mean differences in outcomes between treatment and control. Standard errors were corrected for the cluster-based sampling of women."

Pg. 3/26: Please mention keywords at the end of the abstract.

We have added the following keywords at the end of the abstract:

***Keywords**: reproductive health, call-in-services, health knowledge, Uganda, Viamo.*

Pg. 4/26: Please provide further details regarding the Viamo platform and its operational mechanisms. Specifically, clarify whether the platform enables two-way interaction and whether users can experience a form of interactive support comparable to counseling, or whether information is delivered in a predominantly one-way manner, functioning mainly as a communication channel for educational purposes.
Please review relevant studies that have implemented similar interventions in the same research area, either locally or in other regions. Additionally, address studies that have evaluated the effectiveness of the

Viamo platform or comparable mobile- or voice-based platforms for different purposes. Finally, summarize the existing literature and highlight the comparative advantage and necessity of the present intervention by identifying the gaps it aims to address. If no relevant studies are available, this should be clearly stated.

Based on the feedback provided by Reviewer 4, we have removed the description of the service from the Introduction (page 4) and instead provide more details on the platform in an expanded Methods-Intervention section, where we provide the requested details. We compare this to other studies in the Discussion section – there is however not that much literature on similar models. In practice, Viamo does not provide direct counseling, but it offers a range of different topics users can select from – we think it can be best described as something like a “user-guided learning system”.

Pg. 5/26 (Methods section):
- Please specify the exact nature of any encouragement designs and whether they may have preferentially attracted certain subgroups of participants. For example, as noted on page 7, "Participants received UGX 10,000 (~USD $2.77) of phone credit as a token of gratitude after each survey interview." Offering such incentives could disproportionately attract individuals with lower socioeconomic status, potentially limiting the generalizability. Therefore, if participants were aware of the incentives at the time of study invitation, this should be explicitly stated and discussed in the study limitations.

We have provided further details on the encouragement in the Methods-Randomization section now, where we write:

“Women selected for the treatment arm were then provided with a personalized introduction to the service (after the baseline survey was completed) and called the service directly from their own phone with the support of the interviewer to see how the system works. Treated women were also given a promotional calendar to serve as a physical reminder on how to navigate the service and the benefits of the service. Women in the control group were not given any intervention. Given that the service was freely available to all women, they were in theory able to call the service at any time, but not given any encouragement or support to do so by the study team.”

The UGX 10,000 were proposed by the Ugandan IRB. On the consent form, these were stated, but not as incentive to participate, but simply to “compensate you for the time it will take to complete the survey”. Given that the surveys took up to two hours, this seemed fair – we did not observe any differential participation in the study (i.e. there were almost no refusals).

- Please specify the exclusion (withdrawal) criteria during the study period, which criteria refer to circumstances that necessitate the removal of participants after enrollment, such as the death of a close family member.

We did not have any such exclusion criteria during the study and have added this information to the Inclusion and Exclusion criteria section now, where we write:

“There were no exclusion criteria (or exclusions) during the study.”

- Please specify the sample size and describe the method used for its calculation in the Methods section.

We have revised the Power Calculations section to directly explain how the sample size was derived:

"The study was powered to detect a 0.1 standard deviation (SD) difference in standardized knowledge scores (mean 0, SD 1) with power 0.9. Assuming an average cluster size of 30, a follow-up rate of 90% and a design effect of 1.3, this required 100 clusters per arm, and a total sample size of 6000 women."

- Please provide additional details on how participants used the Viamo platform. Clarify whether platform use was need-based and participant-driven or task-oriented as part of the intervention.

We have expanded to Intervention section to provide further details on this. The answer to your question is a mix of both – users could call at any time, but were also (in the treatment group only) encouraged to use the system:

"Treated women were encouraged to use the system whenever they had questions or wanted to know more about sexual and reproductive health. Viamo also employed targeted nudges, personalized campaigns and other outreach activities (such as release of new content), with the aim of maintaining and incentivizing usage rates (active calling into the Viamo Platform) among women in the treatment group (see Supplemental Materials S1 for an example)."

- You mentioned in the limitations that information leak or unintended use of the platform by control group participants may have occurred. Please clarify whether any measures were taken to limit access to the platform or prevent its use by the control group and, if so, describe these measures in the Methods section. In addition, indicate whether any promotional activities (such as social media or other communication channels) were used that might have increased awareness of the platform and led to its use among control group participants.

We were not able to prevent anybody from using the system, and state this clearly now in the revised Randomization section, where we write:

"Women in the control group were not given any intervention. Given that the service was freely available to all women, they were in theory able to call the service at any time, but not given any encouragement or support to do so by the study team."

We are not aware of any social media or other programs promoting this channel and would have known since we worked closely with Viamo on this project.

Pg. 7/26: Were there specific intervals between each measurement time point? If so, please specify these intervals.

The idea was to have 15 months of program exposure between the two surveys. We have clarified this now in the revised Methods section where we write:

"Baseline data was collected via face-to-face interview at each participants' home between December 6, 2022 and March 6, 2023. Upon completion of the baseline survey, treated women were introduced to the service by study staff. Endline data was collected approximately 15 months after recruitment, between May 8, 2024 and August 4, 2024 in two separate surveys."

- If the reproductive health questionnaire used in the study has a specific name or is a standardized tool recognized in the literature, please provide its official name and any relevant references.

This is now explained in the revised Methods section as follows:

“For reproductive health, we used a 15-item questionnaire of true/false statements (items listed in Supplemental Materials S2). This tool was based on previous studies conducted in sub-Saharan Africa [11] and pretested locally to make sure all items were suitable for the study population. To facilitate interpretation of regression results, we converted the raw scores (number of correct answers out of 15) into z-scores with mean zero and standard deviation one.”

- The current description of study variables appears unclear and potentially confusing. The title refers to Sexual and Reproductive Health Knowledge, Behavior, and Outcomes. The explanation provided under primary and secondary outcomes could clearly reflect the study objectives and variables if organized and presented systematically. For instance, the terms reproductive health "behavior" and "outcome" seem to overlap; if this is the case, consider removing one term from the title and text to maintain consistency. Otherwise, please provide a clear definition of each variable and explain how it is measured. Similarly, the term "sexual" may overlap with "reproductive health", as behaviors measured under "sexual behavior" might also fall under reproductive health outcomes. Therefore, please clarify this distinction or consider merging the two concepts where appropriate. A concise, organized, and precise description of variables and outcomes will improve understanding of the study’s objectives and interpretation of results.

We are sorry for the confusion. “Sexual and reproductive health” is used as an umbrella term in the literature; we agree that it is not clear where the boundaries between “sexual” and “reproductive” are, but think this is not central to our question. For our project, we had a clear theory of change, going from knowledge to behavior and outcomes, and have tried to follow this in the way we present the results. As for the specific variables, we have tried to explain them in the revised Methods section, where we write:

“The two primary outcomes of the study were reproductive health knowledge and nutritional supplementation in pregnancy. For reproductive health, we used a 15-item questionnaire of true/false statements For nutrition knowledge, we analyzed a binary indicator for women (falsely) believing that herbs can cure anemia. For nutritional supplementation during pregnancy, we used self-reported iron supplementation as an outcome measure. Secondary outcomes included contraceptive knowledge (number of methods respondents are familiar with), contraceptive use (currently using any modern method), prenatal care uptake (any, number of visits), haemoglobin level (as a biomarker indicating actual iron intake), body mass index, and birth spacing.

For pregnancy related outcomes, we analyzed two separate sets of women: first, women reporting to be pregnant at the time of the endline survey (currently pregnant), and second, women who completed a pregnancy in the year preceding the endline survey (recently pregnant). For current pregnancies, we analyzed self-report iron supplementation as well as self-reported use of antenatal care services, and whether women indicate the pregnancy was planned. For recently completed pregnancies, we analyzed the same variables, but also analyzed pregnancy loss and low birth weight (reported weight at birth < 2.5kgs) as additional secondary outcomes. “

Pg. 9/26: Please describe the descriptive and inferential statistical tests used in the study, and specify the statistical methods applied for the presentation and interpretation of the tables.

We have clarified our main empirical model as follows:

“All primary models were estimated intent-to-treat, comparing outcomes between treated and control women at endline. For all outcomes, we estimated both unadjusted linear regression models (not including any covariates) and fully adjusted models, where we controlled for age, district, marital status, educational attainment and baseline knowledge variables. The main rationale for using linear regression models was to generate mean differences estimates that could be compared across the rather large set of outcome variables. To adjust for non-normal residuals, we used Huber’s cluster-robust standard errors [15]. “

Pg. 11/26: Please mention p-values and place the asterisk indicators (*) next to the p-values only, and not next to other values. Consider this issue for all tables.

Apologies for the lack of clarity. We have edited the legends of all regression tables to clarify what the asterisks mean – the legend for Table 2 now says:

*"All point estimates displayed correspond to estimated mean differences in outcomes. Numbers in parentheses are cluster-robust standard errors. Asterisks indicate p-value range of estimated differences: *p-value<0.10, **p-value<0.05, ***p-value<0.01."*

We have added the same information to all other regression tables.

Pg. 13/26: In Table 4, you referred to "Beliefs that local herbs cure anemia"; however, this variable has not been mentioned in either the primary or secondary outcomes, nor in the measurement instruments described in the supplementary files. Please provide clarification on this issue.

Apologies for this omission: we have added this to the Methods section now:

"For nutrition knowledge, we analyzed a binary indicator for women (falsely) believing that herbs can cure anemia."

Pg. 15/26: Since Survey modality is reported at the end of the table, please refrain from repeating it in the outcome rows.

Good point – we have removed this information from the column heaers.

Pg. 15-20: Was a specific checklist or questionnaire used to collect the data presented in Tables 5–10? Please clarify this point. If data collection and evaluation were based on a checklist, please describe it in the relevant sections of the manuscript.

We did not use a checklist – all of these data were collected through a study tool developed for this project and implemented using tablets with the SurveyCTO software.

Pg. 15-19: The outcomes reported in Tables 5, 7, 8, and 9 may have been influenced by pregnancy and postpartum healthcare recommendations, medication use, or being under the supervision of a physician or nutritionist. Please clarify how these potential confounding factors were addressed in the analyses. If any strategies were implemented to control for them, please describe them explicitly; otherwise, please acknowledge this issue in the limitations section. In addition, it is recommended that the results for pregnant and non-pregnant women be reported separately in these tables.

We fully agree that these outcomes will be the results of many factors such as health care provider recommendations, but don't think this is a concern here since treatment is randomized – as long as the randomization worked (which we are very confident here due to the large number of clusters and individual-level randomization), confounding should not be an issue.
As for the subsamples, we have reported them separately in the tables.

- Not all results have been fully interpreted, and the interpretation provided is rather brief. Please expand the Discussion section to provide a more comprehensive interpretation of the study findings. For instance, the observed reduction in pregnancy loss and low birth weight could be interpreted in relation to the level of antenatal care received. In addition, for each outcome discussed, please contextualize the findings

within the existing literature by comparing them with results from similar studies and discussing areas of consistency or discrepancy.

We have expanded the Discussion section a bit and tried to provide a bit more context, but were not able to say much more on the literature, since we could not find many other studies using a similar approach or technology – please let us know if there are any studies that we missed.

- Please critically assess the generalization of the study findings and clarify the extent to which the results can be applied to other populations or settings.
We have added the following paragraph to the Discussion:

"Even though a more selected sample clearly does not undermine the internal validity of the study (since randomization was done at the individual level), it is not clear whether the final set of women enrolled in the study is fully representative of the larger populations in the four study districts; it is also not clear if the four districts are fully representative of the country as a whole. Since the service is currently only available to Airtel users, this study simply focused on women with access to this service. Even though it seems plausible that similar effects could be found with other phone providers, it is not clear if the results of this study can be generalized to the country as a whole or to other countries."

Manuscript
Click here to access/download;Manuscript;Manuscript 19 January 2026.docx

Estimating the Impact of Viamo Call-in Information Services on Sexual and Reproductive Health Knowledge, Behavior, and Outcomes among Women of Reproductive Age: A 2-Arm Open Label Randomized Controlled Trial in Uganda

Günther Fink[1,2*], Branwen Nia Owen[1,2], Jan Will[3], Ilyena Kozain[4]

1 University of Basel, Basel, Switzerland

2 Swiss Tropical and Public Health Institute, Allschwil, Switzerland

3 Innovations for Poverty Action, Kampala, Uganda

4 Viamo, Kampala, Uganda

*** Corresponding author:** Swiss Tropical and Public Health Institute, Kreuzstrasse 2, 4123 Allschwil, Switzerland. Email: guenther.fink@swisstph.ch.

Abstract

Background. Despite substantial increases in access to schooling and to the internet, health information and knowledge remains limited among women of reproductive health in many low and middle income countries. Given the near universal cell-phone coverage in low- and middle-income countries, call-in services offer an interesting new platform to provide high quality information to targeted populations. In this paper, we assessed the impact of the Viamo 3-2-1 call-in Platform on health knowledge and behavior among women of reproductive age in Uganda.

Methods. We conducted a 2-arm open label randomized controlled trial involving 6,000 randomly selected women, ages 18-49 years, with access to a simple feature Airtel cell phone in Uganda. Fifty enumeration areas were randomly selected in Kampala, Madi-Okollo, Rwampara and Katakwi districts. Consenting women were asked to complete a baseline and endline survey implemented by trained interviewers using tablets through home visits. After completion of baseline, women were randomized with equal probability to treatment and control using a tablet-generated random number draw. Study staff introduced treated women to the call-in service and encouraged its use through various incentives and promotional messages. Primary outcomes were sexual and reproductive health knowledge scores as well as iron supplementation in pregnancy. Secondary outcomes included self-reported use of contraception, healthy diets, birth spacing and birth outcomes as well as hemoglobin levels and BMI. Linear regression models were used to estimate mean differences in outcomes between treatment and control. Standard errors were corrected for the cluster-based sampling of women.

Results. A total of 6,011 women were enrolled into the study between December 6, 2022 and February 11, 2023; 3,052 were assigned to treatment, 2,959 used as controls. Endline data was collected between May 8, 2024 and August 4, 2024. Overall follow-up rates were 92% in both the treatment and the control group. Treatment increased average sexual and reproductive health knowledge by 0.07 standard deviations (95% CI [0.01, 0.11], p-value = 0.02) and increased the likelihood of taking iron supplements by 4% pts among recently pregnant women

([0.003, 0.08], p-value 0.04). Results for secondary outcomes were mixed, with positive changes for nutritional intake and family planning behaviors and intentions and no impact on hemoglobin levels and BMI. No adverse events or outcomes were reported.

Conclusions. The results of this trial suggest that access to call-in services can increase health knowledge and to some extent also health behaviors. Longer and more intensive program exposure may be needed to yield measurable changes in reproductive outcomes and nutritional status.

Keywords: reproductive health, call-in-services, health knowledge, Uganda, Viamo.

Introduction

In low- and middle-income countries (LMIC), access to timely and accurate information in national languages is often limited, particularly among the poor. Whereas the internet is a common source of information in many countries, only 10% of Ugandans, and 36% of Sub-Saharan Africans were estimated to use the internet in 2021; far lower than the global average of 63% [1]. This lack of information access can contribute to poor health decisions; this is particularly important for women of reproductive age, since lack of information often leads to inadequate prenatal care [2] and ineffective family planning methods [3], threatening not only the life of mothers [4] but also their children [5, 6].

Public health messaging in LMICs is often disseminated through mass media channels such as radio messages and billboards. These channels have proven to be effective at reaching large numbers of people [7] and increasing knowledge, but have had an insufficient impact on changing behaviors in many areas of public health [8, 9]. Mass media campaigns often fail to create behavior change because, in part, people cannot access the information at a moment of need.

The rapid growth in cell phone coverage in recent years [10] offers new opportunities to address this gap, as information can be accessed at any time, from anywhere. Recent evidence suggests that interventions to improve reproductive health knowledge through easy access technologies can effectively improve ~~reproductive health~~ knowledge and use of modern contraception [11-13].

In this study, we aimed to assess whether providing access to call-in-services – free numbers that women can dial to obtain information on health – can effectively improve sexual and reproductive health knowledge and attitudes among women of reproductive health in Uganda.

Methods

Study Design

This study was designed as a two-arm, non-blinded, individual-level randomised controlled trial focusing on women's nutrition and reproductive health. Given that the main intervention – a phone-based information service – was equally available to both arms, we used an encouragement design, actively introducing women in the treated arm to the service and

encouraging them to use it throughout the study period. Blinding of the intervention was not possible given the nature of the program.

Study Setting and Participant Recruitment

This study was conducted in four districts of Uganda: Kampala, Katakwi, Madi-Okollo and Rwampara. These four districts were purposely chosen to cover all major regions of Uganda. To recruit women within these four districts, a multi-stage sampling process was followed: first, a list of all enumeration areas in each district was obtained, and 50 enumeration areas were randomly chosen using a simple random number draw created with the Stata statistical software package. In rural areas, enumeration areas corresponded to villages, and in urban areas, to residential blocks. All households within each selected enumeration area were visited to identify eligible women. Fig 1 shows the spatial location of the study districts within Uganda.

Inclusion and exclusion criteria

In a second step, study eligibility was assessed by trained study enumerators. Women living in selected enumeration areas were eligible for inclusion in the study if they were aged 18-49 years and had access to an Airtel (a mobile network operator) phone number within their household. Given the explicit focus on antenatal care, family planning and post-partum, all women who were pregnant during the listing were automatically invited to join the study; non-pregnant women were randomly selected for the study until the 45 women per cluster target was reached. In clusters with less than 45 women, all women were invited to participate in the study.

Women were ineligible if they had used the Viamo platform previously, they planned to move outside of the study area within 12 months of baseline or if they did not speak one of the six languages available on the platform: Ateso, English, Luganda, Lugbara, Luo or Runyakitara.

There were no exclusion criteria (or exclusions) during the study.

[Figure 1 about here]

Intervention

Viamo's platform was created with the objective of addressing the need for accessible and timely health information. Through an easy to remember dial-in number, callers can obtain up-to-date news as well as key information on topics related to nutrition, maternal and child health, and sexual and reproductive health. The platform is free of charge, and currently has over 4 million users in Uganda, and 35 million users in LMICs globally [14]. Users who call hear a pre-recorded menu, and can choose topics to listen to from these menus.

In Uganda, the Viamo platform is available via voice and text channels on all mobile phones with an Airtel number and offers a wide variety of health and other information to all interested callers.

For the study, we aimed to generate random variation in system usage by providing strong encouragement to use the system to a randomly selected subset of women. Specifically, to ensure increased service utilization among women randomly selected for the treatment, all treated women in the sample received:

1. A personalized introduction and mock call into platform (at time of baseline interview)
2. A promotional calendar to serve as a physical reminder on how to navigate the service and the benefits of the service (at time of baseline interview)
3. Continued encouragement to use the service in the form of text messages and outbound calls, incentivizing women to call the service.

The original sixty sexual and reproductive, maternal and neonatal health and nutrition key messages launched at the inception of the project were developed in conjunction with subject experts, end users, communication specialists, and the Ugandan Ministry of Health (MOH). Throughout the implementation period, the key messages (2-4 mins of dialogue) were transformed into stylized dynamic content (7-45 mins), to reiterate critical concepts for enhanced learning and be able to promote new content. The release o[illegible]IV-dynamic content formats (dramas, talk shows, and celebrity interviews) was staggered and began in August 2022.

Treated women were encouraged to use the system whenever they had questions or wanted to know more about sexual and reproductive health. Viamo also employed targeted nudges, personalized campaigns and other outreach activities (such as release of new content), with the

aim of maintaining and incentivizing usage rates (active calling into the Viamo Platform) among women in the treatment group (see Supplemental Materials S1 for an example).

Study Procedures

Study staff visited selected women at their homes, introduced the study and invited them to participate. The consent form was read aloud and explained when necessary. Literate women signed the consent form and illiterate women marked the form with a thumbprint, and a literate witness was asked to sign on their behalf. We did not assess participants' cognitive capacity to provide consent.

Baseline data was collected via face-to-face interview at each participants' home between December 6, 2022 and March 6, 2023. Upon completion of the baseline survey, treated women were introduced to the service by study staff. Endline data was collected approximately 15 months after recruitment, between May 8, 2024 and August 4, 2024 in two separate surveys; a phone survey and a face-to-face in-person survey (see Supplementary Table 1 for dates by district, and Supplementary Table 2 for data collected by phone and in-person interview at endline). During all three surveys, the interviewer recorded responses on a tablet using the SurveyCTO software. Data collectors received training and on-going support and monitoring to ensure data quality. Participants received UGX10,000 (~USD $2.77) of phone credit as a token of gratitude after each survey interview.

Surveys collected data on socio-demographic characteristics, knowledge and use of the Viamo platform, reproductive health knowledge, contraceptive use, pregnancy planning and uptake of antenatal care, pregnancy outcomes, diet and exercise behaviors and select biomarkers.

Randomization

Upon completion of the baseline survey, we randomised arm allocation at the individual level based on a simple number draw generated by the SurveyCT[illegible]ftware. Specifically, at the end of the survey, the software generated a random number from a uniform distribution [0,1] and allocated all draws > 0.5 to treatment.

Women selected for the treatment arm were then provided with a personalized introduction to the service (after the baseline survey was completed) and called the service directly from their own phone with the support of the interviewer to see how the system works. Treated women were also given a promotional calendar to serve as a physical reminder on how to navigate the service and the benefits of the service. Women in the control group were not given any

intervention. Given that the service was freely available to all women, they were in theory able to call the service at any time, but not given any encouragement or support to do so by the study team.

Power Calculations

The study was powered to detect a 0.1 standard deviation (SD) difference in standardized knowledge scores (mean 0, SD 1) with power 0.9. Assuming an average cluster size of 30, a follow-up rate of 90% and a design effect of 1.3, this required 100 clusters per arm, and a total sample size of 6000 women. The targeted effect size was based on an anticipated knowledge score difference of 0.2 SD among compliers (system users), and an average compliance of 10% in the control, and 60% in the treatment arm (a net compliance/usage differential of 50%).

Primary and secondary outcomes

The two primary outcomes of the study were reproductive health knowledge and nutritional supplementation in pregnancy. For reproductive health, we used a 15-item questionnaire of true/false statements (items listed in Supplemental Materials S2). This tool was based on previous studies conducted in sub-Saharan Africa [11] and pretested locally to make sure all items were suitable for the study population. To facilitate interpretation of regression results, we converted the raw scores (number of correct answers out of 15) into z-scores with mean zero and standard deviation one. For nutrition knowledge, we analyzed a binary indicator for women (falsely) believing that herbs can cure anemia. For nutritional supplementation during pregnancy, we used self-reported iron supplementation as an outcome measure. Secondary outcomes included contraceptive knowledge (number of methods respondents are familiar with), contraceptive use (currently using any modern method), prenatal care uptake (any, number of visits), hemoglobin level (as a biomarker indicating actual iron intake), body mass index, and birth spacing.

For pregnancy related outcomes, we analyzed two separate sets of women: first, women reporting to be pregnant at the time of the endline survey (currently pregnant), and second, women who completed a pregnancy in the year preceding the endline survey (recently pregnant). For current pregnancies, we analyzed self-report iron supplementation as well as self-reported use of antenatal care services, and whether women indicate the pregnancy was planned. For recently completed pregnancies, we analyzed the same variables, but also

analyzed pregnancy loss and low birth weight (reported weight at birth < 2.5kgs) as additional secondary outcomes.

Statistical Analysis

We began our analysis with a comparison of baseline characteristics across arms, as well as a comparison of follow-up rates to assess potential selective attrition. All primary models were estimated intent-to-treat, comparing outcomes between treated and control women at endline. For all outcomes, we estimated both unadjusted linear regression models (not including any covariates) and fully adjusted models, where we controlled for age, district, marital status, educational attainment and baseline knowledge variables. The main rationale for using linear regression models was to generate mean differences estimates that could be compared across the rather large set of outcome variables. To adjust for non-normal residuals, we used Huber's cluster-robust standard errors [15].

We used forest plots to illustrate item level changes for aggregated reproductive health knowledge and contraceptive use scores. In an exploratory analysis, we also estimated per protocol models and stratified models. Per protocol models were restricted to compliant women: specifically, we included only women in the control group who had never called the service, and women in the treatment group who had called the service at least five times. To investigate differential impact on subgroups, we estimated stratified models by age group (<30, 30+), educational attainment group (primary or less, secondary, higher) and district.

Study registration and ethical clearance

The trial was registered on June 03, 2022 at https://www.socialscienceregistry.org/trials/9544. Ethical clearance for the study was obtained from the Mildmay Uganda Research Ethics Committee (MUREC-2022-124), the ethics committee for North-Western Switzerland (AO_2022-00034) as well as by the Innovations for Poverty Action internal review board (#00006083).

Results

Of 54,259 women screened, 29% (n=15,532) were eligible to participate in the study (Fig 2). The most common reason for ineligibility was not having access to an Airtel number. A total of 6,011 women completed the baseline survey and were randomised to the control (n=3,052) or the intervention arm (n=2,959). At endline, 87% (n=2,678) of control arm participants, and

86% (n=2,555) of intervention arm participants completed the phone survey. Slightly fewer participants completed the in-person survey (81% of both arms). 92% of participants in both arms completed at least one of the two modalities (i.e. either a phone or an in-person survey). The most common reason for loss to follow were participants being unreachable by phone, and participants having moved house, in the phone survey and in-person survey, respectively.

[Figure 2 about here]

Table 1 shows descriptive statistics for the study sample. Over half were under 30 years old (53%), and a majority were currently married (58%)[illegible]welve percent (12%) of subjects were recruited in Madi-Okollo, 21% in Katakwi, 32% in Rwampara and 35% in Kampala. Fewer than half of women lived in a household with piped water (47%) and very few had health insurance (2%), but most owned their own phone (84%). Most had previously been pregnant (88%) and nearly a seventh were currently pregnant (14%). The trial arms were well-balanced, with all characteristics very similar across arms (Table 1).

Table 1: Sample characteristics overall and by study arm

	Overall (n=6,011)	Control (n=3,052)	Treatment (n=2,959)
Characteristic	N (%)	N (%)	N (%)
Age 18-29	3,159 (53%)	1,609 (53%)	1,550 (52%)
Age 30-39	1,913 (32%)	979 (32%)	934 (32%)
Age 40+	939 (16%)	464 (15%)	475 (16%)
Currently married	3,503 (58%)	1,756 (58%)	1,747 (59%)
No education	159 (2.6%)	95 (3.1%)	64 (2.2%)
Primary education	3,173 (53%)	1,640 (54%)	1,533 (52%)
Secondary or higher education	2,611 (43%)	1,285 (42%)	1,326 (45%)
Education: other	68 (1.1%)	32 (1.0%)	36 (1.2%)
Speaks English	1,716 (29%)	862 (28%)	854 (29%)
Kampala	2,081 (35%)	1,071 (35%)	1,010 (34%)
Katakwi district	1,256 (21%)	619 (20%)	637 (22%)
Madi-Okollo district	741 (12%)	385 (13%)	356 (12%)
Rwampara district	1,933 (32%)	977 (32%)	956 (32%)
Household has piped water	2,799 (47%)	1,431 (47%)	1,368 (46%)
Household has health insurance	100 (1.7%)	45 (1.5%)	55 (1.9%)
Woman has her own phone	5,056 (84%)	2,527 (83%)	2,529 (85%)
Currently pregnant	867 (14%)	441 (14%)	426 (14%)
Previously pregnant (ever)	5,275 (88%)	2,683 (88%)	2,592 (88%)

Table 2 summarizes overall compliance with treatment and platform use. 50% of women in the control group indicated at endline that they had heard of the Viamo platform compared to 96% in the intervention arm. 20% of women in the control arm reported to have ever used the platform, and 7% reported to have used it in the last month. In the treatment arm, the same was

true for 84% and 42% respectively. On average, subjects in the control arm reported four calls to the platform, women in the treatment arm reported an additional 18 calls on average. These numbers also align nicely with the Viamo platform metrics, which recorded an average of four calls for the control group, and an additional 31 calls among women in the treatment group (suggesting that on average women under-reported the number of actual calls they made). In a given month, a treatment user listened to an average of approximately 38 minutes of content on the platform

Table 2: Treatment Compliance

Outcome	Heard of the platform	Ever used the platform	Used the platform in the last month	Total number of self-reported calls to platform [a)]	Total number of registered calls to platform
Treatment effect	0.459***	0.642***	0.346***	18.25***	31.41***
	(0.0192)	(0.0143)	(0.0129)	(0.471)	(0.958)
Control group average	0.499	0.499	0.0709	4.075	4.012
Observations	5,233	5,233	5,233	5,233	5,233
R-squared	0.262	0.412	0.164	0.268	0.271

Notes: All point estimates displayed correspond to estimated mea[illegible]fferences in outcomes. Numbers in parentheses are cluster-robust standard errors. Asterisks indicate p-value range of estimated differences: *p-value<0.10, **p-value<0.05, ***p-value<0.01. [a)] Self-reported number of calls were top-coded at 51 calls.

Table 3 provides an overview over the most popular content. The most popular single item was the local weather forecast, followed by information on food and general news. On average, 71% of the content listened to by women in the treatment group accessing the platform (n=2,825) was content specifically related to maternal health, sexual and reproductive health, family planning, or nutrition. For the control group members who accessed the platform (n=1,024) only 20% listened to the same thematically relevant content for this study.

Table 3. Top ten most popular messages listened to by treatment group on the platform (Dec 2023 - July 2024)

No	Message Title	Key Themes	Times Reached	Times Listened	Total Number of Listeners	C2L Ratio
1	Dynamic daily weather report	weather	14140	14140	2140	100%
2	To learn about what food keeps us healthy	health, nutrition, anemia	8858	6710	1753	89%
3	Dynamic daily news bulletin	news	5959	4488	1046	86%
4	To know what a healthy weight is	health, nutrition, BMI	4335	3585	1310	92%
5	To learn more about cervical cancer	SRH	4056	3417	1179	91%
6	What is folic acid and why take it?	health, pregnancy	4006	3177	1208	89%
7	What is sex?	puberty, SRH	3852	3500	1513	95%
8	DIV Drama Episode 1: "Family planning for a better future"	FP, SRH	3675	2053	633	77%
9	To know the four ways to improve your chance for a healthy pregnancy	SRH, pregnancy	3660	2205	1453	78%
10	To learn how iron keeps us strong	nutrition, anemia	3558	2950	1092	89%

*Note*s: A listener is defined as someone who has listened to at least 75% of a key message. *C2L (caller-to-listener ratio) defines the % completion listening rate of a message, i.e. the proportion of callers who listen to the message all the way to the end. BMI=body mass index, FP=family planning, SRH=sexual and reproductive health

Table 4 shows estimated impact on knowledge. We find a 0.07 SD increase in the primary knowledge outcome score in unadjusted models, and an increase of 0.06 SDs in fully adjusted models. Treatment also increased the average number of reproductive methods women reported to be familiar with by 0.11 points (implying that one in 10 women know one additional method). For nutrition, the treatment reduced the false belief that local herbs could cure anemia by 3 % pts.

Table 4: Impact on Knowledge

Outcome	Reproductive health knowledge z-score	Beliefs that local herbs cure anemia	Number of contraceptive methods familiar with
Treatment effect	0.0708***	-0.0371**	0.116**
(unadjusted)	(0.0265)	(0.0152)	(0.0461)
Adjusted treatment effect	0.0591**	-0.0328**	0.0941**
	(0.0252)	(0.0148)	(0.0455)
Control group average	9.742	0.406	10.33
Survey modality	In person	In person	Phone
Observations	4,877	4,877	5,233

Notes: Table shows estimated impact on primary outcomes. Coefficients displayed are mean differences with standard errors in parentheses. Adjusted models control for age group (age < 20, age 20-34 (ref), age >=35), educational attainment, district, baseline family planning knowledge, baseline hemoglobin level and baseline body mass index. Numbers in parentheses are cluster-robust standard errors. Asterisks indicate p-value range of estimated differences: *p-value<0.10, **p-value<0.05, ***p-value<0.01

Fig 3 shows item-specific changes in sexual and reproductive health knowledge. Largest improvements were observed for myths regarding male condoms causing infertility and post-sex urination preventing pregnancy; small improvements were seen for most questions.

[Figure 3 about here]

Supplemental Materials Figure SF1 further illustrates the observed changes in knowledge for the item with the largest absolute change in knowledge score. Supplemental Materials Table ST1 shows the proportion of women answering each item correctly at baseline and endline by treatment status for all items investigated.

Table 5 summarizes the estimated impact on sexual behavior as reported in person as well as on the phone. The two survey modalities yielded consistent results with 40% of women using

modern contraception and the intervention resulting in an insignificant additional 2% of women using modern contraception. Positive impacts were found for the number of methods ever used (+0.14 methods in adjusted models) and the intention to use modern contraception in the future (+ 5% pts).

Table 5: Impact on self-reported sexual behavior

Outcome	Currently uses any contraception[a)]	Currently uses modern contraception[a)]	Can show contraception to interviewer	Number of methods ever used	Currently uses any contraception[a)]	Currently uses modern contraception[a)]	Plans to use modern contraception in the future
Treatment effect	0.0162	0.0193	0.00320	0.164***	0.0189	0.0218	0.050***
(unadjusted)	(0.0147)	(0.0137)	(0.00667)	(0.0449)	(0.0143)	(0.0138)	(0.0124)
Adjusted treatment effect	0.0157	0.0198	0.00384	0.140***	0.0162	0.0192	0.0468***
	(0.0145)	(0.0134)	(0.00638)	(0.0410)	(0.0138)	(0.0133)	(0.0118)
Control group average	0.455	0.396	0.0718	2.956	0.474	0.396	0.396
Survey modality	In person	In person	In person	Phone	Phone	Phone	Phone
Observations	4,877	4,877	4,877	5,233	5,233	5,233	5,233

Notes: Coefficients displayed are mean differences with standard errors in parentheses. Adjusted models control for age group (age < 20, age 20-34 (ref), age >=35), educational attainment, district, baseline family planning knowledge, baseline hemoglobin level and baseline body mass index. Numbers in parentheses are cluster-robust standard errors. Asterisks indicate p-value range of estimated differences: *p-value<0.10, **p-value<0.05, ***p-value<0.01. a) The full list of contraceptives is shown in Supplemental Materials S2. Contraceptives not considered as “modern” are beads/calendar method, withdrawal and breastfeeding.

Fig 4 provides further details on current use of contraceptive methods with a focus on methods ever used. The intervention increased the use of implants, pills and condoms by 3-4 % pts – changes on other methods were not significant.

[Figure 4 about here]

Table 6 shows estimated impact on prenatal care. A total of 398 current pregnancies, as well as 653 pregnancies completed between baseline and endline were analyzed. No differences were found in antenatal care attendance or deworming. Treatment increased the likelihood of taking iron in current pregnancies by 8% pts and the likelihood of taking supplements in recently completed pregnancies by 4% pts [0.3,7.7].

Table 6: Impact on Prenatal Care

Outcome	Attended antenatal care	Currently taking iron	Deworming	Antenatal care for recent pregnancy	Recent pregnancy >=4 visits	Recent pregnancy: iron	Recent pregnancy: deworming
Treatment effect	0.0593	0.0845*	0.0167	0.00955	0.0229	0.0361*	0.0374
(unadjusted)	(0.0449)	(0.0431)	(0.0459)	(0.0161)	(0.0414)	(0.0189)	(0.0314)
Adjusted treatment effect	0.0453	0.0758*	0.00757	0.0156	0.0209	0.040**	0.0369
	(0.0468)	(0.0456)	(0.0483)	(0.0158)	(0.0412)	(0.0189)	(0.0310)
Control group average	0.621	0.605	0.451	0.958	0.454	0.901	0.808
Sample	Women currently pregnant			Women completing pregnancy last year			
Survey modality	In person						
Observations	398	398	398	653	619	653	647

Notes: Coefficients displayed are mean differences with standard errors in parentheses. Adjusted models control for age group (age < 20, age 20-34 (ref), age >=35), educational attainment, district, baseline family planning knowledge, baseline hemoglobin level and baseline body mass index. Numbers in parentheses are cluster-robust standard errors. Asterisks indicate p-value range of estimated differences: *p-value<0.10, **p-value<0.05, ***p-value<0.01

Table 7 shows estimated impact on other behavior. The intervention increased women's efforts to eat healthy by 4% pts; interestingly, these efforts were generally not linked to losing weight; on average, treated women were 2.5% less likely to attempt for weight loss, and 3% pts more likely to try to gain weight. In terms of food intake, the treatment appears to have increased the

frequency of fruit and vegetable consumption, consistent with the results on healthier diets. This aligns with consistent messaging on increasing healthy habits such as regular activity, the inclusion of nutritious and iron rich foods, and maintaining a healthy body weight.

Table 7: Impact on Other Health Behaviors

Outcome	Aims for health diet	Tried to lose weight	Tried to gain weight	Times at fruit	Times ate vegetables	Times ate protein	Currently taking iron supplements	Exercised
Treated	0.0407***	-0.025**	0.0355***	0.0686**	0.0484**	-0.00060	0.0156	0.0154
(Unadjusted)	(0.0144)	(0.0108)	(0.0128)	(0.0346)	(0.0245)	(0.0183)	(0.00988)	(0.0124)
Adjusted treatment effect	0.0312**	-0.025**	0.0316**	0.0583*	0.0371*	0.00202	0.0174*	0.0119
	(0.0123)	(0.0106)	(0.0126)	(0.0298)	(0.0213)	(0.0172)	(0.00965)	(0.0118)
Control group average	0.552	0.255	0.242	1.335	1.332	1.760	0.136	0.335
Survey modality	Phone survey							
Observations	5,233	5,233	5,233	5,233	5,232	5,233	5,233	5,233

Notes: Coefficients displayed are mean differences with standard errors in parentheses. Adjusted models control for age group (age < 20, age 20-34 (ref), age >=35), educational attainment, district, baseline family planning knowledge, baseline hemoglobin level and baseline body mass index. Numbers in parentheses are cluster-robust standard errors. Asterisks indicate p-value range of estimated differences: *p-value<0.10, **p-value<0.05, ***p-value<0.01

Table 8 shows estimated impacts on pregnancy and birth outcomes. 41% of currently pregnant women in the control group reported that the current pregnancy was not planned; this proportion was 5% pts smaller in the treatment group, but not significantly different. Unplanned pregnancies in the past were slightly more common among treated women (but not statistically different). The proportion of previous pregnancies lost as well as the proportion of most recent births born with low birthweight (< 2500 grams) were substantially lower in the treated group – these differences were however not significant statistically due to the small number of observations here.

Table 8: Impact on pregnancy and birth outcomes

Outcome	Pregnancy was unplanned	Birth spacing < 2 years	Previous pregnancy unplanned	Previous pregnancy low birthweight	Previous pregnancy lost
Treatment effect	-0.0605	0.0519	0.0260	-0.0143	-0.0301
(unadjusted)	(0.0481)	(0.0546)	(0.0341)	(0.0246)	(0.0216)
Adjusted treatment effect	-0.0531	0.0496	0.0215	-0.0157	-0.0305
	(0.0495)	(0.0516)	(0.0337)	(0.0245)	(0.0209)
Control group average	0.410	0.372	0.261	0.0888	0.0384
Survey modality	In person				
Sample	Currently pregnant		Completed pregnancy recently		
Observations	398	371	653	565	653

Notes: Coefficients displayed are mean differences with standard errors in parentheses. Adjusted models control for age group (age < 20, age 20-34 (ref), age >=35), educational attainment, district, baseline family planning knowledge, baseline hemoglobin level and baseline body mass index. Numbers in parentheses are cluster-robust standard errors. Asterisks indicate p-value range of estimated differences: *p-value<0.10, **p-value<0.05, ***p-value<0.01.

Table 9 shows estimated impact on biomarkers. While we see some lower rates of anemia among treated women who currently are, or recently were pregnant (Table 9, column 2), these differences are not statistically significant. No changes were found for BMI overall, or any of the categories analyzed (underweight, overweight, obese).

Table 9: Impact on biomarkers

Outcome	Anemic (Hg<12 g/dL)	Anemic (Hg<12 g/dL)	Body mass index	Overweight (BMI >=25, <30)	Obese (BMI >=30)	Underweight (BMI <18.5)

Treatment effect	0.00745	-0.0495	0.183	0.00277	0.0150	0.0120
(unadjusted)	(0.0129)	(0.0315)	(0.165)	(0.0126)	(0.0106)	(0.00826)
Adjusted treatment effect	0.0111	-0.0468	0.0922	0.000613	0.0102	0.0140*
	(0.0127)	(0.0311)	(0.111)	(0.0121)	(0.00831)	(0.00808)
Control group average	0.23	0.23	24.89	0.26	0.15	0.08
Sample	All	Current or recent pregnancy	All	All	All	All
Observations	4,664	992	4,852	4,852	4,852	4,852

Notes: Coefficients displayed are mean differences with standard errors in parentheses. Adjusted models control for age group (age < 20, age 20-34 (ref), age >=35), educational attainment, district, baseline family planning knowledge, baseline hemoglobin level and baseline body mass index. Numbers in parentheses are cluster-robust standard errors. Asterisks indicate p-value range of estimated differences: *p-value<0.10, **p-value<0.05, ***p-value<0.01.

Table 10 shows the result of the per protocol analysis, where we compare control women who had never called the platform to treated women who had called the platform at least five times.. As expected, estimated impacts on knowledge scores are higher among compliers; however, these estimates become substantially smaller again when we adjust for women's differences in education and reproductive health knowledge at baseline.

Table 10: Per protocol analysis

Outcome	SRH Knowledge z-score	Currently uses modern method	Uses iron supplements	Used iron supplements	Lost pregnancy	Anemic	Anemic post pregnancy
Treatment effect	0.127***	0.0409**	0.0571	0.000959	-0.00654	0.00586	-0.0496
(Unadjusted)	(0.0332)	(0.0159)	(0.0515)	(0.0102)	(0.00671)	(0.0147)	(0.0345)

Adjusted treatment	0.0650**	0.0206	0.0575	0.0117	-0.00911	0.0168	0.0168
effect	(0.0308)	(0.0155)	(0.0523)	(0.00954)	(0.00668)	(0.0144)	(0.0144)
Control group average	-0.0613	0.374	0.643	0.915	0.0386	0.229	0.389
Sample	All	All	Pregnant now	Pregnant last year		All	Pregnant
Restriction	Compliers only						
Observations	3,561	3,561	292	3,313	3,313	3,400	727

Notes:*Only women in the control group who never called the service, and women in the treatment group who called the service at least five times are included in this table. Coefficients displayed are mean differences with standard errors in parentheses. Adjusted models control for age group (age < 20, age 20-34 (ref), age >=35), educational attainment, district, baseline family planning knowledge, baseline hemoglobin level and baseline body mass index. Numbers in parentheses are cluster-robust standard errors. Asterisks indicate p-value range of estimated differences: *p-value<0.10, **p-value<0.05, ***p-value<0.01. SRH=sexual and reproductive health.

Stratified Results

Table 11 shows stratified impact results by age and educational attainment group, as well as by district. For age, we find the weakest effects among women under age 20, and largest impacts among women 35 years and older. Educational level seemed to have little impact, with only slightly larger effects for women with secondary or higher education compared to women with less education. At the district level, impacts vary substantially, with the largest treatment effects in Madi-Okollo (the 0.12 SD increase here is almost twice as large as the effect we see in the sample overall), and the smallest impacts in Rwampara (0.02 SD).

Table 11: Stratified results

Stratum	Age <20	Age 20 - 34	Age 35+	Secondary or higher education	Primary education	Kampala	Katakwi	Madi-Okollo	Rwampara
Treatment effect	0.0213	0.0674*	0.0901*	0.0723 *	0.0587*	0.0949* *	0.0828*	0.122*	0.0221
	(0.127)	(0.0344)	(0.0500)	(0.0430)	(0.0331)	(0.0431)	(0.0455)	(0.0654)	(0.0517)
Observations	291	2,978	1,608	1,804	3,073	1,472	1,093	650	1,662

Notes: Coefficients displayed are mean differences with standard errors in parentheses. Adjusted models control for age group (age < 20, age 20-34 (ref), age >=35), educational attainment, district, baseline family planning knowledge, baseline hemoglobin level and baseline body mass index. Numbers in parentheses are cluster-robust standard errors. Asterisks indicate p-value range of estimated differences: *p-value<0.10, **p-value<0.05, ***p-value<0.01.

Harms statement

No harm was reported during the trial.

Discussion

The main objective of this study was to assess the impact of Viamo's call-in platform on health knowledge, behaviors and outcomes. Conceptually, knowledge platforms could result in changes on three distinct levels: changes in health knowledge, changes in health behavior, and changes in health outcomes. The results presented here suggest that changes in knowledge definitely occurred, with treated women displaying improved sex and reproductive health knowledge, being better informed about anemia treatment and more familiar with a larger range of contraceptive methods. This increased familiarity with contraceptive knowledge was also partially visible in contraceptive use. Treated women were on average only marginally (and insignificantly) more likely to use modern contraception, but used a different mix of contraceptives, with higher usage of implants, and somewhat reduced reliance on injectables and calendar methods. In terms of future plans, the intervention seems to have substantially increased the proportion of women who say they will use modern contraception in the future.

For anemia - one of the key health challenges addressed on the platform - the treatment seems to have resulted in an increased uptake of supplements during pregnancy; these higher rates of supplementation also seem consistent with marginally lower rates of anemia among recently pregnant women. The estimated differences in anemia in this subgroup are however not statistically significant, which may partially be due to the relatively small number of women. Despite the high rates of anemia among non-pregnant women, we did not see any change in general protein intake or consumption of iron supplements. Given this lacking change (and the small proportion of women benefiting from additional supplementation during pregnancy), the insignificant impact on general hemoglobin levels makes sense.

We also see some interesting declines in the rates of pregnancy loss and the prevalence of low-birth-weight births in this subsample, but did not have sufficient statistical power here due to the relatively small number of women completing a pregnancy within the study period.

Given that a larger proportion of women in the treatment group claimed to aim for a healthy diet and to eat more fruit and vegetables, nutrition related content clearly reached its target. Interestingly, the messages received around “healthy body shapes” do not seem to have triggered a major shift towards eating less and lower BMIs, but seems to have induced a desire to change in both directions, i.e. we see an increasing proportion of women who want to lose weight and an increasing proportion of women who want to gain weight (and hence a smaller proportion of women who want to stay as they are). These shifts in intentions and behaviors are not seen in the anthropometric data - the intervention neither changed average BMI nor the proportions of women classified as underweight, overweight or obese.

In terms of the primary outcome, our results seem consistent with a recent review of mHealth intervention, which suggests that digital information sources can improve sexual and reproductive health knowledge as well as use of sexual and reproductive health services [16]. Compared to the estimates reported for an interactive intervention in Ghana [11, 17] the effect sizes observed here seem smaller, which seems plausible given that not all women engaged with the service on a regular service. Compared to previous studies conducted in other parts of Africa we also observed rather high sexual and reproductive health knowledge at baseline – most women were for example familiar with a range of contraceptive methods (beyond traditional methods, condoms and pills), and myths regarding side effects of contraception were less common that seen in other settings [11, 17] – this likely also contributes to the somewhat smaller knowledge impacts seen here.

In terms of policy, one of the main advantages of the platform used is its relatively low cost – particularly when run at large scale; most of the costs of content development are related to the initial set-up, while operating costs are relatively minor. More research is needed here to investigate the cost-effectiveness of these new mobile technology channels as tools to improve health knowledge among vulnerable populations relative to traditional channels such as radio, tv or community outreach activities.

Despite the very large, stratified and regionally representative sample, the study has some limitations. First, there were not as many pregnant women as anticipated, reducing power on pregnancy-related behaviors and outcomes to some extent. Second, we found that the proportion of women using Airtel phones was substantially lower in some rural districts, making it very difficult to reach enrollment targets in some communities. We compensated this by enrolling more women in clusters with higher coverage. Even though a more selected

sample clearly does not undermine the internal validity of the study (since randomization was done at the individual level), it is not clear whether the final set of women enrolled in the study is fully representative of the larger populations in the four study districts; it is also not clear if the four districts are fully representative of the country as a whole. Since the service is currently only available to Airtel users, this study simply focused on women with access to this service. Even though it seems plausible that similar effects could be found with other phone providers, it is not clear if the results of this study can be generalized to the country as a whole or to other countries. Third, many of the behaviors measured were self-reported; while we implemented verification measures by taking pictures of antenatal and child health cards and asking women for evidence of contraceptive packaging, such direct evidence was generally only available for a small minority of women, and could thus not be used in the analysis. It is clearly possible that access to the service changes norms and reporting bias, which could explain some of the behavioral changes reported and detected in our analysis. The primary outcome of the study - the reproductive knowledge score - should not be subject to any reporting bias as all women should have the incentive to give the correct answer on the true/false knowledge quiz. Fourth, the individual-level randomization made the study vulnerable to spillover effects. Qualitative feedback collected at the end of the study suggests that treated women both invited other community members – spouses, friends and other family members – to use the service and actively shared new information with them. Given that some of this shared information likely reached the control group, it seems likely that the impact reported here underestimate the true causal effect of the program. We controlled for direct service use by excluding active users in the per protocol analysis (Table 10) – this does however not capture direct sharing of knowledge among community members.

Conclusions

Overall, the results presented here suggest that the platform can effectively improve health knowledge and to some extent also health behaviors among vulnerable populations. These changes in knowledge, awareness and intentions could plausibly also induce changes in health outcomes in the long run; longer-term studies will likely be needed to accurately measure such effects. Future research should also aim to quantify knowledge spillovers within communities

as well as the cost-effectiveness of call-in platform like the one analyzed here relative to other communication channels such as TV, radio, posters or community-based activities.

Acknowledgements: We would like to thank all involved staff members at IPA Uganda as well as in the Viamo team for their efforts and continued support of the project. We are also grateful to the more than 6000 study participants for their willingness to participate in the study. Lastly, we would like to thank the USAID Development Innovation Ventures (DIV) team for their input and feedback throughout the project.

References

1. World Bank, *Individuals using the Internet (% of population) | Data.* 2025.
2. Sarikhani, Y., S.M. Najibi, and Z. Razavi, *Key barriers to the provision and utilization of maternal health services in low-and lower-middle-income countries; a scoping review.* BMC Womens Health, 2024. **24**(1): p. 325.
3. D'Souza, P., et al., *Factors influencing contraception choice and use globally: a synthesis of systematic reviews.* The European Journal of Contraception & Reproductive Health Care: The Official Journal of the European Society of Contraception, 2022. **27**(5): p. 364-372.
4. Conde-Agudelo, A. and J.M. Belizán, *Maternal morbidity and mortality associated with interpregnancy interval: cross sectional study.* Bmj, 2000. **321**(7271): p. 1255-9.
5. Doku, D.T. and S. Neupane, *Survival analysis of the association between antenatal care attendance and neonatal mortality in 57 low- and middle-income countries.* Int J Epidemiol, 2017. **46**(5): p. 1668-1677.
6. Ota, E., et al., *Antenatal interventions for preventing stillbirth, fetal loss and perinatal death: an overview of Cochrane systematic reviews.* Cochrane Database Syst Rev, 2020. **12**(12): p. Cd009599.
7. Noar, S.M., *A 10-year retrospective of research in health mass media campaigns: where do we go from here?* Journal of Health Communication, 2006. **11**(1): p. 21-42.
8. Wakefield, M.A., B. Loken, and R.C. Hornik, *Use of mass media campaigns to change health behaviour.* Lancet, 2010. **376**(9748): p. 1261-1271.
9. Stead, M., et al., *Mass media to communicate public health messages in six health topic areas: a systematic review and other reviews of the evidence*. Public Health Research. 2019, Southampton (UK): NIHR Journals Library.
10. International Telecommunication Union *Facts and Figures 2024 – Mobile network coverage*. ITU Digital Reports, 2024.
11. Rokicki, S., et al., *Impact of a Text-Messaging Program on Adolescent Reproductive Health: A Cluster–Randomized Trial in Ghana.* American Journal of Public Health, 2017. **107**(2): p. 298-305.
12. Unger, J.A., et al., *Short message service communication improves exclusive breastfeeding and early postpartum contraception in a low- to middle-income country setting: a randomised trial.* BJOG: an international journal of obstetrics and gynaecology, 2018. **125**(12): p. 1620-1629.
13. Harrington, E.K., et al., *An mHealth SMS intervention on Postpartum Contraceptive Use Among Women and Couples in Kenya: A Randomized Controlled Trial.* American Journal of Public Health, 2019. **109**(6): p. 934-941.
14. UNDP. *Digital X Solution Catalogue*. 2025 10 Sep 2025].
15. Huber, P.J., *The behavior of maximum likelihood estimates under nonstandard conditions. Proceedings of the Fifth Berkeley Symposium on Mathematical Statistics and Probability. Berkeley, CA 1, 221–233.* 1967.
16. Onukwugha, F.I., et al., *The effectiveness and characteristics of mHealth interventions to increase adolescent's use of Sexual and Reproductive Health services in Sub-Saharan Africa: A systematic review.* PLOS ONE, 2022. **17**(1): p. e0261973.
17. Rokicki, S. and G. Fink, *Assessing the reach and effectiveness of mHealth: evidence from a reproductive health program for adolescent girls in Ghana.* BMC Public Health, 2017. **17**(1): p. 969.

Figure Legends

Figure 1: Spatial distribution of study districts (orange shapes) and study clusters (small circles). Uganda administrative shapefiles provided by https://gadm.org/data.html. Figure created by authors.

Figure 2: Consort Trial participant flow chart.
Figure 2 Notes: [1] Ateso, English, Luganda, Lugbara, Luo or Runyakitara; [2] Number out of service, phone being switched off, or calls unanswered. [3] Household visited at baseline was not found again. [4] Ill, intoxicated, or deceased

Figure 3: Item-level change in knowledge

Notes: each point/square on the graphic represents the estimated coefficient obtained from regressing a binary indicator of giving a correct answer to the specific question on treatment - lines illustrate 95% confidence intervals.

Figure 4: Estimated impact on contraceptives ever used

Figure notes: each point/square on the graphic represents the estimated coefficient obtained from regressing a binary indicator of having ever used the method in question on treatment - lines illustrate 95% confidence intervals.

Figure 1

Click here to access/download;Figure;Fig1.jpg

~~You need to add the description of the Figure.~~

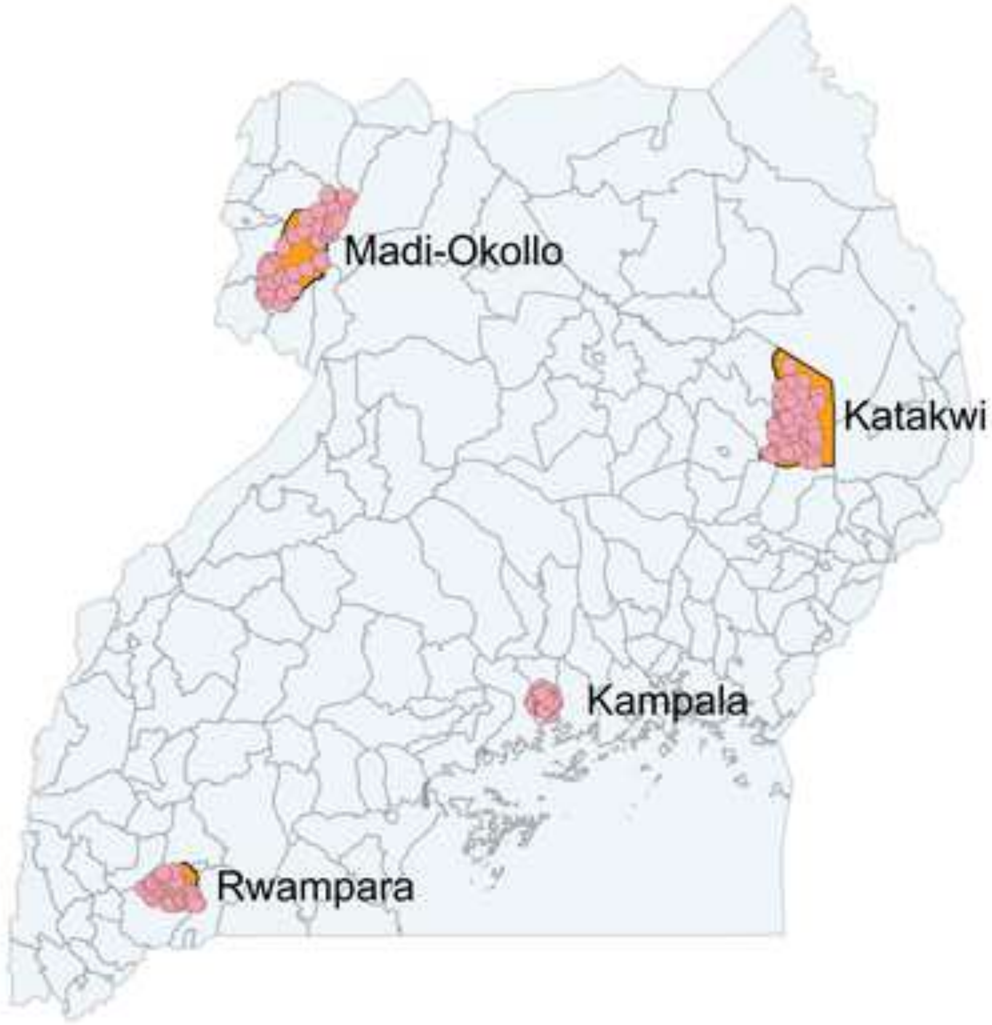

Figure 2

Click here to access/download;Figure;Fig2.png

~~Add the description of Figure 2.~~

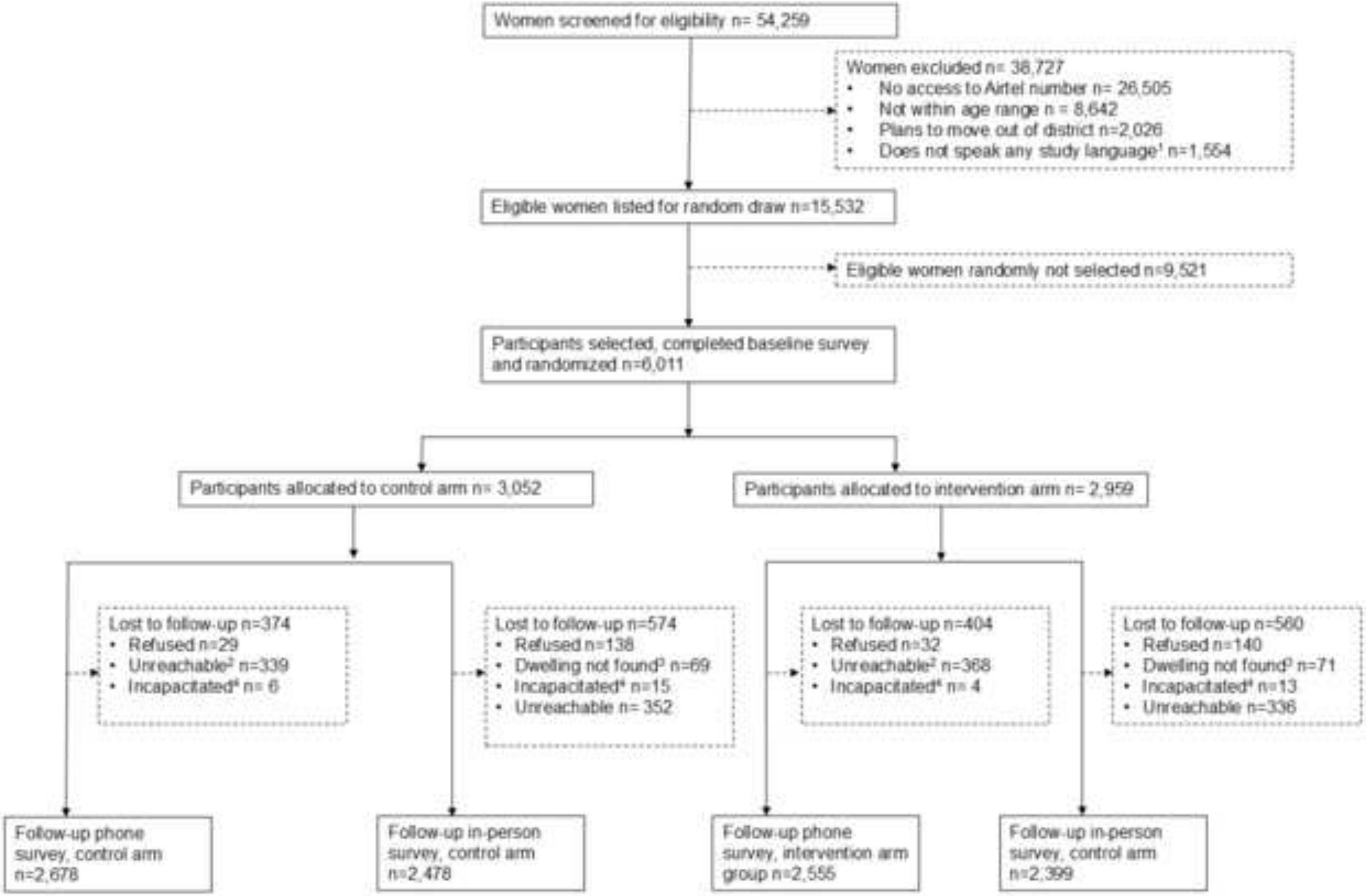

Figure 3

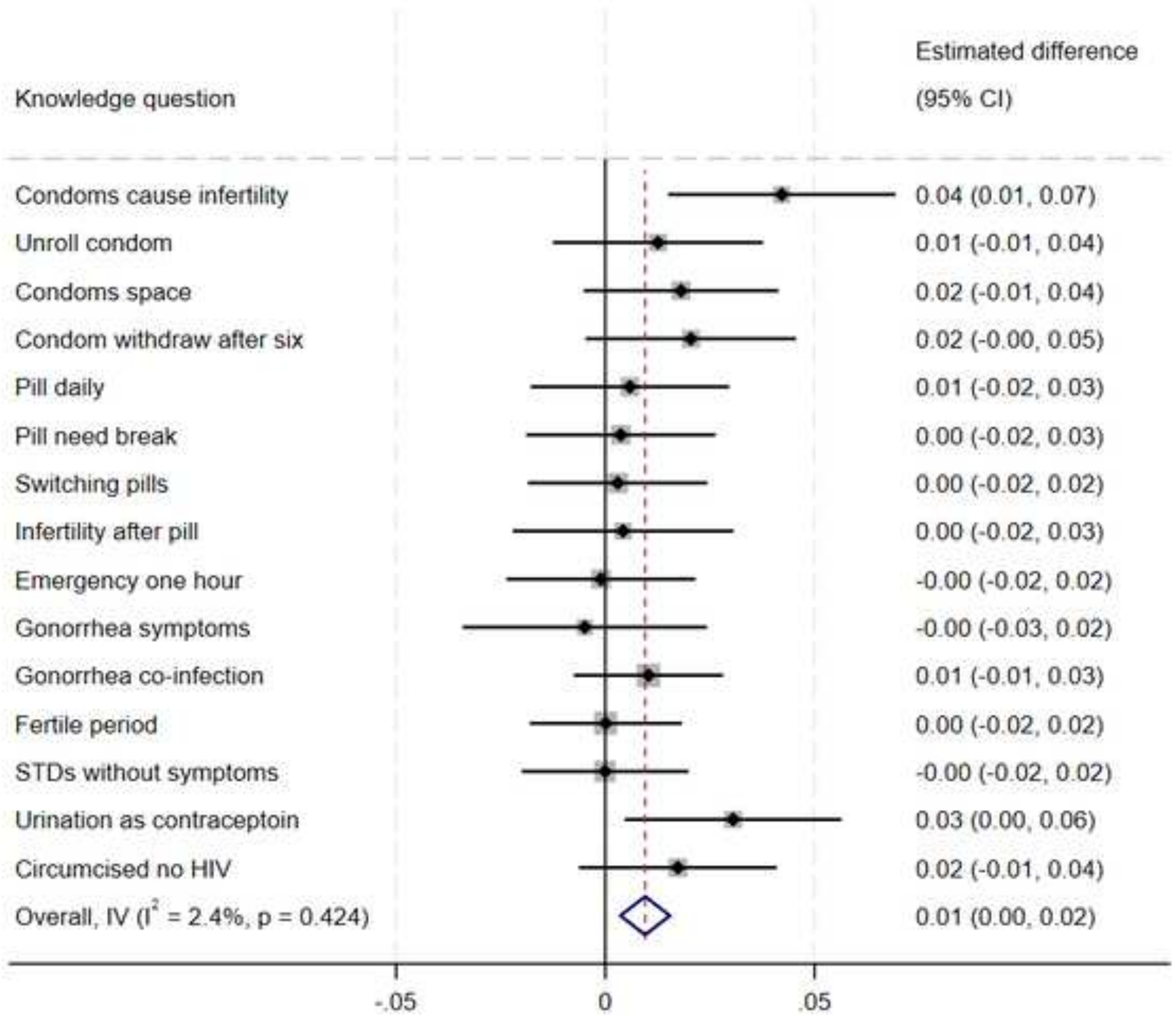
Knowledge question
Estimated difference (95% CI)
Condoms cause infertility
0.04 (0.01, 0.07)
Unroll condom
0.01 (-0.01, 0.04)
Condoms space
0.02 (-0.01, 0.04)
Condom withdraw after six
0.02 (-0.00, 0.05)
Pill daily
0.01 (-0.02, 0.03)
Pill need break
0.00 (-0.02, 0.03)
Switching pills
0.00 (-0.02, 0.02)
Infertility after pill
0.00 (-0.02, 0.03)
Emergency one hour
-0.00 (-0.02, 0.02)
Gonorrhea symptoms
-0.00 (-0.03, 0.02)
Gonorrhea co-infection
0.01 (-0.01, 0.03)
Fertile period
0.00 (-0.02, 0.02)
STDs without symptoms
-0.00 (-0.02, 0.02)
Urination as contraceptoin
0.03 (0.00, 0.06)
Circumcised no HIV
0.02 (-0.01, 0.04)
Overall, IV (I² = 2.4%, p = 0.424)
0.01 (0.00, 0.02)
-.05
0
.05

Figure 4

Click here to access/download;Figure;Fig4.png

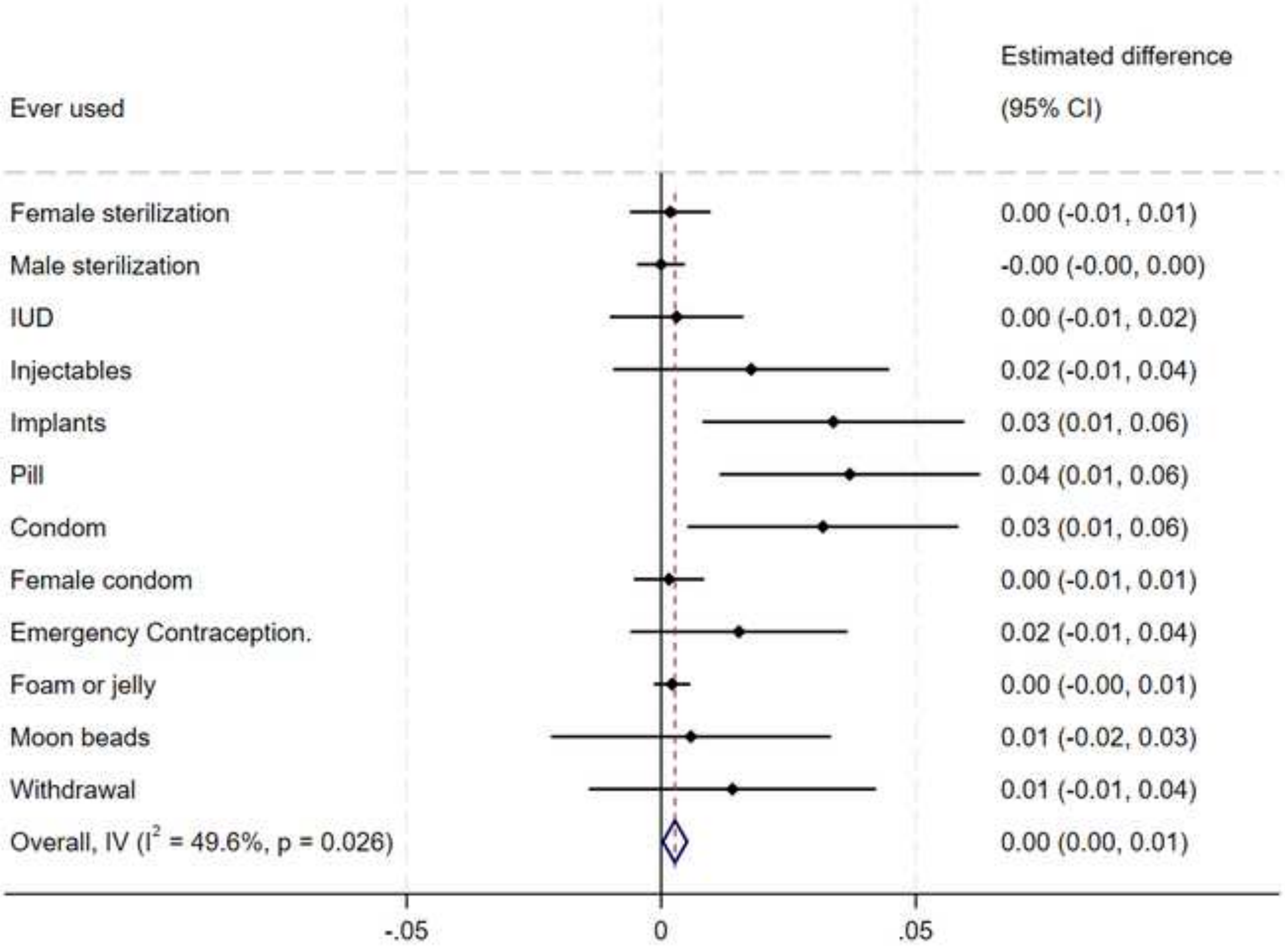

Click here to access/download
Supporting Information
Supplemental Materials 11 Sep 2025.docx

Click here to access/download
Supporting Information
Inclusivity-in-global-research-questionnaire 10 Sep 2025.docx

Replication package

Click here to access/download
Supporting Information - Compressed/ZIP File Archive
Replication package csv.tar

Click here to access/download
Other
Checklist completed 11 Feb 2025.docx

ethics approval Uganda

Click here to access/download
Other
MUREC-2022-124_initial approval.pdf

IRB approval IPA

Click here to access/download

Other

4059_Initial_ApprovalLetter_Expedited.pdf

Click here to access/download
Other
AO_2022-00034_NZ_Ado_pos_220713_en.pdf

Estimating the Impact of Viamo Call-in Information Services on Sexual and Reproductive Health Knowledge, Behavior, and Outcomes among ~~women of reproductive age: Results from a~~Women of Reproductive Age: A 2-Arm Open Label Randomized Controlled Trial in Uganda

Formatted: Left

Günther Fink[1,2*], Branwen Nia Owen[1,2], Jan Will[3], Ilyena Kozain[4]

1 University of Basel, Basel, Switzerland

2 Swiss Tropical and Public Health Institute, Allschwil, Switzerland

3 Innovations for Poverty Action, Kampala, Uganda

4 Viamo, Kampala, Uganda

*** Corresponding author:** Swiss Tropical and Public Health Institute, Kreuzstrasse 2, 4123 Allschwil, Switzerland. Email: guenther.fink@swisstph.ch.

Abstract

Background. Despite substantial increases in access to schooling and to the internet, health information and knowledge remains limited among ~~many vulnerable populations.~~women of reproductive health in many low and middle income countries. Given the near universal cell-phone coverage in low- and middle-income countries, call-in services offer an interesting new platform to provide high quality information to targeted populations. In this paper, we assessed the impact of the Viamo 3-2-1 call-in Platform on health knowledge and behavior among women of reproductive age in Uganda.

Methods. We conducted a 2-arm open label randomized controlled trial involving 6,000 randomly selected women, ages 18-49 years, with access to a simple feature Airtel cell phone in Uganda. Fifty enumeration areas were randomly selected in Kampala, Madi-Okollo, Rwampara and Katakwi districts. Consenting women were asked to complete a baseline and endline survey implemented by trained interviewers using tablets through home visits. After completion of baseline, women were randomized with equal probability to treatment and control using a tablet-generated random number draw. Study staff introduced treated women to the call-in service and encouraged its use through various incentives and promotional messages. Primary outcomes were sexual and reproductive health knowledge scores as well as iron supplementation in pregnancy. Secondary outcomes included self-reported use of contraception, healthy diets, birth spacing and birth outcomes as well as hemoglobin levels and BMI. ~~Intention to treat as well as per protocol estimates~~Linear regression models were used to ~~assess program impact~~estimate mean differences in outcomes between treatment and control. Standard errors were corrected for the cluster-based sampling of women.

Results. A total of 6,011 women were enrolled into the study between December 6, 2022 and February 11, 2023; 3,052 were assigned to treatment, 2,959 used as controls. Endline data was collected between May 8, 2024 and August 4, 2024. Overall follow-up rates were 92% in both the treatment and the control group. Treatment increased average sexual and reproductive health knowledge by 0.07 standard deviations (95% CI [0.01, 0.11], p-value = 0.02) and

increased the likelihood of taking iron supplements by 4% pts among recently pregnant women ([0.003, 0.08], p-value 0.04). Results for secondary outcomes were mixed, with positive changes for nutritional intake and family planning behaviors and intentions and no impact on hemoglobin levels and BMI. No adverse events or outcomes were reported.

Conclusions. The results of this trial suggest that access to call-in services can increase health knowledge and to some extent also health behaviors. Longer and more intensive program exposure may be needed to yield measurable changes in reproductive outcomes and nutritional status.

Keywords: reproductive health, call-in-services, health knowledge, Uganda, Viamo.

Introduction

In low- and middle-income countries (LMIC), access to timely and accurate information in national languages is often limited, particularly among the poor. Whereas the internet is a common source of information in many countries, only 10% of Ugandans, and 36% of Sub-Saharan Africans were estimated to use the internet in 2021; far lower than the global average of 63% [1]. This lack of information access can contribute to poor health decisions, such as inadequate prenatal care and ineffective family planning methods [2]This lack of information access can contribute to poor health decisions; this is particularly important for women of reproductive age, since lack of information often leads to inadequate prenatal care [2] and ineffective family planning methods [3], threatening not only the life of mothers [4] but also their children [5, 6].

Public health messaging in LMICs is often disseminated through mass media channels such as radio messages and billboards. These channels have proven to be effective at reaching large numbers of people [3][7] and increasing knowledge, but have had an insufficient impact on changing behaviors in many areas of public health [4, 58, 9]. Mass media campaigns often fail to create behavior change because, in part, people cannot access the information at a moment of need. Cell phones, abundant in Uganda [6], offer

The rapid growth in cell phone coverage in recent years [10] offers new opportunities to address this gap, as information can be accessed at any time, from anywhere. Recent evidence suggests that interventions to improve reproductive health knowledge through easy access technologies can effectively improve reproductive health knowledge and use of modern contraception [7-911-13].

Viamo's platform was created with the objective of addressing the need for accessible and timely health information. Through an easy to remember dial-in number, callers can obtain up-to-date news as well as key information on topics related to nutrition, maternal and child health, and sexual and reproductive health. The platform is free of charge, and currently has over 4 million users in Uganda, and 35 million users in LMICs globally [10]. With the aim of rigorously assessing the impact of the Viamo's call-in service on sexual and reproductive health knowledge and related behavior, we conducted a randomized controlled trial (RCT) with women of reproductive age across four districts of Uganda. Our primary hypothesis was that access to the platform can improve sexual and reproductive health knowledge and attitudes.

In this study, we aimed to assess whether providing access to call-in-services – free numbers that women can dial to obtain information on health – can effectively improve sexual and reproductive health knowledge and attitudes among women of reproductive health in Uganda.

Methods

Study Design

This study was designed as a two-arm, non-blinded, individual-level randomised controlled trial focusing on women's nutrition and reproductive health. Given that the main intervention – a phone-based information service – was equally available to both arms, we used an encouragement design, actively introducing women in the treated arm to the service and encouraging them to use it throughout the study period. Blinding of the intervention was not possible given the nature of the program.

Study Setting and Participant Recruitment

This study was conducted in four districts of Uganda: Kampala, Katakwi, Madi-Okollo and Rwampara. These four districts were purposely chosen to cover all major regions of Uganda. To recruit women within these four districts, a ~~two~~multi-stage ~~random~~ sampling process was followed: first, a list of all enumeration areas in each district was obtained, and 50 enumeration areas were randomly chosen using a simple random number draw created with the Stata statistical software package. In rural areas, enumeration areas corresponded to villages, and in urban areas, to residential blocks. All households within each selected enumeration area were visited to identify eligible women. Fig 1 shows the spatial location of the study districts within Uganda.

Inclusion and exclusion criteria

~~Study~~In a second step, study eligibility was assessed by trained study enumerators. Women living in selected enumeration areas were eligible for inclusion in the study if they were aged 18-49 years and had access to an Airtel (a mobile network operator) phone number within their household. Given the explicit focus on antenatal care, family planning and post-partum, all women who were pregnant during the listing were automatically invited to join the study; non-pregnant women were randomly selected for the study until the 45 women per cluster target

was reached. In clusters with less than 45 women, all women were invited to participate in the study.

Women were ineligible if they had used the Viamo platform previously, they planned to move outside of the study area within 12 months of baseline or if they did not speak one of the six languages available on the platform: Ateso, English, Luganda, Lugbara, Luo or Runyakitara.

There were no exclusion criteria (or exclusions) during the study.

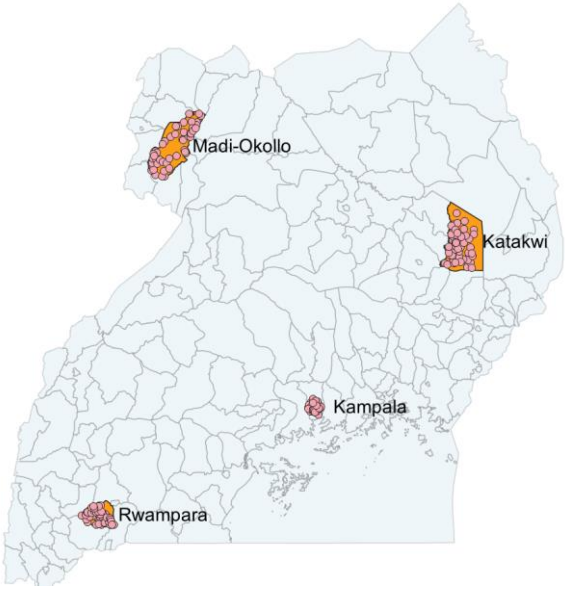

Formatted Table

Figure 1: Spatial distribution of study districts (orange shapes) and study clusters (small circles). Uganda administrative shapefiles provided by https://gadm.org/data.html. Figure created by authors.

Intervention

Viamo's platform was created with the objective of addressing the need for accessible and timely health information. Through an easy to remember dial-in number, callers can obtain up-to-date news as well as key information on topics related to nutrition, maternal and child health, and sexual and reproductive health. The platform is free of charge, and currently has over 4

million users in Uganda, and 35 million users in LMICs globally [14]. Users who call hear a pre-recorded menu, and can choose topics to listen to from these menus.

In Uganda, the Viamo platform is available via voice and text channels on all mobile phones with an Airtel number and offers a wide variety of health and other information to all interested callers. To

For the study, we aimed to generate random variation in system usage by providing strong encouragement to use the system to a randomly selected subset of women. Specifically, to ensure increased service utilization among women randomly selected for the treatment, all treated women in the sample received:

1. A personalized introduction and mock call into platform (at time of baseline interview)
2. A promotional calendar to serve as a physical reminder on how to navigate the service and the benefits of the service (at time of baseline interview)
3. Continued encouragement to use the service in the form of text messages and outbound calls, incentivizing women to call the service.

The original sixty sexual and reproductive, maternal and neonatal health and nutrition key messages launched at the inception of the project were developed in conjunction with subject experts, end users, communication specialists, and the Ugandan Ministry of Health (MOH). Throughout the implementation period, the key messages (2-4 mins of dialogue) were transformed into stylized dynamic content (7-45 mins), to reiterate critical concepts for enhanced learning and be able to promote new content. The release of DIV-dynamic content formats (dramas, talk shows, and celebrity interviews) was staggered and began in August 2022.

Following enrollment in the study (December 2022 - March 2023), ViamoTreated women were encouraged to use the system whenever they had questions or wanted to know more about sexual and reproductive health. Viamo also employed targeted nudges, personalized campaigns and other outreach activities (such as release of new content), with the aim of maintaining and incentivizing usage rates (active calling into the Viamo Platform) among women in the treatment group (see Supplemental Materials S1 for an example).

Study Procedures

Study staff visited selected women at their homes, introduced the study and invited them to participate. The consent form was read aloud and explained when necessary. Literate women

signed the consent form and illiterate women marked the form with a thumbprint, and a literate witness was asked to sign on their behalf. We did not assess participants' cognitive capacity to provide consent.

Baseline data was collected via face-to-face interview at each participants' home between December 6, 2022 and March 6, 2023. Upon completion of the baseline survey, treated women were introduced to the service by study staff. Endline data was collected approximately 15 months after recruitment, between May 8, 2024 and August 4, 2024 in two separate surveys; a phone survey and a face-to-face in-person survey (see Supplementary Table 1 for dates by district, and Supplementary Table 2 for data collected by phone and in-person interview at endline). During all three surveys, the interviewer recorded responses on a tablet using the SurveyCTO software. Data collectors received training and on-going support and monitoring to ensure data quality. Participants received UGX10,000 (~USD $2.77) of phone credit as a token of gratitude after each survey interview.

Surveys collected data on socio-demographic characteristics, knowledge and use of the Viamo platform, reproductive health knowledge, contraceptive use, pregnancy planning and uptake of antenatal care, pregnancy outcomes, diet and exercise behaviors and select biomarkers.

Randomization

Upon completion of the baseline survey, we randomised arm allocation at the individual level based on a simple number draw generated by the SurveyCTO software. ~~At~~Specifically, at the end of the survey, the software generated a random number from a uniform distribution [0,1] and allocated all draws > 0.5 to treatment.

Women selected for the treatment arm were then provided with a personalized introduction to the service (after the baseline survey was completed) and called the service directly from their own phone with the support of the interviewer to see how the system works. Treated women were also given a promotional calendar to serve as a physical reminder on how to navigate the service and the benefits of the service. Women in the control group were not given any intervention. Given that the service was freely available to all women, they were in theory able to call the service at any time, but not given any encouragement or support to do so by the study team.

Power Calculations

The study was powered to detect a 0.1 standard deviation (SD) difference in standardized knowledge scores (mean 0, SD 1) with power 0.9, assuming. Assuming an average cluster size of 30, a follow-up rate of 90% and a design effect of 1.3. This, this required 100 clusters per arm, and a total sample size of 6000 women. The targeted effect size was based on an anticipated knowledge score difference of 0.2 SD among compliers (system users), and an average compliance of 10% in the control, and 60% in the treatment arm (a net compliance/usage differential of 50%).

Primary and secondary outcomes

The two primary outcomes of the study were reproductive health knowledge and nutritional supplementation in pregnancy. For reproductive health, we used a 15-item questionnaire of true/false statements (items listed in Supplemental Materials S2). This tool was based on previous studies conducted in sub-Saharan Africa [7] and adapted to match local context.[11] and pretested locally to make sure all items were suitable for the study population. To facilitate interpretation of regression results, we converted the raw scores (number of correct answers out of 15) into z-scores with mean zero and standard deviation one. For nutrition knowledge, we analyzed a binary indicator for women (falsely) believing that herbs can cure anemia. For nutritional supplementation during pregnancy, we used self-reported iron supplementation as an outcome measure. Secondary outcomes included contraceptive knowledge (number of methods respondents are familiar with), contraceptive use, (currently using any modern method), prenatal care uptake, (any, number of visits), hemoglobin level (as a biomarker indicating actual iron intake), body mass index, and birth spacing.

For pregnancy related outcomes, we analyzed two separate sets of women: first, women reporting to be pregnant at the time of the endline survey (currently pregnant), and second, women who completed a pregnancy in the year preceding the endline survey (recently pregnant). For current pregnancies, we analyzed self-report iron supplementation as well as self-reported use of antenatal care services, and whether women indicate the pregnancy was planned. For recently completed pregnancies, we analyzed the same variables, but also analyzed pregnancy loss and low birth weight (reported weight at birth < 2.5kgs) as additional secondary outcomes.

Statistical Analysis

We began our analysis with a comparison of baseline characteristics across arms, as well as a comparison of follow-up rates to assess potential selective attrition. All primary models were estimated intent-to-treat, comparing outcomes between treated and control women at endline. For all outcomes, we estimated both unadjusted linear regression models (not including any covariates) and fully adjusted models, where we controlled for age, district, marital status, educational attainment and baseline knowledge variables. The main rationale for using linear regression models was to generate mean differences estimates that could be compared across the rather large set of outcome variables. To adjust for non-normal residuals, we used Huber's cluster-robust standard errors [15].

We used forest plots to illustrate item level changes for aggregated reproductive health knowledge and contraceptive use scores. In an exploratory analysis, we also estimated per protocol models and stratified models. Per protocol models were restricted to compliant women: specifically, we included only women in the control group who had never called the service, and women in the treatment group who had called the service at least five times. To investigate differential impact on subgroups, we estimated stratified models by age group (<30, 30+), educational attainment group (primary or less, secondary, higher) and district.

Study registration and ethical clearance

The trial was registered on June 03, 2022 at https://www.socialscienceregistry.org/trials/9544. Ethical clearance for the study was obtained from the Mildmay Uganda Research Ethics Committee (MUREC-2022-124), the ethics committee for North-Western Switzerland (AO_2022-00034) as well as by the Innovations for Poverty Action internal review board (#00006083).

Results

Of 54,259 women screened, 29% (n=15,532) were eligible to participate in the study (Fig 2). The most common reason for ineligibility was not having access to an Airtel number. A total of 6,011 women completed the baseline survey and were randomised to the control (n=3,052) or the intervention arm (n=2,959). At endline, 87% (n=2,678) of control arm participants, and 86% (n=2,555) of intervention arm participants completed the phone survey. Slightly fewer participants completed the in-person survey (81% of both arms). 92% of participants in both

arms completed at least one of the two modalities (i.e. either a phone or an in-person survey). The most common reason for loss to follow were participants being unreachable by phone, and participants having moved house, in the phone survey and in-person survey, respectively.

Formatted Table

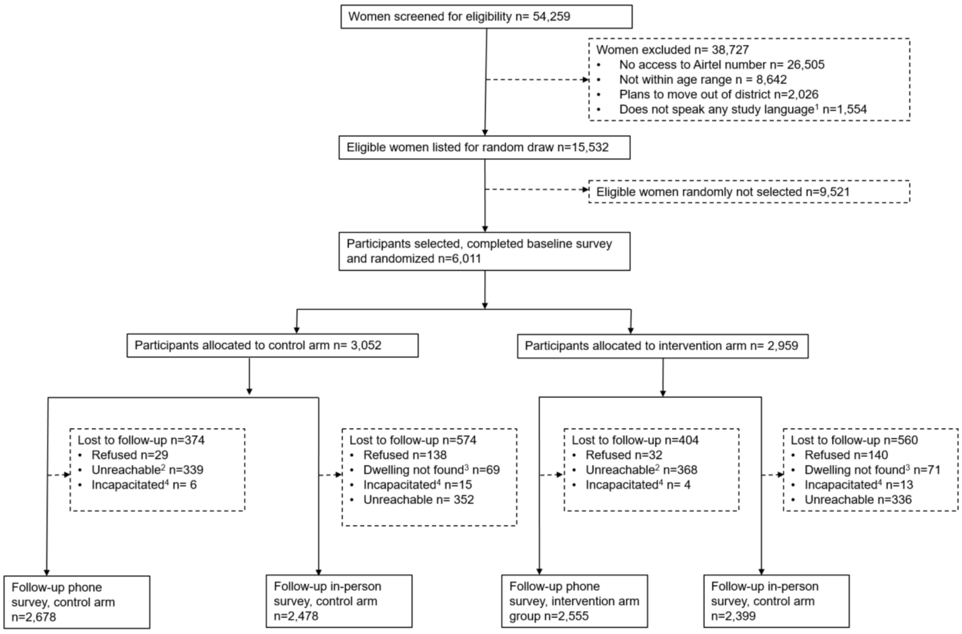

Figure 2: Consort Trial participant flow chart.
Figure 2 Notes: [1] Ateso, English, Luganda, Lugbara, Luo or Runyakitara; [2] Number out of service, phone being switched off, or calls unanswered. [3] Household visited at baseline was not found again. [4] Ill, intoxicated, or deceased

Table 1 shows descriptive statistics for the study sample. Over half were under 30 years old (53%), and a majority were currently married (58%). Twelve percent (12%) of subjects were recruited in Madi-Okollo, 21% in Katakwi, 32% in Rwampara and 35% in Kampala. Fewer than half of women lived in a household with piped water (47%) and very few had health insurance (2%), but most owned their own phone (84%). Most had previously been pregnant (88%) and nearly a seventh were currently pregnant (14%). The trial arms were well-balanced, with all characteristics very similar across arms (Table 1).

Table 1: Sample characteristics overall and by study arm

Characteristic	Overall (n=6,011) N (%)	Control (n=3,052) N (%)	Treatment (n=2,959) N (%)
Age 18-29	3,159 (53%)	1,609 (53%)	1,550 (52%)
Age 30-39	1,913 (32%)	979 (32%)	934 (32%)
Age 40+	939 (16%)	464 (15%)	475 (16%)
Currently married	3,503 (58%)	1,756 (58%)	1,747 (59%)
No education	159 (2.6%)	95 (3.1%)	64 (2.2%)
Primary education	3,173 (53%)	1,640 (54%)	1,533 (52%)
Secondary or higher education	2,611 (43%)	1,285 (42%)	1,326 (45%)
Education: other	68 (1.1%)	32 (1.0%)	36 (1.2%)
Speaks English	1,716 (29%)	862 (28%)	854 (29%)
Kampala	2,081 (35%)	1,071 (35%)	1,010 (34%)
Katakwi district	1,256 (21%)	619 (20%)	637 (22%)
Madi-Okollo district	741 (12%)	385 (13%)	356 (12%)
Rwampara district	1,933 (32%)	977 (32%)	956 (32%)
Household has piped water	2,799 (47%)	1,431 (47%)	1,368 (46%)
Household has health insurance	100 (1.7%)	45 (1.5%)	55 (1.9%)
Woman has her own phone	5,056 (84%)	2,527 (83%)	2,529 (85%)
Currently pregnant	867 (14%)	441 (14%)	426 (14%)
Previously pregnant (ever)	5,275 (88%)	2,683 (88%)	2,592 (88%)

Formatted Table

Table 2 summarizes overall compliance with treatment and platform use. 50% of women in the control group indicated at endline that they had heard of the Viamo platform compared to 96% in the intervention arm. 20% of women in the control arm reported to have ever used the platform, and 7% reported to have used it in the last month. In the treatment arm, the same was true for 84% and 42% respectively. On average, subjects in the control arm reported four calls to the platform, women in the treatment arm reported an additional 18 calls on average. These numbers also align nicely with the Viamo platform metrics, which recorded an average of four calls for the control group, and an additional 31 calls among women in the treatment group (suggesting that on average women under-reported the number of actual calls they made). In a given month, a treatment user listened to an average of approximately 38 minutes of content on the platform

Table 2: Treatment Compliance

Outcome	Heard of the platform	Ever used the platform	Used the platform in the last month	Total number of self-reported calls to platform [a)]	Total number of registered calls to platform
Treatment effect	0.459***	0.642***	0.346***	18.25***	31.41***
	(0.0192)	(0.0143)	(0.0129)	(0.471)	(0.958)

Formatted Table

Control group average	0.499	0.499	0.0709	4.075	4.012
Observations	5,233	5,233	5,233	5,233	5,233
R-squared	0.262	0.412	0.164	0.268	0.271

Notes: All point estimates displayed correspond to estimated mean differences in outcomes. Numbers in parentheses are cluster-robust standard errors. Asterisks indicate p-value range of estimated differences: *p-value<0.10, **p-value<0.05, ***p-value<0.01;. [a)] Self-reported number of calls were top-coded at 51 calls.

Table 3 provides an overview over the most popular content. The most popular single item was the local weather forecast, followed by information on food and general news. On average, 71% of the content listened to by women in the treatment group accessing the platform (n=2,825) was content specifically related to maternal health, sexual and reproductive health, family planning, or nutrition. For the control group members who accessed the platform (n=1,024) only 20% listened to the same thematically relevant content for this study.

Table 3. Top ten most popular messages listened to by treatment group on the platform (Dec 2023 - July 2024)

No	Message Title	Key Themes	Times Reached	Times Listened	Total Number of Listeners	C2L Ratio
1	Dynamic daily weather report	weather	14140	14140	2140	100%
2	To learn about what food keeps us healthy	health, nutrition, anemia	8858	6710	1753	89%
3	Dynamic daily news bulletin	news	5959	4488	1046	86%
4	To know what a healthy weight is	health, nutrition, BMI	4335	3585	1310	92%
5	To learn more about cervical cancer	SRH	4056	3417	1179	91%
6	What is folic acid and why take it?	health, pregnancy	4006	3177	1208	89%
7	What is sex?	puberty, SRH	3852	3500	1513	95%

Formatted Table

No	Message Title	Key Themes	Times Reached	Times Listened	Total Number of Listeners	C2L Ratio
8	DIV Drama Episode 1: "Family planning for a better future"	FP, SRH	3675	2053	633	77%
9	To know the four ways to improve your chance for a healthy pregnancy	SRH, pregnancy	3660	2205	1453	78%
10	To learn how iron keeps us strong	nutrition, anemia	3558	2950	1092	89%

Notes: A listener is defined as someone who has listened to at least 75% of a key message. *C2L (caller-to-listener ratio) defines the % completion listening rate of a message, i.e. the proportion of callers who listen to the message all the way to the end. BMI=body mass index, FP=family planning, SRH=sexual and reproductive health

Table 4 shows estimated impact on knowledge. We find a 0.07 SD increase in the primary knowledge outcome score in unadjusted models, and an increase of 0.06 SDs in fully adjusted models. Treatment also increased the average number of reproductive methods women reported to be familiar with by 0.11 points (implying that one in 10 women know one additional method). For nutrition, the treatment reduced the false belief that local herbs could cure anemia by 3 % pts.

Table 4: Impact on Knowledge

Outcome	Reproductive health knowledge z-score	Beliefs that local herbs cure anemia	Number of contraceptive methods familiar [illegible]
Treatment effect	0.0708***	-0.0371**	0.116**
(unadjusted)	(0.0265)	(0.0152)	(0.0461)
Adjusted treatment effect	0.0591**	-0.0328**	0.0941**
	(0.0252)	(0.0148)	(0.0455)
Control group average	9.742	0.406	10.33

Survey modality	In person	In person	Phone
Observations	4,877	4,877	5,233

Notes: Table shows estimated impact on primary outcomes. Coefficients displayed are mean differences with standard errors in parentheses. Adjusted models control for age group (age < 20, age 20-34 (ref), age >=35), educational attainment, district, baseline family planning knowledge, baseline hemoglobin level and baseline body mass index. ~~Standard~~Numbers in parentheses are cluster-robust standard errors ~~were clustered at the enumeration area level.~~. Asterisks indicate p-value range of estimated differences: *p-value<0.10, **p-value<0.05, ***p-value<0.01~~,~~

Fig 3 shows item-specific changes in sexual and reproductive health knowledge. Largest improvements were observed for myths regarding male condoms causing infertility and post-sex urination preventing pregnancy; small improvements were seen for most questions.

Figure 3: Item-level change in knowledge

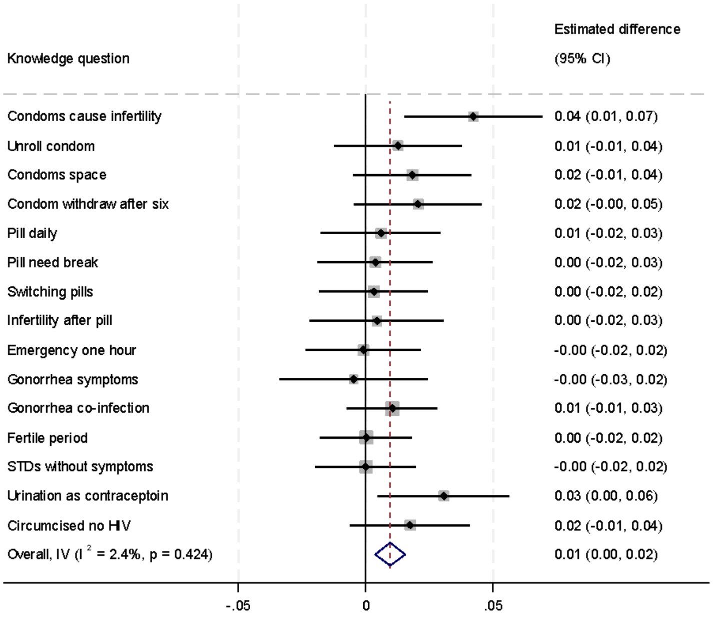

Notes: each point/square on the graphic represents the estimated coefficient obtained from regressing a binary indicator of giving a correct answer to the specific question on treatment - lines illustrate 95% confidence intervals.

Supplemental Materials Figure SF1 further illustrates the observed changes in knowledge for the item with the largest absolute change in knowledge score. Supplemental Materials Table ST1 shows the proportion of women answering each item correctly at baseline and endline by treatment status for all items investigated.

Table 5 summarizes the estimated impact on sexual behavior as reported in person as well as on the phone. The two survey modalities yielded consistent results with 40% of women using modern contraception and the intervention resulting in an insignificant additional 2% of women using modern contraception. Positive impacts were found for the number of methods ever used (+0.14 methods in adjusted models) and the intention to use modern contraception in the future (+ 5% pts).

Table 5: Impact on self-reported sexual behavior

Outcome	Currently uses any ~~contraception (in person interview)~~ ~~a~~contraception[a)]	Currently uses modern ~~contraception (in person report interview)~~ ~~a~~contraception[a)]	Can show contraception to interviewer	Number of methods ever used ~~(phone)~~	Currently uses any ~~contraception (phone interview)~~ ~~a~~contraception[a)]	Currently uses modern ~~contraception (phone)a~~ contraception[a)]	Plans to use modern contraception in the future
Treatment effect	0.0162	0.0193	0.00320	0.164***	0.0189	0.0218	0.050***
(unadjusted)	(0.0147)	(0.0137)	(0.00667)	(0.0449)	(0.0143)	(0.0138)	(0.0124)
Adjusted treatment effect	0.0157	0.0198	0.00384	0.140***	0.0162	0.0192	0.0468***
	(0.0145)	(0.0134)	(0.00638)	(0.0410)	(0.0138)	(0.0133)	(0.0118)
Control group average	0.455	0.396	0.0718	2.956	0.474	0.396	0.396
Survey modality	In person	In person	In person	Phone	Phone	Phone	Phone
Observations	4,877	4,877	4,877	5,233	5,233	5,233	5,233

Notes: Coefficients displayed are mean differences with standard errors in parentheses. Adjusted models control for age group (age < 20, age 20-34 (ref), age >=35), educational attainment, district, baseline family planning

Formatted Table

Formatted: Superscript

Formatted: Superscript

Formatted: Superscript

Formatted: Superscript

knowledge, baseline hemoglobin level and baseline body mass index. StandardNumbers in parentheses are cluster-robust standard errors were clustered at the enumeration area level.. Asterisks indicate p-value range of estimated differences: *p-value<0.10, **p-value<0.05, ***p-value<0.01. a) The full list of contraceptives is shown in Supplemental Materials S2. Contraceptives not considered as "modern" are beads/calendar method, withdrawal and breastfeeding.

Fig 4 provides further details on current use of contraceptive methods with a focus on methods ever used. The intervention increased the use of implants, pills and condoms by 3-4 % pts – changes on other methods were not significant.

Figure 4: Estimated impact on contraceptives ever used

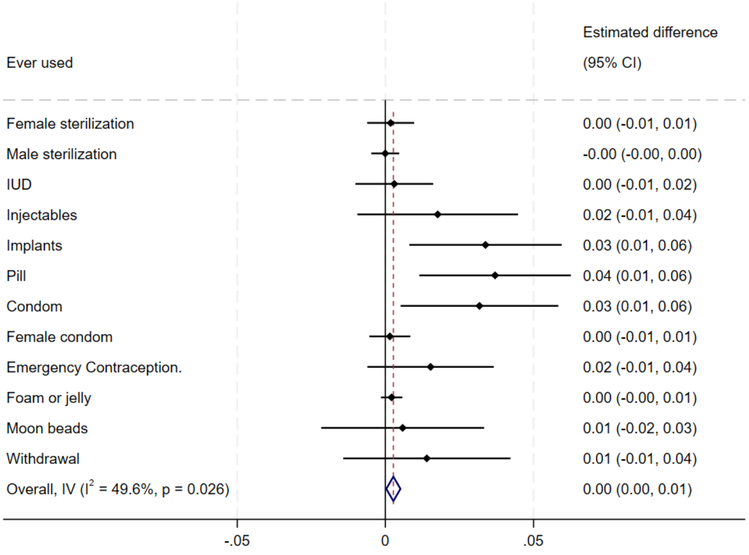

Figure notes: each point/square on the graphic represents the estimated coefficient obtained from regressing a binary indicator of having ever used the method in question on treatment - lines illustrate 95% confidence intervals.

Table 6 shows estimated impact on prenatal care. A total of 398 current pregnancies, as well as 653 pregnancies completed between baseline and endline were analyzed. No differences were found in antenatal care attendance or deworming. Treatment increased the likelihood of taking iron in current pregnancies by 8% pts and the likelihood of taking supplements in recently completed pregnancies by 4% pts [0.3,7.7].

Table 6: Impact on Prenatal Care

Outcome	Attended antenatal care	Currently taking iron	Deworming	Antenatal care for recent pregnancy	Recent pregnancy >=4 visits	Recent pregnancy: iron	Recent pregnancy: deworming
Treatment effect	0.0593	0.0845*	0.0167	0.00955	0.0229	0.0361*	0.0374
(unadjusted)	(0.0449)	(0.0431)	(0.0459)	(0.0161)	(0.0414)	(0.0189)	(0.0314)
Adjusted treatment effect	0.0453	0.0758*	0.00757	0.0156	0.0209	0.040**	0.0369
	(0.0468)	(0.0456)	(0.0483)	(0.0158)	(0.0412)	(0.0189)	(0.0310)
Control group average	0.621	0.605	0.451	0.958	0.454	0.901	0.808
Sample	Women currently pregnant			Women completing pregnancy last year			
Survey modality	In person						
Observations	398	398	398	653	619	653	647

Notes: Coefficients displayed are mean differences with standard errors in parentheses. Adjusted models control for age group (age < 20, age 20-34 (ref), age >=35), educational attainment, district, baseline family planning knowledge, baseline hemoglobin level and baseline body mass index. ~~Standard errors were clustered at the enumeration area level.~~Numbers in parentheses are cluster-robust standard errors. Asterisks indicate p-value range of estimated differences: *p-value<0.10, **p-value<0.05, ***p-value<0.01

Table 7 shows estimated impact on other behavior. The intervention increased women's efforts to eat healthy by 4% pts; interestingly, these efforts were generally not linked to losing weight; on average, treated women were 2.5% less likely to attempt for weight loss, and 3% pts more likely to try to gain weight. In terms of food intake, the treatment appears to have increased the frequency of fruit and vegetable consumption, consistent with the results on healthier diets. This aligns with consistent messaging on increasing healthy habits such as regular activity, the inclusion of nutritious and iron rich foods, and maintaining a healthy body weight.

Table 7: Impact on Other Health Behaviors

Outcome	Aims for health diet	Tried to lose weight	Tried to gain weight	Times at fruit	Times ate vegetables	Times ate protein	Currently taking iron supplements	Exercised

Formatted Table

Formatted: Font: 10 pt

Formatted: Space After: 0 pt, Line spacing: single

Formatted Table

Treated	0.0407***	-0.025**	0.0355***	0.0686**	0.0484**	-0.00060	0.0156	0.0154
(Unadjusted)	(0.0144)	(0.0108)	(0.0128)	(0.0346)	(0.0245)	(0.0183)	(0.00988)	(0.0124)
Adjusted treatment effect	0.0312**	-0.025**	0.0316**	0.0583*	0.0371*	0.00202	0.0174*	0.0119
	(0.0123)	(0.0106)	(0.0126)	(0.0298)	(0.0213)	(0.0172)	(0.00965)	(0.0118)
Control group average	0.552	0.255	0.242	1.335	1.332	1.760	0.136	0.335
Survey modality	Phone survey							
Observations	5,233	5,233	5,233	5,233	5,232	5,233	5,233	5,233

Notes: Coefficients displayed are mean differences with standard errors in parentheses. Adjusted models control for age group (age < 20, age 20-34 (ref), age >=35), educational attainment, district, baseline family planning knowledge, baseline hemoglobin level and baseline body mass index. ~~Standard~~Numbers in parentheses are cluster-robust standard errors~~ were clustered at the enumeration area level.~~. Asterisks indicate p-value range of estimated differences: *p-value<0.10, **p-value<0.05, ***p-value<0.01~~.~~

Formatted: Font: 12 pt

Table 8 shows estimated impacts on pregnancy and birth outcomes. 41% of currently pregnant women in the control group reported that the current pregnancy was not planned; this proportion was 5% pts smaller in the treatment group, but not significantly different. Unplanned pregnancies in the past were slightly more common among treated women (but not statistically different). The proportion of previous pregnancies lost as well as the proportion of most recent births born with low birthweight (< 2500 grams) were substantially lower in the treated group – these differences were however not significant statistically due to the small number of observations here.

Table 8: Impact on pregnancy and birth outcomes

Outcome	Pregnancy was unplanned	Birth spacing < 2 years	Previous pregnancy unplanned	Previous pregnancy low	Previous pregnancy lost

Formatted Table

				birthweight	
Treatment effect	-0.0605	0.0519	0.0260	-0.0143	-0.0301
(unadjusted)	(0.0481)	(0.0546)	(0.0341)	(0.0246)	(0.0216)
Adjusted treatment effect	-0.0531	0.0496	0.0215	-0.0157	-0.0305
	(0.0495)	(0.0516)	(0.0337)	(0.0245)	(0.0209)
Control group average	0.410	0.372	0.261	0.0888	0.0384
Survey modality	In person				
Sample	Currently pregnant		Completed pregnancy recently		
Observations	398	371	653	565	653

Notes: Coefficients displayed are mean differences with standard errors in parentheses. Adjusted models control for age group (age < 20, age 20-34 (ref), age >=35), educational attainment, district, baseline family planning knowledge, baseline hemoglobin level and baseline body mass index. StandardNumbers in parentheses are cluster-robust standard errors were clustered at the enumeration area level.. Asterisks indicate p-value range of estimated differences: *p-value<0.10, **p-value<0.05, ***p-value<0.01.

Formatted: Space After: 0 pt, Line spacing: single

Table 9 shows estimated impact on biomarkers. While we see some lower rates of anemia among treated women who currently are, or recently were pregnant (Table 9, column 2), these differences are not statistically significant. No changes were found for BMI overall, or any of the categories analyzed (underweight, overweight, obese).

Table 9: Impact on biomarkers

Outcome	Anemic (Hg<12 g/dL)	Anemic (Hg<12 g/dL)	Body mass index	Overweight (BMI >=25, <30)	Obese (BMI >=30)	Underweight (BMI <18.5)
Treatment effect	0.00745	-0.0495	0.183	0.00277	0.0150	0.0120
(unadjusted)	(0.0129)	(0.0315)	(0.165)	(0.0126)	(0.0106)	(0.00826)

Formatted Table

Adjusted treatment effect	0.0111	-0.0468	0.0922	0.000613	0.0102	0.0140*
	(0.0127)	(0.0311)	(0.111)	(0.0121)	(0.00831)	(0.00808)
Control group average	0.23	0.23	24.89	0.26	0.15	0.08
Sample	All	Current or recent pregnancy	All	All	All	All
Observations	4,664	992	4,852	4,852	4,852	4,852

Notes: Coefficients displayed are mean differences with standard errors in parentheses. Adjusted models control for age group (age < 20, age 20-34 (ref), age >=35), educational attainment, district, baseline family planning knowledge, baseline hemoglobin level and baseline body mass index. ~~Standard errors were clustered at the enumeration area level.~~Numbers in parentheses are cluster-robust standard errors. Asterisks indicate p-value range of estimated differences: *p-value<0.10, **p-value<0.05, ***p-value<0.01.

Table 10 shows the result of the per protocol analysis, where we compare control women who had never called the platform to treated women who had called the platform at least five times.. As expected, estimated impacts on knowledge scores are higher among compliers; however, these estimates become substantially smaller again when we adjust for women's differences in education and reproductive health knowledge at baseline.

Table 10: Per protocol analysis

Outcome	SRH Knowledge z-score	Currently uses modern method	Uses iron supplements	Used iron supplements	Lost pregnancy	Anemic	Anemic post pregnancy
Treatment effect	0.127***	0.0409**	0.0571	0.000959	-0.00654	0.00586	-0.0496
(Unadjusted)	(0.0332)	(0.0159)	(0.0515)	(0.0102)	(0.00671)	(0.0147)	(0.0345)
Adjusted treatment	0.0650**	0.0206	0.0575	0.0117	-0.00911	0.0168	0.0168
effect	(0.0308)	(0.0155)	(0.0523)	(0.00954)	(0.00668)	(0.0144)	(0.0144)

Formatted Table

Control group average	-0.0613	0.374	0.643	0.915	0.0386	0.229	0.389
Sample	All	All	Pregnant now	Pregnant last year		All	Pregnant
Restriction	Compliers only						
Observations	3,561	3,561	292	3,313	3,313	3,400	727

Notes:*Only women in the control group who never called the service, and women in the treatment group who called the service at least five times are included in this table. Coefficients displayed are mean differences with standard errors in parentheses. Adjusted models control for age group (age < 20, age 20-34 (ref), age >=35), educational attainment, district, baseline family planning knowledge, baseline hemoglobin level and baseline body mass index. ~~Standard~~Numbers in parentheses are cluster-robust standard errors ~~were clustered at the enumeration area level.~~. Asterisks indicate p-value range of estimated differences: *p-value<0.10, **p-value<0.05, ***p-value<0.01. SRH=sexual and reproductive health.

Stratified Results

Table 11 shows stratified impact results by age and educational attainment group, as well as by district. For age, we find the weakest effects among women under age 20, and largest impacts among women 35 years and older. Educational level seemed to have little impact, with only slightly larger effects for women with secondary or higher education compared to women with less education. At the district level, impacts vary substantially, with the largest treatment effects in Madi-Okollo (the 0.12 SD increase here is almost twice as large as the effect we see in the sample overall), and the smallest impacts in Rwampara (0.02 SD).

Table 11: Stratified results

Stratum	Age <20	Age 20 - 34	Age 35+	Secondary or higher education	Primary education	Kampala	Katakwi	Madi-Okollo	Rwampara
Treatment effect	0.0213	0.0674*	0.0901*	0.0723*	0.0587*	0.0949**	0.0828*	0.122*	0.0221
	(0.127)	(0.0344)	(0.0500)	(0.0430)	(0.0331)	(0.0431)	(0.0455)	(0.0654)	(0.0517)
Observations	291	2,978	1,608	1,804	3,073	1,472	1,093	650	1,662

Formatted Table

Notes: Coefficients displayed are mean differences with standard errors in parentheses. Adjusted models control for age group (age < 20, age 20-34 (ref), age >=35), educational attainment, district, baseline family planning knowledge, baseline hemoglobin level and baseline body mass index. ~~Standard~~Numbers in parentheses are cluster-robust standard errors ~~were clustered at the enumeration area level.~~. Asterisks indicate p-value range of estimated differences: *p-value<0.10, **p-value<0.05, ***p-value<0.01.

Formatted: Space After: 0 pt, Line spacing: single

Harms statement

No harm was reported during the trial.

Discussion

The main objective of this study was to assess the impact of Viamo's call-in platform on health knowledge, behaviors and outcomes. Conceptually, knowledge platforms could result in changes on three distinct levels: changes in health knowledge, changes in health behavior, and changes in health outcomes. The results presented here suggest that changes in knowledge definitely occurred, with treated women displaying improved sex and reproductive health knowledge, being better informed about anemia treatment and more familiar with a larger range of contraceptive methods. This increased familiarity with contraceptive knowledge was also partially visible in contraceptive use. Treated women were on average only marginally (and insignificantly) more likely to use modern contraception, but used a different mix of contraceptives, with higher usage of implants, and somewhat reduced reliance on injectables and calendar methods. In terms of future plans, the intervention seems to have substantially increased the proportion of women who say they will use modern contraception in the future.

For anemia - one of the key health challenges addressed on the platform - the treatment seems to have resulted in an increased uptake of supplements during pregnancy; these higher rates of supplementation also seem consistent with marginally lower rates of anemia among recently pregnant women. The estimated differences in anemia in this subgroup are however not statistically significant, which may partially be due to the relatively small number of women. Despite the high rates of anemia among non-pregnant women, we did not see any change in general protein intake or consumption of iron supplements. Given this lacking change (and the small proportion of women benefiting from additional supplementation during pregnancy), the insignificant impact on general hemoglobin levels makes sense.

We also see some interesting declines in the rates of pregnancy loss and the prevalence of low-birth-weight births in this subsample, but did not have sufficient statistical power here due to the relatively small number of women completing a pregnancy within the study period.

Given that a larger proportion of women in the treatment group claimed to aim for a healthy diet and to eat more fruit and vegetables, nutrition related content clearly reached its target.

Interestingly, the messages received around "healthy body shapes" do not seem to have triggered a major shift towards eating less and lower BMIs, but seems to have induced a desire to change in both directions, i.e. we see an increasing proportion of women who want to lose weight and an increasing proportion of women who want to gain weight (and hence a smaller proportion of women who want to stay as they are). These shifts in intentions and behaviors are not seen in the anthropometric data - the intervention neither changed average BMI nor the proportions of women classified as underweight, overweight or obese.

In terms of the primary outcome, our results seem consistent with a recent review of mHealth intervention, which suggests that digital information sources can improve sexual and reproductive health knowledge as well as use of sexual and reproductive health services ~~[11].~~[16]. Compared to the estimates reported for an interactive intervention in Ghana [~~7, 12~~11, 17] the effect sizes observed here seem smaller, which seems plausible given that not all women engaged with the service on a regular service. Compared to previous studies conducted in other parts of Africa we also observed rather high sexual and reproductive health knowledge at baseline – most women were for example familiar with a range of contraceptive methods (beyond traditional methods, condoms and pills), and myths regarding side effects of contraception were less common that seen in other settings [~~7, 12~~11, 17] – this likely also contributes to the somewhat smaller knowledge impacts seen here.

In terms of policy, one of the main advantages of the platform used is its relatively low cost – particularly when run at large scale; most of the costs of content development are related to the initial set-up, while operating costs are relatively minor. More research is needed here to investigate the cost-effectiveness of these new mobile technology channels as tools to improve health knowledge among vulnerable populations relative to traditional channels such as radio, tv or community outreach activities.

Despite the very large, stratified and regionally representative sample, the study has some limitations. First, there were not as many pregnant women as anticipated, reducing power on pregnancy-related behaviors and outcomes to some extent. Second, we found that the proportion of women using Airtel phones was substantially lower in some rural districts, making it very difficult to reach enrollment targets in some communities. We compensated this by enrolling more women in clusters with higher coverage. Even though a more selected sample clearly does not undermine the internal validity of the study (since randomization was done at the individual level), it is not clear whether the final set of women enrolled in the study

is fully representative of the larger populations in the four study districts; it is also not clear if the four districts are fully representative of the country as well as Uganda overall - sincea whole. Since the service is currently only available to Airtel users, this study simply focused on women with access to this service. Even though it seems plausible that similar effects could be found with other phone providers, it is not clear if the results of this study can be generalized to the country as a whole or to other countries. Third, many of the behaviors measured were self-reported; while we implemented verification measures by taking pictures of antenatal and child health cards and asking women for evidence of contraceptive packaging, such direct evidence was generally only available for a small minority of women, and could thus not be used in the analysis. It is clearly possible that access to the service changes norms and reporting bias, which could explain some of the behavioral changes reported and detected in our analysis. The primary outcome of the study - the reproductive knowledge score - should not be subject to any reporting bias as all women should have the incentive to give the correct answer on the true/false knowledge quiz. Fourth, the individual-level randomization made the study vulnerable to spillover effects. Qualitative feedback collected at the end of the study suggests that treated women both invited other community members – spouses, friends and other family members – to use the service and actively shared new information with them. Given that some of this shared information likely reached the control group, it seems likely that the impact reported here underestimate the true causal effect of the program. We controlled for direct service use by excluding active users in the per protocol analysis (Table 10) – this does however not capture direct sharing of knowledge among community members.

Conclusions

Overall, the results presented here suggest that the platform can effectively improve health knowledge and to some extent also health behaviors among vulnerable populations. These changes in knowledge, awareness and intentions could plausibly also induce changes in health outcomes in the long run; longer-term studies will likely be needed to accurately measure such effects. Future research should also aim to quantify knowledge spillovers within communities as well as the cost-effectiveness of call-in platform like the one analyzed here relative to other communication channels such as TV, radio, posters or community-based activities.

Acknowledgements: We would like to thank all involved staff members at IPA Uganda as well as in the Viamo team for their efforts and continued support of the project. We are also grateful to the more than 6000 study participants for their willingness to participate in the study. Lastly, we would like to thank the USAID Development Innovation Ventures (DIV) team for their input and feedback throughout the project.

References

1. World Bank, *Individuals using the Internet (% of population) | Data*. 2025.

Formatted: Font: Garamond, 11 pt

~~2~~2. Sarikhani, Y., S.M. Najibi, and Z. Razavi, *Key barriers to the provision and utilization of maternal health services in low-and lower-middle-income countries; a scoping review*. BMC Womens Health, 2024. **24**(1): p. 325.

3. D'Souza, P., et al., *Factors influencing contraception choice and use globally: a synthesis of systematic reviews*. The European Journal of Contraception & Reproductive Health Care: The Official Journal of the European Society of Contraception, 2022. **27**(5): p. 364-372.

Formatted: Font: Garamond, 11 pt

~~3~~4. Conde-Agudelo, A. and J.M. Belizán, *Maternal morbidity and mortality associated with interpregnancy interval: cross sectional study*. Bmj, 2000. **321**(7271): p. 1255-9.

5. Doku, D.T. and S. Neupane, *Survival analysis of the association between antenatal care attendance and neonatal mortality in 57 low- and middle-income countries*. Int J Epidemiol, 2017. **46**(5): p. 1668-1677.

6. Ota, E., et al., *Antenatal interventions for preventing stillbirth, fetal loss and perinatal death: an overview of Cochrane systematic reviews*. Cochrane Database Syst Rev, 2020. **12**(12): p. Cd009599.

7. Noar, S.M., *A 10-year retrospective of research in health mass media campaigns: where do we go from here?* Journal of Health Communication, 2006. **11**(1): p. 21-42.

Formatted: Font: Garamond, 11 pt

~~4~~8. Wakefield, M.A., B. Loken, and R.C. Hornik, *Use of mass media campaigns to change health behaviour*. Lancet, 2010. **376**(9748): p. 1261-1271.

Formatted: Font: Garamond, 11 pt

~~5~~9. Stead, M., et al., *Mass media to communicate public health messages in six health topic areas: a systematic review and other reviews of the evidence*. Public Health Research. 2019, Southampton (UK): NIHR Journals Library.

Formatted: Font: Garamond, 11 pt

~~6. Uganda Communications Commission, *Market performance report 2022/2023*. 2023, Uganda Communications Commission.~~

~~7~~10. International Telecommunication Union *Facts and Figures 2024 – Mobile network coverage*. ITU Digital Reports, 2024.

11. Rokicki, S., et al., *Impact of a Text-Messaging Program on Adolescent Reproductive Health: A Cluster–Randomized Trial in Ghana*. American Journal of Public Health, 2017. **107**(2): p. 298-305.

Formatted: Font: Garamond, 11 pt

~~8~~12. Unger, J.A., et al., *Short message service communication improves exclusive breastfeeding and early postpartum contraception in a low- to middle-income country setting: a randomised trial*. BJOG: an international journal of obstetrics and gynaecology, 2018. **125**(12): p. 1620-1629.

Formatted: Font: Garamond, 11 pt

~~9~~13. Harrington, E.K., et al., *An mHealth SMS intervention on Postpartum Contraceptive Use Among Women and Couples in Kenya: A Randomized Controlled Trial*. American Journal of Public Health, 2019. **109**(6): p. 934-941.

Formatted: Font: Garamond, 11 pt

~~10~~14. UNDP. *Digital X Solution Catalogue*. 2025 10 Sep 2025].

Formatted: Font: Garamond, 11 pt

~~11~~15. Huber, P.J., *The behavior of maximum likelihood estimates under nonstandard conditions. Proceedings of the Fifth Berkeley Symposium on Mathematical Statistics and Probability. Berkeley, CA 1, 221–233. 1967.*

16. Onukwugha, F.I., et al., *The effectiveness and characteristics of mHealth interventions to increase adolescent's use of Sexual and Reproductive Health services in Sub-Saharan Africa: A systematic review*. PLOS ONE, 2022. **17**(1): p. e0261973.

Formatted: Font: Garamond, 11 pt

~~12~~17. Rokicki, S. and G. Fink, *Assessing the reach and effectiveness of mHealth: evidence from a reproductive health program for adolescent girls in Ghana*. BMC Public Health, 2017. **17**(1): p. 969.

Formatted: Font: Garamond, 11 pt

Reviewer #4:

Thank you for sharing this study for review. Below are some points that might help the authors revamp their manuscript.

Thank you for your kind and constructive comments – we have done our best to address all of them as outlined in detail below.

Abstract
It will be beneficial to add analysis section to the methods section of the abstract. Which statistical test were used.

Thank you for this suggestion. We have added the following sentence to the Abstract to clarify the empirical models used:

"Linear regression models were used to estimate mean differences in outcomes between treatment and control. Standard errors were corrected for the cluster-based sampling of women."

If you decide to retain vulnerable population in the title, it will be important to clarify which specific vulnerable population you studied. Otherwise, I will suggest that you remove the vulnerable population in the title and put "women of reproductive age", and hence the background section will need to be revised.

We have removed the "vulnerable populations" in the background section and now write:

"..knowledge remains limited among women of reproductive health in many low and middle income countries"

I am curious about the choice of test used in the data analysis and why the continuous variables

As stated above, we used linear regression models to estimate mean differences in outcomes between treated and controls. We also considered logistic regression models for the binary outcomes, but decided to stick with mean differences because they make comparisons in outcomes easy to communicate (and use cluster-robust standard errors to address non-normal residuals for binary outcomes).

Introduction
The first paragraph in this section sounds like a problem statement and also this entire section needs to be revised. I suggest that this section be revised as follows:
Introduce the reproductive age group as the predominant age group in Uganda
Health outcomes common among the reproductive age group (vulnerable population)
Emerging role of mobile phone app (Viamo Call-in Information Services)/internet coverage/technology in health education and improvement of reproductive and sexual health outcome in the Ugandan population. The proportion of the Ugandans with access to cell phones and/or internet. Previous studies in the impact of other cell phone apps on reproductive and sexual health outcome. This section should now end with the problem statement and the rational for this study.

We have restructured the Introduction as suggested and added several additional references. The first paragraph introduces the knowledge gap and highlights the health consequences of this gap for mothers and offsprings. The second paragraph summarizes current research on how to best improve knowledge among women of reproductive age. The third paragraph introduces cell phones as new tool and highlights key studies. The last one provides the objective and rationale of the study.

Methods
Study design: Were the 4 purposively selected districts representative of the entire Uganda population. This may act as a threat to external validity given that the 4 district doesn't represent the minority regions. I guess this is a limitation.

We agree this is a limitation and acknowledge this in the revised Discussion section, where we write: .

"…it is not clear whether the final set of women enrolled in the study is fully representative of the larger populations in the four study; it is also not clear if the four districts are fully representative of the country as a whole."

Sampling technique: I think the sampling technique you described was a multistage cluster sampling technique and not a 2-stage random sampling. Also take a good look at different studies and revise the entire sampling procedure. The selection of 45 per cluster makes me worry if the selection was proportionate or representative each cluster? In order words, were the clusters of equal population for 45 to be selected from each clusters?

We have changed the description to multi-stage sampling. The sampling was not proportional to population size and likely did not generate a fully representative sample – we say this clearly in the Discussion, where we write:

"Second, we found that the proportion of women using Airtel phones was substantially lower in some rural districts, making it very difficult to reach enrollment targets in some communities. We compensated this by enrolling more women in clusters with higher coverage. Even though a more selected sample clearly does not undermine the internal validity of the study (since randomization was done at the individual level), it is not clear whether the final set of women enrolled in the study is fully representative of the larger populations in the four study districts."

Sample size: How was the sample size of 6000 arrived at? It will make sense to add how it was calculated

We explain this in the revised Power Calculations section, where we write:
"The study was powered to detect a 0.1 standard deviation (SD) difference in standardized knowledge scores (mean 0, SD 1) with power 0.9. Assuming an average cluster size of 30, a follow-up rate of 90% and a design effect of 1.3, this required 100 clusters per arm, and a total sample size of 6000 women."

Randomization: The section on randomization is very scanty and needs clarification. At this point its still not clear what the non-treatment arm received. Did they also benefit from Viamo Call-in Information Services, its important to clearly state what the intervention the 2 groups received. Its important to clearly define the intervention and the non-intervention group (treatment vs control group) and exactly what they received because its not clear.

Apologies for the lack of clarity. We have added the following text to the Randomization section:

"Women selected for the treatment arm were then provided with a personalized introduction to the service (after the baseline survey was completed) and called the service directly from their own phone with the support of the interviewer to see how the system works. Treated women were also given a promotional calendar to serve as a physical reminder on how to navigate the service and the benefits of the service. Women in the control group were not given any intervention. Given that the service was freely available to all women, they were in theory able to call the service at any time, but not given any encouragement or support to do so by the study team."

Primary and secondary outcomes: The definition of primary and secondary outcomes (how were they measured) needs to be included in the methods section. Which tool was used to measure knowledge? Was it pretested or was it a validated tool?

Apologies for the lack of detail here. We now explain this in more detail in the revised Methods section:

"The two primary outcomes of the study were reproductive health knowledge and nutritional supplementation in pregnancy. For reproductive health, we used a 15-item questionnaire of true/false statements (items listed in Supplemental Materials S2). This tool was based on previous studies conducted in sub-Saharan Africa [11] and pretested locally to make sure all items were suitable for the study population. To facilitate interpretation of regression results, we converted the raw scores (number of correct answers out of 15) into z-scores with mean zero and standard deviation one. For nutrition knowledge, we analyzed a binary indicator for women (falsely) believing that herbs can cure anemia. For nutritional supplementation during pregnancy, we used self-reported iron supplementation as an outcome measure. Secondary outcomes included contraceptive knowledge (number of methods respondents are familiar with), contraceptive use (currently using any modern method), prenatal care uptake (any, number of visits), hemoglobin level (as a biomarker indicating actual iron intake), body mass index, and birth spacing.

For pregnancy related outcomes, we analyzed two separate sets of women: first, women reporting to be pregnant at the time of the endline survey (currently pregnant), and second, women who completed a pregnancy in the year preceding the endline survey (recently pregnant). For current pregnancies, we analyzed self-report iron supplementation as well as self-reported use of antenatal care services, and whether women indicate the pregnancy was planned. For recently completed pregnancies, we analyzed the same variables, but also analyzed pregnancy loss and low birth weight (reported weight at birth < 2.5kgs) as additional secondary outcomes. "

Data analysis: I have noticed that the majority of the variables were analyzed as continuous variables. Did you consider converting the continuous variables (scores) to categorical variables which then permits you to use different methods.

We have explained this in further detail in the revised Methods section, where we write:

"All primary models were estimated intent-to-treat, comparing outcomes between treated and control women at endline. For all outcomes, we estimated both unadjusted linear regression models (not including any covariates) and fully adjusted models, where we controlled for age, district, marital status, educational attainment and baseline knowledge variables. The main rationale for using linear regression models was to generate mean differences estimates that could be compared across the rather large set of outcome variables. To adjust for non-normal residuals, we used Huber's cluster-robust standard errors [15]."

Results

The presentation of results is not very clear. The tables are many and its not clear labelled if its correlation coefficient or a p-value.

We agree that there are many, but think it is important to show all of the results – also one of the reasons we chose PLOS ONE for this paper. We have added notes to all tables to be clear about what is shown (coefficients, SEs, p-values). For example, for the first regression Table (T2), the notes now say:

*"All point estimates displayed correspond to estimated mean differences in outcomes. Numbers in parentheses are cluster-robust standard errors. Asterisks indicate p-value range of estimated differences: *p-value<0.10, **p-value<0.05, ***p-value<0.01"*

Discussion

The discussion needs to be revised. It lack the comparison and contrast of the study findings with studies done locally and international.

There are not so many studies of similar nature, but we try to compare the main outcomes to the related literature in the Discussion, where we write:

"In terms of the primary outcome, our results seem consistent with a recent review of mHealth intervention, which suggests that digital information sources can improve sexual and reproductive health knowledge as well as use of sexual and reproductive health services [16]. Compared to the estimates reported for an interactive intervention in Ghana [11, 17] the effect sizes observed here seem smaller, which seems plausible given that not all women engaged with the service on a regular service. Compared to previous studies conducted in other parts of Africa we also observed rather high sexual and reproductive health knowledge at baseline – most women were for example familiar with a range of contraceptive methods (beyond traditional methods, condoms and pills), and myths regarding side effects of contraception were less common that seen in other settings [11, 17] – this likely also contributes to the somewhat smaller knowledge impacts seen here. "

Please let us know if there are any papers that we missed and should be cited here.

Reviewer #5:

I appreciate the submission of this manuscript. Please find my comments below, which may be helpful in improving the quality of your work.

Thank you for the kind and helpful comments – we really appreciate your input and have done our best to address all suggestions and explained in further detail below.

Pg. 1/26: It is recommended that the phrase "results from" in the title be omitted, as this would shorten the title without detracting from its intended meaning.

Thank you for this suggestion – we have removed "results from" the title and agree that the shortened version sounds better.

Pg. 2/26: - It is recommended that the phrase "and Outcomes" be included in the title, as currently presented at the end of the background, to ensure that the title is complete.

We believe these titles are given by the journal and thus are not allowed to modify them. We do state the objective in the background; the outcomes are provided in the Methods subsection.

- Please ensure that the sampling method, data collection tools, a brief description of the data collection procedure, and the statistical tests used are clearly described in the methods section.

We have expanded the Methods section to provide more details: it now reads as follows:

"We conducted a 2-arm open label randomized controlled trial involving 6,000 randomly selected women, ages 18-49 years, with access to a simple feature Airtel cell phone in Uganda. Fifty enumeration areas were randomly selected in Kampala, Madi-Okollo, Rwampara and Katakwi districts. Consenting women were asked to complete a baseline and endline survey implemented by trained interviewers using tablets through home visits. After completion of baseline, women were randomized with equal probability to treatment and control using a tablet-generated random number draw. Study staff introduced treated women to the call-in service and encouraged its use through various incentives and promotional messages. Primary outcomes were sexual and reproductive health knowledge scores as well as iron supplementation in pregnancy. Secondary outcomes included self-reported use of contraception, healthy diets, birth spacing and birth outcomes as well as hemoglobin levels and BMI. Linear regression models were used to estimate mean differences in outcomes between treatment and control. Standard errors were corrected for the cluster-based sampling of women."

Pg. 3/26: Please mention keywords at the end of the abstract.

We have added the following keywords at the end of the abstract:

***Keywords**: reproductive health, call-in-services, health knowledge, Uganda, Viamo.*

Pg. 4/26: Please provide further details regarding the Viamo platform and its operational mechanisms. Specifically, clarify whether the platform enables two-way interaction and whether users can experience a form of interactive support comparable to counseling, or whether information is delivered in a predominantly one-way manner, functioning mainly as a communication channel for educational purposes.
Please review relevant studies that have implemented similar interventions in the same research area, either locally or in other regions. Additionally, address studies that have evaluated the effectiveness of the Viamo platform or comparable mobile- or voice-based platforms for different purposes. Finally, summarize the existing literature and highlight the comparative advantage and necessity of the present intervention by identifying the gaps it aims to address. If no relevant studies

are available, this should be clearly stated.

Based on the feedback provided by Reviewer 4, we have removed the description of the service from the Introduction (page 4) and instead provide more details on the platform in an expanded Methods-Intervention section, where we provide the requested details. We compare this to other studies in the Discussion section – there is however not that much literature on similar models. In practice, Viamo does not provide direct counseling, but it offers a range of different topics users can select from – we think it can be best described as something like a "user-guided learning system".

Pg. 5/26 (Methods section):
- Please specify the exact nature of any encouragement designs and whether they may have preferentially attracted certain subgroups of participants. For example, as noted on page 7, "Participants received UGX 10,000 (~USD $2.77) of phone credit as a token of gratitude after each survey interview." Offering such incentives could disproportionately attract individuals with lower socioeconomic status, potentially limiting the generalizability. Therefore, if participants were aware of the incentives at the time of study invitation, this should be explicitly stated and discussed in the study limitations.

We have provided further details on the encouragement in the Methods-Randomization section now, where we write:

"Women selected for the treatment arm were then provided with a personalized introduction to the service (after the baseline survey was completed) and called the service directly from their own phone with the support of the interviewer to see how the system works. Treated women were also given a promotional calendar to serve as a physical reminder on how to navigate the service and the benefits of the service. Women in the control group were not given any intervention. Given that the service was freely available to all women, they were in theory able to call the service at any time, but not given any encouragement or support to do so by the study team."

The UGX 10,000 were proposed by the Ugandan IRB. On the consent form, these were stated, but not as incentive to participate, but simply to "compensate you for the time it will take to complete the survey". Given that the surveys took up to two hours, this seemed fair – we did not observe any differential participation in the study (i.e. there were almost no refusals).

- Please specify the exclusion (withdrawal) criteria during the study period, which criteria refer to circumstances that necessitate the removal of participants after enrollment, such as the death of a close family member.

We did not have any such exclusion criteria during the study and have added this information to the Inclusion and Exclusion criteria section now, where we write:

"There were no exclusion criteria (or exclusions) during the study."

- Please specify the sample size and describe the method used for its calculation in the Methods section.

We have revised the Power Calculations section to directly explain how the sample size was derived:

"The study was powered to detect a 0.1 standard deviation (SD) difference in standardized knowledge scores (mean 0, SD 1) with power 0.9. Assuming an average cluster size of 30, a follow-up rate of 90% and a design effect of 1.3, this required 100 clusters per arm, and a total sample size of 6000 women."

- Please provide additional details on how participants used the Viamo platform. Clarify whether platform use was need-based and participant-driven or task-oriented as part of the intervention.

We have expanded to Intervention section to provide further details on this. The answer to your question is a mix of both – users could call at any time, but were also (in the treatment group only) encouraged to use the system:

"Treated women were encouraged to use the system whenever they had questions or wanted to know more about sexual and reproductive health. Viamo also employed targeted nudges, personalized campaigns and other outreach activities (such as release of new content), with the aim of maintaining and incentivizing usage rates (active calling into the Viamo Platform) among women in the treatment group (see Supplemental Materials S1 for an example)."

- You mentioned in the limitations that information leak or unintended use of the platform by control group participants may have occurred. Please clarify whether any measures were taken to limit access to the platform or prevent its use by the control group and, if so, describe these measures in the Methods section. In addition, indicate whether any promotional activities (such as social media or other communication channels) were used that might have increased awareness of the platform and led to its use among control group participants.

We were not able to prevent anybody from using the system, and state this clearly now in the revised Randomization section, where we write:

"Women in the control group were not given any intervention. Given that the service was freely available to all women, they were in theory able to call the service at any time, but not given any encouragement or support to do so by the study team."

We are not aware of any social media or other programs promoting this channel and would have known since we worked closely with Viamo on this project.

Pg. 7/26: Were there specific intervals between each measurement time point? If so, please specify these intervals.

The idea was to have 15 months of program exposure between the two surveys. We have clarified this now in the revised Methods section where we write:

"Baseline data was collected via face-to-face interview at each participants' home between December 6, 2022 and March 6, 2023. Upon completion of the baseline survey, treated women were introduced to the service by study staff. Endline data was collected approximately 15 months after recruitment, between May 8, 2024 and August 4, 2024 in two separate surveys."

- If the reproductive health questionnaire used in the study has a specific name or is a standardized tool recognized in the literature, please provide its official name and any relevant references.

This is now explained in the revised Methods section as follows:

"For reproductive health, we used a 15-item questionnaire of true/false statements (items listed in Supplemental Materials S2). This tool was based on previous studies conducted in sub-Saharan Africa [11] and pretested locally to make sure all items were suitable for the study population. To facilitate interpretation of regression results, we converted the raw scores (number of correct answers out of 15) into z-scores with mean zero and standard deviation one."

- The current description of study variables appears unclear and potentially confusing. The title refers to Sexual and Reproductive Health Knowledge, Behavior, and Outcomes. The explanation

provided under primary and secondary outcomes could clearly reflect the study objectives and variables if organized and presented systematically. For instance, the terms reproductive health "behavior" and "outcome" seem to overlap; if this is the case, consider removing one term from the title and text to maintain consistency. Otherwise, please provide a clear definition of each variable and explain how it is measured. Similarly, the term "sexual" may overlap with "reproductive health", as behaviors measured under "sexual behavior" might also fall under reproductive health outcomes. Therefore, please clarify this distinction or consider merging the two concepts where appropriate. A concise, organized, and precise description of variables and outcomes will improve understanding of the study's objectives and interpretation of results.

We are sorry for the confusion. "Sexual and reproductive health" is used as an umbrella term in the literature; we agree that it is not clear where the boundaries between "sexual" and "reproductive" are, but think this is not central to our question. For our project, we had a clear theory of change, going from knowledge to behavior and outcomes, and have tried to follow this in the way we present the results. As for the specific variables, we have tried to explain them in the revised Methods section, where we write:

"The two primary outcomes of the study were reproductive health knowledge and nutritional supplementation in pregnancy. For reproductive health, we used a 15-item questionnaire of true/false statements For nutrition knowledge, we analyzed a binary indicator for women (falsely) believing that herbs can cure anemia. For nutritional supplementation during pregnancy, we used self-reported iron supplementation as an outcome measure. Secondary outcomes included contraceptive knowledge (number of methods respondents are familiar with), contraceptive use (currently using any modern method), prenatal care uptake (any, number of visits), haemoglobin level (as a biomarker indicating actual iron intake), body mass index, and birth spacing.

For pregnancy related outcomes, we analyzed two separate sets of women: first, women reporting to be pregnant at the time of the endline survey (currently pregnant), and second, women who completed a pregnancy in the year preceding the endline survey (recently pregnant). For current pregnancies, we analyzed self-report iron supplementation as well as self-reported use of antenatal care services, and whether women indicate the pregnancy was planned. For recently completed pregnancies, we analyzed the same variables, but also analyzed pregnancy loss and low birth weight (reported weight at birth < 2.5kgs) as additional secondary outcomes. "

Pg. 9/26: Please describe the descriptive and inferential statistical tests used in the study, and specify the statistical methods applied for the presentation and interpretation of the tables.

We have clarified our main empirical model as follows:

"All primary models were estimated intent-to-treat, comparing outcomes between treated and control women at endline. For all outcomes, we estimated both unadjusted linear regression models (not including any covariates) and fully adjusted models, where we controlled for age, district, marital status, educational attainment and baseline knowledge variables. The main rationale for using linear regression models was to generate mean differences estimates that could be compared across the rather large set of outcome variables. To adjust for non-normal residuals, we used Huber's cluster-robust standard errors [15]. "

Pg. 11/26: Please mention p-values and place the asterisk indicators (*) next to the p-values only, and not next to other values. Consider this issue for all tables.

Apologies for the lack of clarity. We have edited the legends of all regression tables to clarify what the asterisks mean – the legend for Table 2 now says:

*"All point estimates displayed correspond to estimated mean differences in outcomes. Numbers in parentheses are cluster-robust standard errors. Asterisks indicate p-value range of estimated differences: *p-value<0.10, **p-value<0.05, ***p-value<0.01."*

We have added the same information to all other regression tables.

Pg. 13/26: In Table 4, you referred to "Beliefs that local herbs cure anemia"; however, this variable has not been mentioned in either the primary or secondary outcomes, nor in the measurement instruments described in the supplementary files. Please provide clarification on this issue.

Apologies for this omission: we have added this to the Methods section now:

"For nutrition knowledge, we analyzed a binary indicator for women (falsely) believing that herbs can cure anemia."

Pg. 15/26: Since Survey modality is reported at the end of the table, please refrain from repeating it in the outcome rows.

Good point – we have removed this information from the column heaers.

Pg. 15-20: Was a specific checklist or questionnaire used to collect the data presented in Tables 5–10? Please clarify this point. If data collection and evaluation were based on a checklist, please describe it in the relevant sections of the manuscript.

We did not use a checklist – all of these data were collected through a study tool developed for this project and implemented using tablets with the SurveyCTO software.

Pg. 15-19: The outcomes reported in Tables 5, 7, 8, and 9 may have been influenced by pregnancy and postpartum healthcare recommendations, medication use, or being under the supervision of a physician or nutritionist. Please clarify how these potential confounding factors were addressed in the analyses. If any strategies were implemented to control for them, please describe them explicitly; otherwise, please acknowledge this issue in the limitations section. In addition, it is recommended that the results for pregnant and non-pregnant women be reported separately in these tables.

We fully agree that these outcomes will be the results of many factors such as health care provider recommendations, but don't think this is a concern here since treatment is randomized – as long as the randomization worked (which we are very confident here due to the large number of clusters and individual-level randomization), confounding should not be an issue.
As for the subsamples, we have reported them separately in the tables.

- Not all results have been fully interpreted, and the interpretation provided is rather brief. Please expand the Discussion section to provide a more comprehensive interpretation of the study findings. For instance, the observed reduction in pregnancy loss and low birth weight could be interpreted in relation to the level of antenatal care received. In addition, for each outcome discussed, please contextualize the findings within the existing literature by comparing them with results from similar studies and discussing areas of consistency or discrepancy.

We have expanded the Discussion section a bit and tried to provide a bit more context, but were not able to say much more on the literature, since we could not find many other studies using a similar approach or technology – please let us know if there are any studies that we missed.

- Please critically assess the generalization of the study findings and clarify the extent to which the results can be applied to other populations or settings.

We have added the following paragraph to the Discussion:

"Even though a more selected sample clearly does not undermine the internal validity of the study (since randomization was done at the individual level), it is not clear whether the final set of women enrolled in the study is fully representative of the larger populations in the four study districts; it is also not clear if the four districts are fully representative of the country as a whole. Since the service is currently only available to Airtel users, this study simply focused on women with access to this service. Even though it seems plausible that similar effects could be found with other phone providers, it is not clear if the results of this study can be generalized to the country as a whole or to other countries."
