# Supplementary material for: Estimating the impact of Viamo call-in information services on sexual and reproductive health knowledge, behavior, and outcomes among women of reproductive age: A 2-arm open label randomized controlled trial in Uganda
Source: PLoS One. 2026 Feb 13;21(2):e0342327. doi: 10.1371/journal.pone.0342327 (PMC12904432; doi:10.1371/journal.pone.0342327)
Supplement: Attachment — Submitted filename: PONE-D-25-05733_reviewer1.docx [file pone.0342327.s004.docx]

PLOS ONE

Estimating the Knowledge and Health Impact of Call-in Information Services Serving

Vulnerable Populations: Results from a 2-Arm Open Label Randomized Controlled

Trial in Uganda

--Manuscript Draft--

| Manuscript Number: | PONE-D-25-05733 |
| --- | --- |
| Article Type: | Research Article |
| Full Title: | Estimating the Knowledge and Health Impact of Call-in Information Services Serving Vulnerable Populations: Results from a 2-Arm Open Label Randomized Controlled Trial in Uganda |
| Short Title: | Improving health knowledge through call-in information services |
| Corresponding Author: | Günther Fink  Swiss Tropical Institute: Schweizerisches Tropen- und Public Health-Institut  SWITZERLAND |
| Keywords: | health knowledge; call-in services; randomized trial; Uganda; Sexual health; Reproductive Health; nutrition. |
| Abstract: | Background  Despite substantial increases in access to schooling and to the internet, health information and knowledge remains limited among many vulnerable populations. Given the near universal cell-phone coverage in low- and middle-income countries, call-in services offer an interesting new platform to provide high quality information to targeted populations. In this paper, we assessed the impact of the Viamo 3-2-1 call-in Platform on health knowledge and behavior among women of reproductive age in Uganda.  Methods  We conducted a 2-arm open label randomized controlled trial involving 6,000 women, ages 18-49 years, with access to a simple feature cell phone in Uganda. Fifty enumeration areas were randomly selected in Kampala, Madi-Okollo, Rwampara and Katakwi districts. After completion of baseline, women were randomized with equal probability to treatment and control using a tablet-generated random number draw. Study staff introduced treated women to the call-in service and encouraged its use through various incentives and promotional messages. Primary outcomes were sexual and reproductive health knowledge scores as well as iron supplementation in pregnancy. Secondary outcomes included self-reported use of contraception, healthy diets, birth spacing and birth outcomes as well as hemoglobin levels and BMI. Intention to treat as well as per protocol estimates were used to assess program impact. Standard errors were corrected for the cluster-based sampling of women.  Results  A total of 6,011 women were enrolled into the study between December 6, 2022 and February 11, 2023; 3,052 were assigned to treatment, 2,959 used as controls. Endline data was collected between May 8, 2024 and August 4, 2024. Overall follow-up rates were 92% in both the treatment and the control group. Treatment increased average sexual and reproductive health knowledge by 0.07 standard deviations (95% CI [0.01, 0.11], p-value = 0.02) and increased the likelihood of taking iron supplements by 4% pts among recently pregnant women ([0.003, 0.08], p-value 0.04). Results for secondary outcomes were mixed, with positive changes for nutritional intake and family planning behaviors and intentions and no impact on hemoglobin levels and BMI. No adverse events or outcomes were reported.  Conclusions  The results of this trial suggest that access to call-in services can increase health knowledge and to some extent also health behaviours. Longer and more intensive program exposure may be needed to yield measurable changes in reproductive |

|  | outcomes and nutritional status. |
| --- | --- |
| Order of Authors: | Günther Fink |
|  | Branwen Nia Owen |
|  | Jan Will |
|  | Ilyena Kozain |
| Additional Information: |  |
| Question | Response |
| Financial Disclosure  Enter a financial disclosure statement that describes the sources of funding for the work included in this submission. Review the [submission guidelines](http://journals.plos.org/plosone/s/submission-guidelines#loc-financial-disclosure-statement) for detailed requirements. View published research articles from [PLOS ONE](https://journals.plos.org/plosone/) for specific examples.  This statement is required for submission and will appear in the published article if the submission is accepted. Please make sure it is accurate. | Yes |
| Funded studies Enter a statement with the following details: - Initials of the authors who received each award - Grant numbers awarded to each author - The full name of each funder - URL of each funder website - Did the sponsors or funders play any role in the study design, data collection and analysis, decision to publish, or preparation of the manuscript? |  |
| Did you receive funding for this work? |  |
| Please add funding details.   as follow-up to "Financial Disclosure  Enter a financial disclosure statement that describes the sources of funding for the work included in this submission. Review the [submission guidelines](http://journals.plos.org/plosone/s/submission-guidelines#loc-financial-disclosure-statement) for detailed requirements. View published research articles from [PLOS ONE](https://journals.plos.org/plosone/) for specific examples. | This project was funded through USAID DIV grant number 7200AA21FA00018 |

| This statement is required for submission and will appear in the published article if the submission is accepted. Please make sure it is accurate. |  |
| --- | --- |
| Funded studies Enter a statement with the following details: - Initials of the authors who received each award - Grant numbers awarded to each author - The full name of each funder - URL of each funder website - Did the sponsors or funders play any role in the study design, data collection and analysis, decision to publish, or preparation of the manuscript? |  |
| Did you receive funding for this work?" |  |
| Please select the country of your main research funder (please select carefully as in some cases this is used in fee calculation).   as follow-up to "Financial Disclosure  Enter a financial disclosure statement that describes the sources of funding for the work included in this submission. Review the [submission guidelines](http://journals.plos.org/plosone/s/submission-guidelines#loc-financial-disclosure-statement) for detailed requirements. View published research articles from [PLOS ONE](https://journals.plos.org/plosone/) for specific examples.  This statement is required for submission and will appear in the published article if the submission is accepted. Please make sure it is accurate. | UNITED STATES - US |

| Funded studies Enter a statement with the following details: - Initials of the authors who received each award - Grant numbers awarded to each author - The full name of each funder - URL of each funder website - Did the sponsors or funders play any role in the study design, data collection and analysis, decision to publish, or preparation of the manuscript? |  |
| --- | --- |
| Did you receive funding for this work?" |  |
| Competing Interests  Use the instructions below to enter a competing interest statement for this submission. On behalf of all authors, disclose any [competing interests](http://journals.plos.org/plosone/s/competing-interests) that could be perceived to bias this work—acknowledging all financial support and any other relevant financial or nonfinancial competing interests.  This statement is required for submission and will appear in the published article if the submission is accepted. Please make sure it is accurate and that any funding sources listed in your Funding Information later in the submission form are also declared in your Financial Disclosure statement.  View published research articles from [PLOS ONE](http://journals.plos.org/plosone/) for specific examples. | The first, second and third author declare no competing interest. The last author (IK) is currently employed by Viamo, the company operating the service analyzed. She was not involved in the data analysis and did not modify the way the results are presented or described in any way. |

| NO authors have competing interests Enter: The authors have declared that no competing interests exist. Authors with competing interests Enter competing interest details beginning with this statement: I have read the journal's policy and the authors of this manuscript have the following competing interests: [insert competing interests here] |  |
| --- | --- |
| * typeset |  |
| Ethics Statement  Enter an ethics statement for this submission. This statement is required if the study involved:   - Human participants - Human specimens or tissue - Vertebrate animals or cephalopods - Vertebrate embryos or tissues - Field research   Write "N/A" if the submission does not require an ethics statement.  General guidance is provided below. Consult the [submission guidelines](http://journals.plos.org/plosone/s/submission-guidelines#loc-guidelines-for-specific-study-types) for detailed instructions. Make sure that all information entered here is included in the Methods section of the manuscript. | Ethical clearance for the study was obtained from the Mildmay Uganda Research  Ethics Committee (MUREC-2022-124), the ethics committee for North-Western Switzerland (AO_2022-00034) as well as by the Innovations for Poverty Action internal review board ( #00006083). |

Format for specific study types

Human Subject Research (involving human

participants and/or tissue)

Give the name of the institutional review

board or ethics committee that approved the

study

•

Include the approval number and/or a

statement indicating approval of this

research

•

Indicate the form of consent obtained

(

written/oral) or the reason that consent was

not obtained (e.g. the data were analyzed

anonymously)

•

Animal Research (involving vertebrate

animals, embryos or tissues)

Provide the name of the Institutional Animal

Care and Use Committee (IACUC) or other

relevant ethics board that reviewed the

study protocol, and indicate whether they

approved this research or granted a formal

waiver of ethical approval

•

Include an approval number if one was

obtained

•

If the study involved

non-human primates

,

add

additional details

about animal welfare

and steps taken to ameliorate suffering

•

If anesthesia, euthanasia, or any kind of

animal sacrifice is part of the study, include

briefly which substances and/or methods

were applied

•

Field Research

Include the following details if this study

involves the collection of plant, animal, or

other materials from a natural setting:

Field permit number

•

Name of the institution or relevant body that

granted permission

•

Data Availability

Authors are required to make all data

underlying the findings described fully

available, without restriction, and from the

time of publication. PLOS allows rare

exceptions to address legal and ethical

concerns. See the

[PLOS Data Polic](http://journals.plos.org/plosone/s/data-availability)

[y](http://journals.plos.org/plosone/s/data-availability)

and

[FA](http://journals.plos.org/plosone/s/data-availability#loc-faqs-for-data-policy)

[Q](http://journals.plos.org/plosone/s/data-availability#loc-faqs-for-data-policy)

for detailed information.

Yes - all data are fully available without restriction

| A Data Availability Statement describing where the data can be found is required at submission. Your answers to this question constitute the Data Availability Statement and will be published in the article, if accepted. |  |
| --- | --- |
| Important: Stating ‘data available on request from the author’ is not sufficient. If your data are only available upon request, select ‘No’ for the first question and explain your exceptional situation in the text box. |  |
| Do the authors confirm that all data underlying the findings described in their manuscript are fully available without restriction? |  |
| Describe where the data may be found in full sentences. If you are copying our sample text, replace any instances of XXX with the appropriate details.   - If the data are held or will be held in a public repository, include URLs, accession numbers or DOIs. If this information will only be available after acceptance, indicate this by ticking the box below. For example: All XXX files   are available from the XXX database (accession number(s) XXX, XXX.).   - If the data are all contained within the manuscript and/or Supporting Information files, enter the following: All relevant data are within the manuscript and its Supporting Information files. - If neither of these applies but you are able to provide details of access elsewhere, with or without limitations, please do so. For example:   Data cannot be shared publicly because of [XXX]. Data are available from the XXX Institutional Data Access / Ethics Committee (contact via XXX) for researchers who meet the criteria for access to confidential data.  The data underlying the results presented in the study are available from (include the name of the third party | The data in this study will be posted with the article on PLOS One. |

| and contact information or URL).  • This text is appropriate if the data are owned by a third party and authors do not have permission to share the data.  * typeset |  |
| --- | --- |
| Additional data availability information: | Tick here if the URLs/accession numbers/DOIs will be available only after acceptance of the manuscript for publication so that we can ensure their inclusion before publication. |

Manuscript	[Click here to access/download;Manuscript;Manuscript 11 Feb](https://www2.cloud.editorialmanager.com/pone/download.aspx?id=39274518&guid=c88ff9d9-4771-4395-a324-5c8bd136f6a4&scheme=1)

[2025.docx](https://www2.cloud.editorialmanager.com/pone/download.aspx?id=39274518&guid=c88ff9d9-4771-4395-a324-5c8bd136f6a4&scheme=1)

**Title:** Estimating the Knowledge and Health Impact of Call-in Information Services Serving

Vulnerable Populations: Results from a 2-Arm Open Label Randomized Controlled Trial in Uganda

**Authors:** Günther Fink^1,2*^, Branwen Nia Owen^1,2^, Jan Will^3^, Ilyena Kozain^4^

**Author Affiliations:**

1 University of Basel, Basel, Switzerland
2 Swiss Tropical and Public Health Institute, Allschwil, Switzerland
3 Innovations for Poverty Action, Kampala, Uganda
4 Viamo, Kampala, Uganda

*** Corresponding author:** Swiss Tropical and Public Health Institute, Kreuzstrasse 2, 4123 Allschwil, Switzerland. Email: guenther.fink@swisstph.ch.

# Abstract

**Background*.*** Despite substantial increases in access to schooling and to the internet, health information and knowledge remains limited among many vulnerable populations. Given the near universal cellphone coverage in low- and middle-income countries, call-in services offer an interesting new platform to provide high quality information to targeted populations. In this paper, we assessed the impact of the Viamo 3-2-1 call-in Platform on health knowledge and behavior among women of reproductive age in Uganda.

**Methods*.*** We conducted a 2-arm open label randomized controlled trial involving 6,000 women, ages 18-49 years, with access to a simple feature cell phone in Uganda. Fifty enumeration areas were randomly selected in Kampala, Madi-Okollo, Rwampara and Katakwi districts. After completion of baseline, women were randomized with equal probability to treatment and control using a tabletgenerated random number draw. Study staff introduced treated women to the call-in service and encouraged its use through various incentives and promotional messages. Primary outcomes were sexual and reproductive health knowledge scores as well as iron supplementation in pregnancy. Secondary outcomes included self-reported use of contraception, healthy diets, birth spacing and birth outcomes as well as hemoglobin levels and BMI. Intention to treat as well as per protocol estimates were used to assess program impact. Standard errors were corrected for the cluster-based sampling of women.

**Results.** A total of 6,011 women were enrolled into the study between December 6, 2022 and February 11, 2023; 3,052 were assigned to treatment, 2,959 used as controls. Endline data was collected between May 8, 2024 and August 4, 2024. Overall follow-up rates were 92% in both the treatment and the control group. Treatment increased average sexual and reproductive health knowledge by 7% standard deviations (95% CI [0.01, 0.11], p-value = 0.02) and increased the likelihood of taking iron supplements by 4% pts among recently pregnant women (95% CI [0.003, 0.08], p-value=0.04). Results for secondary outcomes were mixed, with positive changes for nutritional intake and family planning behaviors and intentions and no impact on hemoglobin levels and BMI. No adverse events or outcomes were reported.

***Conclusions.*** The results of this trial suggest that access to call-in services can increase health knowledge and to some extent also health behaviours. Longer and more intensive program exposure may be needed to yield measurable changes in reproductive outcomes and nutritional status.

## Introduction

In low- and middle-income countries (LMIC), access to timely and accurate information in national languages is often limited, particularly among the poor. Whereas the internet is a common source of information in many countries, only 10% of Ugandans, and 36% of SubSaharan Africans were estimated to use the internet in 2021; far lower than the global average of 63% [1]. This lack of information access can contribute to poor health decisions, such as inadequate prenatal care and ineffective family planning methods [2].

Public health messaging in LMICs is often disseminated through mass media channels such as radio messages and billboards. These channels have proven to be effective at reaching large numbers of people [3] and increasing knowledge, but have had an insufficient impact on changing behaviors in many areas of public health [4, 5]. Mass media campaigns often fail to create behavior change because, in part, people cannot access the information at a moment of need. Cell phones, abundant in Uganda [6], offer opportunities to address this gap, as information can be accessed at any time, from anywhere. Recent evidence suggests that interventions to improve reproductive health knowledge through easy access technologies can effectively improve reproductive health knowledge and use of modern contraception [7-9].

Viamo’s platform was created with the objective of addressing the need for accessible and timely health information. Through an easy to remember dial-in number, callers can obtain upto-date news as well as key information on topics related to nutrition, maternal and child health, and sexual and reproductive health. The platform is free of charge, and currently has over 4 million users in Uganda, and 42 million users in LMICs globally. With the aim of rigorously assessing the impact of the Viamo’s call-in service on sexual and reproductive health knowledge and related behavior, we conducted a randomized controlled trial (RCT) with women of reproductive age across four districts of Uganda.

**Methods**

## Study Design

This study was designed as a two-arm, non-blinded, individual-level randomised controlled trial focusing on women’s nutrition and reproductive health. Given that the main intervention – a phone-based information service – was equally available to both arms, we used an encouragement design, actively introducing women in the treated arm to the service and encouraging them to use it throughout the study period. Blinding of the intervention was not possible given the nature of the program.

## Study Setting and Participant Recruitment

This study was conducted in four districts of Uganda: Kampala, Katakwi, Madi-Okollo and Rwampara. These four districts were purposely chosen to cover all major regions of Uganda. To recruit women within these four districts, a two-stage random sampling process was followed: first, a list of all enumeration areas in each district was obtained, and 50 enumeration areas were randomly chosen using a simple random number draw created with the Stata statistical software package. In rural areas, enumeration areas corresponded to villages, and in urban areas, to residential blocks. All households within each selected enumeration area were visited to identify eligible women. Women living in selected enumeration areas were eligible for inclusion in the study if they were aged 18-49 years and had access to an Airtel (a mobile network operator) phone number within their household. Women were ineligible if they had used the Viamo platform previously, they planned to move outside of the study area within 12 months of baseline or if they did not speak one of the six languages available on the platform:

Ateso, English, Luganda, Lugbara, Luo or Runyakitara.

In a second and final sampling step, we selected individual women for trial inclusion from a complete list of women in the enumeration unit. Given the explicit focus on antenatal care, family planning and post-partum, all women who were pregnant during the listing were automatically invited to join the study; non-pregnant women were randomly selected for the study until the 45 women per cluster target was reached. In clusters with less than 45 women, all women were invited to participate in the study. Figure 1 shows the spatial location of the study districts within Uganda.

**Figure 1:** Spatial distribution of study districts (orange shapes) and study clusters (small circles)

## Intervention

In Uganda, the Viamo platform is available via voice and text channels on all mobile phones with an Airtel number and offers a wide variety of health and other information to all interested callers. To ensure increased service utilization among women randomly selected for the treatment, all treated women in the sample received:

1. A personalized introduction and mock call into platform (at time of baseline interview)
2. A promotional calendar to serve as a physical reminder on how to navigate the service and the benefits of the service (at time of baseline interview)
3. Continued encouragement to use the service in the form of text messages and outbound calls, incentivizing women to call the service.

The original 60+ sexual and reproductive, maternal and neonatal health and nutrition key messages launched at the inception of the project were developed in conjunction with subject experts, end users, communication specialists, and the Ugandan Ministry of Health (MOH). Throughout the implementation period, the key messages (2-4 mins of dialogue) were transformed into stylized dynamic content (7-45 mins), to reiterate critical concepts for enhanced learning and be able to promote new content. The release of DIV-dynamic content formats (dramas, talk shows, and celebrity interviews) was staggered and began in August 2022.

Following enrollment in the study (December 2022 - March 2023), Viamo employed targeted nudges, personalized campaigns and other outreach activities (such as release of new content), with the aim of maintaining and incentivizing usage rates (active calling into the Viamo Platform) among women in the treatment group (see Supplemental Materials Part 1 for an example).

## Study Procedures

Study staff visited selected women at their homes, introduced the study and invited them to participate. The consent form was read aloud and explained when necessary. Literate women signed the consent form and illiterate women marked the form with a thumbprint, and a literate witness was asked to sign on their behalf.

Participant recruitment and baseline data collection were done in person at each participants’ home between December 6, 2022 and March 6, 2023. Upon their informed consent, participated completed the baseline survey. After the survey, women randomly selected for the treatment were introduced to the service by study staff. Endline data was collected between May 8, 2024 and August 4, 2024 in two separate surveys; a phone survey and a face-to-face in-person survey (see Supplementary Table 1 for dates by district, and Supplementary Table 2 for data collected by phone and in-person interview at endline). During all three surveys, the interviewer recorded responses on a tablet using the SurveyCTO software. Data collectors received training and on-going support and monitoring to ensure data quality. Participants received UGX10,000 (~USD $2.77) of phone credit as a token of gratitude after each survey interview.

Surveys collected data on socio-demographic characteristics, knowledge and use of the Viamo platform, reproductive health knowledge, contraceptive use, pregnancy planning and uptake of antenatal care, pregnancy outcomes, diet and exercise behaviours and select biomarkers.

## Randomization

Upon completion of the baseline survey, we randomised arm allocation at the individual level based on a simple number draw generated by the SurveyCTO software. At the end of the survey, the software generated a random number from a uniform distribution [0,1] and allocated all draws > 0.5 to treatment.

## Power Calculations

The study was powered to detect a 0.1 standard deviation (SD) difference in standardized knowledge scores (mean 0, SD 1) with power 0.9, assuming an average cluster size of 30, a follow-up rate of 90% and a design effect of 1.3. This effect size was based on an anticipated knowledge score difference of 0.2 SD among compliers (system users), and an average compliance of 10% in the control, and 60% in the treatment arm (a net compliance/usage differential of 50%).

## Primary and secondary outcomes

The two primary outcomes of the study were reproductive health knowledge and nutritional supplementation in pregnancy. For reproductive health, we used a 15-item questionnaire of true/false statements (items listed in Supplemental Materials Part 2), which we normalized to a standard normal variable with mean zero and standard deviation one. For antenatal care, we used self-reported iron supplementation as an outcome measure. Secondary outcomes included contraceptive use, prenatal care uptake, hemoglobin level (as a biomarker indicating actual iron intake), body mass index, and birth spacing.

For pregnancy related outcomes, we analyzed two separate sets of women: first, women reporting to be pregnant at the time of the endline survey (currently pregnant), and second, women who completed a pregnancy in the year preceding the endline survey (recently pregnant). For current pregnancies, we analyzed iron supplementation, use of antenatal care services, and whether women indicate the pregnancy was planned. For recently completed pregnancies, we analysed the same variables, but also analysed pregnancy loss and low birth weight births as additional secondary outcomes.

## Statistical Analysis

We began our analysis with a comparison of baseline characteristics across arms, as well as a comparison of follow-up rates to assess potential selective attrition. All primary models were estimated intent-to-treat, comparing outcomes between treated and control women at endline. For all outcomes, we estimated both unadjusted models (not including any covariates) and fully adjusted models, where we controlled for age, district, marital status, educational attainment and baseline knowledge variables. We used forest plots to illustrate item level changes for aggregated reproductive health knowledge and contraceptive use scores. In an exploratory analysis, we also estimated per protocol models and stratified models. Per protocol models were restricted to compliant women: specifically, we included only women in the control group who had never called the service, and women in the treatment group who had called the service at least five times. To investigate differential impact on subgroups, we estimated stratified models by age group (<30, 30+), educational attainment group (primary or less, secondary, higher) and district.

## Study registration and ethical clearance

The trial was registered on June 03, 2022 at [https://www.socialscienceregistry.org/trials/9544.](https://www.socialscienceregistry.org/trials/9544) Ethical clearance for the study was obtained from the Mildmay Uganda Research Ethics Committee (MUREC-2022-124), the ethics committee for North-Western Switzerland (AO_2022-00034) as well as by the Innovations for Poverty Action internal review board ( #00006083).

## Results

Of 54,259 women screened, 29% (n=15,532) were eligible to participate in the study (Figure 2). The most common reason for ineligibility was not having access to an Airtel number. A total of 6,011 women completed the baseline survey and were randomised to the control (n=3,052) or the intervention arm (n=2,959). Follow-up rates (completing either the phone or the in person survey or both) were 92% in both arms. At endline, 87% (n=2,678) of control arm participants, and 86% (n=2,555) of intervention arm participants completed the phone survey. Slightly fewer participants completed the in-person survey (81% of both arms). The most common reason for loss to follow were participants being unreachable by phone, and participants having moved house, in the phone survey and in-person survey, respectively.

**Figure 2:** Consort Trial participant flow chart.

*Figure 2 Notes*: ^1^ Ateso, English, Luganda, Lugbara, Luo or Runyakitara; ^2^ Number out of service, phone being switched off, or calls unanswered. ^3^ Household visited at baseline was not found again. ^4^ Ill, intoxicated, or deceased

Table 1 shows descriptive statistics for the study sample. Over half were under 30 years old (53%), and a majority were currently married (58%). Twelve percent (12%) of subjects were recruited in Madi-Okollo, 21% in Katakwi, 32% in Rwampara and 35% in Kampala. Fewer than half of women lived in a household with piped water (47%) and very few had health insurance (2%), but most owned their own phone (84%). Most had previously been pregnant (88%) and nearly a seventh were currently pregnant (14%). The trial arms were well-balanced, with all characteristics very similar across arms (Table 1). Characteristics of the sample followed-up at endline did not differ systematically from the whole sample at baseline (Supplementary Tables 3-6).

## Table 1: Sample characteristics overall and by study arm

|  | Overall (n=6,011) | Control (n=3,052) | Treatment (n=2,959) |
| --- | --- | --- | --- |
| *Characteristic* | *N (%)* | *N (%)* | *N (%)* |
| Age 18-29 | 3,159 (53%) | 1,609 (53%) | 1,550 (52%) |
| Age 30-39 | 1,913 (32%) | 979 (32%) | 934 (32%) |
| Age 40+ | 939 (16%) | 464 (15%) | 475 (16%) |
| Currently married | 3,503 (58%) | 1,756 (58%) | 1,747 (59%) |
| No education | 159 (2.6%) | 95 (3.1%) | 64 (2.2%) |
| Primary education | 3,173 (53%) | 1,640 (54%) | 1,533 (52%) |
| Secondary or higher education | 2,611 (43%) | 1,285 (42%) | 1,326 (45%) |
| Education: other | 68 (1.1%) | 32 (1.0%) | 36 (1.2%) |
| Speaks English | 1,716 (29%) | 862 (28%) | 854 (29%) |
| Kampala | 2,081 (35%) | 1,071 (35%) | 1,010 (34%) |
| Katakwi district | 1,256 (21%) | 619 (20%) | 637 (22%) |
| Madi-Okollo district | 741 (12%) | 385 (13%) | 356 (12%) |
| Rwampara district | 1,933 (32%) | 977 (32%) | 956 (32%) |
| Household has piped water | 2,799 (47%) | 1,431 (47%) | 1,368 (46%) |
| Household has health insurance | 100 (1.7%) | 45 (1.5%) | 55 (1.9%) |
| Woman has her own phone | 5,056 (84%) | 2,527 (83%) | 2,529 (85%) |
| Currently pregnant | 867 (14%) | 441 (14%) | 426 (14%) |
| Previously pregnant (ever) | 5,275 (88%) | 2,683 (88%) | 2,592 (88%) |

Table 2 summarizes overall compliance with treatment and platform use. Half of women in the control group indicated at endline that they had heard of the Viamo platform compared to 96% in the intervention arm. 20% of women in the control arm reported to have ever used the platform, and 7% reported to have used it in the last month. In the treatment arm, the same was true for 84% and 42% respectively. On average, subjects in the control arm reported four calls to the platform, women in the treatment arm reported an additional 18 calls on average. These numbers also align nicely with the Viamo platform metrics, which recorded an average of four calls for the control group, and an additional 31 calls among women in the treatment group (suggesting that on average women under-reported the number of actual calls they made). In a given month, a treatment user listened to an average of approximately 38 minutes of content on the platform

## Table 2: Treatment Compliance

| Outcome | Heard of the platform | Ever used the platform | Used the platform in the last month | Total number of self-  reported calls to platform ^a)^ | Total number of registered calls to platform |
| --- | --- | --- | --- | --- | --- |
| Treatment effect | 0.459*** | 0.642*** | 0.346*** | 18.25*** | 31.41*** |
|  | (0.0192) | (0.0143) | (0.0129) | (0.471) | (0.958) |
| Control group average | 0.499 | 0.499 | 0.0709 | 4.075 | 4.012 |
| Observations | 5,233 | 5,233 | 5,233 | 5,233 | 5,233 |
| R-squared | 0.262 | 0.412 | 0.164 | 0.268 | 0.271 |

Notes: *p-value<0.10, **p-value<0.05, ***p-value<0.01, a) Self-reported number of calls were top-coded at 51 calls.

Table 3 provides an overview over the most popular content. The most popular single item was the local weather forecast, followed by information on food and general news. On average,

71% of the content listened to by women in the treatment group accessing the platform (n=2,825) was content specifically related to maternal health, sexual and reproductive health, family planning, or nutrition. For the control group members who accessed the platform (n=1,024) only 20% listened to the same thematically relevant content for this study.

**Table 3.** Top ten most popular messages listened to by treatment group on the platform (Dec 2023 - July 2024)

Total

Times	Times	C2L No	Message Title	Key Themes	Number of

Reached	Listened	Ratio

Listeners

| 1 | Dynamic daily weather report weather 14140 | 14140 | 2140 | 100% |
| --- | --- | --- | --- | --- |
| 2 | health, To learn about what food keeps us nutrition,  healthy anemia 8858 | 6710 | 1753 | 89% |
| 3 | Dynamic daily news bulletin news 5959 | 4488 | 1046 | 86% |
| 4 | health,  To know what a healthy weight is nutrition, BMI 4335 | 3585 | 1310 | 92% |
| 5 | To learn more about cervical cancer SRH 4056 | 3417 | 1179 | 91% |
| 6 | health,  What is folic acid and why take it? pregnancy 4006 | 3177 | 1208 | 89% |
| 7 | What is sex? puberty, SRH 3852 | 3500 | 1513 | 95% |
| 8 | DIV Drama Episode 1: “Family  planning for a better future” FP, SRH 3675 | 2053 | 633 | 77% |
| 9 | To know the four ways to improve SRH,  your chance for a healthy pregnancy pregnancy 3660 | 2205 | 1453 | 78% |
| 10 | nutrition,  To learn how iron keeps us strong anemia 3558 | 2950 | 1092 | 89% |
| *A listener is defined as someone who has listened to at least 75% of a key message. | | |  |  |
| *C2L (caller-to-listener ratio) defines the % completion listening | | | rate of | a message. |

BMI=body mass index, FP=family planning, SRH=sexual and reproductive health

Table 4 shows estimated impact on knowledge. We find a 0.07 SD increase in the primary knowledge outcome score in unadjusted models, and an increase of 0.06 SDs in fully adjusted models. Treatment also increased the average number of reproductive methods women reported to be familiar with by 0.11 points (implying that one in 10 women know one additional method). For nutrition, the treatment reduced the false belief that local herbs could cure anemia by 3 % pts.

**Table 4:** Impact on Knowledge

| Outcome | Reproductive health knowledge z-score | Beliefs that local herbs cure anemia | Number of contraceptive methods familiar with |
| --- | --- | --- | --- |
| Treatment effect | 0.0708*** | -0.0371** | 0.116** |
| (unadjusted) | (0.0265) | (0.0152) | (0.0461) |
| Adjusted treatment effect | 0.0591** | -0.0328** | 0.0941** |
|  | (0.0252) | (0.0148) | (0.0455) |
| Control group average | 9.742 | 0.406 | 10.33 |
| Survey modality | In person | In person | Phone |

Observations	4,877	4,877	5,233

*Notes*: Table shows estimated impact on primary outcomes. Coefficients displayed are mean differences with standard errors in parentheses. Adjusted models control for age group (age < 20, age 20-34 (ref), age >=35), educational attainment, district, baseline family planning knowledge, baseline hemoglobin level and baseline body mass index. Standard errors were clustered at the enumeration area level. *p-value<0.10, **p-value<0.05, ***pvalue<0.01,

Figure 3 shows item-specific changes in sexual and reproductive health knowledge. Largest improvements were observed for myths regarding male condoms causing infertility and postsex urination preventing pregnancy; small improvements were seen for most questions.

**Figure 3:** Item-level change in knowledge

*Figure 3 notes:* each point/square on the graphic represents the estimated coefficient obtained from regressing a binary indicator of giving a correct answer to the specific question on treatment - lines illustrate 95% confidence intervals.

Supplemental Materials Figure SF 1 further illustrates the observed changes in knowledge for the item with the largest absolute change in knowledge score. Supplemental Materials Table S2 shows the proportion of women answering each item correctly at baseline and endline by treatment status for all items investigated.

Table 5 summarizes the estimated impact on sexual behaviour as reported in person as well as on the phone. The two survey modalities yielded consistent results with 40% of women using modern contraception and the intervention resulting in an insignificant additional 2% of women using modern contraception. Positive impacts were found for the number of methods ever used (+0.14 methods in adjusted models) and the intention to use modern contraception in the future (+ 5% pts).

**Table 5:** Impact on self-reported sexual behaviour

| Outcome | Currently uses any  contraceptio n (in person  interview)  a) | Currently uses  modern  contracept ion (in person report  interview)  a) | Can show  contrac  eption to  intervie wer | Number of methods  ever used  (phone) | Currently uses any  contracept  ion (phone  interview)  a) | Currentl y uses  modern  contrace ption  (phone)a  ) | Plans to use modern  contraception in the future |
| --- | --- | --- | --- | --- | --- | --- | --- |
| Treatment effect | 0.0162 | 0.0193 | 0.00320 | 0.164*** | 0.0189 | 0.0218 | 0.050*** |
| (unadjusted) | (0.0147) | (0.0137) | (0.0066  7) | (0.0449) | (0.0143) | (0.0138) | (0.0124) |
| Adjusted treatment effect | 0.0157 | 0.0198 | 0.00384 | 0.140*** | 0.0162 | 0.0192 | 0.0468*** |
|  | (0.0145) | (0.0134) | (0.0063 8) | (0.0410) | (0.0138) | (0.0133) | (0.0118) |
| Control group average | 0.455 | 0.396 | 0.0718 | 2.956 | 0.474 | 0.396 | 0.396 |
| Survey modality | In person | In person | In person | Phone | Phone | Phone | Phone |
| Observations | 4,877 | 4,877 | 4,877 | 5,233 | 5,233 | 5,233 | 5,233 |

Notes: Coefficients displayed are mean differences with standard errors in parentheses. Adjusted models control for age group (age < 20, age 20-34 (ref), age >=35), educational attainment, district, baseline family planning knowledge, baseline hemoglobin level and baseline body mass index. Standard errors were clustered at the enumeration area level. *p-value<0.10, **p-value<0.05, ***p-value<0.01. a) The full list of contraceptives is shown in Supplemental Materials Section 1. Contraceptives not considered as “modern” are beads/calendar method, withdrawal and breastfeeding.

Figure 4 provides further details on current use of contraceptive methods. The intervention increased the use of implants by approximately 2 % pts, and lowered the use of beads (calendar method) and injectables marginally, resulting in the small insignificant overall change seen in Table 5.

**Figure 4:** Estimated impact on current contraceptive use

Figure notes: each point/square on the graphic represents the estimated coefficient obtained from regressing a binary indicator of having used the method in question on treatment - lines illustrate 95% confidence intervals.

Table 6 shows estimated impact on prenatal care. A total of 398 current pregnancies, as well as 653 pregnancies completed between baseline and endline were analysed. No differences were found in antenatal care attendance or deworming. Treatment increased the likelihood of taking iron in current pregnancies by 8% pts and the likelihood of taking supplements in recently completed pregnancies by 4% pts [0.3,7.7].

**Table 6:** Impact on Prenatal Care

Antenata	Recent

Attende	Recent

Currentl	l care for	Recent	pregnanc

d	Dewormi	pregnan

Outcome	y taking	recent	pregnanc	y:

antenatal ng cy >=4 iron pregnan y: iron dewormi

care	visits

cy	ng

| Treatment effect | 0.0593 | 0.0845* | 0.0167 | 0.00955 | 0.0229 | 0.0361* | 0.0374 |
| --- | --- | --- | --- | --- | --- | --- | --- |
| (unadjusted) | (0.0449) | (0.0431) | (0.0459) | (0.0161) | (0.0414) | (0.0189) | (0.0314) |
|  |  |  |  |  |  |  |  |
| Adjusted treatment effect | 0.0453 | 0.0758* | 0.00757 | 0.0156 | 0.0209 | 0.040** | 0.0369 |
|  | (0.0468) | (0.0456) | (0.0483) | (0.0158) | (0.0412) | (0.0189) | (0.0310) |
|  |  |  |  |  |  |  |  |
| Control group average | 0.621 | 0.605 | 0.451 | 0.958 | 0.454 | 0.901 | 0.808 |

Sample	Women currently pregnant	Women completing pregnancy last year

Survey modality	In person

Observations	398	398	398	653	619	653	647

*Notes:* Coefficients displayed are mean differences with standard errors in parentheses. Adjusted models control for age group (age < 20, age 20-34 (ref), age >=35), educational attainment, district, baseline family planning knowledge, baseline hemoglobin level and baseline body mass index. Standard errors were clustered at the enumeration area level. *p-value<0.10, **p-value<0.05, ***p-value<0.01

Table 7 shows estimated impact on other behaviour. The intervention increased women’s efforts to eat healthy by 4% pts; interestingly, these efforts were generally not linked to losing weight; on average, treated women were 2.5% less likely to attempt for weight loss, and 3% pts more likely to try to gain weight. In terms of food intake, the treatment appears to have increased the frequency of fruit and vegetable consumption, consistent with the results on healthier diets. This aligns with consistent messaging on increasing healthy habits such as regular activity, the inclusion of nutritious and iron rich foods, and maintaining a healthy body weight.

**Table 7:** Impact on Other Health Behaviours

Aims for	Tried to	Tried to	Times ate	Times	Currentl

Times	Exercise

Outcome	health	lose	gain	vegetable	ate	y taking

at fruit	d diet	weight	weight	s	protein	iron

|  |  |  |  |  |  |  | supplem ents |  |
| --- | --- | --- | --- | --- | --- | --- | --- | --- |
| Treated | 0.0407*** | -0.025** | 0.0355*** | 0.0686** | 0.0484** | -0.00060 | 0.0156 | 0.0154 |
| (Unadjusted) | (0.0144) | (0.0108) | (0.0128) | (0.0346) | (0.0245) | (0.0183) | (0.00988) | (0.0124) |
| Adjusted treatment effect | 0.0312** | -0.025** | 0.0316** | 0.0583* | 0.0371* | 0.00202 | 0.0174* | 0.0119 |
|  | (0.0123) | (0.0106) | (0.0126) | (0.0298) | (0.0213) | (0.0172) | (0.00965) | (0.0118) |
| Control group average | 0.552 | 0.255 | 0.242 | 1.335 | 1.332 | 1.760 | 0.136 | 0.335 |
| Survey modality |  |  |  | Phone survey | |  |  |  |
| Observations | 5,233 | 5,233 | 5,233 | 5,233 5,232 | | 5,233 | 5,233 | 5,233 |

Notes: Coefficients displayed are mean differences with standard errors in parentheses. Adjusted models control for age group (age < 20, age 20-34 (ref), age >=35), educational attainment, district, baseline family planning knowledge, baseline hemoglobin level and baseline body mass index. Standard errors were clustered at the enumeration area level. *p-value<0.10, **p-value<0.05, ***p-value<0.01.

Table 8 shows estimated impacts on pregnancy and birth outcomes. 41% of currently pregnant women in the control group reported that the current pregnancy was not planned; this proportion was 5% pts smaller in the treatment group, but not significantly different. Unplanned pregnancies in the past were slightly more common among treated women (but not statistically different). The proportion of previous pregnancies lost as well as the proportion of most recent births born with low birthweight (< 2500 grams) were substantially lower in the treated group – these differences were however not significant statistically due to the small number of observations here.

**Table 8:** Impact on pregnancy and birth outcomes

Pregnancy	Birth	Previous	Previous	Previous

Outcome was spacing < pregnancy pregnancy pregnancy unplanned 2 years unplanned low lost

|  |  |  |  | | birthweigh t |  |
| --- | --- | --- | --- | --- | --- | --- |
| Treatment effect | -0.0605 | 0.0519 | 0.0260 | | -0.0143 | -0.0301 |
| (unadjusted) | (0.0481) | (0.0546) | (0.0341) | | (0.0246) | (0.0216) |
| Adjusted treatment effect | -0.0531 | 0.0496 | 0.0215 | | -0.0157 | -0.0305 |
|  | (0.0495) | (0.0516) | (0.0337) | | (0.0245) | (0.0209) |
| Control group average | 0.410 | 0.372 | 0.261 | | 0.0888 | 0.0384 |
| Survey modality |  |  | In person | |  |  |
| Sample | Currently pregnant | | | Completed pregnancy recently | | |
| Observations | 398 371 | | | 653 565 653 | | |

*Notes:* Coefficients displayed are mean differences with standard errors in parentheses. Adjusted models control for age group (age < 20, age 20-34 (ref), age >=35), educational attainment, district, baseline family planning knowledge, baseline hemoglobin level and baseline body mass index. Standard errors were clustered at the enumeration area level. *p-value<0.10, **p-value<0.05, ***p-value<0.01

Table 9 shows estimated impact on biomarkers. While we see some lower rates of anemia among treated women who currently are, or recently were pregnant (Table 9, column 2), these differences are not statistically significant. No changes were found for BMI overall, or any of the categories analysed (underweight, overweight, obese).

**Table 9:** Impact on biomarkers

Overweig

Anemic	Anemic	Body	Obese	Underweig

ht (BMI

Outcome	(Hg<12	(Hg<12	mass	(BMI	ht (BMI

>=25, g/dL)	g/dL)	index	>=30)	<18.5)

<30)

Treatment effect	0.00745	-0.0495	0.183	0.00277	0.0150	0.0120 (unadjusted)	(0.0129)	(0.0315)	(0.165)	(0.0126)	(0.0106)	(0.00826)

Adjusted treatment effect	0.0111	-0.0468	0.0922	0.000613	0.0102	0.0140*

(0.0127)	(0.0311)	(0.111)	(0.0121)	(0.00831)	(0.00808)

| Control group average | 0.23 | 0.23 | 24.89 | 0.26 | 0.15 | 0.08 |
| --- | --- | --- | --- | --- | --- | --- |
| Sample | All | Current or recent pregnancy | All | All | All | All |
| Observations | 4,664 | 992 | 4,852 | 4,852 | 4,852 | 4,852 |

Coefficients displayed are mean differences with standard errors in parentheses. Adjusted models control for age group (age < 20, age 20-34 (ref), age >=35), educational attainment, district, baseline family planning knowledge, baseline hemoglobin level and baseline body mass index. Standard errors were clustered at the enumeration area level. *p-value<0.10, **p-value<0.05, ***p-value<0.01.

Table 10 shows the result of the per protocol analysis, where we compare control women who had never called the platform to treated women who had called the platform at least five times.. As expected, estimated impacts on knowledge scores are higher among compliers; however, these estimates become substantially smaller again when we adjust for women’s differences in education and reproductive health knowledge at baseline.

**Table 10:** Per protocol analysis

Currently

SRH	Anemic

uses	Uses iron	Used iron	Lost

Outcome	Knowledge	Anemic	post

modern	supplements	supplements	pregnancy

z-score	pregnancy

method

Treatment effect	0.127***	0.0409**	0.0571	0.000959	-0.00654	0.00586	-0.0496

(Unadjusted)	(0.0332)	(0.0159)	(0.0515)	(0.0102)	(0.00671)	(0.0147)	(0.0345) Adjusted treatment 0.0650** 0.0206 0.0575 0.0117 -0.00911 0.0168 0.0168 effect (0.0308) (0.0155) (0.0523) (0.00954) (0.00668) (0.0144) (0.0144)

Control group average	-0.0613	0.374	0.643	0.915	0.0386	0.229	0.389

Pregnant

All	All	Pregnant last year	All	Pregnant Sample	now

Restriction	Compliers only

Observations	3,561	3,561	292	3,313	3,313	3,400	727

*Only women in the control group who never called the service, and women in the treatment group who called the service at least five times are included in this table. Coefficients displayed are mean differences with standard errors in parentheses. Adjusted models control for age group (age < 20, age 20-34 (ref), age >=35), educational attainment, district, baseline family planning knowledge, baseline hemoglobin level and baseline body mass index. Standard errors were clustered at the enumeration area level. *p-value<0.10, **p-value<0.05, ***pvalue<0.01. SRH=sexual and reproductive health.

## Stratified Results

Table 11 shows stratified impact results by age and educational attainment group, as well as by district. For age, we find the weakest effects among women under age 20, and largest impacts among women 35 years and older. Educational level seemed to have little impact, with only slightly larger effects for women with secondary or higher education compared to women with less education. At the district level, impacts vary substantially, with the largest treatment effects in Madi-Okollo (the 0.12 SD increase here is almost twice as large as the effect we see in the sample overall), and the smallest impacts in Rwampara (0.02 SD). **Table 11:** Stratified results

Seconda ry or

Age 20 -	Primary	Madi-	Rwampar

Stratum	Age <20	Age 35+	higher	Kampala	Katakwi

34	education	Okollo	a

educatio n

Treatment	0.0213	0.0674*	0.0901*	0.0723*	0.0587*	0.0949**	0.0828*	0.122*	0.0221

effect

(0.127)	(0.0344)	(0.0500)	(0.0430)	(0.0331)	(0.0431)	(0.0455)	(0.0654)	(0.0517)

Observations	291	2,978	1,608	1,804	3,073	1,472	1,093	650	1,662

*Notes:* Coefficients displayed are mean differences with standard errors in parentheses. Adjusted models control for age group (age < 20, age 20-34 (ref), age >=35), educational attainment, district, baseline family planning knowledge, baseline hemoglobin level and baseline body mass index. Standard errors were clustered at the enumeration area level. *p-value<0.10, **p-value<0.05, ***p-value<0.01

**Harms statement**

No harm was reported during the trial.

## Discussion

The main objective of this study was to assess the impact of Viamo’s call-in platform on health knowledge, behaviours and outcomes. Conceptually, knowledge platforms could result in changes on three distinct levels: changes in health knowledge, changes in health behaviour, and changes in health outcomes. The results presented here suggest that changes in knowledge definitely occurred, with treated women displaying improved sex and reproductive health knowledge, being better informed about anemia treatment and more familiar with a larger range of contraceptive methods. This increased familiarity with contraceptive knowledge was also partially visible in contraceptive use. Treated women were on average only marginally (and insignificantly) more likely to use modern contraception, but used a different mix of contraceptives, with higher usage of implants, and somewhat reduced reliance on injectables and calendar methods. In terms of future plans, the intervention seems to have substantially increased the proportion of women who say they will use modern contraception in the future.

For anemia - one of the key health challenges addressed on the platform - the treatment seems to have resulted in an increased uptake of supplements during pregnancy; these higher rates of supplementation also seem consistent with marginally lower rates of anemia among recently pregnant women. The estimated differences in anemia in this subgroup are however not statistically significant, which may partially be due to the relatively small number of women. Despite the high rates of anemia among non-pregnant women, we did not see any change in general protein intake or consumption of iron supplements. Given this lacking change (and the small proportion of women benefiting from additional supplementation during pregnancy), the insignificant impact on general hemoglobin levels makes sense.

We also see some interesting declines in the rates of pregnancy loss and the prevalence of lowbirth-weight births in this subsample, but did not have sufficient statistical power here due to the relatively small number of women completing a pregnancy within the study period.

Given that a larger proportion of women in the treatment group claimed to aim for a healthy diet and to eat more fruit and vegetables, nutrition related content clearly reached its target.

Interestingly, the messages received around “healthy body shapes” don’t seem to have triggered a major shift towards eating less and lower BMIs, but seems to have induced a desire to change in both directions, i.e. we see an increasing proportion of women who want to lose weight and an increasing proportion of women who want to gain weight (and hence a smaller proportion of women who want to stay as they are). These shifts in intentions and behaviors are not seen in the anthropometric data - the intervention neither changed average BMI nor the proportions of women classified as underweight, overweight or obese.

Despite the very large, stratified and regionally representative sample, the study has some limitations. First, there were not as many pregnant women as anticipated, reducing power on pregnancy-related behaviours and outcomes to some extent. Second, we found that the proportion of women using Airtel phones was substantially lower in some rural districts, making it very difficult to reach enrollment targets in some communities. Even though a more selected sample clearly does not undermine the internal validity of the study (since randomization was done at the individual level), it is not clear whether the final set of women enrolled in the study is fully representative of the larger populations in the four study districts as well as Uganda overall - since the service is currently only available to Airtel users, this study simply focused on women with access to this service.. Third, many of the behaviours measured were self-reported; while we implemented verification measures by taking pictures of antenatal and child health cards and asking women for evidence of contraceptive packaging, such direct evidence was generally only available for a small minority of women, and could thus not be used in the analysis. It is clearly possible that access to the service changes norms and reporting bias, which could explain some of the behavioural changes reported and detected in our analysis. The primary outcome of the study - the reproductive knowledge score - should not be subject to any reporting bias as all women should have the incentive to give the correct answer on the true/false knowledge quiz. Fourth, the individual-level randomization made the study vulnerable to spillover effects. Qualitative feedback collected at the end of the study suggests that treated women both invited other community members – spouses, friends and other family members – to use the service and actively shared new information with them. Given that some of this shared information likely reached the control group, it seems likely that the impact reported here underestimate the true causal effect of the program. We controlled for direct service use by excluding active users in the per protocol analysis (Table 10) – this does however not capture direct sharing of knowledge among community members.

## Conclusions

Overall, the results presented here suggest that the platform can effectively improve health knowledge and to some extent also health behaviours among vulnerable populations. These changes in knowledge, awareness and intentions could plausibly also induce changes in health outcomes in the long run; longer-term studies will likely be needed to accurately measure such effects. Future research should also aim to quantify knowledge spillovers within communities as well as the cost-effectiveness of call-in platform like the one analysed here relative to other communication channels such as TV, radio, posters or community-based activities.

**Acknowledgements:** We would like to thank all involved staff members at IPA Uganda as well as in the Viamo team for their efforts and continued support of the project. We are also grateful to the more than 6000 study participants for their willingness to participate in the study. Lastly, we would like to thank the USAID Development Innovation Ventures (DIV) team for their input and feedback throughout the project.

**Funding:** this project was funded through USAID DIV grant number 7200AA21FA00018.

**Role of funder:** The funder played no role in the analysis or interpretation of the data.

# References

1. World Bank, *Individuals using the Internet (% of population) | Data.* 2025.
2. D'Souza, P., et al., *Factors influencing contraception choice and use globally: a synthesis of systematic reviews.* The European Journal of Contraception & Reproductive Health Care: The Official Journal of the European Society of Contraception, 2022. **27**(5): p. 364-372.
3. Noar, S.M., *A 10-year retrospective of research in health mass media campaigns: where do we go from here?* Journal of Health Communication, 2006. **11**(1): p. 21-42.
4. Wakefield, M.A., B. Loken, and R.C. Hornik, *Use of mass media campaigns to change health behaviour.* Lancet, 2010. **376**(9748): p. 1261-1271.
5. Stead, M., et al., *Mass media to communicate public health messages in six health topic areas: a systematic review and other reviews of the evidence*. Public Health Research. 2019, Southampton (UK): NIHR Journals Library.
6. Uganda Communications Commission, *Market performance report 2022/2023*. 2023, Uganda Communications Commission.
7. Rokicki, S., et al., *Impact of a Text-Messaging Program on Adolescent Reproductive Health: A Cluster–Randomized Trial in Ghana.* American Journal of Public Health, 2017. **107**(2): p. 298-305.
8. Unger, J.A., et al., *Short message service communication improves exclusive breastfeeding and early postpartum contraception in a low- to middle-income country setting: a randomised trial.* BJOG: an international journal of obstetrics and gynaecology, 2018. **125**(12): p. 1620-1629.
9. Harrington, E.K., et al., *An mHealth SMS intervention on Postpartum Contraceptive Use Among Women and Couples in Kenya: A Randomized Controlled Trial.* American Journal of Public Health, 2019. **109**(6): p. 934-941.

Supporting Information

[Click here to access/download](https://www2.cloud.editorialmanager.com/pone/download.aspx?id=39184685&guid=82be1a18-aa12-456c-9d76-5ca591f4f202&scheme=1)

[Supporting Information](https://www2.cloud.editorialmanager.com/pone/download.aspx?id=39184685&guid=82be1a18-aa12-456c-9d76-5ca591f4f202&scheme=1)

[Supplemental Materials 3 Feb 2025.pdf](https://www2.cloud.editorialmanager.com/pone/download.aspx?id=39184685&guid=82be1a18-aa12-456c-9d76-5ca591f4f202&scheme=1)
