# Supplementary material for: Estimating the impact of Viamo call-in information services on sexual and reproductive health knowledge, behavior, and outcomes among women of reproductive age: A 2-arm open label randomized controlled trial in Uganda
Source: PLoS One. 2026 Feb 13;21(2):e0342327. doi: 10.1371/journal.pone.0342327 (PMC12904432; doi:10.1371/journal.pone.0342327)
Supplement: Attachment — Submitted filename: Reply to reviewers 12 Sep 2025.docx [file pone.0342327.s009.docx]

**Journal Requirements:**

***When submitting your revision, we need you to address these additional requirements.***

***1. Please ensure that your manuscript meets PLOS ONE's style requirements, including those for file naming. The PLOS ONE style templates can be found at***

[***https://journals.plos.org/plosone/s/file?id=wjVg/PLOSOne_formatting_sample_main_body.pdf***](https://journals.plos.org/plosone/s/file?id=wjVg/PLOSOne_formatting_sample_main_body.pdf)

***and***

[***https://journals.plos.org/plosone/s/file?id=ba62/PLOSOne_formatting_sample_title_authors_affiliations.pdf***](https://journals.plos.org/plosone/s/file?id=ba62/PLOSOne_formatting_sample_title_authors_affiliations.pdf)

We have reviewed the manuscript and reformatted the text following the PLOS one style templates.

***2. Please describe in your methods section how capacity to provide consent was determined for the participants in this study. Please also state whether your ethics committee or IRB approved this consent procedure. If you did not assess capacity to consent please briefly outline why this was not necessary in this case.***

We read out the consent form to illiterate women and asked them to provide a thumb print to express their explicit consent instead of signing. This process was suggested and approved by local IRB. Women not able to understand the scope of the study as well as women not able or willing to consent using either modality were excluded from the study.

**3. Please include a complete copy of PLOS’ questionnaire on inclusivity in global research in your revised manuscript. Our policy for research in this area aims to improve transparency in the reporting of research performed outside of researchers’ own country or community. The policy applies to researchers who have travelled to a different country to conduct research, research with Indigenous populations or their lands, and research on cultural artefacts. The questionnaire can also be requested at the journal’s discretion for any other submissions, even if these conditions are not met. Please find more information on the policy and a link to download a blank copy of the questionnaire here: https://journals.plos.org/plosone/s/best-practices-in-research-reporting. Please upload a completed version of your questionnaire as Supporting Information when you resubmit your manuscript.**

We have completed the inclusivity questionnaire and have uploaded it with the revised documents.

***4. Thank you for stating the following financial disclosure:***

***“This project was funded through USAID DIV grant number 7200AA21FA00018”***

***Please state what role the funders took in the study. If the funders had no role, please state:*** ***"The funders had no role in study design, data collection and analysis, decision to publish, or preparation of the manuscript."***

***If this statement is not correct you must amend it as needed.***

***Please include this amended Role of Funder statement in your cover letter; we will change the online submission form on your behalf.***

This is correct - we have included this statement in the cover letter as suggested.

***5. Thank you for stating the following in the Competing Interests section:***

***“The first, second and third author declare no competing interest. The last author (IK) is currently employed by Viamo, the company operating the service analyzed. She was not involved in the data analysis and did not modify the way the results are presented or described in any way.”***

***Please confirm that this does not alter your adherence to all PLOS ONE policies on sharing data and materials, by including the following statement: "******This does not alter our adherence to PLOS ONE policies on sharing data and materials.” (as detailed online in our guide for authors http://journals.plos.org/plosone/s/competing-interests). If there are restrictions on sharing of data and/or materials, please state these. Please note that we cannot proceed with consideration of your article until this information has been declared.***

***Please include your updated Competing Interests statement in your cover letter; we will change the online submission form on your behalf.***

We have included an updated conflict of interest statement in the cover letter.

***6. When completing the data availability statement of the submission form, you indicated that you will make your data available on acceptance. We strongly recommend all authors decide on a data sharing plan before acceptance, as the process can be lengthy and hold up publication timelines. Please note that, though access restrictions are acceptable now, your entire data will need to be made freely accessible if your manuscript is accepted for publication. This policy applies to all data except where public deposition would breach compliance with the protocol approved by your research ethics board. If you are unable to adhere to our open data policy, please kindly revise your statement to explain your reasoning and we will seek the editor's input on an exemption. Please be assured that, once you have provided your new statement, the assessment of your exemption will not hold up the peer review process.***

We have uploaded a complete replication package comprising both the data files and the Stata DO files creating figures and tables as supplementary materials.

***7. We note that Figure 1 in your submission contain [map/satellite] images which may be copyrighted. All PLOS content is published under the Creative Commons Attribution License (CC BY 4.0), which means that the manuscript, images, and Supporting Information files will be freely available online, and any third party is permitted to access, download, copy, distribute, and use these materials in any way, even commercially, with proper attribution. For these reasons, we cannot publish previously copyrighted maps or satellite images created using proprietary data, such as Google software (Google Maps, Street View, and Earth). For more information, see our copyright guidelines: http://journals.plos.org/plosone/s/licenses-and-copyright.***

***We require you to either (1) present written permission from the copyright holder to publish these figures specifically under the CC BY 4.0 license, or (2) remove the figures from your submission:***

This shapefile comes from GADM, which explicitly allows such use: “*Using the data to create maps for publishing of academic research articles is allowed. Thus you can use the maps you made with GADM data for figures in articles published by PLoS, Springer Nature, Elsevier, MDPI, etc. You are allowed (but not required) to publish these articles (and the maps they contain) under an open license such as CC-BY as is the case with PLoS journals and may be the case with other open access articles.*” (https://gadm.org/license.html),

We have added the source to the legend of Figure 1.

***8. Please include captions for your Supporting Information files at the end of your manuscript, and update any in-text citations to match accordingly. Please see our Supporting Information guidelines for more information: http://journals.plos.org/plosone/s/supporting-information.***

We have relabeled the supplemental material figures following these guidelines.

***9. If the reviewer comments include a recommendation to cite specific previously published works, please review and evaluate these publications to determine whether they are relevant and should be cited. There is no requirement to cite these works unless the editor has indicated otherwise.***

NA – we did not received any such requests.

**Reviewers' comments:**

**Reviewer #1:**

***Thank you for sharing this very interesting study for review. Below are some points that might help the authors improve their manuscript.***

Thank you for the careful review and very helpful suggestions – we have done our best to address all of them as outlined in further detail below.

***Introduction:***

***- It would be beneficial to mention a clear hypothesis or research question at the end of the introduction. This will guide readers in understanding how the results align with the study’s goals.***

Thank you for this suggestion. We have added the following sentence to the Introduction:

*“Our primary hypothesis was that access to the platform can improve sexual and reproductive health knowledge and attitudes.”*

***Methods:***

***- The methodology is well-designed and aligns with the CONSORT checklist. However, further details are needed regarding the data collection tools. Specifically:***

***- Which tools were used to assess the primary and secondary variables?***

Apologies for the lack of detail here in the original manuscript. We provide further details on the outcomes variables on page 8 and 9 now, where we write:

*“The two primary outcomes of the study were reproductive health knowledge and nutritional supplementation in pregnancy. For reproductive health, we used a 15-item questionnaire of true/false statements (items listed in Supplemental Materials S2). This tool was based on previous studies conducted in sub-Saharan Africa [7] and adapted to match local context. To facilitate interpretation of regression results, we converted the raw scores (number of correct answers out of 15) into z-scores with mean zero and standard deviation one. For antenatal care, we used self-reported iron supplementation as an outcome measure. Secondary outcomes included contraceptive use, prenatal care uptake, hemoglobin level (as a biomarker indicating actual iron intake), body mass index, and birth spacing.*

*For pregnancy related outcomes, we analyzed two separate sets of women: first, women reporting to be pregnant at the time of the endline survey (currently pregnant), and second, women who completed a pregnancy in the year preceding the endline survey (recently pregnant). For current pregnancies, we analyzed self-report iron supplementation as well as self-reported use of antenatal care services, and whether women indicate the pregnancy was planned. For recently completed pregnancies, we analysed the same variables, but also analysed pregnancy loss and low birth weight (reported weight at birth < 2.5kgs) as additional secondary outcomes.*

***- Validity and reliability of the measurement tools should be addressed to ensure the robustness of the results.***

We added some more details in the Methods section, where we write:

“*This tool was based on previous studies conducted in sub-Saharan Africa [7] and adapted to match local context.*”

For the secondary outcomes, self-reporting biases are definitely a concern which we discuss in the Limitations section of the paper, where we write:

*“Third, many of the behaviors measured were self-reported; while we implemented verification measures by taking pictures of antenatal and child health cards and asking women for evidence of contraceptive packaging, such direct evidence was generally only available for a small minority of women, and could thus not be used in the analysis. It is clearly possible that access to the service changes norms and reporting bias, which could explain some of the behavioral changes reported and detected in our analysis. The primary outcome of the study - the reproductive knowledge score - should not be subject to any reporting bias as all women should have the incentive to give the correct answer on the true/false knowledge quiz.”*

***- The term “normalized” is used to describe the data collection tool. This terminology is not common in this context. Consider replacing it with terms like “valid and reliable.”***

Our apologies – we just meant to say that we created a z-score out of the raw number of correct answers prior to analysis. We have rephrased this as follows in the revised Methods section:

*“To facilitate interpretation of regression results, we converted the raw scores (number of correct answers out of 15) into z-scores with mean zero and standard deviation one.”*

***- The terms “reproductive health knowledge” and “antenatal care” seem to overlap or create confusion. Please clarify the distinction between these terms and ensure consistency throughout the manuscript.***

Apologies for the confusion: we meant to refer to nutritional supplementation during antenatal care here, and have made this more explicit in the revised version of the manuscript where we write:

“For nutritional supplementation during pregnancy, we used self-reported iron supplementation as an outcome measure”

***- Details regarding exclusion criteria are missing and should be provided.***

Apologies for the lack of detail here – we have added a new section on inclusion and exclusion criteria, which reads as follows:

*“..Study eligibility was assessed by trained study enumerators. Women living in selected enumeration areas were eligible for inclusion in the study if they were aged 18-49 years and had access to an Airtel (a mobile network operator) phone number within their household. Given the explicit focus on antenatal care, family planning and post-partum, all women who were pregnant during the listing were automatically invited to join the study; non-pregnant women were randomly selected for the study until the 45 women per cluster target was reached. In clusters with less than 45 women, all women were invited to participate in the study. Women were ineligible if they had used the Viamo platform previously, they planned to move outside of the study area within 12 months of baseline or if they did not speak one of the six languages available on the platform: Ateso, English, Luganda, Lugbara, Luo or Runyakitara.*”

***- Clarify who assessed eligibility and whether they were blinded during the randomization process.***

We have added the following information***:***

*“Study eligibility was assessed by trained study enumerators.”*

***- The manuscript contains 11 tables, which may be excessive. Consider merging some tables or limiting the number to those most essential for clarity. Ensure all tables mentioned in the manuscript are explained in the results section.***

We agree that there is lots of information in this manuscript, but really believe that all of these results are important. Given that there is no formal limit at PLOS One, we would thus prefer to leave all figures and tables in, and have verified all of them are mentioned in the text. If you feel strongly that some figures should go to the Supplemental Materials we would of course be happy to accommodate that***.***

***- There is no mention of the interventional materials used with the experimental group. This information is necessary for understanding the study design and replication in future research.***

We fully agree with this. We provide a detailed description of the intervention on page 6. The intervention is easy and complex at the same time: it is easy, because essentially women are just invited to call a toll-free number and then get to access content designed for them. The complexity comes from the fact that women can freely navigate content. We show the most commonly accessed items in Table 3, and provide a more detailed example in supplemental materials S1.

***- The discussion section lacks comparisons with previous studies. Including these would contextualize the findings and add depth.***

***Thank you for this suggestion. We have added the following paragraph to the Disucssion:***

***“****In terms of the primary outcome, our results seem consistent with a recent review of mHealth intervention, which suggests that digital information sources can improve sexual and reproductive health knowledge as well as use of sexual and reproductive health services [10]. Compared to the estimates reported for an interactive intervention in Ghana [7, 11] the effect sizes observed here seem smaller, which seems plausible given that not all women engaged with the service on a regular service. “*

***- Discuss the study’s limitations and strengths to aid future researchers.***

We discuss strengths and limitatios at the very end of the Discussion section, where we write:

*“Despite the very large, stratified and regionally representative sample, the study has some limitations. First, there were not as many pregnant women as anticipated, reducing power on pregnancy-related behaviors and outcomes to some extent. Second, we found that the proportion of women using Airtel phones was substantially lower in some rural districts, making it very difficult to reach enrollment targets in some communities. Even though a more selected sample clearly does not undermine the internal validity of the study (since randomization was done at the individual level), it is not clear whether the final set of women enrolled in the study is fully representative of the larger populations in the four study districts as well as Uganda overall - since the service is currently only available to Airtel users, this study simply focused on women with access to this service.* *Third, many of the behaviors measured were self-reported; while we implemented verification measures by taking pictures of antenatal and child health cards and asking women for evidence of contraceptive packaging, such direct evidence was generally only available for a small minority of women, and could thus not be used in the analysis. It is clearly possible that access to the service changes norms and reporting bias, which could explain some of the behavioral changes reported and detected in our analysis. The primary outcome of the study - the reproductive knowledge score - should not be subject to any reporting bias as all women should have the incentive to give the correct answer on the true/false knowledge quiz. Fourth, the individual-level randomization made the study vulnerable to spillover effects. Qualitative feedback collected at the end of the study suggests that treated women both invited other community members – spouses, friends and other family members – to use the service and actively shared new information with them. Given that some of this shared information likely reached the control group, it seems likely that the impact reported here underestimate the true causal effect of the program. We controlled for direct service use by excluding active users in the per protocol analysis (Table 10) – this does however not capture direct sharing of knowledge among community members.”*

***- The trial flowchart should be reshaped to align with the original CONSORT 2010 format. available at CONSORT 2010***

We tried to follow the original template as closely as possible. The main challenge with our study is that we had two follow-up modalities, which required as to make the format a bit wider than usual. We are not sure how to simplify this but are open to suggestions.

***Reviewer #2:***

***I commend the authors' work to assess the impact of Viamo’s call-in platform on sexual and reproductive health knowledge, behaviors and outcomes.***

***Please find my comments below (pg numbers used in the absence of line numbers):***

Thank you for the nice feedback and very helpful suggestions – we really appreciate it!

***-pg 1/27: Title: Please consider revising the title to reflect the aim of this manuscript and the analysis/discussion/conclusion. For instance, "Estimating the Impact of Viamo Call-in Information Services on Sexual and Reproductive Health Knowledge, Behavior, and Outcomes among women of reproductive age: Results from a 2-Arm Open Label Randomized Controlled Trial in Uganda" may be a more appropriate title***

Thank you for this suggestion – we have adopted this new title.

***-pg 1/27: Please maintain a consistent spelling for "behavior". In some aspects of the manuscript, this is spelt at "behaviour"***

Thanks for catching this and our apologies for the inconsistent US/UK spelling – we now use behavior throughout.

***-pg 2/27: Please cite this source of this sentence, "The platform is free of charge, and currently has over 4 million users in Uganda, and 42 million users in LMICs globally."***

***This was an internal number provided by Viamo; we have replaced this now with an official UNDP estimate (which is slightly lower):***

*“The platform is free of charge, and currently has over 4 million users in Uganda, and 35 million users in LMICs globally [10].»*

***-pg 9/27: "Follow-up rates (completing either the phone or the in person survey or both) were 92% in both arms." Please review this sentence and consider removing it since the estimates do not correspond with Figure 2 and the other sentences in this section of the manuscript better interpret the numbers.***

Apologies for the lack of clarity here. We used two parallel modalities (with overlapping questions) to reach households at endline. Some of the households completed both and some just one survey – 92% completed either one or both surveys. We have tried to be more clear about this in the revised Results section where we write:

*“At endline, 87% (n=2,678) of control arm participants, and 86% (n=2,555) of intervention arm participants completed the phone survey. Slightly fewer participants completed the in-person survey (81% of both arms).* *92% of participants in both arms completed at least one of the two modalities (i.e. either a phone or an in-person survey).”*

***-pg 11/27: Table 2: Please review the results with *** (p-value <0.01). All the P-values are >0.01 and ** (p-value<0.05) would be more appropriate. In addition, some p-values are not significant (0.471 and 0.958) and should not have any asterisk on them.***

Sorry for the confusion. The numbers in parentheses here are standard errors, and the p-values are all substantially below 0.01 in Table 2. We have made this clear in the revised Table 2 legend that now says:

*“Notes: All point estimates displayed correspond to estimated mean differences in outcomes. Numbers in parentheses are robust standard errors.»*

The asterisk notation (* for < 0.10, *** for < 0.01) is the default of Stata’s outreg package – we would be happy to revise this if you feel strongly about this.

***-pg 12/27: Table 3 footnote: How was C2L calculated? It doesn’t seem to Times listened / Times reached based on the table. Please add the formula to the footnote.***

***This rate is simply the proportion of callers who listen to the message all the way to the end – this is automatically registered in the system. We have clarified this in the revised legend of the table, where we now write:***

*“C2L (caller-to-listener ratio) defines the % completion listening rate of a message, i.e. the proportion of callers who listen to the message all the way to the end.”*

***-pg 13/27: Table 4: Please review the result with *** (p-value <0.01) because ** (p-value<0.05) would be more appropriate for p-value 0.0265 – row 2, column 2.***

Once again, we apologize for the lack of clarity. The numbers in parentheses are not p-values but estimated standard errors. We have made this clear in the legend of the table now where we write:

*“Coefficients displayed are mean differences with standard errors in parentheses.”*

***-pg 15/27: Table 5: Please review the results with *** (p-value <0.01). All the P-values are >0.01 but <0.05 and ** (p-value<0.05) would be more appropriate.***

***-pg 16/27: Table 6: Please review the results with * (p-value <0.01). All of them are <0.05 and ** (p-value<0.05) would be more appropriate.***

***-pg 17/27: Table 7: Please review the results with *** (p-value <0.01) and * (p-value <0.10) and correct them as appropriate. All the p-values should have ** except p-value for 0.0174 (0.00965) which should have-pg 18/27: Table 8: Please include the corresponding asterix for the estimates with significant p-values in this table.***

***-pg 19/27: Table 9: Please include the corresponding asterix for the estimates with significant p-values in this table.***

***-pg 20/27: Table 10: Please include the corresponding asterix for the estimates with significant p-values in this table.***

***-pg 21/27: Table 11: Please review the results with p-values 0.0344*, 0.0331* and 0.0455* (where * is p-value <0.10) and correct them to ** (p-value <0.05) as appropriate. All should have ** except p-value: 0.00965 which should have ******

Once again, we apologize for the lack of clarity. The numbers in parentheses are not p-values but estimated standard errors. We have clearly indicated in the legends of all tables now.

***-pgs 22-24: Discussion: Please incorporate existing studies that support or dispute your findings in the discussion section. This section needs a lot more work and is currently only a repetition/elaboration of the results' interpretation.***

We have carefully revised the Discussion and added the following next text block to better place the study results into the existing literature:

*“In terms of the primary outcome, our results seem consistent with a recent review of mHealth intervention, which suggests that digital information sources can improve sexual and reproductive health knowledge as well as use of sexual and reproductive health services [11]. Compared to the estimates reported for an interactive intervention in Ghana [7, 12] the effect sizes observed here seem smaller, which seems plausible given that not all women engaged with the service on a regular service. Compared to previous studies conducted in other parts of Africa we also observed rather high sexual and reproductive health knowledge at baseline – most women were for example familiar with a range of contraceptive methods (beyond traditional methods, condoms and pills), and myths regarding side effects of contraception were less common that seen in other settings [7, 12] – this likely also contributes to the somewhat smaller knowledge impacts seen here.*

*In terms of policy, one of the main advantages of the platform used is its relatively low cost – particularly when run at large scale; most of the costs of content development are related to the initial set-up, while operating costs are relatively minor. More research is needed here to investigate the cost-effectiveness of these new mobile technology channels as tools to improve health knowledge among vulnerable populations relative to traditional channels such as radio, tv or community outreach activities. “*

***Reviewer #3: I struggled to review this paper specifically the results section.***

***I could not figure out what metric was used to measure impact (such odd ratios).***

***The measurement of the outcome is not clear or easily understood.***

We apologize for the lack of clarity in the original manuscript. We have added additional details on the outcome measurements in the Tables, and also have added further details in on the main outcome in the revised Methods section, where we write:

*“For reproductive health, we used a 15-item questionnaire of true/false statements (items listed in Supplemental Materials S2).* *This tool was based on previous studies conducted in sub-Saharan Africa [7] and adapted to match local context.* *To facilitate interpretation of regression results, we converted the raw scores (number of correct answers out of 15) into z-scores with mean zero and standard deviation one.* *For nutritional supplementation during pregnancy, we used self-reported iron supplementation as an outcome measure. Secondary outcomes included contraceptive use, prenatal care uptake, hemoglobin level (as a biomarker indicating actual iron intake), body mass index, and birth spacing.”*

We hope this makes it easier to understand our analysis now.
