# Supplementary material for: Estimating the impact of Viamo call-in information services on sexual and reproductive health knowledge, behavior, and outcomes among women of reproductive age: A 2-arm open label randomized controlled trial in Uganda
Source: PLoS One. 2026 Feb 13;21(2):e0342327. doi: 10.1371/journal.pone.0342327 (PMC12904432; doi:10.1371/journal.pone.0342327)
Supplement: Attachment — Submitted filename: Review Comments to the Author.docx [file pone.0342327.s007.docx]

**Review Comments to the Author**

**Recommendation:** **Major Revision**

The authors present a well-written and well-conducted RCT evaluating the impact of a mobile based information dissemination strategy (Viamo) on sexual and reproductive health knowledge and behaviors among women. I believe the study addresses a significant public health problem. The authors are requested to provide many clarifications and improvements before publication.

**Section by section review and suggestions**

**Title**

- The revised title is clear and concise containing all necessary information.
- **Suggestions:**
- **Avoid unnecessary capitalizations** in the title (all should be written in lowercase except the beginning of the starting word and the name “Uganda”).
- I also recommend **replacing the word “Results” by “Findings”** in the statement: Results from a 2-Arm Open Label Randomized Controlled Trial in Uganda.

**Abstract**

- The abstract is well-organized and succinctly summarizes each section of the study.
- **Suggestion:**
- Do not use abbreviations in abstract section (e.g. “BMI, 4% pts”) to make it easy for unfamiliar readers.
- Use two points (:) rather than a dot two separate the subtitles of the abstract with the details. E.g. Background: xxxxxxxx
- Better to replace “Background” by “Introduction”

**Introduction**

- The introduction effectively sets the context and states the primary hypothesis.
- **Suggestion:** Briefly mention the specific mechanisms by which call-in services might overcome limitations of mass media (e.g., on-demand access, personalized content).

**Methods**

- **Concerns:**
  1. You stated that “Blinding of the intervention was not possible given the nature of the program”. It lacks detail.
  2. **Consent process:**
- Not assessing cognitive capacity to consent may raise potential ethical concern. It should be clearly justified.
- In the inclusion and exclusion criteria, the authors write that “if they did not speak one of the six languages available on the platform, they will be excluded”: this may raise ethical concern. Why didn’t you consider language translators for women not speaking the six languages rather than excluding them?
  3. **Measurment tools:** The authors simply write “15-item knowledge tool is adapted from prior studies”, but more detail on tool validation in the context of Ugandan would make the manuscript more strong.
  4. **Blinding:** Describe in detail how outcomes are influenced by the lack of blinding.
  5. **Sample representativeness:** The restriction to Airtel users may limit generalizability, which is noted in limitations but could be emphasized earlier.

**Results**

- Results are comprehensive and well-presented across multiple tables and figures.
- **Concerns:**
  1. **Table clarity:** Many tables use asterisks to denote significance, but parentheses contain standard errors, not p-values. This is now clarified in legends, but the presentation remains potentially confusing. Consider adding a separate column for p-values.
  2. **Figure 2 (CONSORT):** The flowchart is informative but visually crowded. Consider simplifying or splitting into two panels (phone vs. in-person follow-up).

**Discussion**

- The discussion appropriately interprets results in light of prior literature and acknowledges limitations.
- **Strengths:** The addition of comparisons with mHealth studies in Ghana and discussion of cost-effectiveness.
- **Suggestion:** Expand on why effect sizes were smaller than in similar interventions (e.g., lower engagement, higher baseline knowledge). Discuss implications for scaling and tailoring such interventions.

**Conclusion**

- Conclusions are supported by the data and highlight the need for longer-term studies.
- **Suggestion:** Include a brief statement on policy or programmatic recommendations.

**Supplementary Materials**

- The replication package, inclusivity questionnaire, and supplemental tables/figures are commendable and enhance transparency.
- **Suggestion:** Ensure all supplemental files are clearly referenced in the main text (e.g., “Supplemental Materials S2” is referenced but should be listed in the manuscript’s Supporting Information captions section).

**Responses to PLOS ONE Review Questions**

**1. Is the manuscript technically sound, and do the data support the conclusions?**
Yes. The study is well-designed, and the data generally support the conclusions. Minor revisions are needed to clarify methodological details and result presentation.

**2. Has the statistical analysis been performed appropriately and rigorously?**
Yes. Appropriate methods (ITT, per-protocol, cluster-adjusted SEs) are used. The stratified analyses add depth.

**3. Have the authors made all data underlying the findings fully available?**
Yes. The authors have provided a replication package with data and code, meeting PLOS ONE’s data availability policy.

**4. Is the manuscript presented intelligibly and in standard English?**
Mostly. The manuscript is well-written, but some sections are dense with tables. A few typos remain (e.g., “behavious” in Abstract, “den’t” in Discussion). Recommend thorough proofreading.

**5. Additional Comments:**

- The study adheres to CONSORT guidelines, and the protocol is registered.
- The inclusivity questionnaire is a valuable addition.
- No major ethical or dual-use concerns were identified.
- Software used (Stata) is standard and accessible.

**Confidential to Editor**

I have no competing interests.

**Overall Recommendation**

This is a valuable contribution to the literature on mHealth interventions in LMICs. With the suggested revisions—particularly around clarifying methodological details, improving table/figure readability, and strengthening the discussion—the manuscript will be suitable for publication in *PLOS ONE*.
