# Supplementary material for: Estimating the impact of Viamo call-in information services on sexual and reproductive health knowledge, behavior, and outcomes among women of reproductive age: A 2-arm open label randomized controlled trial in Uganda
Source: PLoS One. 2026 Feb 13;21(2):e0342327. doi: 10.1371/journal.pone.0342327 (PMC12904432; doi:10.1371/journal.pone.0342327)
Supplement: Attachment — Submitted filename: REVIEWER COMMENTS.docx [file pone.0342327.s006.docx]

**REVIEWER COMMENTS**

Thank you for sharing this study for review. Below are some points that might help the authors revamp their manuscript.

**Abstract**

It will be beneficial to add dad analysis section to the methods section of the abstract. Which statistical test were used.

If you decide to retain vulnerable population in the title, it will be important to clarify which specific vulnerable population you studied. Otherwise, I will suggest that you remove the vulnerable population in the title and put “women of reproductive age”, and hence the background section will need to be revised.

I am curious about the choice of test used in the data analysis and why the continuous variables

**Introduction**

The first paragraph in this section sounds like a problem statement and also this entire section needs to be revised. I suggest that this section be revised as follows:

Introduce the reproductive age group as the predominant age group in Uganda

Health outcomes common among the reproductive age group (vulnerable population)

Emerging role of mobile phone app (Viamo Call-in Information Services)/internet coverage/technology in health education and improvement of reproductive and sexual health outcome in the Ugandan population. The proportion of the Ugandans with access to cell phones and/or internet. Previous studies in the impact of other cell phone apps on reproductive and sexual health outcome.

This section should now end with the problem statement and the rational for this study.

**Methods**
Study design: Were the 4 purposively selected districts representative of the entire Uganda population. This may act as a threat to external validity given that the 4 district doesn’t represent the minority regions. I guess this is a limitation.

**Sampling technique:** I think the sampling technique you described was a multistage cluster sampling technique and not a 2-stage random sampling. Also take a good look at different studies and revise the entire sampling procedure. The selection of 45 per cluster makes me worry if the selection was proportionate or representative each cluster? In order words, were the clusters of equal population for 45 to be selected from each clusters?

**Sample size:** How was the sample size of 6000 arrived at? It will make sense to add how it was calculated

**Randomization:** The section on randomization is very scanty and needs clarification. At this point its still not clear what the non-treatment arm received. Did they also benefit from Viamo Call-in Information Services, its important to clearly state what the intervention the 2 groups received. Its important to clearly define the intervention and the non-intervention group (treatment vs control group) and exactly what they received because its not clear.

**Primary and secondary outcomes:** The definition of primary and secondary outcomes (how were they measured) needs to be included in the methods section. Which tool was used to measure knowledge? Was it pretested or was it a validated tool?

**Data analysis:** I have noticed that the majority of the variables were analyzed as continuous variables. Did you consider converting the continuous variables (scores) to categorical variables which then permits you to use different methods.

**Results**

The presentation of results is not very clear. The tables are many and its not clear labelled if its correlation coefficient or a p-value.

**Discussion**

The discussion needs to be revised. It lack the comparison and contrast of the study findings with studies done locally and international.
