# Supplementary material for: Estimating the impact of Viamo call-in information services on sexual and reproductive health knowledge, behavior, and outcomes among women of reproductive age: A 2-arm open label randomized controlled trial in Uganda
Source: PLoS One. 2026 Feb 13;21(2):e0342327. doi: 10.1371/journal.pone.0342327 (PMC12904432; doi:10.1371/journal.pone.0342327)
Supplement: Attachment — Submitted filename: Reply to reviewer comments 16 Jan 2026.docx [file pone.0342327.s011.docx]

**Reviewer #4:**

***Thank you for sharing this study for review. Below are some points that might help the authors revamp their manuscript.***

Thank you for your kind and constructive comments – we have done our best to address all of them as outlined in detail below.

***Abstract
It will be beneficial to add analysis section to the methods section of the abstract. Which statistical test were used.***

Thank you for this suggestion. We have added the following sentence to the Abstract to clarify the empirical models used:

*“Linear regression models were used to estimate mean differences in outcomes between treatment and control. Standard errors were corrected for the cluster-based sampling of women.”*

***If you decide to retain vulnerable population in the title, it will be important to clarify which specific vulnerable population you studied. Otherwise, I will suggest that you remove the vulnerable population in the title and put “women of reproductive age”, and hence the background section will need to be revised.***

We have removed the “vulnerable populations” in the background section and now write:

*“..knowledge remains limited among women of reproductive health in many low and middle income countries”*

***I am curious about the choice of test used in the data analysis and why the continuous variables***

As stated above, we used linear regression models to estimate mean differences in outcomes between treated and controls. We also considered logistic regression models for the binary outcomes, but decided to stick with mean differences because they make comparisons in outcomes easy to communicate (and use cluster-robust standard errors to address non-normal residuals for binary outcomes).

***Introduction
The first paragraph in this section sounds like a problem statement and also this entire section needs to be revised. I suggest that this section be revised as follows:
Introduce the reproductive age group as the predominant age group in Uganda
Health outcomes common among the reproductive age group (vulnerable population)
Emerging role of mobile phone app (Viamo Call-in Information Services)/internet coverage/technology in health education and improvement of reproductive and sexual health outcome in the Ugandan population. The proportion of the Ugandans with access to cell phones and/or internet. Previous studies in the impact of other cell phone apps on reproductive and sexual health outcome. This section should now end with the problem statement and the rational for this study.***

We have restructured the Introduction as suggested and added several additional references. The first paragraph introduces the knowledge gap and highlights the health consequences of this gap for mothers and offsprings. The second paragraph summarizes current research on how to best improve knowledge among women of reproductive age. The third paragraph introduces cell phones as new tool and highlights key studies. The last one provides the objective and rationale of the study.

***Methods
Study design: Were the 4 purposively selected districts representative of the entire Uganda population. This may act as a threat to external validity given that the 4 district doesn’t represent the minority regions. I guess this is a limitation.***

We agree this is a limitation and acknowledge this in the revised Discussion section, where we write: .

*“…it is not clear whether the final set of women enrolled in the study is fully representative of the larger populations in the four study; it is also not clear if the four districts are fully representative of the country as a whole.”*

***Sampling technique: I think the sampling technique you described was a multistage cluster sampling technique and not a 2-stage random sampling. Also take a good look at different studies and revise the entire sampling procedure. The selection of 45 per cluster makes me worry if the selection was proportionate or representative each cluster? In order words, were the clusters of equal population for 45 to be selected from each clusters?***

We have changed the description to multi-stage sampling. The sampling was not proportional to population size and likely did not generate a fully representative sample – we say this clearly in the Discussion, where we write:

*“Second, we found that the proportion of women using Airtel phones was substantially lower in some rural districts, making it very difficult to reach enrollment targets in some communities. We compensated this by enrolling more women in clusters with higher coverage. Even though a more selected sample clearly does not undermine the internal validity of the study (since randomization was done at the individual level), it is not clear whether the final set of women enrolled in the study is fully representative of the larger populations in the four study districts.”*

***Sample size: How was the sample size of 6000 arrived at? It will make sense to add how it was calculated***

We explain this in the revised Power Calculations section, where we write:

*“The study was powered to detect a 0.1 standard deviation (SD) difference in standardized knowledge scores (mean 0, SD 1) with power 0.9. Assuming an average cluster size of 30, a follow-up rate of 90% and a design effect of 1.3, this required 100 clusters per arm, and a total sample size of 6000 women.”*

***Randomization: The section on randomization is very scanty and needs clarification. At this point its still not clear what the non-treatment arm received. Did they also benefit from Viamo Call-in Information Services, its important to clearly state what the intervention the 2 groups received. Its important to clearly define the intervention and the non-intervention group (treatment vs control group) and exactly what they received because its not clear.***

Apologies for the lack of clarity. We have added the following text to the Randomization section:

*“Women selected for the treatment arm were then provided with a personalized introduction to the service (after the baseline survey was completed) and called the service directly from their own phone with the support of the interviewer to see how the system works. Treated women were also given a promotional calendar to serve as a physical reminder on how to navigate the service and the benefits of the service. Women in the control group were not given any intervention. Given that the service was freely available to all women, they were in theory able to call the service at any time, but not given any encouragement or support to do so by the study team.”*

***Primary and secondary outcomes: The definition of primary and secondary outcomes (how were they measured) needs to be included in the methods section. Which tool was used to measure knowledge? Was it pretested or was it a validated tool?***

Apologies for the lack of detail here. We now explain this in more detail in the revised Methods section:

*“The two primary outcomes of the study were reproductive health knowledge and nutritional supplementation in pregnancy. For reproductive health, we used a 15-item questionnaire of true/false statements (items listed in Supplemental Materials S2). This tool was based on previous studies conducted in sub-Saharan Africa [11] and pretested locally to make sure all items were suitable for the study population. To facilitate interpretation of regression results, we converted the raw scores (number of correct answers out of 15) into z-scores with mean zero and standard deviation one. For nutrition knowledge, we analyzed a binary indicator for women (falsely) believing that herbs can cure anemia. For nutritional supplementation during pregnancy, we used self-reported iron supplementation as an outcome measure. Secondary outcomes included contraceptive knowledge (number of methods respondents are familiar with), contraceptive use (currently using any modern method), prenatal care uptake (any, number of visits), hemoglobin level (as a biomarker indicating actual iron intake), body mass index, and birth spacing.*

*For pregnancy related outcomes, we analyzed two separate sets of women: first, women reporting to be pregnant at the time of the endline survey (currently pregnant), and second, women who completed a pregnancy in the year preceding the endline survey (recently pregnant). For current pregnancies, we analyzed self-report iron supplementation as well as self-reported use of antenatal care services, and whether women indicate the pregnancy was planned. For recently completed pregnancies, we analyzed the same variables, but also analyzed pregnancy loss and low birth weight (reported weight at birth < 2.5kgs) as additional secondary outcomes. “*

***Data analysis: I have noticed that the majority of the variables were analyzed as continuous variables. Did you consider converting the continuous variables (scores) to categorical variables which then permits you to use different methods.***

We have explained this in further detail in the revised Methods section, where we write:

*“All primary models were estimated intent-to-treat, comparing outcomes between treated and control women at endline. For all outcomes, we estimated both unadjusted linear regression models (not including any covariates) and fully adjusted models, where we controlled for age, district, marital status, educational attainment and baseline knowledge variables. The main rationale for using linear regression models was to generate mean differences estimates that could be compared across the rather large set of outcome variables. To adjust for non-normal residuals, we used Huber’s cluster-robust standard errors [15].”*

***Results
The presentation of results is not very clear. The tables are many and its not clear labelled if its correlation coefficient or a p-value.***

We agree that there are many, but think it is important to show all of the results – also one of the reasons we chose PLOS ONE for this paper. We have added notes to all tables to be clear about what is shown (coefficients, SEs, p-values). For example, for the first regression Table (T2), the notes now say:

*“All point estimates displayed correspond to estimated mean differences in outcomes. Numbers in parentheses are cluster-robust standard errors. Asterisks indicate p-value range of estimated differences: *p-value<0.10, **p-value<0.05, ***p-value<0.01”*

***Discussion
The discussion needs to be revised. It lack the comparison and contrast of the study findings with studies done locally and international***.

There are not so many studies of similar nature, but we try to compare the main outcomes to the related literature in the Discussion, where we write:

*“In terms of the primary outcome, our results seem consistent with a recent review of mHealth intervention, which suggests that digital information sources can improve sexual and reproductive health knowledge as well as use of sexual and reproductive health services [16]. Compared to the estimates reported for an interactive intervention in Ghana [11, 17] the effect sizes observed here seem smaller, which seems plausible given that not all women engaged with the service on a regular service. Compared to previous studies conducted in other parts of Africa we also observed rather high sexual and reproductive health knowledge at baseline – most women were for example familiar with a range of contraceptive methods (beyond traditional methods, condoms and pills), and myths regarding side effects of contraception were less common that seen in other settings [11, 17] – this likely also contributes to the somewhat smaller knowledge impacts seen here. “*

Please let us know if there are any papers that we missed and should be cited here.

**Reviewer #5:**

***I appreciate the submission of this manuscript. Please find my comments below, which may be helpful in improving the quality of your work.***

Thank you for the kind and helpful comments – we really appreciate your input and have done our best to address all suggestions and explained in further detail below.

***Pg. 1/26: It is recommended that the phrase “results from” in the title be omitted, as this would shorten the title without detracting from its intended meaning.***

Thank you for this suggestion – we have removed “results from” the title and agree that the shortened version sounds better.

***Pg. 2/26: - It is recommended that the phrase “and Outcomes” be included in the title, as currently presented at the end of the background, to ensure that the title is complete.***

We believe these titles are given by the journal and thus are not allowed to modify them. We do state the objective in the background; the outcomes are provided in the Methods subsection.

**- Please ensure that the sampling method, data collection tools, a brief description of the data collection procedure, and the statistical tests used are clearly described in the methods section.**

We have expanded the Methods section to provide more details: it now reads as follows:

*“We conducted a 2-arm open label randomized controlled trial involving 6,000 randomly selected women, ages 18-49 years, with access to a simple feature Airtel cell phone in Uganda. Fifty enumeration areas were randomly selected in Kampala, Madi-Okollo, Rwampara and Katakwi districts. Consenting women were asked to complete a baseline and endline survey implemented by trained interviewers using tablets through home visits. After completion of baseline, women were randomized with equal probability to treatment and control using a tablet-generated random number draw. Study staff introduced treated women to the call-in service and encouraged its use through various incentives and promotional messages. Primary outcomes were sexual and reproductive health knowledge scores as well as iron supplementation in pregnancy. Secondary outcomes included self-reported use of contraception, healthy diets, birth spacing and birth outcomes as well as hemoglobin levels and BMI. Linear regression models were used to estimate mean differences in outcomes between treatment and control. Standard errors were corrected for the cluster-based sampling of women.”*

***Pg. 3/26: Please mention keywords at the end of the abstract.***

We have added the following keywords at the end of the abstract:

***Keywords****: reproductive health, call-in-services, health knowledge, Uganda, Viamo.*

***Pg. 4/26: Please provide further details regarding the Viamo platform and its operational mechanisms. Specifically, clarify whether the platform enables two-way interaction and whether users can experience a form of interactive support comparable to counseling, or whether information is delivered in a predominantly one-way manner, functioning mainly as a communication channel for educational purposes.
Please review relevant studies that have implemented similar interventions in the same research area, either locally or in other regions. Additionally, address studies that have evaluated the effectiveness of the Viamo platform or comparable mobile- or voice-based platforms for different purposes. Finally, summarize the existing literature and highlight the comparative advantage and necessity of the present intervention by identifying the gaps it aims to address. If no relevant studies are available, this should be clearly stated.***

Based on the feedback provided by Reviewer 4, we have removed the description of the service from the Introduction (page 4) and instead provide more details on the platform in an expanded Methods-Intervention section, where we provide the requested details. We compare this to other studies in the Discussion section – there is however not that much literature on similar models. In practice, Viamo does not provide direct counseling, but it offers a range of different topics users can select from – we think it can be best described as something like a “user-guided learning system”.

***Pg. 5/26 (Methods section):
- Please specify the exact nature of any encouragement designs and whether they may have preferentially attracted certain subgroups of participants. For example, as noted on page 7, "Participants received UGX 10,000 (~USD $2.77) of phone credit as a token of gratitude after each survey interview." Offering such incentives could disproportionately attract individuals with lower socioeconomic status, potentially limiting the generalizability. Therefore, if participants were aware of the incentives at the time of study invitation, this should be explicitly stated and discussed in the study limitations.***

We have provided further details on the encouragement in the Methods-Randomization section now, where we write:

*“Women selected for the treatment arm were then provided with a personalized introduction to the service (after the baseline survey was completed) and called the service directly from their own phone with the support of the interviewer to see how the system works. Treated women were also given a promotional calendar to serve as a physical reminder on how to navigate the service and the benefits of the service. Women in the control group were not given any intervention. Given that the service was freely available to all women, they were in theory able to call the service at any time, but not given any encouragement or support to do so by the study team.”*

The UGX 10,000 were proposed by the Ugandan IRB. On the consent form, these were stated, but not as incentive to participate, but simply to “compensate you for the time it will take to complete the survey”. Given that the surveys took up to two hours, this seemed fair – we did not observe any differential participation in the study (i.e. there were almost no refusals).

***- Please specify the exclusion (withdrawal) criteria during the study period, which criteria refer to circumstances that necessitate the removal of participants after enrollment, such as the death of a close family member.***

We did not have any such exclusion criteria during the study and have added this information to the Inclusion and Exclusion criteria section now, where we write:

*“There were no exclusion criteria (or exclusions) during the study.”*

***- Please specify the sample size and describe the method used for its calculation in the Methods section.***

We have revised the Power Calculations section to directly explain how the sample size was derived:

*“The study was powered to detect a 0.1 standard deviation (SD) difference in standardized knowledge scores (mean 0, SD 1) with power 0.9. Assuming an average cluster size of 30, a follow-up rate of 90% and a design effect of 1.3, this required 100 clusters per arm, and a total sample size of 6000 women.”*

***- Please provide additional details on how participants used the Viamo platform. Clarify whether platform use was need-based and participant-driven or task-oriented as part of the intervention.***

We have expanded to Intervention section to provide further details on this. The answer to your question is a mix of both – users could call at any time, but were also (in the treatment group only) encouraged to use the system:

*“Treated women were encouraged to use the system whenever they had questions or wanted to know more about sexual and reproductive health. Viamo also employed targeted nudges, personalized campaigns and other outreach activities (such as release of new content), with the aim of maintaining and incentivizing usage rates (active calling into the Viamo Platform) among women in the treatment group (see Supplemental Materials S1 for an example).”*

***- You mentioned in the limitations that information leak or unintended use of the platform by control group participants may have occurred. Please clarify whether any measures were taken to limit access to the platform or prevent its use by the control group and, if so, describe these measures in the Methods section. In addition, indicate whether any promotional activities (such as social media or other communication channels) were used that might have increased awareness of the platform and led to its use among control group participants.***

We were not able to prevent anybody from using the system, and state this clearly now in the revised Randomization section, where we write:

*“Women in the control group were not given any intervention. Given that the service was freely available to all women, they were in theory able to call the service at any time, but not given any encouragement or support to do so by the study team.”*

We are not aware of any social media or other programs promoting this channel and would have known since we worked closely with Viamo on this project.

***Pg. 7/26: Were there specific intervals between each measurement time point? If so, please specify these intervals.***

The idea was to have 15 months of program exposure between the two surveys. We have clarified this now in the revised Methods section where we write:

*“Baseline data was collected via face-to-face interview at each participants’ home between December 6, 2022 and March 6, 2023. Upon completion of the baseline survey, treated women were introduced to the service by study staff. Endline data was collected approximately 15 months after recruitment, between May 8, 2024 and August 4, 2024 in two separate surveys.”*

***- If the reproductive health questionnaire used in the study has a specific name or is a standardized tool recognized in the literature, please provide its official name and any relevant references.***

This is now explained in the revised Methods section as follows:

*“For reproductive health, we used a 15-item questionnaire of true/false statements (items listed in Supplemental Materials S2). This tool was based on previous studies conducted in sub-Saharan Africa [11] and pretested locally to make sure all items were suitable for the study population. To facilitate interpretation of regression results, we converted the raw scores (number of correct answers out of 15) into z-scores with mean zero and standard deviation one.”*

***- The current description of study variables appears unclear and potentially confusing. The title refers to Sexual and Reproductive Health Knowledge, Behavior, and Outcomes. The explanation provided under primary and secondary outcomes could clearly reflect the study objectives and variables if organized and presented systematically. For instance, the terms reproductive health "behavior" and "outcome" seem to overlap; if this is the case, consider removing one term from the title and text to maintain consistency. Otherwise, please provide a clear definition of each variable and explain how it is measured. Similarly, the term "sexual" may overlap with "reproductive health", as behaviors measured under "sexual behavior" might also fall under reproductive health outcomes. Therefore, please clarify this distinction or consider merging the two concepts where appropriate. A concise, organized, and precise description of variables and outcomes will improve understanding of the study’s objectives and interpretation of results.***

We are sorry for the confusion. “Sexual and reproductive health” is used as an umbrella term in the literature; we agree that it is not clear where the boundaries between “sexual” and “reproductive” are, but think this is not central to our question. For our project, we had a clear theory of change, going from knowledge to behavior and outcomes, and have tried to follow this in the way we present the results. As for the specific variables, we have tried to explain them in the revised Methods section, where we write:

*“The two primary outcomes of the study were reproductive health knowledge and nutritional supplementation in pregnancy. For reproductive health, we used a 15-item questionnaire of true/false statements* *For nutrition knowledge, we analyzed a binary indicator for women (falsely) believing that herbs can cure anemia. For nutritional supplementation during pregnancy, we used self-reported iron supplementation as an outcome measure. Secondary outcomes included contraceptive knowledge (number of methods respondents are familiar with), contraceptive use (currently using any modern method), prenatal care uptake (any, number of visits), haemoglobin level (as a biomarker indicating actual iron intake), body mass index, and birth spacing.*

*For pregnancy related outcomes, we analyzed two separate sets of women: first, women reporting to be pregnant at the time of the endline survey (currently pregnant), and second, women who completed a pregnancy in the year preceding the endline survey (recently pregnant). For current pregnancies, we analyzed self-report iron supplementation as well as self-reported use of antenatal care services, and whether women indicate the pregnancy was planned. For recently completed pregnancies, we analyzed the same variables, but also analyzed pregnancy loss and low birth weight (reported weight at birth < 2.5kgs) as additional secondary outcomes. “*

***Pg. 9/26: Please describe the descriptive and inferential statistical tests used in the study, and specify the statistical methods applied for the presentation and interpretation of the tables.***

We have clarified our main empirical model as follows:

*“Al**l primary models were estimated intent-to-treat, comparing outcomes between treated and control women at endline. For all outcomes, we estimated both unadjusted linear regression models (not including any covariates) and fully adjusted models, where we controlled for age, district, marital status, educational attainment and baseline knowledge variables. The main rationale for using linear regression models was to generate mean differences estimates that could be compared across the rather large set of outcome variables. To adjust for non-normal residuals, we used Huber’s cluster-robust standard errors [15]. “*

***Pg. 11/26: Please mention p-values and place the asterisk indicators (*) next to the p-values only, and not next to other values. Consider this issue for all tables.***

Apologies for the lack of clarity. We have edited the legends of all regression tables to clarify what the asterisks mean – the legend for Table 2 now says:

*“All point estimates displayed correspond to estimated mean differences in outcomes. Numbers in parentheses are cluster-robust standard errors. Asterisks indicate p-value range of estimated differences: *p-value<0.10, **p-value<0.05, ***p-value<0.01.”*

We have added the same information to all other regression tables.

***Pg. 13/26: In Table 4, you referred to “Beliefs that local herbs cure anemia”; however, this variable has not been mentioned in either the primary or secondary outcomes, nor in the measurement instruments described in the supplementary files. Please provide clarification on this issue.***

Apologies for this omission: we have added this to the Methods section now:

*“For nutrition knowledge, we analyzed a binary indicator for women (falsely) believing that herbs can cure anemia.”*

***Pg. 15/26: Since Survey modality is reported at the end of the table, please refrain from repeating it in the outcome rows.***

Good point – we have removed this information from the column heaers.

***Pg. 15-20: Was a specific checklist or questionnaire used to collect the data presented in Tables 5–10? Please clarify this point. If data collection and evaluation were based on a checklist, please describe it in the relevant sections of the manuscript.***

We did not use a checklist – all of these data were collected through a study tool developed for this project and implemented using tablets with the SurveyCTO software.

***Pg. 15-19: The outcomes reported in Tables 5, 7, 8, and 9 may have been influenced by pregnancy and postpartum healthcare recommendations, medication use, or being under the supervision of a physician or nutritionist. Please clarify how these potential confounding factors were addressed in the analyses. If any strategies were implemented to control for them, please describe them explicitly; otherwise, please acknowledge this issue in the limitations section. In addition, it is recommended that the results for pregnant and non-pregnant women be reported separately in these tables.***

We fully agree that these outcomes will be the results of many factors such as health care provider recommendations, but don’t think this is a concern here since treatment is randomized – as long as the randomization worked (which we are very confident here due to the large number of clusters and individual-level randomization), confounding should not be an issue.

As for the subsamples, we have reported them separately in the tables.

***- Not all results have been fully interpreted, and the interpretation provided is rather brief. Please expand the Discussion section to provide a more comprehensive interpretation of the study findings. For instance, the observed reduction in pregnancy loss and low birth weight could be interpreted in relation to the level of antenatal care received. In addition, for each outcome discussed, please contextualize the findings within the existing literature by comparing them with results from similar studies and discussing areas of consistency or discrepancy.***

We have expanded the Discussion section a bit and tried to provide a bit more context, but were not able to say much more on the literature, since we could not find many other studies using a similar approach or technology – please let us know if there are any studies that we missed.

***- Please critically assess the generalization of the study findings and clarify the extent to which the results can be applied to other populations or settings.***

We have added the following paragraph to the Discussion:

*“Even though a more selected sample clearly does not undermine the internal validity of the study (since randomization was done at the individual level), it is not clear whether the final set of women enrolled in the study is fully representative of the larger populations in the four study districts; it is also not clear if the four districts are fully representative of the country as a whole. Since the service is currently only available to Airtel users, this study simply focused on women with access to this service. Even though it seems plausible that similar effects could be found with other phone providers, it is not clear if the results of this study can be generalized to the country as a whole or to other countries.”*
